# Multicenter proteome-wide Mendelian randomization study identifies causal plasma proteins in melanoma and non-melanoma skin cancers

**DOI:** 10.1038/s42003-024-06538-2

**Published:** 2024-07-13

**Authors:** Yajia Li, Qiangxiang Li, Ziqin Cao, Jianhuang Wu

**Affiliations:** 1 Department of Dermatology, Xiangya Hospital, Central South University, Changsha, China; 2 National Clinical Research Center for Geriatric Disorders, Xiangya Hospital, Central South University, Changsha, China; 3 Department of Spine Surgery and Orthopaedics, Xiangya Hospital, Central South University, Changsha, China

**Keywords:** Melanoma, Diagnostic markers

## Abstract

This study addresses the diagnostic and therapeutic challenges in malignant melanoma (MM) and non-melanoma skin cancers (NMSC). We aim to identify circulating proteins causally linked to MM and NMSC traits using a multicenter Mendelian randomization (MR) framework. We utilized large-scale cis-MR to estimate the impact of numerous plasma proteins on MM, NMSC, squamous cell carcinoma (SCC), and basal cell carcinoma (BCC). To ensure robustness, additional analyses like MR Steiger and Bayesian colocalization are conducted, followed by replication through meta-analytical methods. The associations between identified proteins and outcomes are also validated at the tissue level using Transcriptome-Wide Association Study methods. Furthermore, a protein-protein interaction analysis is conducted to explore the relationship between identified proteins and existing cancer medication targets. The MR analysis has identified associations of 13 plasma proteins with BCC, 2 with SCC, and 1 with MM. Specifically, ASIP and KRT5 are associated with BCC, with ASIP also potentially targeting MM. CTSS and TNFSF8 are identified as promising druggability candidates for BCC. This multidimensional approach nominates ASIP, KRT5, CTSS, and TNFSF8 as potential diagnostic and therapeutic targets for skin cancers.

## Abstract (web summary)

This study suggests that the circulating proteins ASIP, KRT5, CTSS, and TNFSF8 are causally associated with melanoma and non-melanoma skin cancer-related traits through a multicenter Mendelian randomization.

## Introduction

Skin cancer is the fifth most common cancer type and rates are predicted to rise sharply over the next 20 years^1–4^. Malignant melanoma (MM) arises when mutations occur in melanocytes. It is highly invasive and metastatic and accounts for around 75% of skin cancer-related deaths despite representing only 4% of all skin cancers^5,6^. Non-melanoma skin cancer (NMSC) develops from the epidermis and may be subdivided into squamous cell carcinoma (SCC) and basal cell carcinoma (BCC). It is the most frequently occurring form of skin cancer and is considered curable at an early stage, although the identification of novel drugs, clinical success, and drug resistance present challenges^7^. On the one hand, traditionally, screening via imaging and pathology tests has had limited effectiveness in early-stage detection. On the other hand, until date, the main treatments for MM and NMSC, including surgery, chemotherapy, radiotherapy, photothermal therapy, etc, are often painful and can have negative consequences, rarely resulting in cancer eradication^8,9^. Current treatments often fail to specifically target cancer cells, resulting in only modest extensions in survival for advanced MM or NSMC patients^1,10–13^. Such therapies frequently impact healthy cells without adequately targeting cancerous ones^13–15^. Skin cancer’s complexity and cellular heterogeneity lead to varied progression and metastasis, driven by the dysregulation at multiple targeting sites. Recent advancements in targeted therapies offer promising alternatives, utilizing drugs that directly inhibit specific molecules or pathways critical to cancer cells, like BRAF^16^. However, the myriad skin cancer subtypes and emerging drug resistances continue to complicate achieving a comprehensive cure with a single therapeutic approach^17,18^. Therefore, developing innovative strategies for early detection and treatment remains critical to addressing these challenges in managing MM and NSMC.

Plasma proteins mediate signaling, transportation, growth, repair, and defense against infections. Many are also cancer biomarkers, enabling early diagnosis, prognostic evaluation, and treatment monitoring^19,20^. Immune checkpoint inhibitors (ICIs) are effective in treating MM and NMSC but only a small proportion of patients respond to them^21–24^. Biomarkers, such as PD-1 and PD-L1, have been identified to indicate the likelihood of a satisfactory response to ICIs but have limited utility^25–28^. Therefore, it is critical to search for more satisfactory predictive immunotherapy biomarkers.

Large-scale proteomics enables simultaneous measurement of thousands of plasma proteins^29^ and genome-wide association studies (GWAS) allow the identification of protein quantitative trait loci (pQTLs)^30^. The current study examined pQTLs and disease variant associations by Mendelian randomization (MR) as a means of identifying plasma protein biomarkers and suitable drug targets^30^. MR is an established genetic epidemiological approach to enhance causal inference of exposure-outcome relationships, reducing potential confounding and reverse causality^31^. The pQTLs were used as instrumental variables (IV) for their corresponding plasma proteins and high-throughput MR analysis was performed to determine causal disease associations^29^. To the best of our knowledge, no prior MR studies have been conducted on integrated GWAS and pQTL data to identify potential drug targets for skin cancer treatment.

**Fig. 1:**
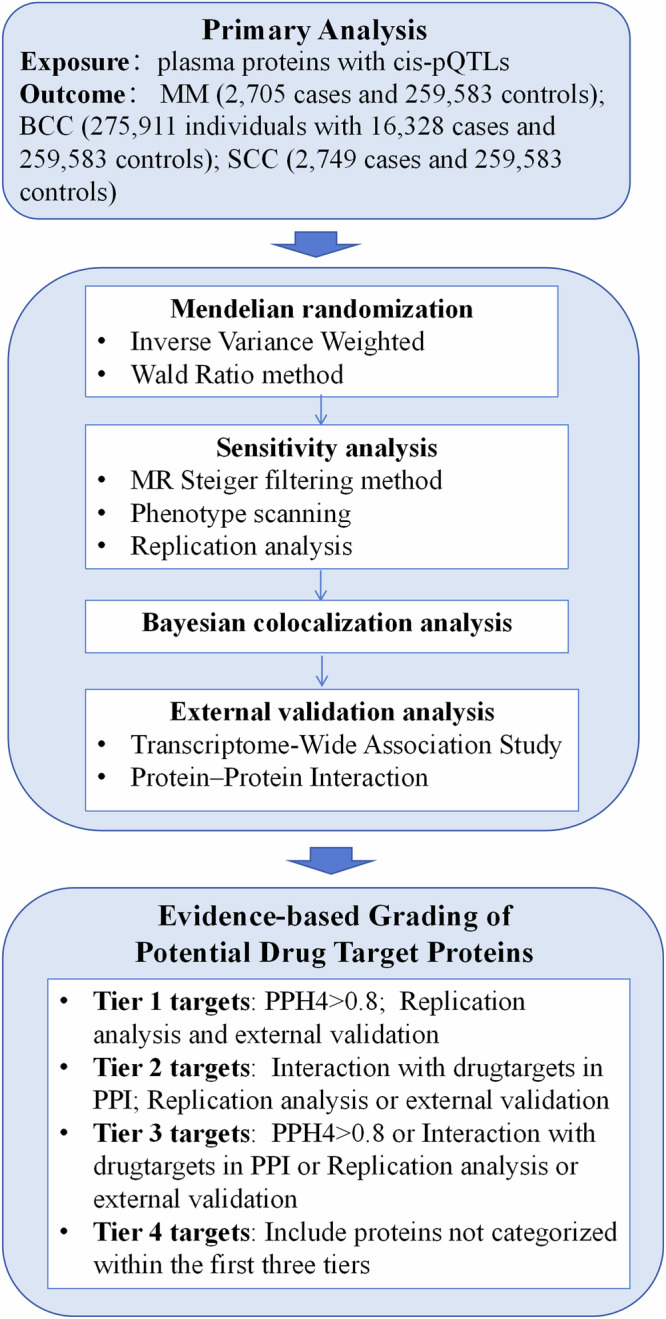
Workflow of Mendelian randomization study revealing causality from plasma protein on skin cancers. MM malignant melanoma, SCC squamous cell carcinoma, BCC basal cell carcinoma, cis-pQTL cis-protein quantitative trait loci, PPH4 posterior probability of hypothesis 4.

Previous studies investigating the association of plasma proteins with MM and NMSC have used low through-put protein measurements with limited sample size or focused on very few plasma proteins, excluding the remaining majority ^32–36^. The current study aimed to identify potential plasma protein drug targets for MM and NMSC treatment by MR analysis. Primary discovery analysis revealed information on proteins correlated with outcomes and sensitivity analyses were conducted to test the robustness of the associations. pQTLs from diverse sources were incorporated into outcome datasets for re-evaluation and meta-analyses were performed to verify the reproducibility of results. Associations between protein targets and outcomes were validated at a tissue level by Transcriptome-Wide Association Study (TWAS) analysis. Results from the DrugBank database were integrated to enable the construction of a protein-protein (PPI) Interaction network combining Bayesian colocalization analysis. The aim was to identify plasma proteins to which a causal influence on MM and NMSC could be assigned and to reveal novel drug targets. A detailed flowchart of the protocol is presented in Fig. 1.

## Results

### Discovery MR analysis

**Fig. 2:**
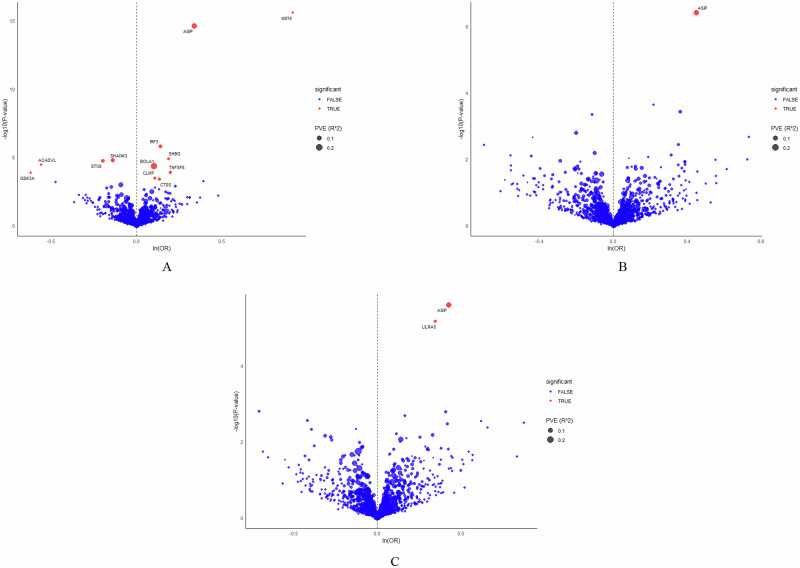
Volcano plot of MR results from the discovery analysis, displaying the associations between 1544 proteins from ARIC and the risk of skin cancer. The increased OR for cancer risk is represented as increments in SD of plasma protein levels. Red dots indicate significant proteins. “ln” refers to the natural logarithm; “PVE” stands for the proportion of variance explained. **A** BCC; **B** MM; **C** SCC. MR Mendelian randomization, ARIC American cohort of the Atherosclerosis Risk in Communities, OR odds ratio, SD standard deviations, MM malignant melanoma, SCC squamous-cell carcinoma, BCC basal cell carcinoma.

**Fig. 3:**
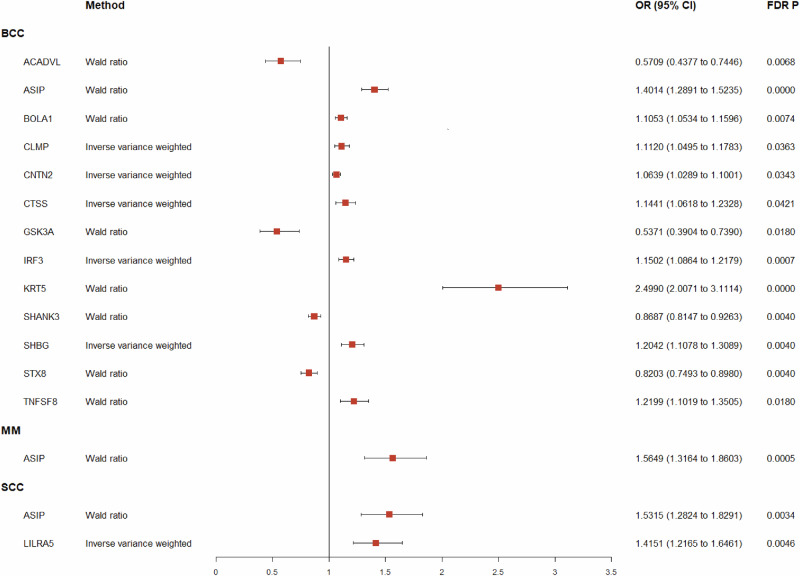
Forest plot of MR results from the discovery analysis. MR Mendelian randomization, MM malignant melanoma, SCC squamous-cell carcinoma, BCC basal cell carcinoma.

A total of 1544 proteins with 2810 SNPs (Supplementary Data 1) with relevance to BCC, SCC, and MM were analyzed by proteome-wide MR, and significant associations were revealed after FDR adjustment (Fig. 2). Thirteen proteins were linked with BCC, 2 with SCC and 1 with MM. Manhattan plots illustrating chromosomal distribution characteristics of the proteins are shown in Supplementary Fig. 1-3. Proteins associated with increased risk of BCC were ASIP (OR: 1.4014, 95% CI: 1.2891 to 1.5235), BOLA1 (OR: 1.1053, CI: 1.0534 to 1.1596), CLMP (OR: 1.1120, CI: 1.0495 to 1.1783), CNTN2 (OR: 1.0639, CI: 1.0289 to 1.1001), CTSS (OR: 1.1441, CI: 1.0618 to 1.2328), IRF3 (OR: 1.1502, CI: 1.0864 to 1.2179), KRT5 (OR: 2.4990, CI: 2.0071 to 3.1114), SHBG (OR: 1.2042, CI: 1.1078 to 1.3089) and TNFSF8 (OR: 1.2199, CI: 1.1019 to 1.3505). Proteins associated with a reduced risk of BCC were ACADVL (OR: 0.5709, CI: 0.4377 to 0.7446), GSK3A (OR: 0.5371, CI: 0.3904 to 0.7390), SHANK3 (OR: 0.8687, CI: 0.8147 to 0.9263) and STX8 (OR: 0.8203, CI: 0.7493 to 0.8980). ASIP (OR: 1.5315, CI: 1.2824 to 1.8291) and LILRA5 (OR: 1.4151, CI: 1.2165 to 1.6461) were associated with SCC and ASIP with MM (OR: 1.5649, CI: 1.3164 to 1.8603) (Table 1). The forest plot is shown in Fig. 3.

**Table 1:** MR analysis results and reverse causality detection for significant proteins identified in the discovery analysis

Proteins	Uniprot ID	Outcomes	Method	nSNP	Cochran’s Q-statistic, *Q* vaule, *P*	OR (95%CI)	*P*	FDR *P*	R2exp	R2out	Steigier *P*	direction test	CAUSE analysis, OR (95%CI), *P*	*F* statistic
ACADVL	P49748	BCC	Wald ratio	1	-	0.5709 (0.4377–0.7446)	3.55E-05	6.80E-03	0.45%	0.01%	5.67E-07	Pass	1.4049 (1.0202, 1.9542),0.1100	32.95
ASIP	P42127		Wald ratio	1	-	1.4014 (1.2891– 1.5235)	2.38E-15	1.60E-12	12.87%	0.02%	2.01E-200	Pass	1.2840 (1.1275, 1.4770),0.0012	1064.99
BOLA1	Q9Y3E2		Wald ratio	1	-	1.1053 (1.0534– 1.1596)	4.41E-05	7.39E-03	16.83%	0.01%	5.19E-282	Pass	-	1459.03
CLMP	Q9H6B4		IVW	2	0.2626, 0.6083	1.1120 (1.0495– 1.1783)	3.25E-04	3.63E-02	10.09%	0.00%	1.41E-160	Pass	1.2586 (0.8437, 1.5068),0.2500	809.29
CNTN2	Q02246		IVW	3	0.1627, 0.9219	1.0639 (1.0289– 1.1001)	2.82E-04	3.43E-02	22.21%	0.00%	0.00E + 00	Pass	0.8353 (0.7788, 0.9048),0.0004	2058.9
CTSS	P25774		IVW	2	3.1257, 0.0771	1.1441 (1.0618– 1.2328)	4.08E-04	4.21E-02	18.27%	0.02%	1.15E-303	Pass	1.1618 (1.1275, 1.1972),0.0015	1611.73
GSK3A	Q2NL51		Wald ratio	1	-	0.5371 (0.3904– 0.7390)	1.34E-04	1.80E-02	0.41%	0.01%	1.68E-06	Pass	1.3499 (0.8607, 1.7160),0.5100	29.94
IRF3	Q14653		IVW	2	0.4097,0.5221	1.1502 (1.0864 to 1.2179)	1.56E-06	6.97E-04	10.68%	0.01%	1.91E-168	Pass	0.9231 (0.8781, 1.1972),0.0512	862.24
KRT5	P13647		Wald ratio	1	-	2.4990 (2.0071– 3.1114)	2.62E-16	3.51E-13	0.71%	0.02%	6.85E-09	Pass	1.1503 (1.0513, 1.2586),0.0087	51.88
SHANK3	Q9BYB0		Wald ratio	1	-	0.8687 (0.8147– 0.9263)	1.71E-05	4.01E-03	6.32%	0.01%	1.90E-96	Pass	1.1388 (0.7261, 1.3499),0.0712	486.07
SHBG	P04278		IVW	2	0.4121, 0.5209	1.2042 (1.1078– 1.3089)	1.26E-05	4.01E-03	3.98%	0.01%	2.59E-59	Pass	0.8106 (0.6376, 1.3364),0.3900	298.73
STX8	Q9UNK0		Wald ratio	1	-	0.8203 (0.7493– 0.8980)	1.80E-05	4.01E-03	3.93%	0.01%	8.83E-59	Pass	1.3499 (0.7634, 1.7683),0.2100	295.32
TNFSF8	P32971		Wald ratio	1	-	1.2199 (1.1019 to 1.3505)	1.28E-04	1.80E-02	2.93%	0.01%	8.85E-44	Pass	1.2214 (1.1388, 1.3100),0.0000	217.55
ASIP	P42127	MM	Wald ratio	1	-	1.5649 (1.3164– 1.8603)	3.85E-07	5.17E-04	12.87%	0.01%	6.98E-206	Pass	1.3364 (1.1503, 1.5068),0.0160	1064.99
ASIP	P42127	SCC	Wald ratio	1	-	1.5315 (1.2824– 1.8291)	2.52E-06	3.39E-03	12.87%	0.01%	1.09E-206	Pass	1.3364 (1.1275, 1.5841),0.0310	1064.99
LILRA5	A6NI73		Wald ratio	1	-	1.4151 (1.2165– 1.6461)	6.82E-06	4.58E-03	2.58%	0.00%	7.17E-39	Pass	0.9048 (0.6570, 1.2712),0.8800	190.68

*OR* odds ratio, *MM* malignant melanoma, *SCC* squamous-cell carcinoma, *BCC* basal cell carcinoma.

### Sensitivity analysis

Steiger filtering confirmed the directionality of these causal relationships and ruled out any reverse associations between the proteins and skin cancer (Table 1).

The CAUSE method was applied to validate the results of the discovery analysis. Only ASIP (OR_CAUSE_: 1.2840, CI: 1.1275–1.4770), KRT5 (OR_CAUSE_: 1.1503, CI: 1.0513–1.2586), CTSS (OR_CAUSE_: 1.1618, CI: 1.1275–1.1972), and TNFSF8 (OR_CAUSE_: 1.2214, CI: 1.1388–1.3100) for BCC, along with ASIP for MC (OR_CAUSE_: 1.3364, CI: 1.1503–1.5068) and SCC (OR_CAUSE_: 1.3364, CI: 1.1275–1.5841), passed the CAUSE test (Table 1). No significant associations were found between the remaining proteins and skin cancer as identified by the CAUSE method, suggesting the possibility of false positives due to pleiotropic effects. Thus, caution is required when interpreting these results.

Potential pleiotropic effects were indicated by phenotype scanning. STX8 has been associated with congenital skin malformations which may account for its link with skin cancer (Supplementary Data 2). Besides that, ASIP was strongly linked with BCC, MM, and other malignant skin neoplasms, and KRT5 with BCC and other malignant skin neoplasms. CLMP was linked to T-cell lymphomas, acute lymphoblastic leukemia, chronic myeloid leukemia, mesothelioma, malignant neoplasm of the rectosigmoid junction, and stomach cancer. CNTN2 was associated with acute lymphoblastic leukemia, B-cell lymphoma, malignant neoplasm of the thyroid gland, and malignant neoplasm of the ureter. TNFSF8 was linked with mesothelioma, secondary malignant neoplasm of retroperitoneum and peritoneum, and mesothelioma. Although there is no direct evidence linking these proteins to known risk factors for skin cancer, the complex etiology of malignancies, which involves genetic predispositions, immune status, and lifestyle factors, suggests that potential pleiotropic effects should also be considered.

**Fig. 4:**
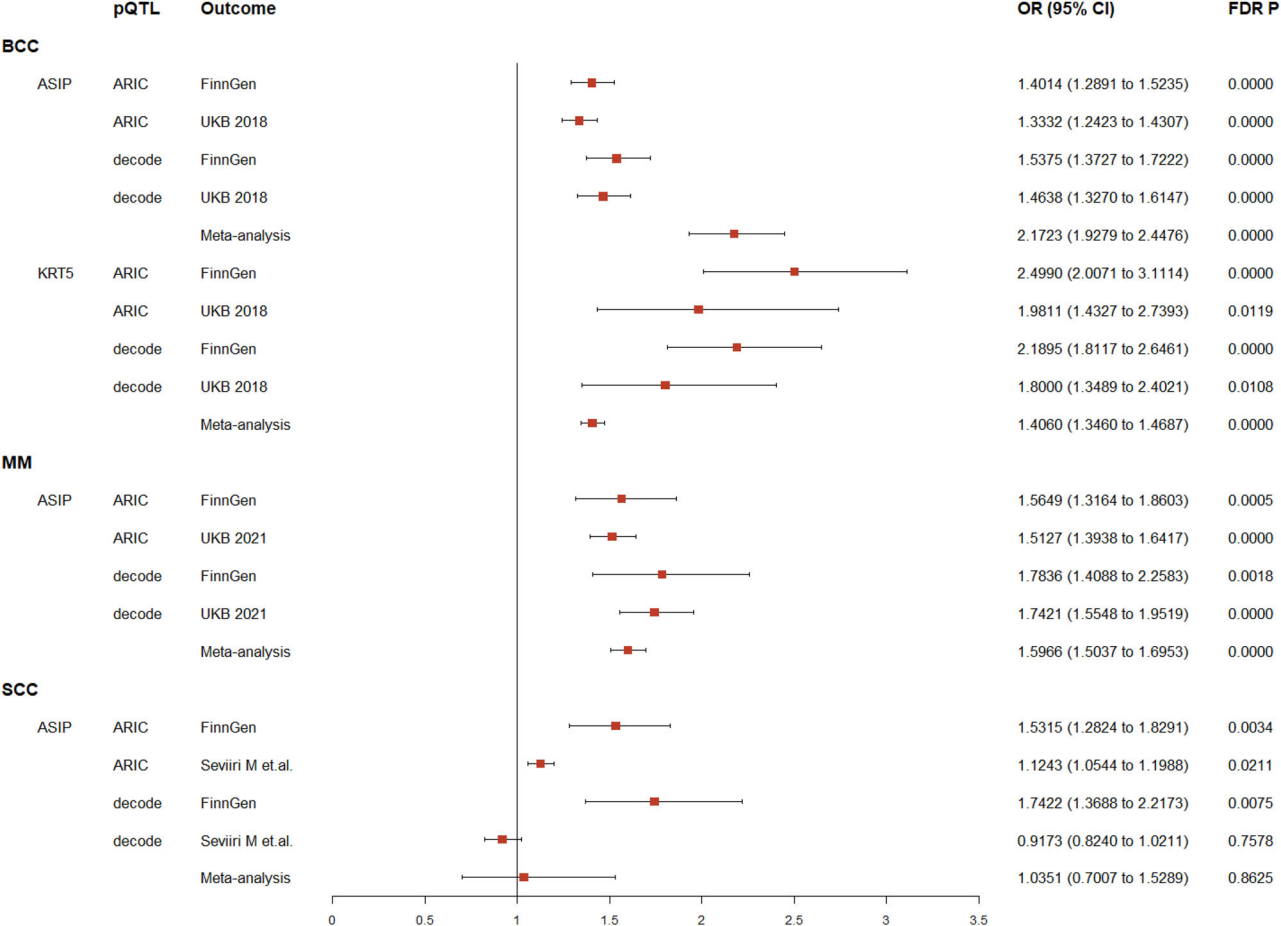
Forest plot showing MR analysis results for proteins that remained significant in the replication analysis, along with results from subsequent meta-analyses. MR Mendelian randomization, MM malignant melanoma, SCC squamous-cell carcinoma, BCC basal cell carcinoma.

We conducted replication analyses on the significant proteins identified in the discovery analysis. Besides the original ARIC and FinnGen combination used in the discovery phase, pairwise combinations of four datasets (including exposure datasets ARIC, deCODE, and outcome datasets FinnGen and UKB) were utilized for replication. A total of 1394 new proteins with 4144 SNPs were identified by a similar IV selection process from deCODE dataset. Three robust associations for ASIP with BCC (*I*^2^ = 42.55%; OR: 1.4176 95% CI: 1.3357–1.5031), KRT5 with BCC (*I*^2^ = 14.71%; OR: 2.1723, 95%CI: 1.9279–2.4476) and ASIP with MM (*I*^2^ = 97.92%; OR: 1.5966, 95%CI: 1.5037–1.6953) were confirmed by meta-analysis. However, all other associations were no longer significant in the replications and the further meta-analysis. BOLA1 was excluded from further analysis due to the failure of replication (Fig. 4 and Table 2).

**Table 2:** Details of the replication analyses and the further meta-analysis

Proteins	OR (95%CI)	FDR *P*	OR (95%CI)	FDR *P*	OR (95%CI)	FDR *P*	*I*^2^	Models	OR (95%CI)	*P*
BCC	ARIC and UKB2018		decode and FinnGen		decode and UKB2018				Meta-analysis	
ACADVL	0.7240 (0.4812–1.0894)	7.86E-01	-	-	-	-	0.00%	Fixed effect	−0.4899 (−0.7127 to −0.2672)	1.63E-05
ASIP	1.3332 (1.2423–1.4307)	1.34E-12	1.5375 (1.3727–1.7222)	6.11014E-11	1.4638 (1.3270 to 1.6147)	1.97E-11	42.55%	Fixed effect	1.4176 (1.3357–1.5031)	5.92E-31
BOLA1	-	-	-	-	-	-	-	-	-	-
CLMP	0.9875 (0.9046–1.0779)	9.93E-01	1.2815 (0.8985–1.8278)	0.839616706	0.9574 (0.8007 to 1.1449)	9.57E-01	59.42%	Random effect	1.0487 (0.9577–1.1483)	3.05E-01
CNTN2	1.0586 (0.9966–1.1244)	6.54E-01	0.9956 (0.9500–1.0435)	0.98988248	-	-	62.62%	Random effect	1.0396 (0.9959–1.0851)	7.63E-02
CTSS	-	-	0.8127 (0.6962–0.9487)	0.31713091	-	-	93.43%	Random effect	0.9710 (0.6947–1.3573)	8.64E-01
GSK3A	0.7949 (0.5224–1.2094)	8.98E-01	-	-	-	-	52.91%	Random effect	0.6375 (0.4355–0.9332)	2.06E-02
IRF3	1.1466 (1.0522–1.2495)	1.92E-01	-	-	-	-	0.00%	Fixed effect	1.1491 (1.0957–1.2051)	1.02E-08
KRT5	1.9811 (1.4327–2.7393)	1.19E-02	2.1895 (1.8117–2.6461)	5.93161E-13	1.8000 (1.3489 to 2.4021)	1.08E-02	14.71%	Fixed effect	2.1723 (1.9279–2.4476)	3.48E-37
SHANK3	0.9491 (0.8479–1.0624)	9.05E-01	1.0781 (0.9740–1.1933)	0.824009245	1.1106 (0.9956 to 1.2388)	8.22E-01	85.84%	Random effect	0.9922 (0.8834–1.1142)	8.95E-01
SHBG	1.0140 (0.8210– 1.2523)	9.97E-01	0.8849 (0.7602–1.0301)	0.79612375	1.0607 (0.8405 to 1.3386)	9.57E-01	77.01%	Random effect	1.0442 (0.9006–1.2105)	5.67E-01
STX8	0.9691 (0.8439– 1.1128)	9.85E-01	-	-	-	-	74.40%	Random effect	0.8840 (0.7514–1.0400)	1.37E-01
TNFSF8	1.1115 (0.9442–1.3084)	8.31E-01	0.7444 (0.6270–0.8838)	0.124096101	0.8353 (0.6387 to 1.0926)	9.52E-01	88.88%	Random effect	0.9698 (0.7665–1.2272)	7.99E-01
MM	ARIC and UKB2021	decode and FinnGen	decode and UKB2018	Meta-analysis						
ASIP	1.5127 (1.3938–1.6417)	5.06E-20	1.7836 (1.4088–2.2583)	0.001782921	1.7421 (1.5548 to 1.9519)	1.29E-18	37.89%	Fixed effect	1.5966 (1.5037–1.6953)	8.76E-53
SCC	ARIC and Seviiri M et.al.	decode and FinnGen	decode and Seviiri et al.	Meta-analysis						
ASIP	1.1243 (1.0544–1.1988)	2.11E-02	1.7422 (1.3688–2.2173)	0.007487227	0.9173 (0.8240 to 1.0211)	7.58E-01	94.53%	Random effect	0.0345 (−0.3556–0.4245)	8.63E-01
LILRA5	0.9742 (0.9451–1.0043)	5.88E-01	-	-	-	-	93.99%	Random effect	−0.1777 (−0.4919–0.1364)	2.68E-01

*OR* odds ratio, *FDR* false discovery rate, *MM* malignant melanoma, *SCC* squamous-cell carcinoma, *BCC* basal cell carcinoma.

### Colocalization analysis and external validation

Analysis with a predefined threshold of PPH4 ≥ 0.8 demonstrated colocalization of proteins and skin cancers. ASIP was colocalized with all three types of skin cancer and STX8, KRT5, GSK3A, CTSS, and TNFSF8 with BCC (Supplementary Figs. 4, 5 and Supplementary Table 2).

Analysis of sun-exposed and non-sun-exposed skin tissues from GTEx gene expression data showed a correlation of four out of the thirteen proteins, CTSS, KRT5, STX8, and ASIP, with BCC. Only ASIP correlated with SCC and MM. The expression levels of these four proteins all showed a significant association with BCC risk. However, over-expression of two proteins, CTSS (Z-score for sun-exposed: −8.0797, *p* value: 0.000; Z-score for non-sun-exposed: −7.8144, *p* value: 0.000) and STX8 (Z-score for sun-exposed: −4.2603, *p* value: 0.000; Z-score for non-sun-exposed: −4.3050, *p* value: 0.000), gave trends contrary to the primary findings. The correlations of ASIP and KRT5 with the three types of skin cancer remained consistent with the initial analysis results (Table 3).

**Table 3:** Assessment of druggability and evidence grading

Proteins	Outcomes	Discovery analysis		Replication analysis	Meta results of replication analysis			CAUSE analysis	Colocalization analysis		External validation			Steigier direction test		PPI network	Levels of Evidence
		OR (95%)	FDR *P*	Pass/Fail	OR (95%)	FDR *P*	Pass/Fail	Pass/Fail	PPH4	Pass/Fail	TWAS Z, P for sun-exposed skin	TWAS Z, P for not-sun-exposed skin	Pass/Fail	*P*	Pass/Fail	Pass/Fail	
ASIP	BCC	1.4014 (1.2891–1.5235)	1.60E-12	Pass	1.4129 (1.3621 to 1.4655)	1.62E-76	Pass	Pass	100.0%	Pass	6.2628, 3.78E-10	5.6136, 1.98E-08	Pass	2.01E-200	Pass	Fail	Tier 1
ASIP	MC	1.5649 (1.3164–1.8603)	5.17E-04	Pass	1.5649 (1.3164– 1.8603)	1.51E-05	Pass	Pass	100.0%	Pass	3.2007, 1.370E-03	3.7430, 1.82E-04	Pass	6.98E-206	Pass	Fail	Tier 1
KRT5	BCC	2.4990 (2.0071–3.1114)	3.51E-13	Pass	1.6781 (1.1011– 2.5575)	1.60E-02	Pass	Pass	98.3%	Pass	4.7248, 2.30E-06	7.8444, 4.35E-15	Pass	6.85E-09	Pass	Fail	Tier 1
CTSS	BCC	1.1441 (1.0618–1.2328)	4.21E-02	Fail	1.0211 (0.8358– 1.2475)	8.38E-01	Fail	Pass	82.4%	Pass	-8.0797, 6.50E-16	-7.8144, 5.52E-15	Pass	1.15E-303	Pass	Pass	Tier 2
TNFSF8	BCC	1.2199 (1.1019–1.3505)	1.80E-02	Fail	0.9904 (0.8281– 1.1845)	9.16E-01	Fail	Pass	81.7%	Pass	NA	NA	Fail	8.85E-44	Pass	Pass	Tier 2
ASIP	SCC	1.5315 (1.2824–1.8291)	3.39E-03	Fail	1.0212 (0.7596– 1.3729)	8.90E-01	Fail	Pass	99.8%	Pass	2.5841, 9.80E-03	3.9707, 7.170E-05	Pass	1.09E-206	Pass	Fail	Tier 3
GSK3A	BCC	0.5371 (0.3904–0.7390)	1.80E-02	Fail	0.8662 (0.4617– 1.6252)	6.55E-01	Fail	Fail	93.0%	Pass	NA	NA	Fail	1.68E-06	Pass	Fail	Tier 4
IRF3	BCC	1.1502 (1.0864–1.2179)	6.97E-04	Fail	1.0629 (0.9077– 1.2448)	4.49E-01	Fail	Fail	69.3%	Fail	NA	NA	Fail	1.91E-168	Pass	Pass	Tier 4
STX8	BCC	0.8203 (0.7493– 0.8980)	4.01E-03	Fail	0.8030 (1.0634– 1.3100)	2.70E-01	Fail	Fail	96.9%	Pass	4.3050, 1.67E-05	4.2603, 2.04E-05	Pass	8.83E-59	Pass	Fail	Tier 4
ACADVL	BCC	0.5709 (0.4377–0.7446)	6.80E-03	Fail	0.8040 (0.4865– 1.3286)	3.95E-01	Fail	Fail	0.0%	Fail	NA	NA	Fail	5.67E-07	Pass	Fail	Tier 4
CLMP	BCC	1.1120 (1.0495–1.1783)	3.63E-02	Fail	1.0274 (0.9548– 1.1054)	4.70E-01	Fail	Fail	13.3%	Fail	NA	NA	Fail	1.41E-160	Pass	Fail	Tier 4
CNTN2	BCC	1.0639 (1.0289–1.1001)	3.43E-02	Fail	1.0019 (0.9485– 1.0583)	9.46E-01	Fail	Fail	1.6%	Fail	NA	NA	Fail	0.00E + 00	Pass	Fail	Tier 4
LILRA5	SCC	1.4151 (1.2165–1.6461)	4.58E-03	Fail	0.9008 (0.7245– 1.1200)	3.47E-01	Fail	Fail	67.2%	Fail	NA	NA	Fail	7.17E-39	Pass	Fail	Tier 4
SHANK3	BCC	0.8687 (0.8147–0.9263)	4.01E-03	Fail	0.9913 (0.9062– 1.0843)	8.48E-01	Fail	Fail	78.5%	Fail	NA	NA	Fail	1.90E-96	Pass	Fail	Tier 4
SHBG	BCC	1.2042 (1.1078–1.3089)	4.01E-03	Fail	1.0186 (0.8985– 1.1547)	7.73E-01	Fail	Fail	1.3%	Fail	NA	NA	Fail	2.59E-59	Pass	Fail	Tier 4
BOLA1	BCC	1.1053 (1.0534–1.1596)	7.39E-03	-	-	-	-	-	68.2%	Fail	NA	NA	Fail	5.19E-282	Pass	Fail	Excluded

*OR* odds ratio, *FDR* false discovery rate, *MM* malignant melanoma, *SCC* squamous-cell carcinoma, *BCC* basal cell carcinoma.

Tier 1 Targets: Include proteins with substantial supportive evidence (PPH4 > 0.8), validated successfully through replication analysis and external TWAS validation.

Tier 2 Targets: Include proteins that are directly associated with known drug targets within the PPI network and have been successfully validated in at least replication analysis or external TWAS validation.

Tier 3 Targets: Include proteins that either demonstrate a PPH4 > 0.8, fulfill the criteria for replication analysis or external TWAS validation, or are solely associated with known drug targets within the PPI network.

Tier 4 Targets: Include proteins not categorized within the first three tiers.

A PPI was constructed to incorporate anti-cancer drug targets and the proteins of interest using data from the DrugBank database to illuminate mechanisms of action (Supplementary Table 3). The BCC-specific PPI network showed interactions of CTSS, IRF3 and TNFSF8 with Cemiplimab and Imiquimod (Interaction Affinity Score (IAS) > 0.4). Strong interactions were observed for CTSS-TLR7 (IAS = 0.706), IRF3-TLR7 (IAS = 0.886) and IRF3-TLR8 (IAS = 0.730). No interactions were found between proteins and drug targets for the MM- and SCC-specific PPI networks (Supplementary Fig. 6–8). Disease-gene association enrichment analysis (FDR *p* < 0.05) indicated associations of ASIP with melanoma, cell type cancer, disease of cellular proliferation and cancer; of KRT5 with basal cell carcinoma, cell type cancer, carcinoma and disease of cellular proliferation and of SHBG with disease of cellular proliferation (Supplementary Fig. 9 and Supplementary Table 4).

### Drug target potential of proteins

Proteins were categorized into four tiers based on colocalization analysis, external validation, and PPI networks (Table 3). The potential for ASIP to be a drug target for the treatment of BCC and MM was found to be excellent, as was the potential of KRT5 for BCC (Tier 1). CTSS and TNFSF8 had good potential for BCC (Tier 2) and other proteins fell into lower tiers (Tier 3 or below).

## Discussion

To the best of our knowledge, the current study is the first to use an integrated approach of MR, Steiger filtering analysis, Bayesian colocalization analyses, TWAS, and PPI to examine the causal associations of 4657 plasma proteins with MM and NNSC. MR analyses revealed associations of 13 plasma proteins with BCC, 2 with SCC, and one with MM which were confirmed by sensitivity and replication analyses of independent data sets. Proteins could be classified into four tiers, according to their potential as drug targets, and the later part of the study focused on tier 1 and 2 targets. ASIP and KRT5 were found to be associated with BCC by genetic prediction and ASIP may also be a potential therapeutic target for MM. CTSS and TNFSF8 also showed good drug target potential for BCC. No tier 1 or 2 targets were identified for SCC. Transcriptomic evidence for associations between proteins and skin cancer risk resulted from TWAS analysis.

Steiger filtering was used to ensure the directionality of causal association and mitigate the effects of reverse causality and horizontal pleiotropy for associations of proteins with MM and NMSC. IVs were restricted to plasma protein cispQTLs to reduce the impact of horizontal pleiotropy. Bayesian colocalization analysis was incorporated to eliminate bias and PPH4 values used to classify proteins. Phenotype scanning revealed that STX8 was associated with skin cancer due to its influence on congenital skin malformations. A PPI network was constructed to illuminate connections between proteins of interest and known drug targets.

Some of the current proteins of interest have previously reported associations with MM. For example, ASIP (Agouti signaling protein) determines mammalian hair pigment production via an interaction with the melanocortin 1 receptor in the hair follicle. MC1R is a G-protein-coupled receptor known to be linked to MM via the UVR stimulation of α-melanocyte stimulating hormone which binds MC1R to enhance melanin synthesis and DNA damage repair^37,38^. ASIP is a competitive inhibitor of the MC1R, preventing α-MSH binding and explaining the link between UV irradiation and skin cancer^39,40^. ASIP has also been identified as an inverse agonist that decreased basal MC1R signaling and inhibited eumelanogenesis^40,41^. Therefore, higher levels of ASIP expression may increase the risk of skin cancer by reducing MC1R activity and melanin production^42^. The current study gives a causal basis to substantiate previous observational studies on the association between ASIP and skin cancer risk.

CD30 (or TNFRSF8) is a type I transmembrane glycoprotein receptor and a member of the tumor necrosis factor receptor superfamily. CD30 expression is restricted to a subset of immunoblasts in the healthy individual^43^ but is also expressed in malignant lymphocytes when Hodgkin lymphoma or anaplastic large cell lymphoma occurs. Its ligand, CD30L (also known as TNFSF8), is a water- and fat-soluble type II transmembrane protein. CD30L binding to CD30 activated the NF-κB pathway, leading to phosphorylation of STAT3 and STAT6 activating cell proliferation and inhibiting apoptosis^44^. CD30L+ mast cells were enriched in the BCC lesional dermis, suggesting mast cell activation during tumorigenesis^45^. Mast cells secrete chemokines, including the inflammatory pre-chemokine IL-8, when challenged with soluble CD30^46^. Indeed, increased IL-8-positive mast cells in BCC lesions may result from CD30L–CD30 inverse signaling interactions^46,47^.

KRT5 encodes keratin 5, an intermediate filament-forming protein expressed in the basal layer of the epithelium where BCC originates. Increased KRT5 expression is a diagnostic marker for BCC, indicating tumor origins in the skin’s basal cell layer^48–50^. Mutations of KRT5 have been linked to severe hereditary skin fragility, such as epidermolysis bullosa (EBS)^50^. Mutant keratins disrupt the intermediate filament cytoskeleton and the resulting stress causes continuous activation of MAP kinase signaling pathways and altered expression of proteins involved in cell-cell and cell–surface adhesion^51,52^. Patients with a severe EBS-Dowling Meara (EBS-DM) phenotype are more likely to develop BCC^53^. Cysteine protease cathepsins, such as CTSS, are involved in extracellular matrix protein degradation, apoptosis, and angiogenesis^54,55^ and increased expression has been observed during premalignant dysplasia leading to epithelial carcinogenesis^56^. Further work is required to clarify the role of CTSS in BCC.

Our analysis identified several proteins (tier 1 or 2) associated with skin cancers that also have potential applications in treating other diseases. Some of these proteins are already targeted by existing or investigational drugs. For instance, brentuximab vedotin, an anti-CD30 antibody-drug conjugate, represents a significant advancement in targeted therapy options^57,58^. Brentuximab vedotin was an antibody-drug conjugate made of a humanized chimeric antibody directly against CD30 covalently bound to a potent microtubule disruptor via a linker that can be cleaved by proteases. The drug could bind to the membrane receptor of CD30, forming a complex with the anti-CD30 drug and enters the cell through receptor-mediated endocytosis, and then the complex fuses with lysozyme, resulting in the aforementioned linker being cleaved by proteases, releasing free drugs in the cytoplasm^59,60^. Phase I and II studies in adults with relapsed/refractory CD30+lymphomas, including ALCL, demonstrated the safety and efficacy of brentuximab vedotin, leading to FDA approval for relapsed/refractory ALCL in adults and successful incorporation into frontline therapies^61^. In an open-label trial, brentuximab vedotin was also identified to improve the severity of cutaneous systemic sclerosis without safety concerns^62^. Besides, LY-3000328, a mall molecule inhibitor of CTSS^63^, could attenuate the degree of liver fibrosis and endostatin in the liver and serum of mice^64^. In the animal model of scarring, LY3000328 treatment reduced the cross-sectional area of scar tissue and effectively inhibited the activation of myofibroblasts, suggesting a reduction in scarring. IRF7 can stimulate the transcriptional activation of CTSS in fibroblasts and alleviate fibrosis, and thus CTSS was found to be pivotal for cutaneous wound healing and may serve as therapeutic targets^65^. Though there is currently a lack of targeted drugs for ASIP or KRT5 and their related proteins, many studies have confirmed their potential as potential targets in some disease therapies. the protein ASIP was associated with MC2R (targeted by corticotropin) and considered to be closely associated with diabetic kidney disease risk^66^. The expression level of the KRT5 gene was significantly lower in metastatic melanoma than in primary melanoma, and it was confirmed in vitro experiments that KRT5 knockdown promoted cell proliferation, migration, and invasion of MM^67^. Our study highlighted the potential of these proteins for future drug development, although further validation and clinical studies are needed to determine their ultimate medicinal value.

The current study has several strengths. First, it is the first and the largest proteome-wide MR study of MM and NMSC and includes many proteins with potential links to skin cancer pathogenesis. Second, TWAS was used to verify risk proteins with independent MR validation analysis. Third, proteins were categorized based on Bayesian colocalization, TWAS, and PPI, to assist future drug discovery. We acknowledge some limitations to the current study. First, proteomic datasets came from individuals with European ancestry and conclusions may not be generalizable to other racial or ethnic groups. Second, TWAS analysis was based on GWAS and pQTL and gave transcriptomic information which may limit the robustness of our conclusions. Future PWAS analysis would illuminate molecular mechanisms involved in MM and NMSC. Third, we employed CAUSE analysis to assess pleiotropy between the proteins and skin cancers. It is important to note that even CAUSE analysis recommends using at least 100,000 variants to estimate parameters accurately. However, the scale of pQTLs currently does not meet this requirement, whether from ARIC, deCODE, or other datasets, which we chose not to use in order to avoid sample overlap. Therefore, until sufficiently large pQTL datasets become available, this limitation remains unresolved. Consequently, results from CAUSE analysis should be interpreted with caution. Lastly, the current conclusions are limited by data available for cancer subtypes, and future study of MM and NSMC subtypes is merited. In vitro and in vivo experiments are required for validation of the current findings.

In conclusion, the MR and Bayesian colocalization analyses were combined to identify potential drug targets for the treatment of MM, BCC, and SCC. TWAS validation and PPI network analysis categorized proteins of interest into four groups of which tier 1 and 2 proteins are the most promising therapeutic targets. Proteins, ASIP, KRT5, CTSS, and TNFSF8, have the potential as diagnostic and therapeutic targets for skin cancers, and validation through future biological experiments is required.

## Methods

### Data sources and selection of IVs

Data from the Atherosclerosis Risk in Communities (ARIC) study, involving 7213 European-descent American participants, provided whole-blood pQTL data for 4657 proteins^68^. Proteomic profiling utilized the SomaScan platform version 4.1. To ensure genetic representativeness, pQTLs were rigorously selected based on specific criteria: 1. Only variants with cis-acting effects within a 1 Mb range of the gene coding for the protein were considered; 2. pQTLs had to reach genome-wide significance with *p* values below 5 × 10^−8^; 3. There was minimal linkage disequilibrium among selected pQTLs (*r*^2^ < 0.001); 4. All chosen pQTLs were located outside the major histocompatibility complex area on chromosome 6 (26–34 Mb). pQTL data from a cohort of 35559 Icelanders with 4907 aptamer-measured plasma protein levels (the Icelandic Cancer Project and deCODE genetics, Reykjavík, Iceland)^30^ were used for replication analysis. SomaScan version 4 (SomaLogic) was used to measure plasma samples. pQTL data was screened under similar conditions for the selection of IVs.

BCC data from 275,911 individuals, including 16,328 cases and 259,583 controls, MM data from 262,288 individuals, including 2705 cases and 259,583 controls, and SCC data from 262,332 individuals, including 2749 cases and 259,583 controls, were obtained from the FinnGen Biobank Analysis Consortium database (https://finngen.gitbook.io/documentation/) with diagnoses according to ICD-10 (International Classification of Diseases) criteria. Summary statistics on skin cancers were also obtained from the UK Biobank and other sources^69^ for external validation. We specifically selected exposure and outcome datasets from different databases and with different genetic backgrounds to avoid the impact of sample overlap. Details of datasets are given in Supplementary Table 1.

### Statistical analysis

#### Mendelian randomization analysis

The Wald ratio was used to generate effect estimates when a plasma protein was instrumented by a single SNP during discovery analysis^70^. The Inverse Variance Weighted (IVW) method^71^ was used for proteins instrumented by two or more SNPs, followed by sensitivity analysis. Results are presented as odds ratios per standard deviation increase in genetically determined plasma proteins. A false discovery rate (FDR) correction^72^ was made to validate MR results through multiple comparisons with correlations having an FDR *p* < 0.05 being considered significant. Effect size is reported as odds ratio (OR) for each standard deviation (SD) increase in genetically predicted protein levels.

#### Sensitivity analysis

Reverse causality between the proteins identified in the discovery analysis and skin cancers was evaluated using the MR Steiger method^73^ and bidirectional MR analysis. IVs used for reverse MR analysis and the Steiger method were sieved based on the same strict selection criteria of pQTL from BCC, SCC, and MM to enhance reliability. A FDR *p* value of <0.05 was also considered to indicate statistical significance in both two analyses. However, bidirectional MR was attempted, but the SNPs remaining in the skin cancer GWAS dataset after selection and clumping were insufficient for harmonization with most of the proteins. As a result, reverse causation was assessed by Steiger filtering alone, and plasma proteins involved in reverse causality were excluded.

The Causal Analysis Using Summary Effect Estimates (CAUSE), a Bayesian posterior probabilities-based MR method, was employed as a sensitivity analysis. This approach enhances statistical power by using summarized full genome-wide results, rather than only significant loci^74^. CAUSE also addresses potential sample overlap effects between exposure and outcome traits, leveraging the maximum sample sizes available for both. Compared to other MR methods, CAUSE is less susceptible to false positive associations due to correlated and uncorrelated horizontal pleiotropy. Associations that do not align with CAUSE results are likely affected by incoherent pleiotropy, leading to a downgrade of such proteins to Tier 4, which indicates no proven potential or unreliable results.

Additionally, heterogeneity was assessed using Cochran’s Q-statistic, with *P* < 0.05 indicating significant heterogeneity. In cases where significant heterogeneity is identified, the results from the random-effect IVW method would be preferred.

Potential links between pQTLs and confounders were investigated via phenotype scanning using the phenoscanner database^75^ with a genome-wide significance threshold of *p* < 5 × 10^−8^. Building on previous research, we believe that ultraviolet exposure (including sunburn, childhood sunburn)^76^, skin types I and II (including phenotypes such as skin color, eye color, hair color)^77^, vitamin D levels^78^, the number and type of moles, past radiation therapy, chemical exposures (history of exposure to arsenic or certain petroleum products), lifestyle factors (such as outdoor work, radiation exposure), and local chronic inflammatory skin diseases (such as long-standing dermatitis, chronic ulcers, or scars) are potential confounding factors related to the risk of skin cancer. pQTLs associated with these risk factors were identified as having pleiotropic effects and data was interpreted with caution.

Significant proteins underwent external validation during replication analysis using the multicenter MR analyses. Alternating validation was performed and genome-wide significant SNPs were used as genetic instruments in separate analyses with two sets of pQTL data and two sets of outcome data from FinnGen and UKB. The stability of causal associations was evaluated by meta-analysis. A value of *I*² above 50% was considered to suggest significant heterogeneity and a random-effects model was adopted^79^.

#### Bayesian colocalization analysis

Colocalization analysis was used to investigate if a specific genetic variant affected both an exposure factor and an outcome through gene expression changes at common loci^80^. This analysis was conducted using the “coloc” R package, setting the default prior probabilities at 1e-4 for any single SNP being linked to each trait (P1 and P2) and 1e-5 for an SNP associated with both traits (P12)^81^. The Bayesian framework assessed four hypotheses: H0, denoting the absence of causal variants for both traits; H1, indicating the presence of a causal variant for trait 1; H2, suggesting a causal variant for trait 2; H3, positing two separate causal variants for traits 1 and 2; and H4, proposing a shared causal variant between the two traits^82^. We deemed significant colocalization between two signals to be manifest when there was compelling evidence, marked by a posterior probability of hypothesis 4 (PPH4) for shared causal variants being ≥0.8^83^.

#### External validation analysis

TWAS was conducted to confirm the correlation of protein-coding genes with the risk of skin cancer at the tissue level^84^. Functional Summary-Based Imputation (FUSION) software enables the construction of predictive models for the influence of genes on phenotype, leveraging GWAS summary statistics to assess relevance to disease, indicating associations between GWAS phenotypes and functional phenotypes. The eQTL reference panel for target skin proteins was derived from the Genotype-Tissue Expression version 8 database.

A PPI network was established to represent interactions among proteins and with targets of pre-existing anticancer drugs. Drug target information relevant to skin cancer was obtained from the DrugBank database. The PPI network was constructed by Search Tool for the Retrieval of Interacting Genes database (STRING)^85,86^ with the threshold for the minimum required interaction score (IAS) of 0.4^87^.

### Evidence-based grading of potential drug targets

Criteria for grading proteins were derived from the methodology described by ref. ^88^.

Tier 1 Targets: proteins with substantial supportive evidence (PPH4 > 0.8) and successfully validated by replication analysis and external TWAS validation.

Tier 2 Targets: proteins directly associated with known drug targets within the PPI network and validated successfully by replication analysis or external TWAS validation.

Tier 3 Targets: proteins that have PPH4 > 0.8, fulfill the criteria for replication analysis or external TWAS validation, or are solely associated with known drug targets within the PPI network.

Tier 4 Targets: proteins not categorized within the first three tiers.

### Statistics and reproducibility

All statistical analyses were performed using R software (version 4.1.3). The Mendelian randomization analysis was carried out using the “TwoSampleMR (version 0.5.6)^73^ ” R package. For the Bayesian colocalization analysis, the “coloc” (version 5.0, available at https://github.com/chr1swallace/coloc) R package was employed. The TWAS analysis utilized the FUSION software (available at https://github.com/gusevlab/fusion_twas), while the PPI network was constructed using STRING (version 11.5). All data used in this study are publicly accessible, and detailed information on sample sizes and sources can be found in Supplementary Table 1.

### Reporting summary

Further information on research design is available in the Nature Portfolio Reporting Summary linked to this article.

### Supplementary information

Supplementary Information

Description of Additional Supplementary Files

Supplementary Data 1

Supplementary Data 2

Reporting Summary

## Data Availability

All analyses were conducted using publicly available data. Detailed sources for each dataset used in our analyses are provided in Supplementary Table 1, which lists the data sources, access links, publication years, identifiers, and the populations studied. The primary datasets that support this study are openly available in UK Biobank at [https://www.ukbiobank.ac.uk/], and FinnGen, at [https://www.finngen.fi/en]. Supplementary Table 1 additionally includes other datasets such as ARIC, deCODE, and datasets utilized for external validation like GTEx and DrugBank. All other data are available from the corresponding author upon reasonable request. Supplementary Data file 1 provides the details of the instrumental variables of plasma proteins used in MR analysis. Supplementary Data file 2 provides the information on previously identified genome-wide significant associations of SNPs, which are used as genetic instruments for potential causal proteins in this study, according to results from the Phenoscanner V2 database. All other data are available from the corresponding author upon reasonable request.

## Code availability

The analysis in this study was conducted using R software (version 4.1.3). The tools used in the analysis, which are publicly available, include: “TwoSampleMR” R package (version 0.5.6), “coloc” R package (version 5.0, available at https://github.com/chr1swallace/coloc), FUSION software (available at https://github.com/gusevlab/fusion_twas), and STRING (version 11.5) tools. No custom algorithms or code were used in this study. For details on the analysis scripts or any specific code used in our study, please contact the corresponding author upon request.

## References

[1] Hasan N (2023). Advanced multifunctional nano-lipid carrier loaded gel for targeted delivery of 5-flurouracil and cannabidiol against non-melanoma skin cancer. Environ. Res..

[2] An S (2021). Indoor tanning and the risk of overall and early-onset melanoma and non-melanoma skin cancer: systematic review and meta-analysis. Cancers.

[3] Tiwari N (2023). Recent progress in polymeric biomaterials and their potential applications in skin regeneration and wound care management. J. Drug Deliv. Sci. Technol..

[4] Siegel RL, Miller KD, Wagle NS, Jemal A (2023). Cancer statistics, 2023. CA Cancer J. Clin..

[5] Elder DE, Bastian BC, Cree IA, Massi D, Scolyer RA (2020). The 2018 World Health Organization classification of cutaneous, mucosal, and uveal melanoma: detailed analysis of 9 distinct subtypes defined by their evolutionary pathway. Arch. Pathol. Lab. Med..

[6] Davis LE, Shalin SC, Tackett AJ (2019). Current state of melanoma diagnosis and treatment. Cancer Biol. Ther..

[7] Hasan N (2023). Formulation and development of novel lipid-based combinatorial advanced nanoformulation for effective treatment of non-melanoma skin cancer. Int. J. Pharm..

[8] Siegel RL, Miller KD, Jemal A (2020). Cancer statistics, 2020. CA Cancer J. Clin..

[9] Overman MJ (2013). Use of research biopsies in clinical trials: are risks and benefits adequately discussed?. J. Clin. Oncol..

[10] Ahmadi SM (2023). Recent advances in novel miRNA-mediated approaches for targeting breast cancer. J. Drug Target.

[11] Sheikh A, Abourehab MAS, Tulbah AS, Kesharwani P (2023). Aptamer-grafted, cell membrane-coated dendrimer loaded with doxorubicin as a targeted nanosystem against epithelial cellular adhesion molecule (EpCAM) for triple-negative breast cancer therapy. J. Drug Deliv. Sci. Technol..

[12] Sonam Dongsar T (2023). Targeted therapy of breast tumor by PLGA-based nanostructures: the versatile function in doxorubicin delivery. Environ. Res..

[13] Zeng H (2022). Melanoma and nanotechnology-based treatment. Front. Oncol..

[14] Dachani SR (2024). A Comprehensive review of various therapeutic strategies for the management of skin cancer. ACS Omega.

[15] Hasan N (2023). Skin cancer: understanding the journey of transformation from conventional to advanced treatment approaches. Mol. Cancer.

[16] Khan, I. & Kashani-Sabet, M. Bromodomain inhibition targeting BPTF in the treatment of melanoma and other solid tumours. *Clin. Exp. Metastasis*, 10.1007/s10585-024-10265-7 (2024). 10.1007/s10585-024-10265-7 38683257

[17] Fateeva A, Eddy K, Chen S (2024). Current state of melanoma therapy and next steps: battling therapeutic resistance. Cancers.

[18] Wang R, Chen Y, Xie Y, Ma X, Liu Y (2024). Deciphering and overcoming anti-PD-1 resistance in Melanoma: a comprehensive review of Mechanisms, biomarker developments, and therapeutic strategies. Int. Immunopharmacol..

[19] Davies MPA (2023). Plasma protein biomarkers for early prediction of lung cancer. EBioMedicine.

[20] Suhre K (2017). Connecting genetic risk to disease endpoints through the human blood plasma proteome. Nat. Commun..

[21] O’Leary, K. Earlier appears better for immunotherapy in melanoma. *Nat. Med.*, 10.1038/d41591-023-00028-4 (2023). 10.1038/d41591-023-00028-4 36928620

[22] Jung J, Heo YJ, Park S (2023). High tumor mutational burden predicts favorable response to anti-PD-(L)1 therapy in patients with solid tumor: a real-world pan-tumor analysis. J. Immunother. Cancer.

[23] Wolchok JD (2022). Long-term outcomes with nivolumab plus ipilimumab or nivolumab alone versus ipilimumab in patients with advanced melanoma. J. Clin. Oncol..

[24] Ascierto PA, Schadendorf D (2022). Update in the treatment of non-melanoma skin cancers: the use of PD-1 inhibitors in basal cell carcinoma and cutaneous squamous-cell carcinoma. J. Immunother. Cancer.

[25] Patel SP, Kurzrock R (2015). PD-L1 expression as a predictive biomarker in cancer immunotherapy. Mol. Cancer Ther..

[26] In GK (2022). Clinical activity of PD-1 inhibition in the treatment of locally advanced or metastatic basal cell carcinoma. J. Immunother. Cancer.

[27] Huang AC, Zappasodi R (2022). A decade of checkpoint blockade immunotherapy in melanoma: understanding the molecular basis for immune sensitivity and resistance. Nat. Immunol..

[28] McGrail DJ (2021). High tumor mutation burden fails to predict immune checkpoint blockade response across all cancer types. Ann. Oncol..

[29] Yazdanpanah N (2022). Clinically relevant circulating protein biomarkers for type 1 diabetes: evidence from a two-sample Mendelian randomization study. Diabetes Care.

[30] Ferkingstad E (2021). Large-scale integration of the plasma proteome with genetics and disease. Nat. Genet..

[31] Sheehan NA, Didelez V, Burton PR, Tobin MD (2008). Mendelian randomisation and causal inference in observational epidemiology. PLoS Med..

[32] Triozzi PL (2022). Circulating immune bioenergetic, metabolic, and genetic signatures predict melanoma patients’ response to anti-PD-1 immune checkpoint blockade. Clin. Cancer Res..

[33] Lebeau S (2005). Comparative analysis of the expression of ERBIN and Erb-B2 in normal human skin and cutaneous carcinomas. Br. J. Dermatol..

[34] Gosman LM, Țăpoi DA, Costache M (2023). Cutaneous melanoma: a review of multifactorial pathogenesis, immunohistochemistry, and emerging biomarkers for early detection and management. Int. J. Mol. Sci..

[35] Cheng B (2020). Discovery of novel and highly potent resorcinol dibenzyl ether-based PD-1/PD-L1 inhibitors with improved drug-like and pharmacokinetic properties for cancer treatment. J. Med. Chem..

[36] Zila N (2023). Proteomic profiling of advanced melanoma patients to predict therapeutic response to anti-PD-1 therapy. Clin. Cancer Res..

[37] Sun Y (2023). AMPK phosphorylates ZDHHC13 to increase MC1R activity and suppress melanomagenesis. Cancer Res..

[38] Guida S, Guida G, Goding CR (2022). MC1R functions, expression, and implications for targeted therapy. J. Investig. Dermatol..

[39] Blanchard SG (1995). Agouti antagonism of melanocortin binding and action in the B16F10 murine melanoma cell line. Biochemistry.

[40] Wolf Horrell EM, Boulanger MC, D’Orazio JA (2016). Melanocortin 1 receptor: structure, function, and regulation. Front. Genet..

[41] Wilson BD (1995). Structure and function of ASP, the human homolog of the mouse agouti gene. Hum. Mol. Genet..

[42] Brudnik U, Branicki W, Wojas-Pelc A, Kanas P (2009). The contribution of melanocortin 1 receptor gene polymorphisms and the agouti signalling protein gene 8818A>G polymorphism to cutaneous melanoma and basal cell carcinoma in a Polish population. Exp. Dermatol..

[43] Stein H (1985). The expression of the Hodgkin’s disease associated antigen Ki-1 in reactive and neoplastic lymphoid tissue: evidence that Reed-Sternberg cells and histiocytic malignancies are derived from activated lymphoid cells. Blood.

[44] Sperling S (2019). Chronic CD30 signaling in B cells results in lymphomagenesis by driving the expansion of plasmablasts and B1 cells. Blood.

[45] Diaconu NC (2007). Increase in CD30 ligand/CD153 and TNF-alpha expressing mast cells in basal cell carcinoma. Cancer Immunol. Immunother..

[46] Fischer M (2006). Mast cell CD30 ligand is upregulated in cutaneous inflammation and mediates degranulation-independent chemokine secretion. J. Clin. Investig..

[47] Wiley SR, Goodwin RG, Smith CA (1996). Reverse signaling via CD30 ligand. J. Immunol..

[48] Depianto D, Kerns ML, Dlugosz AA, Coulombe PA (2010). Keratin 17 promotes epithelial proliferation and tumor growth by polarizing the immune response in skin. Nat. Genet..

[49] Morgan HJ (2020). Hair follicle differentiation-specific keratin expression in human basal cell carcinoma. Clin. Exp. Dermatol..

[50] Zupancic T (2017). Keratin gene mutations influence the keratinocyte response to DNA damage and cytokine-induced apoptosis. Arch. Dermatol. Res..

[51] Liovic M (2008). Dual-specificity phosphatases in the hypo-osmotic stress response of keratin-defective epithelial cell lines. Exp. Cell Res..

[52] Liovic M (2009). Severe keratin 5 and 14 mutations induce down-regulation of junction proteins in keratinocytes. Exp. Cell Res..

[53] Fine JD, Johnson LB, Weiner M, Li KP, Suchindran C (2009). Epidermolysis bullosa and the risk of life-threatening cancers: the National EB Registry experience, 1986-2006. J. Am. Acad. Dermatol..

[54] Kruszewski WJ (2004). Overexpression of cathepsin B correlates with angiogenesis in colon adenocarcinoma. Neoplasma.

[55] Wang B (2006). Cathepsin S controls angiogenesis and tumor growth via matrix-derived angiogenic factors. J. Biol. Chem..

[56] Goldstein MR, Mascitelli L (2017). Might tumor secreted cathepsin proteases leave specific molecular signals in skin, hair and nails years before a cancer becomes clinically apparent?. Med. Hypotheses.

[57] Ong SY, Zain JM (2024). Aggressive T-cell lymphomas: 2024: updates on diagnosis, risk stratification, and management. Am. J. Hematol..

[58] Zhang X (2023). Brentuximab vedotin in treating Chinese patients with lymphoma: a multicenter, real-world study. Cancer Med..

[59] Prince HM, Hutchings M, Domingo-Domenech E, Eichenauer DA, Advani R (2023). Anti-CD30 antibody-drug conjugate therapy in lymphoma: current knowledge, remaining controversies, and future perspectives. Ann. Hematol..

[60] Veyri M, Spano JP, Le Bras F, Marcelin AG, Todesco E (2023). CD30 as a therapeutic target in adult haematological malignancies: where are we now?. Br. J. Haematol..

[61] Agrusa JE, Egress ER, Lowe EJ (2023). Brentuximab vedotin use in pediatric anaplastic large cell lymphoma. Front. Immunol..

[62] Fernández-Codina, A. et al. Brentuximab vedotin for skin involvement in refractory diffuse cutaneous systemic sclerosis, an open-label trial. *Rheumatology*, 10.1093/rheumatology/keae235 (2024). 10.1093/rheumatology/keae235 PMC11879290 38652570

[63] Payne CD (2014). Pharmacokinetics and pharmacodynamics of the cathepsin S inhibitor, LY3000328, in healthy subjects. Br. J. Clin. Pharm..

[64] Zuo T (2023). Macrophage-derived cathepsin S remodels the extracellular matrix to promote liver fibrogenesis. Gastroenterology.

[65] Yin J (2023). IRF7 and CTSS are pivotal for cutaneous wound healing and may serve as therapeutic targets. Signal Transduct. Target. Ther..

[66] Zhang W (2024). Therapeutic targets for diabetic kidney disease: proteome-wide mendelian randomization and colocalization analyses. Diabetes.

[67] Xie R, Li B, Jia L, Li Y (2022). Identification of core genes and pathways in melanoma metastasis via bioinformatics analysis. Int. J. Mol. Sci..

[68] Zhang J (2022). Plasma proteome analyses in individuals of European and African ancestry identify cis-pQTLs and models for proteome-wide association studies. Nat. Genet..

[69] Seviiri M (2022). A multi-phenotype analysis reveals 19 susceptibility loci for basal cell carcinoma and 15 for squamous cell carcinoma. Nat. Commun..

[70] Burgess S, Small DS, Thompson SG (2017). A review of instrumental variable estimators for Mendelian randomization. Stat. Methods Med. Res..

[71] Burgess S, Butterworth A, Thompson SG (2013). Mendelian randomization analysis with multiple genetic variants using summarized data. Genet. Epidemiol..

[72] Benjamini Y, Hochberg Y (2018). Controlling the false discovery rate: a practical and powerful approach to multiple testing. J. R. Stat. Soc. Ser. B (Methodol.).

[73] Hemani G (2018). The MR-Base platform supports systematic causal inference across the human phenome. Elife.

[74] Morrison J, Knoblauch N, Marcus JH, Stephens M, He X (2020). Mendelian randomization accounting for correlated and uncorrelated pleiotropic effects using genome-wide summary statistics. Nat. Genet..

[75] Kamat MA (2019). PhenoScanner V2: an expanded tool for searching human genotype-phenotype associations. Bioinformatics.

[76] Li Y, Wu J, Cao Z (2023). Childhood sunburn and risk of melanoma and non-melanoma skin cancer: a Mendelian randomization study. Environ. Sci. Pollut. Res. Int..

[77] Healy E (2004). Melanocortin 1 receptor variants, pigmentation, and skin cancer susceptibility. Photodermatol. Photoimmunol. Photomed..

[78] Bounas N, Seretis K (2024). Vitamin D and cutaneous melanoma risk: an umbrella review of systematic reviews and meta-analyses. Photobiomodul. Photomed. Laser Surg..

[79] Higgins JP, Thompson SG, Deeks JJ, Altman DG (2003). Measuring inconsistency in meta-analyses. BMJ.

[80] Folkersen L (2017). Mapping of 79 loci for 83 plasma protein biomarkers in cardiovascular disease. PLoS Genet..

[81] Wang G, Sarkar A, Carbonetto P, Stephens M (2020). A simple new approach to variable selection in regression, with application to genetic fine mapping. J. R. Stat. Soc. Ser. B Stat. Methodol..

[82] Giambartolomei C (2014). Bayesian test for colocalisation between pairs of genetic association studies using summary statistics. PLoS Genet..

[83] Chen J (2023). Therapeutic targets for inflammatory bowel disease: proteome-wide Mendelian randomization and colocalization analyses. EBioMedicine.

[84] Gusev A (2016). Integrative approaches for large-scale transcriptome-wide association studies. Nat. Genet..

[85] Szklarczyk D (2023). The STRING database in 2023: protein-protein association networks and functional enrichment analyses for any sequenced genome of interest. Nucleic Acids Res..

[86] Szklarczyk D (2021). The STRING database in 2021: customizable protein-protein networks, and functional characterization of user-uploaded gene/measurement sets. Nucleic Acids Res..

[87] Szklarczyk D (2019). STRING v11: protein-protein association networks with increased coverage, supporting functional discovery in genome-wide experimental datasets. Nucleic Acids Res..

[88] Ren F, Jin Q, Liu T, Ren X, Zhan Y (2023). Proteome-wide mendelian randomization study implicates therapeutic targets in common cancers. J. Transl. Med..

