# Supplementary material for: Multicenter proteome-wide Mendelian randomization study identifies causal plasma proteins in melanoma and non-melanoma skin cancers
Source: Commun Biol. 2024 Jul 13;7:857. doi: 10.1038/s42003-024-06538-2 (PMC11246481; doi:10.1038/s42003-024-06538-2)
Supplement: Supplementary file 1 — Supplementary Information [file 42003_2024_6538_MOESM1_ESM.pdf]

Supplementary Figure 1. Manhattan plot illustrating the chromosomal distribution of proteins for basal cell carcinoma.

Supplementary Figure 2. Manhattan plot illustrating the chromosomal distribution of proteins for melanoma.

Supplementary Figure 3. Manhattan plot illustrating the chromosomal distribution of proteins for squamous cell carcinoma.

Supplementary Figure 4. Colocalization analysis of positive results. Red dots indicate SNPs exhibiting significant combined P-values in both the protein GWAS and cancer GWAS analyses. (A) ASIP with BCC; (B) ASIP with MM; (C) ASIP with SCC; (D) CTSS with BCC; (E) GSK3A with BCC; (F) KRT5 with BCC; (G) TNFSF8 with BCC; (H) STX8 with BCC.

Supplementary Figure 5. Colocalization analysis of negative results. Red dots indicate SNPs exhibiting significant combined P-values in both the protein GWAS and cancer GWAS analyses. (A) ACADVL with BCC; (B) BOLA1 with BCC; (C) CNTN2 with BCC; (D) CLMP with BCC; (E) IRF3 with BCC; (F) SHANK3 with BCC; (G) SHBG with BCC; (H) LILRA5 with BCC.

Supplementary Figure 6. Protein-protein interaction network between identified proteins linked to BCC outcome and cancer-related drug targets. Circles represent proteins, and the lines between them indicate significant associations, with the thickness of the lines denoting the strength of these associations.

Supplementary Figure 7. Protein-protein interaction network between identified proteins linked to MM outcome and cancer-related drug targets. Circles represent proteins, and the lines between them indicate significant associations, with the thickness of the lines denoting the strength of these associations.

Supplementary Figure 8. Protein-protein interaction network between identified proteins linked to SCC outcome and cancer-related drug targets. Circles represent proteins, and the lines between them indicate significant associations, with the thickness of the lines denoting the strength of these associations.

Supplementary Figure 9. Results of disease-gene enrichment analysis. The depth of color represents the strength of enrichment. (A) BCC; (B) MM; (C) SCC.

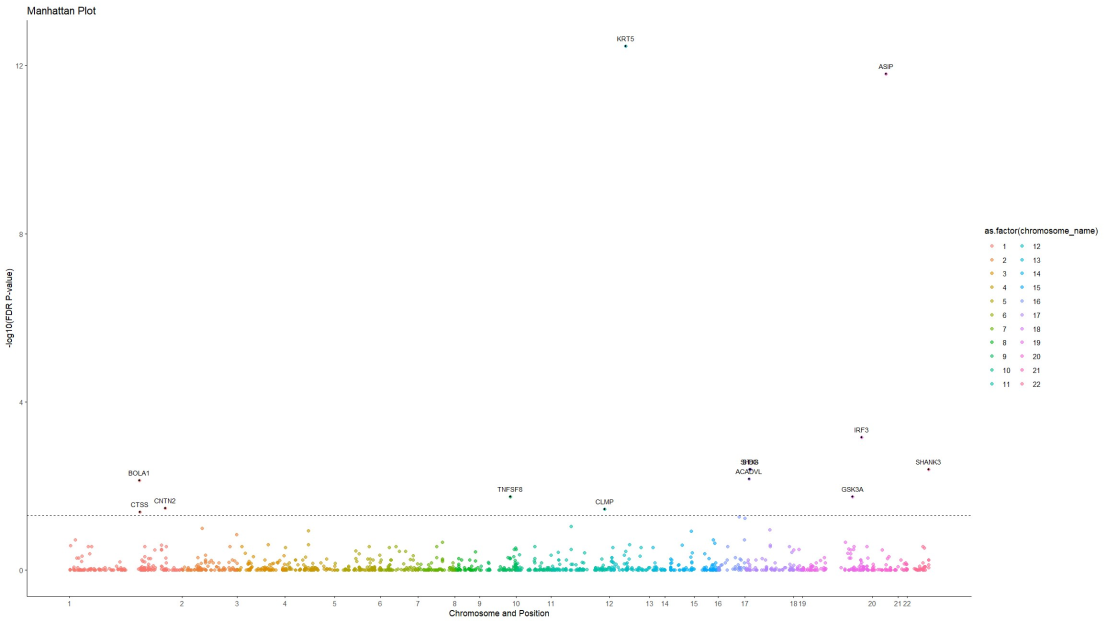

Supplementary Figure 1. Manhattan plot illustrating the chromosomal distribution of proteins for basal cell carcinoma.

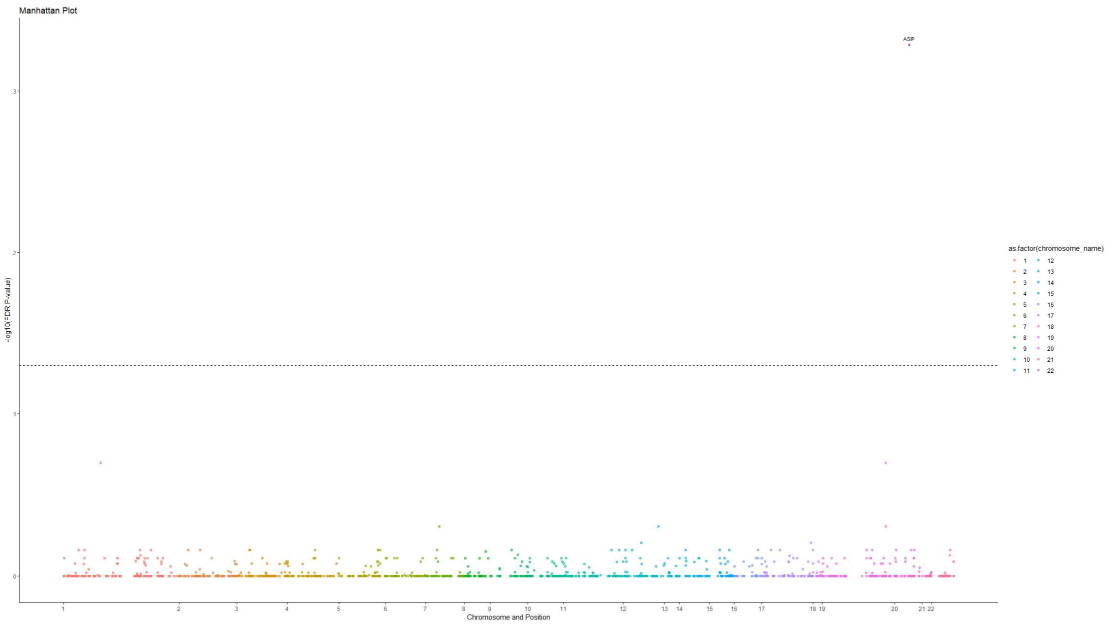

Supplementary Figure 2. Manhattan plot illustrating the chromosomal distribution of proteins for melanoma.

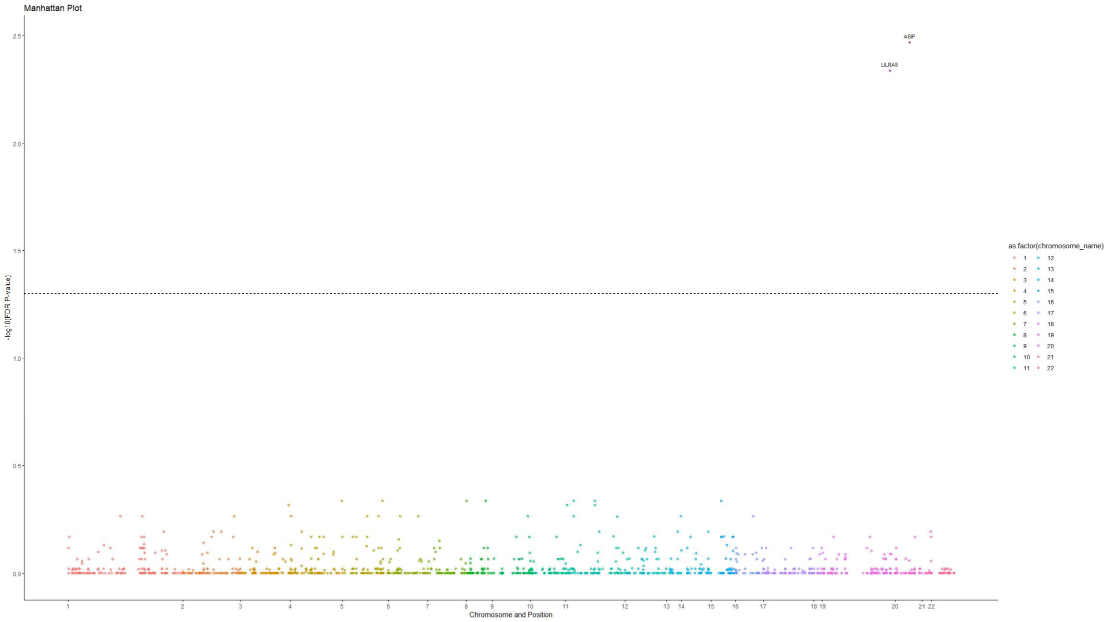

Supplementary Figure 3. Manhattan plot illustrating the chromosomal distribution of proteins for squamous cell carcinoma.

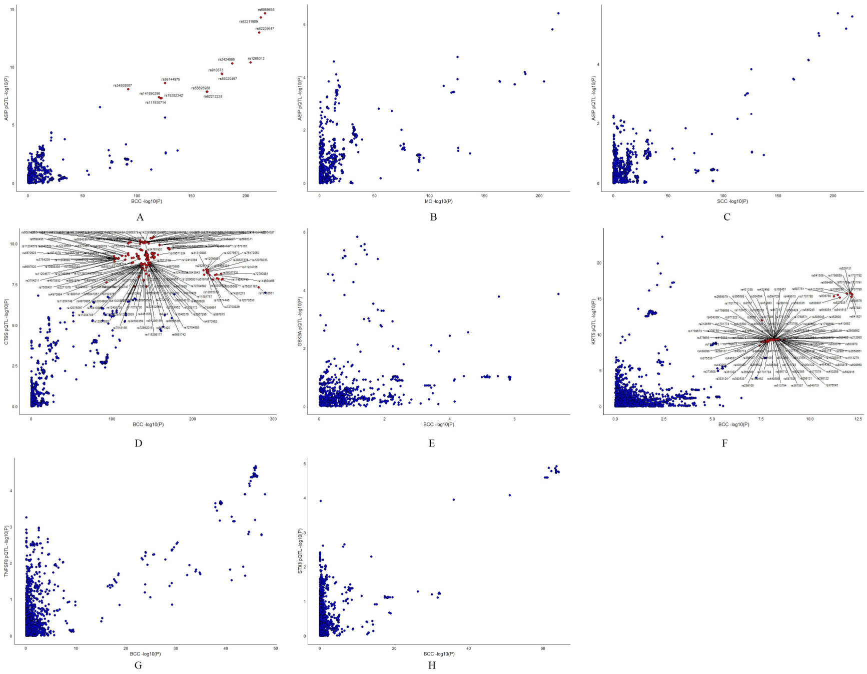

Supplementary Figure 4. Colocalization analysis of positive results. Red dots indicate SNPs exhibiting significant combined P-values in both the protein GWAS and cancer GWAS analyses. (A) ASIP with BCC; (B) ASIP with MM; (C) ASIP with SCC; (D) CTSS with BCC; (E) GSK3A with BCC; (F) KRT5 with BCC; (G) TNFSF8 with BCC; (H) STX8 with BCC.

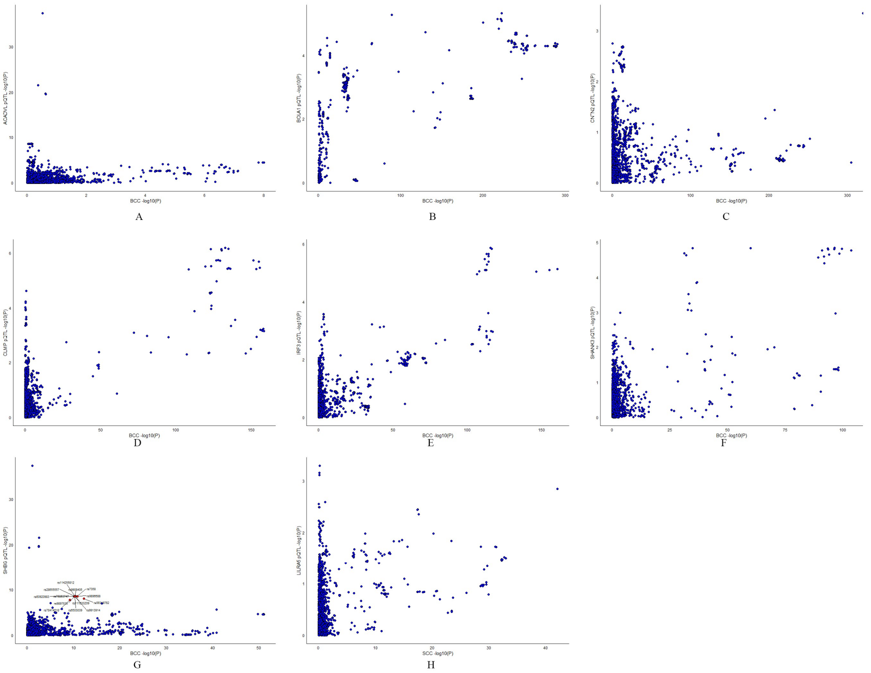

Supplementary Figure 5. Colocalization analysis of negative results. Red dots indicate SNPs exhibiting significant combined P-values in both the protein GWAS and cancer GWAS analyses. (A) ACADVL with BCC; (B) BOLA1 with BCC; (C) CNTN2 with BCC; (D) CLMP with BCC; (E) IRF3 with BCC; (F) SHANK3 with BCC; (G) SHBG with BCC; (H) LILRA5 with BCC.

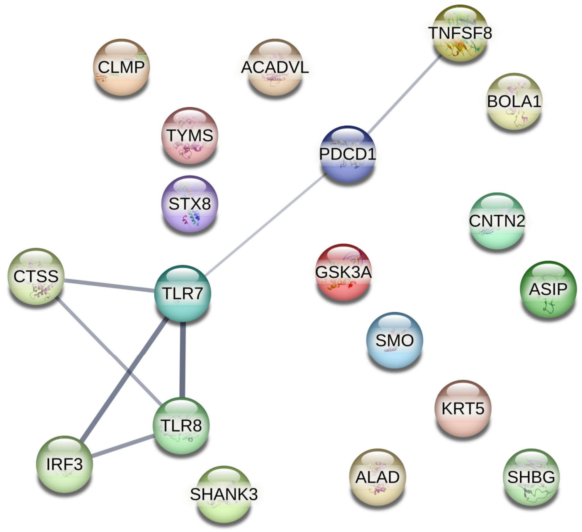

Supplementary Figure 6. Protein-protein interaction network between identified proteins linked to BCC outcome and cancer-related drug targets. Circles represent proteins, and the lines between them indicate significant associations, with the thickness of the lines denoting the strength of these associations.

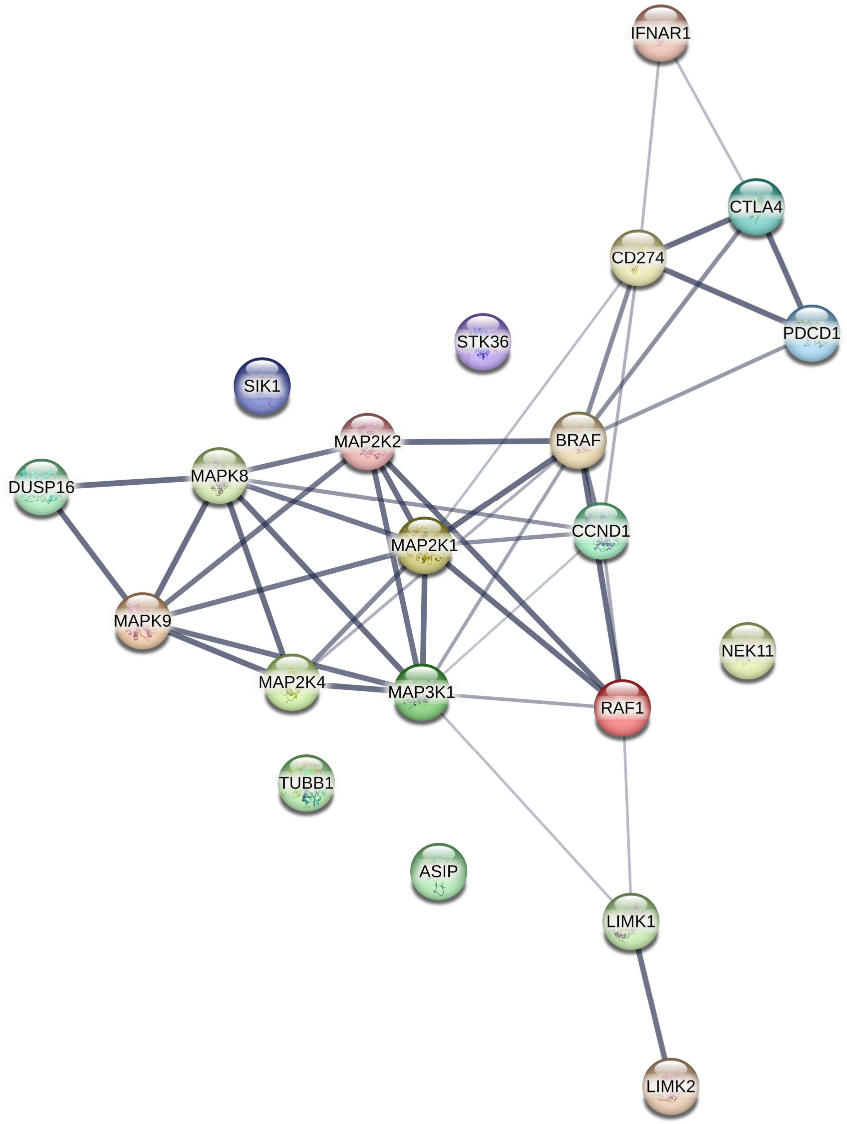

Supplementary Figure 7. Protein-protein interaction network between identified proteins linked to MM outcome and cancer-related drug targets. Circles represent proteins, and the lines between them indicate significant associations, with the thickness of the lines denoting the strength of these associations.

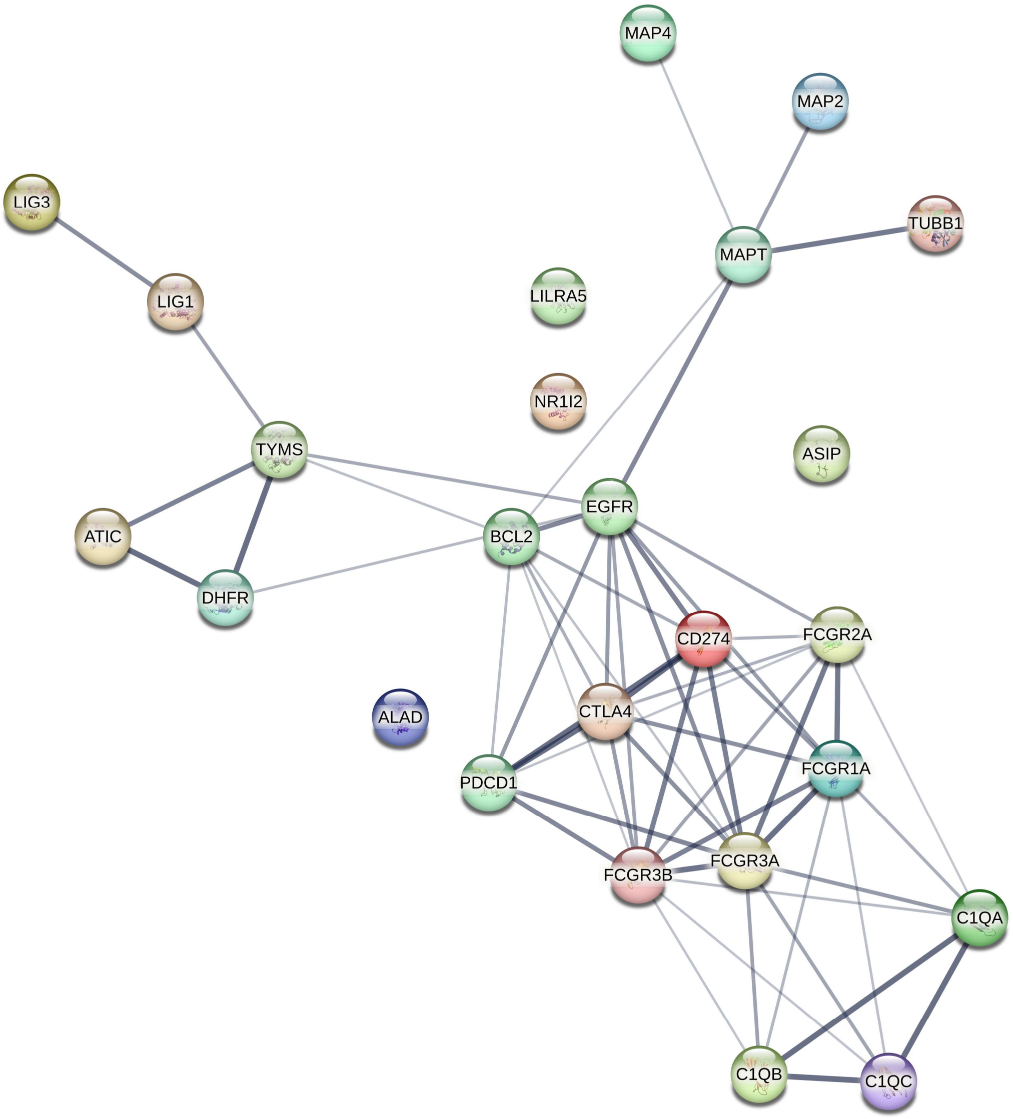

Supplementary Figure 8. Protein-protein interaction network between identified proteins linked to SCC outcome and cancer-related drug targets. Circles represent proteins, and the lines between them indicate significant associations, with the thickness of the lines denoting the strength of these associations.

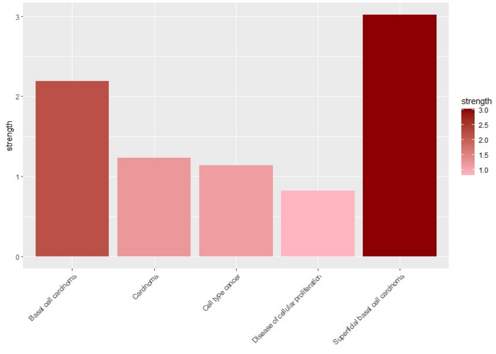

A

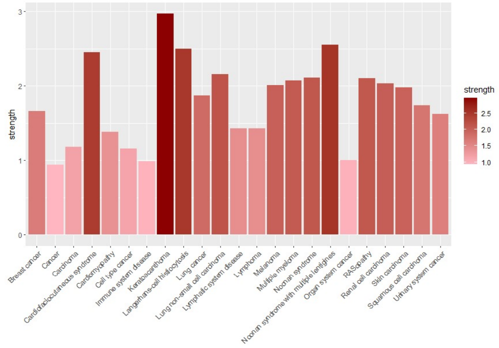

B

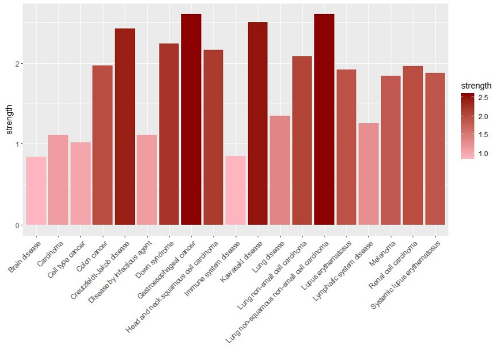

C

Supplementary Figure 9. Results of disease-gene enrichment analysis. The depth of color represents the strength of enrichment. (A) BCC; (B) MM; (C) SCC.

Supplementary Table 1. The sources for all statistical summary datasets used in this study

Usage purposes	Phenotype	Source	Access	Years	ID / name	Population
Discovery analysis	pQTL	ARIC	https://pubmed.ncbi.nlm.nih.gov/	2022	PMID: 35501419	European
	BCC	FinnGen	https://www.finngen.fi/	2021	C3_BASAL_CELL_CARCINOMA_EXALLC	European
	MM	FinnGen	https://www.finngen.fi/	2021	C3_MELANOMA_SKIN_EXALLC	European
	SCC	FinnGen	https://www.finngen.fi/	2021	C3_SQUOMOUS_CELL_CARCINOMA_SKIN_EXALLC	European
Replication analysis	pQTL	deCODE	https://pubmed.ncbi.nlm.nih.gov/	2021	PMID: 34857953	European
	BCC	UKB	www.ukbiobank.ac.uk	2018	ukb-b-8837	European
	MM	UKB	www.ukbiobank.ac.uk	2021	ieu-b-4969	European
	SCC	Seviiri M et.al.	https://pubmed.ncbi.nlm.nih.gov/	2022	PMID: 36496446	European
External validation	TWAS	GTEx	www.gtexportal.org	2020	GTEx version 8	European
	PPI network	Drugbank	https://go.drugbank.com/	-	-	-

Supplementary Table 2. Detailed results of the colocalization analyses.

Proteins	Outcome	$PP.H_0$	$PP.H_1$	$PP.H_2$	$PP.H_3$	$PP.H_4$	PP abf for shared variant
ACADVL	*BCC*	2.67E-33	1.29E-30	2.07E-03	9.98E-01	1.40E-04	0.01%
ASIP	*BCC*	2.49E-229	4.02E-12	6.21E-221	0.00E+00	1.00E+00	100.00%
BOLA1	*BCC*	1.68E-301	2.04E-02	8.08E-303	0.00E+00	6.82E-01	68.20%
CLMP	*BCC*	2.50E-160	5.57E-02	3.64E-159	8.12E-01	1.33E-01	13.30%
CNTN2	*BCC*	2.85E-306	1.10E-06	2.55E-300	9.85E-01	1.55E-02	1.55%
CTSS	*BCC*	8.07E-44	7.77E-02	1.03E-43	9.86E-02	8.24E-01	82.40%
GSK3A	*BCC*	1.99E-03	6.78E-02	2.74E-05	0.00E+00	9.30E-01	93.00%
IRF3	*BCC*	3.05E-53	6.63E-02	4.57E-64	2.27E-107	6.93E-01	69.30%
KRT5	*BCC*	1.73E-20	3.44E-13	8.94E-10	1.68E-02	9.83E-01	98.30%
SHANK3	*BCC*	1.59E-51	1.51E-01	2.04E-172	0.00E+00	7.85E-01	78.50%
SHBG	*BCC*	7.38E-50	2.48E-04	2.94E-46	9.87E-01	1.28E-02	1.28%
STX8	*BCC*	2.04E-61	1.29E-02	3.01E-61	1.80E-02	9.69E-01	96.90%
TNFSF8	*BCC*	7.87E-44	7.71E-02	1.09E-43	1.06E-01	8.17E-01	81.70%
ASIP	*MM*	2.63E-221	4.23E-04	6.21E-221	0.00E+00	1.00E+00	100.00%
ASIP	*SCC*	1.30E-220	2.10E-03	6.20E-221	0.00E+00	9.98E-01	99.80%
LILRA5	*SCC*	4.02E-37	3.28E-01	8.21E-40	0.00E+00	6.72E-01	67.20%

Supplementary Table 3 . Current anticancer medications and corresponding targets for cancer treatment

Medication	DrugBank ID	Status	Target	Gene Name	Uniprot ID	Pharmacological Action	Diseases
Cemiplimab	DB14707	Approved, Investigational	Programmed cell death prote	PDCD1	Q15116	Inhibitor/Antibody	BCC
Vismodegib	DB08828	Approved, Investigational	Smoothened homolog	SMO	Q99835	Antagonist/Inhibitor	
Aminolevulinic acid	DB00855	Approved	Delta-aminolevulinic acid de	ALAD	P13716	Inducer	
Fluorouracil	DB00544	Approved	DNA	NA	NA	Incorporation into and desta	
			RNA	NA	NA	Incorporation into and desta	
			Thymidylate synthase	TYMS	P04818	Unknown	
Imiquimod	DB00724	Approved, Investigational	Toll-like receptor 7	TLR7	Q9NYK1	Agonist	
			Toll-like receptor 8	TLR8	Q9NR97	Unknown	
Sonidegib	DB09143	Approved, Investigational	Smoothened homolog	SMO	Q99835	Antagonist/Inhibitor	
Vemurafenib	DB08881	Approved	Serine/threonine-protein kin	BRAF	P15056	Inhibitor	MM
Dabrafenib	DB08912	Approved, Investigational	Serine/threonine-protein kin	BRAF	P15056	Inhibitor	
			RAF proto-oncogene serine/	RAF1	P04049	Inhibitor	
			Serine/threonine-protein kin	SIK1	P57059	Inhibitor	
			Serine/threonine-protein kin	NEK11	Q8NG66	Inhibitor	
			LIM domain kinase 1	LIMK1	P53667	Inhibitor	
Encorafenib	DB11718	Approved, Investigational	G1/S-specific cyclin-D1	CCND1	P24385	Inhibitor	
			Serine/threonine-protein kin	BRAF	P15056	Inhibitor	
			RAF proto-oncogene serine/	RAF1	P04049	Inhibitor	
			Mitogen-activated protein ki	MAPK8	P45983	Inhibitor	
			Mitogen-activated protein ki	MAPK9	P45984	Inhibitor	
			Mitogen-activated protein ki	MAPK10	P53779	Inhibitor	
			LIM domain kinase 1	LIMK1	P53667	Inhibitor	
			LIM domain kinase 2	LIMK2	P53671	Inhibitor	
			Dual specificity mitogen-act	MAP2K4	P45985	Inhibitor	
			Serine/threonine-protein kin	STK36	Q9NRP7	Inhibitor	
Trametinib	DB08911	Approved	Dual specificity mitogen-act	MAP2K1	Q02750	Inhibitor	
			Dual specificity mitogen-act	MAP2K2	P36507	Inhibitor	
Cobimetinib	DB05239	Approved	Dual specificity mitogen-act	MAP2K1	Q02750	Inhibitor	
Binimetinib	DB11967	Approved, Investigational	Dual specificity mitogen-act	MAP2K1	Q02750	Inhibitor	
			Dual specificity mitogen-act	MAP2K2	P36507	Inhibitor	
Ipilimumab	DB06186	Approved	Cytotoxic T-lymphocyte pro	CTLA4	P16410	Inhibitor	
Pembrolizumab	DB09037	Approved	Programmed cell death prote	PDCD1	Q15116	Inhibitor/Antibody	
			Programmed cell death 1 lig	CD274	Q9NZQ7	Inhibitor/Antibody	
Trametinib	DB08911	Approved	Dual specificity mitogen-act	MAP2K1	Q02750	Inhibitor	
			Dual specificity mitogen-act	MAP2K2	P36507	Inhibitor	
Vindesine	DB00309	Approved, Investigational	Tubulin beta-1 chain	TUBB1	Q9H4B7	Inhibitor	
Nivolumab	DB09035	Approved	Programmed cell death prote	PDCD1	Q15116	Inhibitor/Antibody	
			Programmed cell death 1 lig	CD274	Q9NZQ7	Inhibitor/Antibody	
Peginterferon alfa-2b	DB00022	Approved	Interferon alpha/beta recepte	IFNAR1	P17181	Agonist	

			Interferon alpha/beta recepto	IFNAR2	P48551	Agonist	
Bleomycin	DB00290	Approved, Investigational	DNA	NA	NA	Incorporation into and desta	SCC
			DNA ligase 1	LIG1	P18858	Inhibitor	
			DNA ligase 3	LIG3	P49916	Inhibitor	
Methotrexate	DB00563	Approved	Thymidylate synthase	TYMS	P04818	Inhibitor	
			Bifunctional purine biosynth	ATIC	P31939	Inhibitor	
			Dihydrofolate reductase	DHFR	P00374	Inhibitor	
Ipilimumab	DB06186	Approved	Cytotoxic T-lymphocyte pro	CTLA4	P16410	Inhibitor	
Nivolumab	DB09035	Approved	Programmed cell death prote	PDCD1	Q15116	Inhibitor/Antibody	
			Programmed cell death 1 lig	CD274	Q9NZQ7	Inhibitor/Antibody	
Cemiplimab	DB14707	Approved, Investigational	Programmed cell death prote	PDCD1	Q15116	Inhibitor/Antibody	
Pembrolizumab	DB09037	Approved	Programmed cell death prote	PDCD1	Q15116	Inhibitor/Antibody	
			Programmed cell death 1 lig	CD274	Q9NZQ7	Inhibitor/Antibody	
Cetuximab	DB00002	Approved	Epidermal growth factor rec	EGFR	P00533	Binder	
			Low affinity immunoglobuli	FCGR3B	O75015	Binder	
			Complement C1q subcompo	C1QA	P02745	Binder	
			Complement C1q subcompo	C1QB	P02746	Binder	
			Complement C1q subcompo	C1QC	P02747	Binder	
			Low affinity immunoglobuli	FCGR3A	P08637	Binder	
			High affinity immunoglobul	FCGR1A	P12314	Binder	
			Low affinity immunoglobuli	FCGR2A	P12318	Binder	
Docetaxel	DB01248	Approved, Investigational	Tubulin beta-1 chain	TUBB1	Q9H4B7	Inhibitor	
			Microtubule-associated prot	MAP2	P11137	Inhibitor	
			Microtubule-associated prot	MAP4	P27816	Inhibitor	
			Microtubule-associated prot	MAPT	P10636	Inhibitor	
			Apoptosis regulator Bcl-2	BCL2	P10415	Inhibitor	
			Nuclear receptor subfamily	NR1I2	O75469	Inhibitor	
Aminolevulinic acid	DB00855	Approved	Delta-aminolevulinic acid de	ALAD	P13716	Inducer	

Supplementary Table 4. Detailes of the disease-gene enrichment analysis.

Term ID	Term description	Observed gene count	Background gene count	Strength	False discovery rate	Matching proteins
			BCC			
DOID:2513	Basal cell carcinoma	4	27	2.19	0.0000875	SMO,KRT5,TLR8,TLR7
DOID:0050687	Cell type cancer	6	451	1.14	0.0029	SMO,KRT5,TLR8,PDCD1,TLR7,ASIP
DOID:4300	Superficial basal cell ca	2	2	3.02	0.004	TLR8,TLR7
DOID:305	Carcinoma	5	307	1.23	0.0061	SMO,KRT5,TLR8,PDCD1,TLR7
DOID:14566	Disease of cellular prol	7	1101	0.82	0.0276	SMO,KRT5,TLR8,PDCD1,SHBG,TLR7,ASIP
			MM			
DOID:1909	Melanoma	5	46	2.01	0.00000857	PDCD1,CD274,BRAF,ASIP,CTLA4
DOID:1324	Lung cancer	5	64	1.87	0.0000203	MAP2K1,PDCD1,CD274,BRAF,CTLA4
DOID:3908	Lung non-small cell car	4	26	2.16	0.0000392	PDCD1,CD274,BRAF,CTLA4
DOID:0080690	RASopathy	4	30	2.1	0.0000397	MAP2K2,MAP2K1,RAF1,BRAF
DOID:3451	Skin carcinoma	4	39	1.98	0.0000749	CD274,RAF1,BRAF,CTLA4
DOID:14291	Noonan syndrome with	3	8	2.55	0.0000981	MAP2K1,RAF1,BRAF
DOID:2571	Langerhans-cell histioc	3	9	2.5	0.00012	MAP2K1,MAP3K1,BRAF
DOID:0050687	Cell type cancer	7	451	1.16	0.00014	CCND1,PDCD1,CD274,RAF1,BRAF,ASIP,CTLA4
DOID:0060233	Cardiofaciocutaneous s	3	10	2.45	0.00014	MAP2K2,MAP2K1,BRAF
DOID:162	Cancer	9	978	0.94	0.00014	CCND1,MAP2K1,PDCD1,CD274,MAP3K1,RAF1,BRAF,ASIP,CTLA4
DOID:0050686	Organ system cancer	8	757	1	0.00021	CCND1,MAP2K1,PDCD1,CD274,MAP3K1,RAF1,BRAF,CTLA4
DOID:75	Lymphatic system disea	5	174	1.43	0.00027	CCND1,MAP2K1,CD274,MAP3K1,BRAF
DOID:3490	Noonan syndrome	3	22	2.11	0.00054	MAP2K1,RAF1,BRAF
DOID:3996	Urinary system cancer	4	89	1.62	0.00057	PDCD1,CD274,RAF1,CTLA4
DOID:4450	Renal cell carcinoma	3	26	2.03	0.00077	PDCD1,CD274,CTLA4
DOID:2914	Immune system disease	7	675	0.99	0.0008	CCND1,MAP2K1,PDCD1,CD274,MAP3K1,BRAF,CTLA4
DOID:3149	Keratoacanthoma	2	2	2.97	0.0011	RAF1,BRAF
DOID:305	Carcinoma	5	307	1.18	0.0025	PDCD1,CD274,RAF1,BRAF,CTLA4
DOID:0050700	Cardiomyopathy	4	158	1.38	0.0036	MAP2K2,MAP2K1,RAF1,BRAF
DOID:1749	Squamous cell carcinor	3	51	1.74	0.0037	CD274,RAF1,BRAF
DOID:1612	Breast cancer	3	62	1.66	0.0061	CCND1,MAP3K1,CTLA4
DOID:9538	Multiple myeloma	2	16	2.07	0.0203	CCND1,BRAF
DOID:0060058	Lymphoma	3	105	1.43	0.0233	CCND1,CD274,BRAF
			SCC			

DOID:3908	Lung non-small cell car	4	26	2.08	0.00025	EGFR,PDCD1,CD274,CTLA4
DOID:8857	Lupus erythematosus	4	38	1.92	0.00041	FCGR2A,PDCD1,FCGR3A,CTLA4
DOID:11949	Creutzfeldt-Jakob disea	3	9	2.42	0.00045	C1QB,C1QC,C1QA
DOID:1909	Melanoma	4	46	1.84	0.00045	PDCD1,CD274,ASIP,CTLA4
DOID:850	Lung disease	5	178	1.35	0.0017	EGFR,PDCD1,MAPT,CD274,CTLA4
DOID:0050117	Disease by infectious a	6	368	1.11	0.0027	C1QB,MAPT,C1QC,C1QA,ALAD,DHFR
DOID:4450	Renal cell carcinoma	3	26	1.96	0.0027	PDCD1,CD274,CTLA4
DOID:9074	Systemic lupus erythem	3	31	1.88	0.0034	FCGR2A,PDCD1,CTLA4
DOID:0050687	Cell type cancer	6	451	1.02	0.0056	EGFR,PDCD1,CD274,BCL2,ASIP,CTLA4
DOID:0080374	Gastroesophageal canc	2	4	2.6	0.0066	EGFR,CD274
DOID:0080521	Lung non-squamous no	2	4	2.6	0.0066	EGFR,CD274
DOID:13378	Kawasaki disease	2	5	2.5	0.0078	FCGR2A,FCGR3A
DOID:305	Carcinoma	5	307	1.11	0.009	EGFR,PDCD1,CD274,BCL2,CTLA4
DOID:936	Brain disease	7	806	0.84	0.0103	ATIC,EGFR,C1QB,MAPT,C1QC,C1QA,BCL2
DOID:75	Lymphatic system disea	4	174	1.26	0.0134	FCGR2A,FCGR3A,CD274,BCL2
DOID:14250	Down syndrome	2	9	2.24	0.0155	C1QB,C1QA
DOID:5520	Head and neck squamo	2	11	2.16	0.0209	EGFR,CD274
DOID:2914	Immune system disease	6	675	0.85	0.0256	FCGR2A,PDCD1,FCGR3A,CD274,BCL2,CTLA4
DOID:219	Colon cancer	2	17	1.97	0.0362	EGFR,BCL2
