# Supplementary material for: Multicenter proteome-wide Mendelian randomization study identifies causal plasma proteins in melanoma and non-melanoma skin cancers
Source: Commun Biol. 2024 Jul 13;7:857. doi: 10.1038/s42003-024-06538-2 (PMC11246481; doi:10.1038/s42003-024-06538-2)
Supplement: Supplementary file 2 — Description of Additional Supplementary Files [file 42003_2024_6538_MOESM2_ESM.pdf]

Description of Additional Supplementary Files

File name: Supplementary Data 1
Description: Details of the instrumental variables of plasma proteins used in MR analysis.

File name: Supplementary Data 2
Description: Investigating the Previous Genome-Wide Significant Associations of SNPs as Genetic Instruments for Potential Causal Proteins
