# Supplementary material for: Multicenter proteome-wide Mendelian randomization study identifies causal plasma proteins in melanoma and non-melanoma skin cancers
Source: Commun Biol. 2024 Jul 13;7:857. doi: 10.1038/s42003-024-06538-2 (PMC11246481; doi:10.1038/s42003-024-06538-2)
Supplement: Supplementary file 3 — Supplementary Data 1 [file 42003_2024_6538_MOESM3_ESM.pdf]

Supplementary Data 1 . Detailes of the instrumental variables of plasma proteins used in MR analysis

Proteins	SNP	chr.exposure	pos.exposure	effect_allele.exposure	other_allele.exposure	eaf.exposure	beta.exposure	se.exposure	Pval.exposure	Samplesize.exposure
SIGLEC12	rs36520	19	51442795	A	G	0.181201	-0.122388	0.0215707	1.45E-08	7213
SIGLEC12	rs8113048	19	51503888	T	G	0.153196	1.08903	0.0192155	0	7213
SIGLEC12	rs138536366	19	51619189	C	T	0.114793	0.161595	0.0259376	4.92E-10	7213
SIGLEC12	rs115294736	19	51683077	C	T	0.130875	0.152858	0.0243415	3.59E-10	7213
PLCG2	rs62045706	16	81851810	G	A	0.204353	0.280027	0.0202477	5.91E-43	7213
PLCG2	rs55880431	16	81943852	C	G	0.447317	0.119169	0.0167451	1.21E-12	7213
ACBD6	rs75468761	1	180281203	T	G	0.0432552	-0.331618	0.0403574	2.45E-16	7213
EIF2B1	rs111963801	12	123557234	C	A	0.0148343	-0.554848	0.0680866	4.29E-16	7213
APRT	rs474632	16	88788099	C	T	0.335159	-0.101389	0.0176556	9.70E-09	7213
APRT	rs535070	16	88812323	A	G	0.34015	-0.110548	0.0175576	3.23E-10	7213
ENO2	rs7138740	12	6874600	T	A	0.284972	0.182513	0.0183448	3.59E-23	7213
STAT3	rs4796791	17	42378745	T	C	0.34625	-0.428859	0.0167608	2.72E-138	7213
SMAD2	rs11874858	18	47931447	A	G	0.46534	0.135265	0.0167105	6.69E-16	7213
PDGFRA	rs35597368	4	54273604	T	C	0.109871	-0.282808	0.0263715	1.25E-26	7213
STAT6	rs703817	12	57096045	C	T	0.489602	-0.105123	0.0166196	2.68E-10	7213
ANGPTL3	rs10889333	1	62491359	G	A	0.331623	-0.33608	0.0170489	2.70E-84	7213
MCL1	rs72700845	1	150596273	C	T	0.0128241	-1.31999	0.072826	7.79E-72	7213
SCARA5	rs2085767	8	27863097	A	G	0.116248	0.178269	0.0255653	3.38E-12	7213
SCARA5	rs2685422	8	27947867	G	A	0.291557	-0.253335	0.0181218	7.73E-44	7213
NPDC1	rs71483302	9	137069998	C	T	0.295716	-0.110541	0.0181736	1.24E-09	7213
CLMP	rs7106958	11	123141950	G	A	0.352697	0.110371	0.0173317	2.03E-10	7213
CLMP	rs2302605	11	123194975	C	T	0.442812	-0.436321	0.01583	4.35E-159	7213
TMEM190	rs4806666	19	55376727	C	T	0.456606	-0.828066	0.0135126	0	7213
TMEM190	rs4801651	19	55459766	G	A	0.195342	0.145619	0.0208998	3.51E-12	7213
NUCB1	rs28450126	19	48904758	G	A	0.121309	-0.385366	0.0251079	2.43E-52	7213
RPN1	rs9880064	3	128636666	A	G	0.385692	0.418723	0.016427	2.65E-137	7213
CSF2RB	rs1555015	22	36883930	A	T	0.218841	0.147726	0.0200118	1.73E-13	7213
CSF2RB	rs1534881	22	36933406	G	A	0.455982	-0.695712	0.0144645	0	7213
PTGDS	rs11145948	9	136963460	G	A	0.483571	-0.262711	0.0162539	9.45E-58	7213
MXRA8	rs2765021	1	1362042	C	T	0.0637737	-0.252527	0.033597	6.31E-14	7213
PCDH9	rs4884685	13	66678757	A	C	0.376126	-0.126729	0.0172446	2.22E-13	7213
PCDH9	rs956277	13	66805577	C	T	0.214543	0.135007	0.0203332	3.37E-11	7213
PGLYRP3	rs55991125	1	153307220	C	T	0.0534452	-0.38828	0.0366033	4.27E-26	7213
SLITRK3	rs62282372	3	165194166	C	G	0.124151	-0.570619	0.0242687	7.54E-118	7213
SLITRK3	rs78729408	3	165436289	T	A	0.0136559	0.416428	0.0720578	7.82E-09	7213
HTN3	rs76237622	4	70127829	G	A	0.022806	-0.401352	0.0558659	7.44E-13	7213
APMAP	rs6036977	20	24921032	C	T	0.128726	0.220866	0.0247425	5.50E-19	7213
APMAP	rs78661674	20	24971511	G	A	0.0218356	-0.336762	0.0567146	3.02E-09	7213
MSMB	rs12774026	10	45605081	C	A	0.0171912	-0.388692	0.0639099	1.25E-09	7213
MSMB	rs7091141	10	45652878	T	C	0.169486	0.251683	0.0219022	2.68E-30	7213
MSMB	rs539197038	10	45854864	C	T	0.0741716	-0.428605	0.031351	5.03E-42	7213

MSMB	rs78951441	10	46000727	C	G	0.0177457	-0.547403	0.0627863	3.46E-18	7213
MSMB	rs12569640	10	46055209	G	T	0.132123	-0.418581	0.023916	3.33E-67	7213
MSMB	rs6602880	10	46175087	T	C	0.339872	-0.412648	0.0168838	8.20E-127	7213
MSMB	rs11259826	10	46228813	C	T	0.0356994	0.426819	0.0447819	2.08E-21	7213
MSMB	rs187916528	10	46284579	G	A	0.0338971	-0.403299	0.0456826	1.32E-18	7213
APLP2	rs61349706	11	130094940	A	C	0.0538611	0.409769	0.0365023	5.29E-29	7213
HTATIP2	rs10437608	11	20364060	G	A	0.459448	-0.297298	0.0162591	4.78E-73	7213
GNPTG	rs6600137	16	1357440	A	G	0.106474	0.685275	0.025686	1.25E-149	7213
GNPTG	rs3829564	16	1437018	G	A	0.0632885	-0.266474	0.0338398	3.92E-15	7213
SFTPB	rs1130866	2	85666618	G	A	0.481492	0.516735	0.0154599	7.38E-228	7213
SFTPB	rs4832182	2	85689161	C	T	0.423125	-0.109421	0.0168008	7.87E-11	7213
GNRH2	rs6138990	20	3041972	C	T	0.156384	-0.161103	0.0230146	2.79E-12	7213
ACE	rs139119668	17	63295385	T	C	0.0171219	0.366776	0.064051	1.07E-08	7213
ACE	rs4324	17	63485810	A	G	0.463746	-0.733846	0.0141312	0	7213
ACE	rs3815358	17	63932910	A	G	0.351518	-0.132442	0.0174735	3.90E-14	7213
SYK	rs75328154	9	90790154	C	T	0.244836	-0.197586	0.0191483	8.62E-25	7213
SERPINB1	rs316343	6	2839825	C	T	0.454873	-0.302428	0.0162916	3.51E-75	7213
PROK2	rs7644362	3	71780091	G	A	0.167545	0.785577	0.0204439	4.62E-294	7213
PROK2	rs2322154	3	71804036	A	T	0.444822	0.117697	0.0168227	2.86E-12	7213
CLEC4G	rs115293707	19	7729723	G	C	0.073825	0.542015	0.0310012	4.55E-67	7213
SERPINH1	rs688727	11	75544665	A	C	0.269721	-0.104411	0.0186216	2.14E-08	7213
SERPINH1	rs584961	11	75566583	A	G	0.105573	0.213548	0.0269593	2.71E-15	7213
SERPINH1	rs599816	11	75582937	G	A	0.462152	-0.211427	0.0165167	4.10E-37	7213
PILRA	rs12878	7	100149507	G	A	0.395467	-0.322072	0.016668	3.79E-81	7213
PILRA	rs35305377	7	100341332	G	A	0.445168	0.76748	0.0141316	0	7213
PILRA	rs2553022	7	100804240	A	G	0.235824	0.190456	0.0192348	5.73E-23	7213
SMPD1	rs11040883	11	6388560	G	A	0.152017	-0.299479	0.0228978	1.19E-38	7213
SMPD1	rs1050239	11	6394233	G	A	0.231318	-0.605083	0.0184173	9.89E-221	7213
LINGO3	rs117849041	19	2312904	C	T	0.106336	-0.166257	0.027002	7.80E-10	7213
B4GALT6	rs113222817	18	31636351	T	G	0.0383336	-1.51803	0.0395506	1.89E-293	7213
B4GALT6	rs144333451	18	31885600	T	G	0.0259254	0.462427	0.0520751	8.33E-19	7213
HHIP	rs11727676	4	144737912	T	C	0.0907389	0.299585	0.0287735	3.30E-25	7213
SIGLEC15	rs515373	18	45826966	G	A	0.483363	0.136359	0.0168132	5.89E-16	7213
EBI3	rs4807558	19	4257186	G	A	0.41141	-0.672107	0.0149489	0	7213
EBI3	rs4807575	19	4287128	T	C	0.429364	0.240912	0.0165551	2.64E-47	7213
OSMR	rs1697890	5	38864561	T	C	0.478164	0.114405	0.0167181	8.38E-12	7213
OSMR	rs357277	5	38931817	G	A	0.392416	-0.204229	0.0169662	4.68E-33	7213
STX8	rs35082231	17	9437887	C	T	0.238805	0.329143	0.0191529	6.66E-65	7213
NTM	rs12800878	11	131246794	G	A	0.385138	-0.302827	0.0168116	5.45E-71	7213
NTM	rs2511781	11	131328850	C	T	0.426244	0.574718	0.0154717	1.60E-276	7213
GALNT13	rs67335822	2	154010909	G	T	0.157216	0.145814	0.0228428	1.84E-10	7213
PLA2R1	rs3828327	2	159885584	G	A	0.452378	0.652967	0.0150661	0	7213
PLA2R1	rs147994001	2	160089369	C	T	0.0237765	0.579049	0.0545542	3.99E-26	7213

PLA2R1	rs138152291	2	160220553	G	T	0.0216276	-0.411445	0.0569007	5.29E-13	7213
PLA2R1	rs72998719	2	160271143	A	G	0.0357688	0.329306	0.0444944	1.50E-13	7213
GNGT2	rs35638197	17	49207373	T	C	0.0558713	-0.221329	0.0364152	1.28E-09	7213
CYB5D2	rs17176322	17	4153082	G	A	0.390129	0.254739	0.0167682	2.49E-51	7213
CYB5D2	rs140231030	17	4280059	G	T	0.0215583	-0.820399	0.0567535	1.04E-46	7213
CD58	rs60612523	1	116517087	A	G	0.0165673	0.704774	0.0646769	1.95E-27	7213
SRL	rs11640587	16	4214222	C	G	0.377651	0.187831	0.0169797	3.23E-28	7213
PLD3	rs4803321	19	40237362	A	C	0.0366699	-0.55044	0.0438729	9.83E-36	7213
SPINK7	rs9784645	5	148337418	G	T	0.265285	0.293556	0.0184713	6.30E-56	7213
UCMA	rs10796041	10	13222896	C	T	0.435672	0.169732	0.0168232	8.87E-24	7213
UCMA	rs1537771	10	13241177	C	T	0.147581	-0.440307	0.0230104	1.18E-79	7213
PDXK	rs2186924	21	43708287	G	C	0.383405	0.132496	0.0171726	1.37E-14	7213
PDXK	rs148593140	21	43863088	G	C	0.0128934	-0.748543	0.0733369	2.70E-24	7213
REG4	rs2298069	1	119811396	C	T	0.140094	-0.259668	0.0237392	1.24E-27	7213
HSPB1	rs10255591	7	76117091	T	C	0.193817	0.325435	0.020792	2.51E-54	7213
HSPB1	rs574196569	7	76281659	G	A	0.0284209	-0.300368	0.0500721	2.08E-09	7213
HSPB1	rs28584236	7	76298731	T	C	0.26785	-0.788506	0.0164161	0	7213
CHI3L1	rs74475064	1	203111545	T	G	0.0608623	0.278275	0.0346083	1.04E-15	7213
CHI3L1	rs35405821	1	203181771	C	G	0.473243	0.830486	0.0135907	0	7213
CHI3L1	rs147096276	1	203206772	C	T	0.0133093	0.485363	0.0725197	2.35E-11	7213
CHI3L1	rs7541299	1	203247362	C	A	0.0411063	0.270147	0.0416726	9.61E-11	7213
CHI3L1	rs75356477	1	203279299	A	C	0.0287675	0.290218	0.0498019	5.87E-09	7213
CHI3L1	rs77214546	1	203323737	G	A	0.0405518	0.311624	0.0419548	1.23E-13	7213
SVEP1	rs10116674	9	110369372	G	A	0.0355608	0.571835	0.0444517	1.84E-37	7213
SVEP1	rs138764012	9	110393715	G	A	0.0122002	-0.5006	0.075628	3.87E-11	7213
SVEP1	rs61751937	9	110549951	G	C	0.0300846	1.03657	0.0471196	7.26E-104	7213
SVEP1	rs191541154	9	110552023	C	T	0.0284209	-0.352123	0.0498988	1.86E-12	7213
SPATA20	rs112815427	17	50516409	T	C	0.0453348	0.224962	0.0400419	2.00E-08	7213
SPATA20	rs8076632	17	50548567	C	G	0.369749	-0.676147	0.0152265	0	7213
SPATA20	rs142230609	17	50778190	G	A	0.0282129	-0.41033	0.0500057	2.69E-16	7213
MTRF1L	rs3757067	6	152984732	G	A	0.177804	-0.123684	0.0216502	1.16E-08	7213
ANGPTL1	rs10913568	1	178544276	C	T	0.478442	0.435634	0.0158255	1.06E-158	7213
ANGPTL1	rs871632	1	178934631	T	C	0.2831	0.227769	0.0180073	2.76E-36	7213
KIAA1549L	rs2753408	11	33378145	G	A	0.469638	-0.545943	0.0154196	2.58E-253	7213
KIAA1549L	rs77101501	11	33631478	C	T	0.111049	0.202352	0.0262735	1.52E-14	7213
KLK13	rs2569459	19	51036176	C	T	0.352697	-0.393619	0.0168497	2.08E-116	7213
KRT5	rs636676	12	52513634	G	C	0.277138	-0.134218	0.0186345	6.50E-13	7213
SVEP1	rs1327532	9	110364034	A	C	0.329336	0.141675	0.0176516	1.17E-15	7213
SVEP1	rs1889322	9	110447224	C	T	0.451199	0.21795	0.0165034	2.30E-39	7213
SVEP1	rs61751937	9	110549951	G	C	0.0300846	1.10566	0.0469013	1.96E-118	7213
CLEC12A	rs2922162	12	9966674	T	C	0.45626	0.790066	0.0139253	0	7213
CLEC12A	rs11053548	12	10018128	A	G	0.24435	-0.44362	0.0186665	2.90E-120	7213
TINAGL1	rs9425884	1	31571326	T	C	0.463746	-0.0943742	0.0165451	1.22E-08	7213

COL6A3	rs1050785	2	237324109	C	A	0.401705	-0.220663	0.0167889	5.20E-39	7213
COL6A3	rs10929229	2	237376075	A	G	0.247886	0.119244	0.0189678	3.43E-10	7213
NAGPA	rs12922333	16	4968368	G	A	0.270692	-0.151533	0.018909	1.29E-15	7213
NAGPA	rs12599777	16	5029465	A	G	0.190628	-0.574707	0.0201233	4.75E-170	7213
TBCE	rs3856245	1	235408113	G	A	0.346943	0.403095	0.0168155	3.01E-122	7213
TBCE	rs10926189	1	235597517	C	T	0.305351	-0.111637	0.0179525	5.30E-10	7213
TXNDC5	rs73719371	6	7897914	A	G	0.0901844	-0.320533	0.0287804	1.41E-28	7213
TXNDC5	rs149742777	6	7909587	T	A	0.0127547	0.927539	0.0734425	3.50E-36	7213
TXNDC5	rs72829254	6	7915821	C	T	0.0376404	-0.262324	0.0430684	1.18E-09	7213
FGFBP3	rs78398688	10	91897439	T	C	0.0962152	0.224746	0.0283501	2.57E-15	7213
FGFBP3	rs11186737	10	91906592	C	T	0.309649	-0.600419	0.0165673	2.34E-264	7213
TBL2	rs76029572	7	73578528	C	G	0.0372245	0.461667	0.0440668	1.69E-25	7213
PCOLCE	rs7385804	7	100638347	C	A	0.368779	-0.225072	0.0170842	3.49E-39	7213
ASL	rs34942108	7	65854580	G	T	0.105989	-0.197902	0.0272033	3.83E-13	7213
ASL	rs28412533	7	66531410	C	G	0.161237	0.21655	0.0221654	2.09E-22	7213
UROS	rs1935451	10	125816857	G	A	0.085332	0.270185	0.0297457	1.34E-19	7213
QDPR	rs35216955	4	17525490	A	G	0.237072	-0.738647	0.0174205	0	7213
QDPR	rs17564585	4	17561747	G	A	0.0255788	0.343294	0.0525287	6.78E-11	7213
SELPLG	rs73191242	12	108620180	G	A	0.191321	-0.355148	0.0209511	3.12E-63	7213
SELPLG	rs7298509	12	108638368	T	A	0.446693	-0.130684	0.0167784	7.71E-15	7213
GSTT2B	rs5751777	22	23924860	C	T	0.4094	0.146477	0.0169234	6.00E-18	7213
CYB5A	rs6566789	18	74266264	A	G	0.280882	-0.147174	0.0186349	3.26E-15	7213
NT5C2	rs12257472	10	103173692	C	T	0.304728	0.155142	0.0179904	7.89E-18	7213
NT5C2	rs79780963	10	103192742	C	T	0.0849854	-0.229234	0.0299464	2.19E-14	7213
TNR	rs2235256	1	175599911	A	G	0.418342	-0.218953	0.0166152	3.33E-39	7213
CREB3L4	rs4845586	1	153970121	T	G	0.474213	0.288777	0.0163483	2.16E-68	7213
PCBD1	rs72816586	10	70826163	C	T	0.0402745	-0.714534	0.041627	9.25E-65	7213
ARHGAP25	rs13413887	2	68769536	A	G	0.232636	-0.308586	0.0194601	1.08E-55	7213
ARHGAP25	rs2311420	2	68817396	A	T	0.437613	-0.143984	0.0165865	4.81E-18	7213
TALDO1	rs10902210	11	733639	G	A	0.0962845	0.159003	0.0281311	1.64E-08	7213
RRM1	rs1662161	11	4115744	A	G	0.459795	0.104714	0.0165221	2.47E-10	7213
TYMP	rs131805	22	50525724	T	C	0.221475	-0.362562	0.019519	2.87E-75	7213
ADH5	rs62325239	4	99066341	A	G	0.0381949	-0.980676	0.0421683	2.00E-115	7213
ADH5	rs7375649	4	99094995	G	A	0.281159	0.221387	0.0183132	2.54E-33	7213
ADH7	rs17529509	4	99430279	C	A	0.100305	-0.447271	0.0272598	1.99E-59	7213
SYK	rs10993706	9	90840685	A	G	0.0747262	-0.371118	0.0312663	3.39E-32	7213
BLVRA	rs10229520	7	43790865	T	G	0.332663	-0.241779	0.0174539	4.37E-43	7213
WFDC2	rs6032226	20	45485639	C	T	0.489949	-0.101732	0.016718	1.22E-09	7213
FAH	rs11555096	15	80180184	C	T	0.0213503	-1.65648	0.0542398	9.54E-193	7213
FAH	rs1437222	15	80193822	T	A	0.143006	-0.156264	0.0237401	4.96E-11	7213
PDLIM1	rs1328599	10	95294018	G	T	0.0817968	0.451264	0.0300558	3.38E-50	7213
RECQL	rs74626198	12	21543317	T	C	0.0273118	-0.390423	0.0505161	1.23E-14	7213
PYGL	rs12717412	14	50935708	G	A	0.37474	-0.302739	0.0168641	1.57E-70	7213

GALK1	rs60521621	17	75765315	T	C	0.13441	0.161404	0.0241468	2.49E-11	7213
CAPZA1	rs6700673	1	112614432	C	T	0.475669	0.136276	0.0167032	3.96E-16	7213
VPS4A	rs9924894	16	69333482	T	G	0.24747	0.133523	0.0190733	2.78E-12	7213
ALDH3A1	rs887241	17	19742625	A	C	0.340496	-0.216368	0.0171714	5.02E-36	7213
DYNLL2	rs9902118	17	58093234	C	T	0.325731	-0.3846	0.0171764	2.05E-107	7213
DYNLL2	rs117940828	17	58166282	G	C	0.014765	-0.433695	0.0690155	3.49E-10	7213
APOL1	rs9610469	22	36257706	G	C	0.315749	-0.425378	0.0173578	1.84E-127	7213
APOL1	rs3886200	22	36267581	C	T	0.456814	0.10435	0.0167606	5.06E-10	7213
CD59	rs831630	11	33721473	C	T	0.309025	-0.394336	0.0172189	4.17E-112	7213
CD59	rs10836121	11	33848167	C	T	0.469707	0.113532	0.0166781	1.08E-11	7213
FABP1	rs2241883	2	88124547	T	C	0.337793	-0.282662	0.0171942	1.20E-59	7213
HMBS	rs72995454	11	119060830	C	T	0.0275198	0.448389	0.0510729	2.03E-18	7213
C10orf54	rs10762476	10	71771220	C	A	0.143144	0.584715	0.0228588	3.25E-138	7213
C10orf54	rs4747217	10	71905893	A	G	0.487037	0.106998	0.0164503	8.33E-11	7213
FKBP1B	rs13034709	2	23934696	C	G	0.172258	0.278776	0.0218313	6.08E-37	7213
FKBP1B	rs72781698	2	24048636	C	T	0.205878	-0.630194	0.0190058	1.62E-224	7213
MAP1LC3B	rs9972730	16	87384100	G	A	0.43484	-0.124283	0.0166605	9.68E-14	7213
DAPP1	rs3822103	4	99833508	G	T	0.470609	-0.343316	0.0161009	6.98E-98	7213
TAGLN2	rs2789422	1	159922298	G	A	0.423957	-0.252272	0.0164524	3.03E-52	7213
CHST9	rs2020157	18	26924940	G	T	0.367184	-0.174481	0.0170351	1.88E-24	7213
CHST9	rs11660451	18	27118770	T	C	0.321919	0.304102	0.0175253	4.12E-66	7213
SMAP1	rs2462507	6	70635945	A	T	0.302717	-0.750098	0.015846	0	7213
SMAP1	rs13212771	6	70820998	T	G	0.156315	-0.185406	0.0228566	5.82E-16	7213
SMAP1	rs4267918	6	71130403	G	A	0.0381949	0.284759	0.043179	4.56E-11	7213
EVL	rs34612764	14	99946845	G	A	0.346319	0.161853	0.0173698	1.54E-20	7213
EVL	rs1190972	14	100078063	T	C	0.106336	-0.225969	0.026725	3.33E-17	7213
CLINT1	rs12284	5	157787179	A	G	0.136767	-0.246025	0.0238108	7.48E-25	7213
AGFG1	rs11679534	2	227575661	C	T	0.296964	0.413824	0.0172538	2.26E-122	7213
GGA3	rs7222782	17	75275977	T	A	0.0860252	-0.209533	0.0294109	1.15E-12	7213
CRABP2	rs3806412	1	156706761	G	T	0.388743	-0.54454	0.0157623	3.74E-242	7213
LCN1	rs12340697	9	135513873	C	T	0.151809	-0.20562	0.0229118	3.58E-19	7213
ARHGAP1	rs11039024	11	46901617	C	T	0.138431	-0.138597	0.0240227	8.28E-09	7213
S100A2	rs58056804	1	153552230	G	A	0.0521281	-0.309332	0.0369879	7.27E-17	7213
CBL	rs4938644	11	119282649	G	A	0.0713295	-0.709014	0.0315185	2.31E-108	7213
BPGM	rs4732044	7	134656210	G	A	0.486621	0.112028	0.016776	2.60E-11	7213
RPIA	rs11686263	2	88811341	G	A	0.386524	0.185536	0.0170896	3.01E-27	7213
SHMT1	rs2688030	17	18359730	A	G	0.349854	-0.648341	0.0158074	0	7213
EGFLAM	rs2006889	5	38264633	A	G	0.496187	-0.104831	0.0164378	1.91E-10	7213
EGFLAM	rs2434498	5	38351387	G	T	0.231249	-0.198109	0.0195248	4.98E-24	7213
AARS	rs2070203	16	70269677	G	A	0.490018	0.108285	0.0165976	7.30E-11	7213
CCM2	rs7810512	7	45110732	A	C	0.265424	0.107052	0.0187901	1.27E-08	7213
SARS2	rs1808661	19	38933313	G	A	0.382989	0.113394	0.0170513	3.14E-11	7213
SRI	rs76354035	7	88224953	G	C	0.203521	0.28658	0.0206045	2.03E-43	7213

APOF	rs2066818	12	56360038	C	A	0.0680715	-0.602174	0.0322579	5.51E-76	7213
CBR1	rs16993864	21	36074301	C	A	0.0188548	-1.4996	0.0580213	6.08E-141	7213
RNPEP	rs2678208	1	201853976	T	G	0.0962152	-0.412362	0.0278018	4.77E-49	7213
RNPEP	rs6702334	1	201985696	T	A	0.31471	0.536648	0.0168623	4.24E-208	7213
PDLIM4	rs4877	5	132271895	G	T	0.0924026	-0.449204	0.0281706	2.77E-56	7213
DARS2	rs57808485	1	173356642	C	T	0.14259	-0.153855	0.0239382	1.38E-10	7213
DARS2	rs12564699	1	173708900	G	A	0.284625	-0.301577	0.0182009	1.51E-60	7213
HIBCH	rs291462	2	190316952	T	C	0.404062	-0.823854	0.0138004	0	7213
HIBCH	rs56101840	2	190416306	A	C	0.062526	0.257501	0.0344615	8.82E-14	7213
CCDC50	rs147604673	3	191389569	C	T	0.0171912	-0.55557	0.0642744	6.63E-18	7213
MAX	rs762810	14	65077649	C	A	0.343962	-0.39197	0.0166978	1.74E-117	7213
CACYBP	rs16847450	1	174793184	A	G	0.116664	-0.703685	0.0246472	5.98E-170	7213
GSTO1	rs11191922	10	104094626	A	T	0.127132	0.145852	0.02486	4.64E-09	7213
GSTO1	rs11191970	10	104245971	G	T	0.295023	-0.922594	0.014741	0	7213
GSTO1	rs1571455	10	104600971	A	G	0.0822127	-0.247768	0.0302117	2.80E-16	7213
GSTO1	rs1334608	10	104687920	C	T	0.4878	-0.148302	0.016558	4.20E-19	7213
PPIH	rs78676038	1	42663404	T	C	0.0356994	0.299979	0.0448328	2.38E-11	7213
PLEKHA1	rs10788274	10	122301287	G	A	0.472896	0.22619	0.0163057	3.35E-43	7213
MAPRE1	rs80061974	20	32500132	G	C	0.129696	0.170729	0.0246385	4.59E-12	7213
MAPRE1	rs414049	20	32831982	T	C	0.286219	-0.517638	0.0171113	2.30E-189	7213
CLIC5	rs35822882	6	45949262	G	T	0.0234299	-1.27056	0.0531232	1.01E-121	7213
CLIC5	rs1555216	6	46010441	C	A	0.460488	0.104475	0.0165599	2.98E-10	7213
GLRX2	rs143131184	1	193166834	C	T	0.0171219	1.04886	0.0635432	4.15E-60	7213
GLRX2	rs12141336	1	193231175	A	G	0.051019	-0.248356	0.037759	5.12E-11	7213
GABARAPL2	rs2454867	16	75522922	T	C	0.116872	-0.303645	0.0257063	6.65E-32	7213
TAX1BP3	rs160589	17	3668569	G	A	0.024539	0.488345	0.0540253	2.00E-19	7213
VPS24	rs59613942	2	86552964	A	G	0.154721	0.307012	0.0228898	1.56E-40	7213
PDCD5	rs11669629	19	32593444	G	C	0.273673	0.682782	0.0167301	0	7213
PDCD5	rs73035385	19	32832001	C	T	0.052544	-0.261634	0.0370713	1.85E-12	7213
PDCD5	rs149248211	19	32838194	C	T	0.023222	-0.368605	0.0549114	2.06E-11	7213
RAD23B	rs11573709	9	107322974	G	A	0.228268	0.18305	0.0195203	8.87E-21	7213
SAT2	rs13894	17	7626584	G	A	0.06925	-0.317648	0.032488	1.94E-22	7213
SAT2	rs1050541	17	7657517	T	G	0.459032	-0.100574	0.0167652	2.08E-09	7213
CALCOCO2	rs318100	17	48876268	T	G	0.160543	-0.281609	0.0223358	4.57E-36	7213
SMTN	rs7287178	22	31043611	C	T	0.287259	0.126388	0.0183937	6.90E-12	7213
HPGDS	rs7692683	4	93894993	G	A	0.36254	0.120414	0.0171386	2.32E-12	7213
HPGDS	rs111816772	4	94172298	C	T	0.0104672	0.459701	0.0809551	1.41E-08	7213
HPGDS	rs10033662	4	94318311	G	A	0.372453	-0.571691	0.0158114	3.10E-263	7213
UBASH3B	rs3937027	11	122655065	C	A	0.248371	-0.367631	0.0188611	1.67E-82	7213
NT5C	rs78625720	17	75144846	G	A	0.0258561	-1.52849	0.0490837	7.35E-200	7213
TNFAIP8	rs1032859	5	119305605	T	A	0.119437	-0.490494	0.024938	6.18E-84	7213
TNFAIP8	rs79272926	5	119390942	C	A	0.226189	-0.216994	0.0198281	1.17E-27	7213
CCT5	rs11557652	5	10256060	A	T	0.0207265	-0.516992	0.0583311	9.68E-19	7213

ARL3	rs72847755	10	102535686	T	C	0.016498	0.491096	0.0648024	3.94E-14	7213
ARL3	rs8354	10	102676884	C	T	0.0913628	-0.741442	0.0278268	2.87E-149	7213
ARL3	rs11191401	10	102813646	A	G	0.298489	0.231782	0.0180009	1.59E-37	7213
TRIM3	rs2344827	11	6464439	A	G	0.167267	-0.149853	0.0223323	2.09E-11	7213
MTHFD1	rs10146204	14	64352051	G	A	0.415985	-0.189337	0.0168838	6.03E-29	7213
MTHFD1	rs1542312	14	64538148	T	C	0.306253	0.12395	0.0181102	8.32E-12	7213
GCA	rs17783344	2	162352383	T	G	0.133024	-0.5921	0.0235545	9.73E-134	7213
FERMT3	rs7948329	11	64139829	G	A	0.430403	-0.142634	0.0167957	2.44E-17	7213
PSMB1	rs3734763	6	170576824	T	C	0.411757	-0.529913	0.0158832	4.34E-227	7213
PPM1A	rs183404450	14	60238965	T	C	0.03972	-0.270711	0.0425316	2.07E-10	7213
LANCL2	rs7786168	7	55364530	T	C	0.349092	-0.118414	0.0173217	8.80E-12	7213
CRAT	rs3124499	9	129106712	A	G	0.289339	-0.256183	0.018144	1.13E-44	7213
INPP5B	rs14614	1	37803125	A	G	0.136906	-0.91618	0.021556	0	7213
INPP5B	rs72663618	1	38034753	G	C	0.0180923	-0.424919	0.06234	1.01E-11	7213
ARRB1	rs504683	11	75340189	A	G	0.293775	0.5338	0.0172021	1.48E-198	7213
RPE	rs2723211	2	210015768	G	A	0.453487	0.147632	0.0166034	7.51E-19	7213
KLC1	rs2273175	14	103693804	T	C	0.323167	0.110967	0.0174702	2.26E-10	7213
OLA1	rs10930634	2	174077998	C	T	0.197768	0.697832	0.019166	1.31E-266	7213
ECH1	rs2229259	19	38816463	C	T	0.108693	0.703511	0.0254121	2.10E-160	7213
DARS	rs2304371	2	135803987	G	A	0.170872	0.418086	0.0211557	1.06E-84	7213
FLII	rs145840264	17	18258631	G	T	0.0252322	0.611659	0.0519322	9.88E-32	7213
CCBL2	rs12130865	1	88930764	T	A	0.497227	-0.186871	0.0165222	2.06E-29	7213
SCIN	rs7803810	7	12568345	G	A	0.424026	-0.106183	0.0167774	2.62E-10	7213
DECR1	rs1805806	8	90005517	T	C	0.360044	-0.193941	0.0170808	1.26E-29	7213
PIP4K2A	rs7098978	10	22855287	C	G	0.289824	-0.481854	0.0174734	3.10E-159	7213
NEK7	rs142662761	1	198041651	A	G	0.0105365	-0.960715	0.0806259	1.97E-32	7213
AP1G2	rs35309020	14	23554817	G	A	0.0900458	-0.446406	0.0289956	1.20E-52	7213
PTGFRN	rs12036504	1	116812722	T	C	0.0471371	-0.317503	0.0389185	3.98E-16	7213
PTGFRN	rs4546904	1	116945089	T	A	0.0921253	-1.22341	0.0246776	0	7213
PTGFRN	rs12239381	1	116976139	C	T	0.0825593	-0.63182	0.0294645	6.15E-99	7213
PLEKHA7	rs397373	11	16864091	C	T	0.106336	0.389561	0.0266412	9.69E-48	7213
ACYP2	rs3930909	2	53977785	C	T	0.0684875	-0.180148	0.0328814	4.43E-08	7213
ACYP2	rs11125524	2	54152396	T	C	0.494246	0.383966	0.015985	1.02E-122	7213
TESC	rs2393124	12	117055665	C	G	0.347983	-0.357116	0.0170976	4.12E-94	7213
ECI2	rs7757606	6	4119470	G	A	0.307847	-0.445632	0.0171826	7.30E-142	7213
DDX6	rs6589677	11	118713262	A	G	0.113753	0.156736	0.0259784	1.69E-09	7213
KIAA1279	rs17471869	10	68990113	A	G	0.480313	0.0938728	0.016487	1.29E-08	7213
TSG101	rs1395319	11	18526636	T	G	0.338278	0.527376	0.0164693	1.73E-210	7213
RFK	rs10869782	9	76485106	A	G	0.481561	0.105905	0.0165466	1.65E-10	7213
RFK	rs594586	9	76699375	G	A	0.234576	-0.165003	0.0193892	2.09E-17	7213
GMFG	rs251906	19	39336198	C	G	0.0862332	-0.396104	0.0291698	1.72E-41	7213
UNC45A	rs8041035	15	90953012	C	T	0.0286289	-0.384635	0.0500775	1.79E-14	7213
S100A6	rs472778	1	153538628	G	A	0.0551088	-0.238888	0.0360286	3.59E-11	7213

S100A6	rs60969679	1	153546477	G	A	0.0388881	0.518715	0.0422943	3.08E-34	7213
SECTM1	rs78330892	17	82092116	C	T	0.127686	-0.158272	0.0247421	1.69E-10	7213
SECTM1	rs1132115	17	82321536	T	A	0.486899	-0.350322	0.0161921	1.25E-100	7213
RSPO3	rs853974	6	126747838	T	C	0.270415	0.129691	0.0186678	4.05E-12	7213
RSPO3	rs2154167	6	127166661	T	C	0.462498	-0.444226	0.0156135	7.54E-169	7213
REG1A	rs11126696	2	79096762	A	G	0.384167	-0.294665	0.016885	7.85E-67	7213
REG1A	rs13422544	2	79133210	A	T	0.109455	-0.194896	0.0264194	1.80E-13	7213
REG1A	rs11685616	2	79187880	G	T	0.054277	0.251469	0.0367332	8.23E-12	7213
FAM3D	rs72877731	3	58647547	C	G	0.0221822	0.382069	0.0559015	8.89E-12	7213
FAM3D	rs7433100	3	58665459	T	C	0.0993345	-0.438052	0.0271122	1.04E-57	7213
LYPD3	rs11666797	19	43458246	C	A	0.151047	-0.245002	0.0227987	9.82E-27	7213
NEGR1	rs78738825	1	71587342	C	T	0.0435325	-0.242873	0.0406705	2.46E-09	7213
NEGR1	rs11209871	1	71934973	A	G	0.139817	-0.238782	0.0238095	1.62E-23	7213
FSTL1	rs916510	3	120425010	C	T	0.277901	0.168963	0.0185992	1.33E-19	7213
SPP1	rs1471400	4	87853095	G	A	0.358173	-0.102178	0.0173951	4.44E-09	7213
LUM	rs78550277	12	91028164	T	G	0.0728546	0.223069	0.0321294	4.18E-12	7213
LUM	rs3759223	12	91113006	A	G	0.0198946	-0.712224	0.0589871	3.02E-33	7213
LUM	rs77651188	12	91138494	C	T	0.0108138	0.478362	0.0807301	3.26E-09	7213
LUM	rs1803343	12	91146007	T	C	0.0221822	-0.60556	0.0558058	3.19E-27	7213
CD177	rs144764562	19	43035153	G	A	0.0153196	0.515794	0.0667907	1.29E-14	7213
CD177	rs138707456	19	43132267	A	G	0.110426	0.356833	0.0260726	4.12E-42	7213
CD177	rs74557322	19	43305369	T	C	0.0254402	-0.327718	0.0523816	4.17E-10	7213
CD177	rs79338083	19	43322051	C	T	0.0196174	1.54842	0.0575927	8.09E-152	7213
CD177	rs78530667	19	43396545	T	C	0.129904	-0.530804	0.0238591	4.17E-106	7213
CD177	rs141280798	19	43453735	G	A	0.0572577	-0.25795	0.0357286	5.74E-13	7213
SMOC1	rs8011342	14	69502464	A	G	0.0779149	-0.170058	0.030865	3.72E-08	7213
SMOC1	rs1958078	14	69888141	A	C	0.157077	-0.516105	0.0219425	6.29E-118	7213
SMOC1	rs8019103	14	70019087	T	C	0.369818	0.159445	0.0172102	2.54E-20	7213
SMOC1	rs61977430	14	70111634	C	T	0.152364	-0.155558	0.022862	1.10E-11	7213
SERPINA10	rs4900218	14	94119515	C	T	0.046028	0.221045	0.0395935	2.45E-08	7213
SERPINA10	rs6575399	14	94213439	G	A	0.168862	0.852455	0.0199171	0	7213
FLRT2	rs1667515	14	85227560	G	A	0.372106	0.120834	0.0171147	1.82E-12	7213
FLRT2	rs17796783	14	85343567	T	C	0.286219	-0.536857	0.0172367	6.31E-200	7213
FLRT2	rs17094470	14	85645416	G	C	0.166158	-0.309092	0.0222384	2.32E-43	7213
FLRT3	rs117932517	20	13980957	T	C	0.0375711	-0.25424	0.0437717	6.58E-09	7213
FLRT3	rs2423816	20	14554153	A	G	0.297726	0.722384	0.0161104	0	7213
FLRT3	rs6033994	20	14640308	G	A	0.149868	-0.382925	0.0229191	1.64E-61	7213
FLRT3	rs11087104	20	14714733	C	G	0.246569	-0.202348	0.0192492	1.17E-25	7213
ISLR2	rs4886578	15	74180694	C	T	0.0564952	-0.233593	0.0362355	1.22E-10	7213
ISLR2	rs4887134	15	74317938	A	T	0.403438	0.289794	0.0164583	5.60E-68	7213
DSC2	rs1789064	18	31094439	A	T	0.238528	0.371646	0.0190072	5.35E-83	7213
DSC2	rs77239293	18	31151789	G	T	0.0219742	0.320488	0.0573053	2.32E-08	7213
DSC2	rs55663303	18	31157234	A	G	0.0293221	-0.300135	0.0488792	8.67E-10	7213

HK2	rs641180	2	74812957	A	G	0.281575	0.147991	0.0184499	1.21E-15	7213
HK2	rs3771788	2	74849662	A	C	0.415569	0.0950248	0.0167276	1.39E-08	7213
SEMA5A	rs13174956	5	9436585	T	C	0.35845	-0.249122	0.0170913	1.88E-47	7213
SEMA5A	rs17329324	5	9547846	C	G	0.0895605	0.939395	0.0267277	6.26E-250	7213
SEMA5A	rs218052	5	9580161	A	C	0.0433939	-0.259153	0.0407813	2.22E-10	7213
LTBP4	rs112009052	19	40593595	T	A	0.0166366	-0.568793	0.0639164	7.03E-19	7213
SHANK3	rs6009946	22	50656858	C	T	0.258214	0.401098	0.0181928	2.68E-104	7213
B4GALT1	rs7019909	9	33113324	C	T	0.100444	0.630544	0.0264335	4.11E-121	7213
FH	rs10926507	1	241531114	A	G	0.289408	-0.188048	0.0184963	4.05E-24	7213
PCSK1	rs4078005	5	96083565	A	G	0.0365313	0.255527	0.0442342	7.94E-09	7213
PCSK1	rs11744722	5	96205127	T	A	0.0187855	0.471654	0.0611855	1.44E-14	7213
PCSK1	rs6235	5	96393194	C	G	0.27395	-0.999388	0.0144638	0	7213
SPINK2	rs7694521	4	56755581	G	A	0.469084	0.216886	0.0164046	1.90E-39	7213
SPINK2	rs9992616	4	56781880	C	A	0.402606	-0.108981	0.0170896	1.92E-10	7213
TMEM132D	rs7299188	12	129289849	C	G	0.370512	0.170914	0.0171449	2.95E-23	7213
TMEM132D	rs139076549	12	129568952	A	G	0.0189242	0.504236	0.0609351	1.52E-16	7213
FAM213A	rs10788623	10	80439659	G	A	0.44808	-0.093119	0.0167792	2.96E-08	7213
MAN1C1	rs11247595	1	25682168	T	C	0.491543	0.292094	0.0161227	8.98E-72	7213
USP8	rs62016940	15	50425726	A	G	0.169694	0.246684	0.0219595	4.86E-29	7213
CHAD	rs71379398	17	50319091	T	C	0.0843616	-0.168555	0.0298932	1.78E-08	7213
PXDN	rs1054241	2	1633876	T	C	0.403022	0.11593	0.0169181	7.86E-12	7213
PXDN	rs10202812	2	1742696	A	T	0.240399	0.249433	0.0192405	5.20E-38	7213
HDHD2	rs117792675	18	47044338	G	A	0.0259254	-0.863907	0.0513612	2.64E-62	7213
HDHD2	rs114475395	18	47164639	A	G	0.0566339	1.31254	0.0322773	0	7213
ARFIP1	rs4619875	4	152779978	C	T	0.39304	0.425011	0.0161998	5.61E-145	7213
ARHGEF25	rs10437954	12	57610139	G	A	0.102731	0.310478	0.0275137	2.75E-29	7213
MYOM2	rs3824184	8	2098172	G	A	0.144045	-0.172151	0.0236548	3.76E-13	7213
HMHA1	rs2240051	19	1076684	A	G	0.379662	0.351603	0.0168121	2.43E-94	7213
EIF1AD	rs182527881	11	65955537	C	T	0.0255095	0.622983	0.0528404	8.61E-32	7213
SWAP70	rs11042412	11	9533946	G	C	0.18834	0.165753	0.0208952	2.47E-15	7213
SWAP70	rs12804730	11	9613169	G	T	0.0340358	-0.265529	0.0456422	6.22E-09	7213
SWAP70	rs415895	11	9748015	C	G	0.344517	0.627812	0.0158332	3.1597999999998e-311	7213
SWAP70	rs140020287	11	9935957	G	C	0.0192708	-0.352253	0.0603354	5.50E-09	7213
ABLIM3	rs114464628	5	149154161	G	A	0.0341051	-0.311998	0.0459482	1.21E-11	7213
REXO2	rs4938104	11	114427673	C	G	0.149799	-0.285735	0.0232605	2.43E-34	7213
REXO2	rs7122337	11	114526777	T	C	0.271385	-0.105287	0.01866	1.74E-08	7213
GGA1	rs12483880	22	37655992	C	T	0.421461	0.0931995	0.0167132	2.54E-08	7213
NCK2	rs10192144	2	105823398	A	G	0.377859	-0.177641	0.0169584	1.71E-25	7213
NCK2	rs3769497	2	105873565	C	A	0.0622487	-0.241425	0.0344569	2.66E-12	7213
AP2A2	rs4963151	11	920911	T	C	0.433384	-0.168879	0.0166356	4.72E-24	7213
PPP2R5A	rs11119862	1	212167815	A	G	0.164079	0.155083	0.0226827	8.74E-12	7213
NADK	rs4648629	1	1764023	C	A	0.440663	-0.311603	0.0164263	2.40E-78	7213
ZYX	rs7801889	7	143499080	T	C	0.381741	0.107615	0.0169482	2.29E-10	7213

PNKP	rs2290774	19	49871778	T	C	0.426175	-0.101033	0.0169952	2.90E-09	7213
CA10	rs2106331	17	51660546	G	A	0.348607	-0.0984152	0.0173423	1.44E-08	7213
CA10	rs1354276	17	52100122	A	T	0.380147	0.17408	0.0171605	5.09E-24	7213
CA10	rs117399000	17	52136371	G	A	0.0294607	-0.734537	0.0480137	5.10E-52	7213
FGFR3	rs79825628	4	1684828	G	A	0.183488	0.366185	0.0210873	3.32E-66	7213
FGFR3	rs3135878	4	1802650	G	A	0.202482	0.211748	0.0206512	1.68E-24	7213
ELANE	rs58350690	19	794670	T	C	0.153473	-0.149593	0.0229148	7.11E-11	7213
ELANE	rs56283881	19	893183	C	T	0.247331	-0.238824	0.0187831	1.21E-36	7213
INHBB	rs17050272	2	120548864	G	A	0.425828	0.421393	0.0160361	2.04E-145	7213
CSF1R	rs35341726	5	150024616	G	T	0.0684875	0.220091	0.032705	1.83E-11	7213
CSF1R	rs115138219	5	150112084	A	G	0.0223901	-0.625059	0.0558902	8.45E-29	7213
IL5RA	rs340827	3	3066046	G	A	0.4033	0.178751	0.0166923	1.47E-26	7213
IL5RA	rs77400868	3	3109280	A	G	0.138361	0.500071	0.0232641	2.15E-99	7213
WISP1	rs2739148	8	133071105	T	C	0.211978	0.229893	0.0200852	4.48E-30	7213
WISP1	rs60282000	8	133192798	T	G	0.354291	-0.292064	0.0172647	5.48E-63	7213
ANXA2	rs10851680	15	60386905	T	G	0.172466	-0.211118	0.0218773	6.66E-22	7213
ANXA2	rs8033800	15	60396980	T	A	0.386178	0.397233	0.0165742	3.35E-122	7213
FCN2	rs11103544	9	134843197	T	C	0.14155	-0.331951	0.0234272	5.66E-45	7213
FCN2	rs11103563	9	134886852	A	G	0.112159	-0.999999	0.0236189	0	7213
PAK4	rs11667387	19	39184097	C	T	0.350132	-0.11375	0.0174807	8.17E-11	7213
PRTN3	rs2930903	19	887824	G	C	0.40219	-0.146841	0.0170108	7.32E-18	7213
IL12B	rs17052525	5	159164158	G	A	0.107791	-0.193616	0.0267796	5.33E-13	7213
IL12B	rs2901313	5	159347171	T	C	0.309996	-0.58191	0.0164568	1.03E-252	7213
INHBA	rs7808613	7	41707324	C	G	0.244628	-0.105985	0.0193724	4.63E-08	7213
FRZB	rs12991615	2	182623586	C	A	0.11611	0.150523	0.0260335	7.69E-09	7213
FRZB	rs288326	2	182838608	G	A	0.116595	0.637811	0.0249547	5.29E-138	7213
FRZB	rs72890325	2	183063340	T	G	0.0932344	-0.5444	0.0279908	3.66E-82	7213
CCL8	rs1821140	17	34326665	C	T	0.180438	-1.02947	0.0182287	0	7213
CCL8	rs159247	17	34412574	A	G	0.26168	-0.108548	0.0187391	7.22E-09	7213
CROT	rs31659	7	87411313	C	T	0.10488	-0.60755	0.026257	2.55E-114	7213
APOBEC3G	rs5757442	22	39040041	T	A	0.480937	0.116444	0.0165775	2.35E-12	7213
APOBEC3G	rs3891126	22	39088173	C	G	0.10086	-0.332835	0.0273062	7.65E-34	7213
PSMD9	rs10743185	12	121918529	G	A	0.431582	-0.205487	0.0166913	1.75E-34	7213
IFI16	rs856051	1	159031502	T	C	0.180577	-0.242172	0.0214018	1.95E-29	7213
DPY30	rs140841871	2	32226651	C	T	0.0451269	0.453859	0.0399842	1.31E-29	7213
SULT1A3	rs148788997	16	30311847	G	C	0.118883	0.185788	0.02578	6.31E-13	7213
NUDT12	rs11242495	5	103535378	A	G	0.15576	-0.32002	0.02268	1.28E-44	7213
COL4A3BP	rs5744681	5	75584525	G	A	0.0859559	0.259632	0.0291844	7.20E-19	7213
GNPNAT1	rs72684248	14	52538492	A	C	0.0463746	0.234726	0.0395802	3.16E-09	7213
GNPNAT1	rs2296494	14	52783575	G	C	0.382504	-0.348992	0.0167281	6.67E-94	7213
LAP3	rs114838597	4	17578371	C	T	0.0259947	0.452524	0.052167	5.07E-18	7213
TOLLIP	rs145066684	11	1251598	T	C	0.0294607	-0.272049	0.0486814	2.38E-08	7213
TOLLIP	rs2056083	11	1348325	G	A	0.149452	-0.151326	0.0233187	9.18E-11	7213

TXNRD1	rs7298739	12	104296440	T	C	0.447941	0.139226	0.0166977	8.97E-17	7213
KPNA6	rs72666741	1	32102997	G	A	0.0282129	0.858735	0.0492103	7.97E-67	7213
HSD17B14	rs12978094	19	48809982	A	C	0.0394427	-0.273356	0.0425046	1.35E-10	7213
PPFIA1	rs12577692	11	70503671	A	G	0.325731	-0.133938	0.0178127	6.18E-14	7213
CRYZ	rs11210463	1	74456718	G	C	0.196312	0.122754	0.021179	7.08E-09	7213
CRYZ	rs12061533	1	74692434	A	G	0.157632	0.210443	0.0228933	4.94E-20	7213
CRYZ	rs11485299	1	74753462	A	G	0.122487	1.03177	0.0220581	0	7213
CRYZ	rs2065728	1	74949323	C	T	0.395051	-0.186945	0.0168333	2.00E-28	7213
LANCL1	rs2287418	2	210476432	C	G	0.496742	-0.136082	0.0166449	3.45E-16	7213
NMRAL1	rs11557236	16	4469438	G	A	0.0849854	-0.510755	0.0292133	4.55E-67	7213
ADSSL1	rs10142660	14	104759283	G	T	0.126022	0.185815	0.0251712	1.73E-13	7213
GNMT	rs2395943	6	42972935	A	G	0.420006	0.428481	0.0162219	7.70E-147	7213
GNMT	rs9472044	6	43420486	T	C	0.0259254	-0.323377	0.0528059	9.61E-10	7213
TNFAIP3	rs2230926	6	137874929	T	G	0.0305005	0.280835	0.0478128	4.45E-09	7213
BDNF	rs16917304	11	27764235	T	C	0.0392347	0.375336	0.0427327	1.96E-18	7213
IL1RAP	rs139644748	3	190576842	G	A	0.024747	-0.302963	0.052769	9.78E-09	7213
IL1RAP	rs4686558	3	190632099	A	G	0.154305	1.27403	0.01768	0	7213
IL1RAP	rs79383051	3	190649137	C	T	0.0408984	-0.316137	0.0419432	5.39E-14	7213
IL1RAP	rs79593794	3	190802174	C	A	0.0361153	-0.375499	0.0445208	3.99E-17	7213
IL15RA	rs41294151	10	5957517	G	T	0.0854707	-0.328029	0.0297284	4.38E-28	7213
IL15RA	rs8177655	10	5973156	G	A	0.317898	0.649259	0.0159459	0	7213
IL15RA	rs12722557	10	6028445	C	T	0.0325801	0.286888	0.0466264	8.01E-10	7213
IL15RA	rs58820784	10	6128373	C	T	0.142035	-0.179992	0.0235558	2.43E-14	7213
PKP2	rs11052286	12	32886492	T	C	0.290725	0.114803	0.0182234	3.16E-10	7213
CST4	rs7263473	20	23709830	G	A	0.103078	-0.458975	0.026956	9.06E-64	7213
IL18R1	rs74664310	2	101853351	G	A	0.0250936	0.343501	0.0533	1.23E-10	7213
IL18R1	rs13012334	2	102119560	A	G	0.127825	-0.189433	0.0248745	2.96E-14	7213
IL18R1	rs2160232	2	102430420	G	A	0.245321	0.851481	0.016545	0	7213
IGFBP6	rs822688	12	53099603	C	T	0.142728	-0.221984	0.02372	1.06E-20	7213
DEF6	rs1041528	6	35222343	T	A	0.149383	-0.133666	0.023209	8.80E-09	7213
DEF6	rs45482297	6	35297758	G	A	0.0336892	-0.547559	0.0457004	9.08E-33	7213
CBR3	rs62229277	21	36118748	C	T	0.338555	-0.80311	0.0148492	0	7213
CBR3	rs73202262	21	36249629	A	G	0.036046	0.370777	0.0442081	5.94E-17	7213
HBEGF	rs1991801	5	140623810	A	G	0.293221	0.109534	0.0180057	1.24E-09	7213
CARS	rs445375	11	3034705	T	A	0.406211	0.149995	0.0170772	1.96E-18	7213
C1QC	rs75643365	1	22441151	C	T	0.0171219	0.368019	0.06405	9.52E-09	7213
C1QC	rs11810828	1	22615195	C	T	0.0340358	-0.301355	0.0458047	5.06E-11	7213
C1QC	rs141997172	1	22634878	A	G	0.0119229	0.807774	0.0752161	1.06E-26	7213
C1QC	rs75380810	1	22663039	T	C	0.0395813	0.962427	0.0415124	9.54E-115	7213
C1QC	rs75837320	1	22678038	A	G	0.0119229	1.01284	0.0748712	3.40E-41	7213
CNTFR	rs115034926	9	34551922	G	A	0.0194787	0.385108	0.0604448	1.99E-10	7213
CNTFR	rs73645429	9	34594286	G	A	0.0181617	-0.996468	0.0605838	1.05E-59	7213
CNTFR	rs12352811	9	34614684	G	C	0.0605851	-0.372232	0.0344651	5.52E-27	7213

MTHFS	rs282778	15	79758743	C	T	0.235478	0.327801	0.0192964	1.72E-63	7213
MTHFS	rs34397803	15	79922335	T	G	0.357688	-0.698557	0.015117	0	7213
MTHFS	rs28561542	15	80155569	C	T	0.439623	-0.121744	0.0166859	3.28E-13	7213
THBS2	rs73041853	6	169203903	G	A	0.0978788	0.214101	0.0278352	1.64E-14	7213
RELT	rs56801796	11	73379220	A	G	0.216276	0.154046	0.0198475	9.55E-15	7213
PIANP	rs11064321	12	6700730	G	C	0.376958	0.289271	0.0167018	7.08E-66	7213
S100A4	rs79399329	1	153544257	G	A	0.0564259	0.245223	0.0358169	8.19E-12	7213
TNFRSF10D	rs4460371	8	23134288	T	C	0.489325	0.10224	0.0165486	6.84E-10	7213
C10orf54	rs10999983	10	71754145	C	G	0.314363	-0.165283	0.0178283	2.40E-20	7213
C10orf54	rs12415873	10	71770478	C	T	0.143283	0.610659	0.0227584	2.99E-151	7213
EFNB2	rs59166663	13	106647317	A	C	0.142035	0.278786	0.0235026	3.71E-32	7213
EFNB2	rs3803241	13	106916932	G	T	0.316512	0.175732	0.0178381	9.40E-23	7213
IL1R2	rs2310170	2	101999692	T	G	0.401497	-0.521562	0.0160216	5.20E-217	7213
AMIGO2	rs1101751	12	47092599	G	A	0.300014	0.149186	0.0180804	1.85E-16	7213
ISG15	rs3128111	1	995371	C	G	0.382227	0.307143	0.0167996	4.97E-73	7213
EFNA3	rs17723260	1	155086187	G	A	0.142243	-0.138962	0.0236523	4.41E-09	7213
YWHAB	rs2425681	20	44918221	A	T	0.36656	-0.663194	0.0153346	0	7213
YWHAB	rs2868217	20	44963260	G	A	0.370096	0.152437	0.017172	8.55E-19	7213
ANXA7	rs117332875	10	73370976	C	G	0.0203106	-0.46588	0.0587495	2.52E-15	7213
RBP7	rs35232720	1	9991107	C	T	0.054485	-1.01757	0.0345785	7.42E-180	7213
RBP7	rs143320801	1	10019021	C	T	0.0166366	-0.375433	0.0646532	6.64E-09	7213
DOK2	rs2054713	8	21983023	A	G	0.432136	0.111271	0.0167426	3.23E-11	7213
RAB6B	rs9813363	3	133885899	G	A	0.15583	-0.68858	0.0212426	2.64E-215	7213
PREP	rs1051484	6	105278161	C	T	0.156662	0.793538	0.0210403	2.87E-284	7213
PREP	rs11755530	6	105339937	A	G	0.202898	-0.453896	0.0199683	1.58E-110	7213
VOPP1	rs117042408	7	55555698	G	A	0.0305698	-0.489085	0.0475311	1.16E-24	7213
SUMO3	rs34329954	21	44747002	G	A	0.220505	0.114689	0.0199734	9.73E-09	7213
SUMO3	rs235343	21	44829837	A	C	0.418966	0.105019	0.0167782	4.09E-10	7213
SUMO3	rs8133302	21	44835300	A	C	0.227298	0.216968	0.019798	9.93E-28	7213
HRSP12	rs10955148	8	98107592	T	C	0.316512	-0.410085	0.0172782	5.78E-120	7213
DNAJC17	rs57825040	15	40800118	C	G	0.365659	-0.115275	0.0173211	3.04E-11	7213
EIF4B	rs146008363	12	53037514	T	A	0.0571191	-0.212298	0.0358059	3.19E-09	7213
CAPN2	rs188530181	1	223691121	A	C	0.0108831	-1.43837	0.0788757	1.13E-72	7213
CAPN2	rs10915880	1	223853332	G	A	0.150146	0.164604	0.0230659	1.05E-12	7213
C8G	rs7862602	9	136946019	G	T	0.465132	0.497576	0.0154524	1.13E-212	7213
CST6	rs3825068	11	66000622	A	G	0.0256481	0.616729	0.0527134	2.45E-31	7213
CRLF1	rs141412226	19	18606827	G	A	0.204423	0.337598	0.0201955	1.42E-61	7213
MESDC2	rs1012540	15	80953751	A	T	0.0483849	-0.439129	0.0386173	1.04E-29	7213
MESDC2	rs2663943	15	81096638	G	C	0.346319	-0.0999867	0.0174504	1.05E-08	7213
CCDC134	rs1807590	22	41313231	G	A	0.41647	-0.092472	0.0168216	3.99E-08	7213
CCDC134	rs11090057	22	41735443	G	A	0.357688	0.361625	0.0168082	1.45E-99	7213
CCDC134	rs11704061	22	41832344	G	C	0.0175378	0.396112	0.0620533	1.84E-10	7213
REG3A	rs72913277	2	79140251	A	G	0.039512	-0.336005	0.0428031	4.76E-15	7213

REG3A	rs116652994	2	79163078	A	G	0.0587828	0.465378	0.0350482	9.05E-40	7213
VWC2	rs76473891	7	49504033	G	C	0.0246083	0.33122	0.0536436	7.00E-10	7213
VWC2	rs75731564	7	49644661	C	A	0.02648	0.328445	0.0512932	1.62E-10	7213
VWC2	rs148681119	7	49718162	C	A	0.0222515	0.837146	0.0556706	2.40E-50	7213
VWC2	rs77510717	7	49731032	G	A	0.0230833	-0.598638	0.0546142	9.71E-28	7213
VWC2	rs1876569	7	49782154	A	T	0.142798	-0.639182	0.0221821	6.48E-173	7213
VWC2	rs76707602	7	50220893	C	A	0.0336892	0.272402	0.0458455	2.95E-09	7213
CLIC4	rs113965554	1	24394806	G	A	0.0336892	0.398846	0.0458149	3.87E-18	7213
CLIC4	rs4649017	1	24746529	T	C	0.373492	-0.258788	0.0167082	2.96E-53	7213
TXNL4B	rs116891509	16	72071661	C	T	0.0734091	0.557124	0.0311312	4.09E-70	7213
CRADD	rs60169972	12	93837506	G	C	0.186469	-0.149609	0.0212386	2.04E-12	7213
GBP1	rs71666209	1	88989898	G	A	0.0388881	-0.295397	0.0428283	5.75E-12	7213
GBP1	rs61798920	1	89051436	C	T	0.35034	-0.168436	0.0173204	3.24E-22	7213
SELM	rs117551362	22	31104089	G	A	0.0490087	0.285625	0.0385448	1.40E-13	7213
KNG1	rs5029993	3	186721506	G	A	0.114446	0.145151	0.0261537	2.96E-08	7213
KNG1	rs5030062	3	186736391	A	C	0.37682	0.313407	0.0166336	2.42E-77	7213
APOA5	rs12286037	11	116781491	C	T	0.0687647	1.25848	0.0294108	0	7213
APOA5	rs10750096	11	116786072	C	A	0.06925	-0.37948	0.0323254	1.55E-31	7213
APOC1	rs12721046	19	44917997	G	A	0.155691	0.143516	0.0228703	3.69E-10	7213
APOC1	rs5112	19	44927023	C	G	0.466449	-0.307528	0.0162896	1.24E-77	7213
BPIFB1	rs2424963	20	33106642	T	C	0.413282	0.320032	0.0165234	1.64E-81	7213
BMPER	rs16879245	7	33764873	A	G	0.082074	-0.381904	0.0296743	1.72E-37	7213
BMPER	rs34372750	7	34062779	T	C	0.262443	-0.143123	0.0188842	3.92E-14	7213
BMPER	rs2058681	7	34154490	A	G	0.316512	-0.1525	0.0177523	1.05E-17	7213
BOLA1	rs1044808	1	149900352	G	C	0.0777762	1.08971	0.0285285	6.76E-291	7213
CPB1	rs388488	3	148645190	C	T	0.205878	-0.113932	0.0205142	2.89E-08	7213
CPB1	rs56745771	3	148827514	C	T	0.370581	-0.115322	0.0172467	2.45E-11	7213
CPB1	rs13318851	3	148844610	G	C	0.227506	0.409447	0.019168	3.24E-98	7213
KL	rs7321110	13	32956017	T	C	0.046028	-0.316373	0.0395037	1.34E-15	7213
KL	rs9527032	13	33056892	G	C	0.151601	0.213542	0.0227746	8.97E-21	7213
FABP2	rs4549362	4	119129891	C	A	0.0784694	-0.22929	0.0306423	8.14E-14	7213
FABP2	rs7656644	4	119306880	G	A	0.261195	-0.920018	0.0157312	0	7213
FABP4	rs1486006	8	81478382	G	C	0.183072	-0.172477	0.0217118	2.26E-15	7213
NRP2	rs62172729	2	205182223	C	T	0.19042	-0.252313	0.0210351	7.75E-33	7213
NRP2	rs3771004	2	205766163	C	T	0.285942	0.162317	0.0181104	3.98E-19	7213
FCGR3A	rs116601080	1	161339137	C	T	0.0505338	-0.26527	0.0379514	3.00E-12	7213
FCGR3A	rs6700241	1	161531185	A	G	0.201026	0.821891	0.0184636	0	7213
FCGR3A	rs2185550	1	161692211	T	C	0.410925	0.215283	0.0167226	1.63E-37	7213
GAS6	rs6602909	13	113849020	T	C	0.322335	-0.405253	0.0171732	1.16E-118	7213
UNC5B	rs145365000	10	71047349	G	C	0.0154582	0.441676	0.0678106	7.84E-11	7213
UNC5B	rs7907590	10	71292875	G	A	0.141758	0.262621	0.0234328	6.50E-29	7213
UNC5B	rs13419	10	71299632	A	G	0.282615	0.137846	0.018499	1.03E-13	7213
GSTM1	rs116362610	1	109397043	C	G	0.0185082	0.411154	0.0612113	2.00E-11	7213

GSTM1	rs115181845	1	109510351	C	T	0.0721614	0.463196	0.0315545	4.31E-48	7213
GSTM1	rs2269340	1	109629614	T	C	0.0752807	0.627972	0.0306016	5.36E-91	7213
GSTM1	rs115929572	1	109703431	G	A	0.0751421	0.767389	0.0302131	2.21E-136	7213
LDHA	rs7112492	11	18383963	G	T	0.174962	0.190473	0.0216972	2.04E-18	7213
SERPINB5	rs7244879	18	63484141	A	G	0.319077	-0.138506	0.017872	1.05E-14	7213
LOXL3	rs17010021	2	74534412	T	A	0.0367392	-0.290539	0.0442673	5.63E-11	7213
PNP	rs1049564	14	20472447	G	A	0.176279	-0.659672	0.0202087	4.10E-218	7213
PNP	rs1760930	14	20486851	A	G	0.189658	-0.244497	0.0208882	2.31E-31	7213
PCSK2	rs6044716	20	17269967	A	T	0.494246	-0.131171	0.0165402	2.51E-15	7213
GM2A	rs72794132	5	151239432	C	T	0.165812	0.706237	0.0208557	2.10E-233	7213
GM2A	rs375396	5	151287372	C	A	0.184181	0.429414	0.0206856	5.11E-93	7213
GM2A	rs10071361	5	151304950	A	T	0.0354915	-0.255705	0.0448059	1.20E-08	7213
SORD	rs56060952	15	45036783	T	C	0.11916	0.467346	0.0250176	4.35E-76	7213
SORD	rs28662287	15	45068813	C	T	0.125191	-0.25599	0.025053	2.41E-24	7213
TIMD4	rs58198139	5	156972028	C	T	0.364827	0.2057	0.0171373	7.03E-33	7213
NT5E	rs2593335	6	85378941	C	T	0.499515	0.231981	0.0164681	1.78E-44	7213
NT5E	rs6903114	6	85696953	T	C	0.311174	0.464768	0.0171471	3.73E-154	7213
AMBP	rs10982050	9	114066493	A	G	0.242132	-0.150718	0.0192038	4.82E-15	7213
ANPEP	rs1042499	15	89785074	T	C	0.0594759	0.21923	0.0352545	5.30E-10	7213
DPP4	rs13015258	2	162074215	T	G	0.394704	0.124202	0.0168236	1.73E-13	7213
CD8A	rs3020726	2	86789383	A	G	0.165604	0.510069	0.0217112	1.15E-117	7213
COL9A1	rs13199337	6	70279372	C	T	0.175378	0.143297	0.0218324	5.62E-11	7213
CFHR1	rs67908756	1	196852250	T	G	0.205254	-0.939397	0.0169717	0	7213
CFHR1	rs61820789	1	197022933	G	C	0.425551	0.118008	0.0168808	2.98E-12	7213
HEXB	rs13164140	5	74734513	G	A	0.247193	0.46273	0.0185465	7.39E-132	7213
HEXB	rs113358700	5	74923508	G	C	0.147511	-0.13651	0.0236412	8.05E-09	7213
PNLIPRP2	rs2301179	10	116645109	A	G	0.494801	-0.819875	0.0133175	0	7213
PNLIPRP2	rs3010459	10	116671159	C	T	0.47962	-0.136543	0.0164656	1.31E-16	7213
PNLIPRP2	rs11596597	10	116855346	C	A	0.137599	-0.163637	0.0241378	1.30E-11	7213
LRP11	rs73604783	6	149566749	T	C	0.0155968	0.909092	0.0662442	2.50E-42	7213
LRP11	rs35830138	6	149668635	T	C	0.365105	0.864711	0.0140913	0	7213
LRP11	rs75672805	6	149804154	A	G	0.0203106	-0.440953	0.0585709	5.75E-14	7213
PLTP	rs76070947	20	45484646	C	G	0.0456814	-0.243084	0.039641	9.13E-10	7213
PLTP	rs111602331	20	45928835	T	C	0.184597	-0.738606	0.0196868	1.43E-281	7213
PLTP	rs73312715	20	46157375	C	T	0.0609317	0.192722	0.0345892	2.61E-08	7213
REG3G	rs430298	2	79022646	G	T	0.218148	0.584061	0.0188785	1.90E-197	7213
REG3G	rs140715469	2	79078756	T	C	0.0449189	0.342595	0.039762	8.41E-18	7213
VNN2	rs56739181	6	132755350	C	A	0.342715	-0.737598	0.0151326	0	7213
VNN2	rs73773521	6	133081796	C	G	0.0120616	-0.473388	0.0760723	5.15E-10	7213
AGRN	rs4970350	1	1036800	G	A	0.427423	-0.294348	0.0166672	2.30E-68	7213
AOC1	rs73163584	7	150772831	C	G	0.0194094	0.357195	0.0599093	2.60E-09	7213
AOC1	rs6977381	7	150845558	G	A	0.444406	0.671188	0.0148534	0	7213
AOC1	rs3778871	7	150973684	A	C	0.106474	0.177913	0.0269209	4.15E-11	7213

CES1	rs76875916	16	55818117	T	C	0.0469985	-0.460061	0.0391383	1.30E-31	7213
CES1	rs74019283	16	55836327	G	T	0.0378483	-0.247582	0.0434671	1.28E-08	7213
CD248	rs551243	11	66307012	G	C	0.479343	0.0955545	0.0167382	1.18E-08	7213
FGFBP1	rs73230203	4	15962080	G	A	0.0925412	0.192551	0.0286293	1.88E-11	7213
FOLR3	rs637195	11	71889354	G	T	0.277416	-0.11592	0.0183394	2.76E-10	7213
FOLR3	rs146694036	11	72145571	C	T	0.0938583	1.02158	0.0259233	8.72E-308	7213
FOLR3	rs79341752	11	72287283	A	G	0.0524054	0.325506	0.0369386	1.52E-18	7213
ATRN	rs138318457	20	3292219	C	T	0.0386108	-0.55941	0.042679	8.28E-39	7213
ATRN	rs151544	20	3503901	G	C	0.0641203	-0.656063	0.0332792	2.64E-84	7213
ATRN	rs144469244	20	3816499	C	G	0.0119922	-0.843718	0.0758405	1.62E-28	7213
LEFTY2	rs360078	1	225896277	G	A	0.354707	-0.331182	0.0169318	4.71E-83	7213
LEFTY2	rs10799316	1	225480329	G	A	0.245876	0.114486	0.0194382	4.04E-09	7213
LEFTY2	rs360088	1	225869991	G	A	0.363718	0.72069	0.015092	0	7213
LEFTY2	rs4653441	1	226107091	G	A	0.116456	-0.218726	0.0257313	2.27E-17	7213
NAGLU	rs1123363	17	42080196	G	A	0.0337585	0.286662	0.0455028	3.15E-10	7213
NAGLU	rs72823057	17	42415242	G	C	0.0674477	-0.224002	0.0331152	1.44E-11	7213
NAGLU	rs41283429	17	42480711	C	T	0.0419382	1.0998	0.039432	1.15E-162	7213
NPTXR	rs4821839	22	38831788	G	A	0.0186469	1.62178	0.0588682	6.09E-159	7213
NPTXR	rs735306	22	38864027	C	T	0.385554	-0.14004	0.0169834	1.93E-16	7213
PRSS8	rs1060506	16	31122128	C	T	0.294191	0.124778	0.0182774	9.38E-12	7213
BCHE	rs3863090	3	165666699	G	T	0.126993	-0.144174	0.024947	7.82E-09	7213
BCHE	rs62295996	3	165764276	G	A	0.203106	-0.438648	0.0198952	2.63E-104	7213
BCHE	rs148077724	3	165952844	A	G	0.0422848	-0.29932	0.0409249	2.87E-13	7213
SAA1	rs11024589	11	18256876	A	C	0.104741	-0.798865	0.0253572	3.19E-204	7213
SAA1	rs76204461	11	18493515	C	T	0.0752114	0.232937	0.0313111	1.13E-13	7213
SAA4	rs151172395	11	18195559	G	A	0.0176071	1.481	0.0611696	1.43E-124	7213
SAA4	rs4757629	11	18224223	C	G	0.470331	0.590198	0.0150417	2.19E-305	7213
SAA4	rs10430893	11	18259448	C	T	0.104395	-0.549938	0.02633	4.17E-94	7213
CLSTN1	rs35331030	1	9751595	C	T	0.0167059	1.78296	0.060609	9.77E-180	7213
CLSTN1	rs114513597	1	9769606	G	A	0.0158048	-0.692718	0.0664827	3.05E-25	7213
GLIPR2	rs72729437	9	35963179	A	G	0.0370858	0.262566	0.0439333	2.39E-09	7213
GLIPR2	rs10814339	9	36154681	A	C	0.269444	-0.353374	0.0182988	4.68E-81	7213
ALAD	rs11794302	9	113131922	T	C	0.0288368	-0.293745	0.04962	3.37E-09	7213
ALAD	rs7025333	9	113353500	C	T	0.419728	0.174843	0.0168566	4.96E-25	7213
ALAD	rs1800435	9	113391611	C	G	0.0797865	-0.67226	0.0297402	2.31E-109	7213
PGAM2	rs6956492	7	44064620	A	G	0.234992	-0.129707	0.0195828	3.76E-11	7213
ADH1C	rs2602885	4	99119979	C	G	0.185221	-0.143485	0.0211413	1.24E-11	7213
ADH1C	rs283415	4	99349450	C	T	0.434909	-0.542217	0.0155412	1.42E-246	7213
GSS	rs6088642	20	34895383	G	T	0.375711	-0.438391	0.0162007	1.13E-153	7213
DLD	rs3735602	7	107923141	A	G	0.420907	-0.0968738	0.0169326	1.10E-08	7213
CSRP1	rs3767541	1	201486782	C	T	0.346527	-0.180995	0.0174334	4.49E-25	7213
EPHB4	rs314345	7	100799705	G	C	0.472064	-0.258365	0.0165199	3.02E-54	7213
MSR1	rs41341748	8	16155085	G	A	0.0102593	-1.49438	0.0783621	3.87E-79	7213

MSR1	rs7831768	8	16169622	C	T	0.0749341	-0.506351	0.0309684	4.93E-59	7213
MDH2	rs10256	7	76066295	A	G	0.0491474	0.345975	0.0380857	1.33E-19	7213
PRSS27	rs71386687	16	2717893	G	T	0.077083	0.192378	0.0312443	7.80E-10	7213
SLITRK1	rs2876803	13	83794650	T	A	0.168723	0.158333	0.0222406	1.19E-12	7213
VIM	rs359276	10	17271304	G	A	0.293775	-0.111299	0.0181517	9.16E-10	7213
CKMT1A	rs2614819	15	43604248	C	A	0.0986413	-0.260674	0.0274066	2.51E-21	7213
KLK14	rs2569441	19	51011366	C	T	0.394427	-0.137146	0.0170209	9.04E-16	7213
KLK14	rs2691224	19	51041887	G	A	0.495702	-0.103894	0.0165272	3.44E-10	7213
KLK14	rs17658926	19	51081153	G	A	0.192916	0.279168	0.020928	4.07E-40	7213
KLK14	rs11666870	19	51095412	A	G	0.477956	-0.244253	0.0161602	7.77E-51	7213
AMY2B	rs12076610	1	103521319	G	T	0.093789	0.612807	0.0276844	4.28E-105	7213
AMY2B	rs114500706	1	103849546	A	G	0.0469985	-0.532566	0.0391361	1.17E-41	7213
AMY2B	rs77395797	1	103869827	C	T	0.011715	-0.618082	0.0770128	1.17E-15	7213
AMY2B	rs4847148	1	103907224	C	A	0.0154582	-0.413726	0.0672126	7.89E-10	7213
ENPEP	rs13132825	4	110223610	G	T	0.0535145	-0.349773	0.0369437	3.79E-21	7213
ENPEP	rs28421498	4	110479469	T	C	0.0924719	-0.19579	0.0287054	9.80E-12	7213
ENPEP	rs11735606	4	110517862	C	G	0.322751	0.382592	0.0172796	3.80E-105	7213
ANTXR2	rs17004062	4	79685611	G	A	0.16498	-0.187876	0.0222085	3.21E-17	7213
ANTXR2	rs10009674	4	79880234	T	A	0.249896	0.379264	0.0184663	3.83E-91	7213
TCN2	rs55679829	22	30604644	A	G	0.0240538	-0.451958	0.054095	7.78E-17	7213
TCN2	rs5753259	22	30634541	T	C	0.407736	0.820184	0.0140535	0	7213
TCN2	rs111351288	22	30635968	G	A	0.011507	-0.447123	0.0778491	9.65E-09	7213
TCN2	rs5997786	22	30858049	C	T	0.150908	0.209191	0.0229389	9.63E-20	7213
GUSB	rs2949690	7	66018255	C	T	0.445515	-0.177169	0.0165544	1.57E-26	7213
MUC16	rs193266507	19	8854282	T	C	0.0662692	-0.288148	0.0329812	2.95E-18	7213
MUC16	rs8110763	19	9020790	T	G	0.486829	-0.0966851	0.016631	6.38E-09	7213
CNN1	rs138405741	19	11543596	C	T	0.0318869	-0.325829	0.0475807	8.11E-12	7213
CR2	rs74810716	1	207333596	T	G	0.105157	0.153573	0.026717	9.39E-09	7213
CR2	rs61821130	1	207457543	C	A	0.112575	-0.237385	0.0263242	2.42E-19	7213
NCAN	rs2228603	19	19219115	C	T	0.0811036	-0.451966	0.030094	3.18E-50	7213
RNASE3	rs111391792	14	20875328	T	C	0.0737557	-0.370047	0.0311392	2.88E-32	7213
RNASE3	rs147307766	14	20917832	C	T	0.0497019	0.946463	0.0365163	1.07E-141	7213
RNASE3	rs150971918	14	20976668	G	C	0.0134479	-0.46435	0.0717985	1.06E-10	7213
FCN1	rs7037264	9	134883366	G	A	0.403022	0.489293	0.0158671	2.99E-196	7213
FCN1	rs7873100	9	134964997	C	G	0.261403	0.314048	0.0186976	3.91E-62	7213
FCRLB	rs61801180	1	161718974	G	C	0.0538611	0.697481	0.0360986	3.88E-81	7213
FCRLB	rs2880055	1	161952575	C	T	0.138639	0.256043	0.0238511	1.11E-26	7213
CFHR2	rs76541854	1	196479019	G	A	0.0203106	0.343847	0.0582575	3.75E-09	7213
CFHR2	rs10737679	1	196710106	G	T	0.208304	-0.777254	0.0183596	0	7213
CFHR2	rs150638768	1	196933743	C	T	0.0154582	-1.43682	0.064916	4.45E-105	7213
FBLN5	rs2267994	14	91890602	A	G	0.35644	0.14798	0.017247	1.15E-17	7213
FOLR2	rs80345326	11	72216368	T	C	0.0249549	0.393716	0.0529326	1.14E-13	7213
GC	rs7697091	4	71759380	A	C	0.360876	-0.160771	0.0171732	1.03E-20	7213

GC	rs2139646	4	71905686	A	G	0.35034	0.132789	0.0172946	1.83E-14	7213
GPX1	rs9823546	3	49668079	T	A	0.291834	-0.16557	0.0182205	1.29E-19	7213
HTRA1	rs7074542	10	122353283	C	T	0.308679	-0.190676	0.0179041	2.74E-26	7213
HEXIM1	rs1044977	17	45149847	T	C	0.25759	0.210601	0.0189678	2.06E-28	7213
IL6R	rs6688376	1	154353890	C	T	0.299043	-0.196202	0.0181997	6.82E-27	7213
IL6R	rs12753254	1	154444459	G	A	0.405864	0.992553	0.0121309	0	7213
IL6R	rs61103655	1	154657590	C	T	0.280743	-0.172403	0.0184929	1.48E-20	7213
ITGA2	rs246413	5	52982280	G	A	0.321226	0.143417	0.0177592	7.81E-16	7213
MAPK9	rs11741142	5	180307139	T	C	0.204977	0.340169	0.0202635	4.51E-62	7213
LAP3	rs114838597	4	17578371	C	T	0.0259947	0.520345	0.0520792	2.36E-23	7213
LIN7B	rs4802563	19	49116979	C	A	0.40933	0.162023	0.0167265	4.68E-22	7213
LILRA2	rs2151868	19	54593448	T	C	0.107584	-0.583312	0.0259809	5.79E-108	7213
LILRB3	rs112045636	19	54079321	G	A	0.0366006	0.302682	0.0438962	5.83E-12	7213
LILRB3	rs145888232	19	54211473	C	T	0.0223208	-0.496734	0.0563325	1.44E-18	7213
LILRB3	rs12978577	19	54213755	T	A	0.309511	0.523234	0.0167712	1.50E-200	7213
LILRB3	rs12984962	19	54265351	T	C	0.346458	-0.181135	0.0175479	8.31E-25	7213
XCL2	rs4656601	1	168538235	T	A	0.198045	0.221446	0.0205592	7.54E-27	7213
NLGN1	rs497383	3	173407133	A	G	0.270345	0.230434	0.0184662	2.26E-35	7213
HSPG2	rs12742444	1	21854230	C	T	0.0589214	0.731954	0.0345143	7.09E-97	7213
PSG1	rs2005772	19	42881078	G	A	0.200957	-0.142168	0.0202972	2.71E-12	7213
RBP4	rs10882283	10	93601207	A	C	0.385138	-0.18099	0.0170941	5.26E-26	7213
SMOC2	rs56296467	6	168463127	T	C	0.268751	-0.471633	0.0178607	9.25E-147	7213
SMOC2	rs187316322	6	168502959	G	A	0.0128934	0.444427	0.0732782	1.39E-09	7213
SMOC2	rs56284947	6	168595562	A	C	0.271524	-0.185596	0.0185868	2.50E-23	7213
TMEFF1	rs10989102	9	100475300	T	C	0.12221	-0.241732	0.0253666	2.11E-21	7213
BTD	rs62240447	3	15132809	A	G	0.0579509	-0.213645	0.0353668	1.61E-09	7213
BTD	rs144961978	3	15487998	T	C	0.0174685	-0.375771	0.0636946	3.81E-09	7213
BTD	rs35034250	3	15645027	C	T	0.0241231	-0.920398	0.0535202	5.44E-65	7213
BTD	rs13078881	3	15645186	G	C	0.0399972	-1.4571	0.0389051	1.11E-280	7213
COL10A1	rs9488842	6	116123005	A	C	0.26577	-0.362481	0.0183708	1.90E-84	7213
COL10A1	rs17078099	6	116594928	T	C	0.0177457	-0.387533	0.0627005	6.73E-10	7213
DKK2	rs6815075	4	107118897	G	A	0.11715	-0.145507	0.0258263	1.83E-08	7213
INHBC	rs141299103	12	57322004	C	T	0.0151809	0.418784	0.0681093	8.23E-10	7213
INHBC	rs12313306	12	57358071	C	T	0.234854	-1.15233	0.0140351	0	7213
INHBC	rs55852335	12	57459552	A	G	0.0480383	0.265297	0.0384286	5.50E-12	7213
INHBC	rs192828744	12	57508571	G	T	0.0237765	-0.570072	0.0545673	2.29E-25	7213
INHBC	rs141016935	12	57566868	C	T	0.0284902	0.293335	0.0501486	5.15E-09	7213
GPT	rs4244610	8	144512131	G	A	0.47553	-0.137154	0.0164884	1.06E-16	7213
FLT4	rs1130379	5	180612606	C	T	0.107584	-0.255045	0.0267445	1.98E-21	7213
CFHR5	rs6690982	1	196662999	G	A	0.220782	-0.318737	0.0199286	1.31E-56	7213
CFHR5	rs35662416	1	196998224	G	A	0.0239152	-1.20255	0.0519707	2.53E-114	7213
IGF2R	rs7775650	6	159820408	G	C	0.177457	0.221555	0.0216321	1.89E-24	7213
IGF2R	rs220727	6	159902832	T	C	0.229308	-0.109706	0.0197064	2.68E-08	7213

IGF2R	rs3777404	6	160077017	G	A	0.135311	0.870276	0.022201	7.36E-305	7213
NID2	rs941622	14	52032320	G	A	0.458894	-0.507152	0.0156856	2.74E-214	7213
NID2	rs1557192	14	52188223	G	A	0.471648	-0.145171	0.0165246	1.93E-18	7213
WIF1	rs462847	12	64920170	C	T	0.460003	-0.10588	0.0166097	1.95E-10	7213
TEC	rs4543082	4	48186832	C	G	0.202828	0.164147	0.0207069	2.58E-15	7213
EPHA4	rs4674597	2	221423124	T	C	0.431305	0.160647	0.0166189	5.68E-22	7213
EPHA4	rs16862777	2	221490753	G	C	0.274088	-0.291608	0.018375	8.95E-56	7213
EPHA4	rs2680844	2	221569000	A	G	0.077083	0.235331	0.0311113	4.39E-14	7213
LGR5	rs17109804	12	71551792	A	G	0.17004	0.156248	0.0218296	9.02E-13	7213
LGR5	rs35800540	12	71734869	C	T	0.0167753	-0.452132	0.0648785	3.48E-12	7213
TREM2	rs143332484	6	41161469	C	T	0.0104672	-1.5304	0.0796624	2.95E-80	7213
LGR4	rs2448001	11	27372266	G	A	0.361916	0.170415	0.0172143	5.85E-23	7213
UNC5D	rs3108622	8	35257097	G	A	0.433176	-0.290632	0.0164437	1.81E-68	7213
UNC5D	rs16884340	8	35731786	C	T	0.0101899	0.908818	0.0826236	6.40E-28	7213
DKKL1	rs1465697	19	49333989	C	T	0.246985	-0.160239	0.0190946	5.70E-17	7213
ACVRL1	rs78197611	12	51924840	C	T	0.0885207	0.496669	0.0285246	1.52E-66	7213
PACAP	rs116498185	5	139190262	C	T	0.0537224	0.20891	0.0366272	1.22E-08	7213
TLR1	rs77243175	4	38751996	A	C	0.124428	-0.168848	0.0250711	1.77E-11	7213
TLR1	rs5743618	4	38797027	C	A	0.274851	0.290832	0.0182813	4.91E-56	7213
MYOC	rs6660098	1	171471775	T	C	0.049286	0.210473	0.0382338	3.82E-08	7213
MYOC	rs235879	1	171646796	T	C	0.411757	-0.434479	0.01607	2.00E-153	7213
MYOC	rs56178029	1	171785024	C	T	0.260363	-0.212471	0.0187272	1.39E-29	7213
A1BG	rs1268538	19	58341997	C	T	0.047553	-1.12335	0.0369558	4.54E-191	7213
NUDT2	rs145758424	9	34040089	G	T	0.02953	0.873874	0.0478753	8.36E-73	7213
NUDT2	rs4879773	9	34302155	T	C	0.223971	-0.876654	0.0171821	0	7213
NUDT2	rs277587	9	34820473	G	A	0.381672	0.158165	0.0169216	1.18E-20	7213
FAIM	rs641320	3	138629115	G	A	0.0695966	-0.727406	0.0315316	1.14E-113	7213
GLRX3	rs12269149	10	130146915	C	T	0.283655	-0.126512	0.018459	7.79E-12	7213
C1QTNF9	rs9507306	13	24328550	C	A	0.0795092	0.19354	0.0305278	2.44E-10	7213
C1QTNF9	rs56069023	13	24357997	A	G	0.3136	-0.341222	0.0175055	1.65E-82	7213
AKR1B1	rs2229542	7	134450869	T	C	0.0122695	-0.907527	0.0740315	3.30E-34	7213
AKR1B1	rs782545	7	134503187	A	G	0.0304312	0.645917	0.0479378	6.93E-41	7213
GSN	rs10985196	9	121270766	C	A	0.20872	-0.158452	0.0201285	4.00E-15	7213
KIRREL2	rs35854130	19	35861033	G	T	0.208096	-0.17087	0.0203919	6.34E-17	7213
LRP10	rs182793305	14	22894897	G	A	0.0194094	0.531241	0.0590868	3.09E-19	7213
CDH17	rs2251734	8	94146031	G	A	0.278802	0.271719	0.0182769	2.89E-49	7213
CDH17	rs12056840	8	94191166	G	A	0.469777	-0.340079	0.0161707	2.40E-95	7213
RSPO1	rs36043533	1	37613845	T	G	0.051019	0.760438	0.0367423	1.74E-92	7213
RSPO1	rs116685741	1	37627295	C	T	0.0194094	-0.395959	0.0600941	4.74E-11	7213
ENO3	rs238238	17	4953081	A	G	0.292597	0.359933	0.017708	2.33E-89	7213
LY75	rs72947565	2	159466884	T	C	0.0371551	0.30884	0.0435168	1.40E-12	7213
LY75	rs7599157	2	159900954	C	T	0.393456	-0.735907	0.0148491	0	7213
LY75	rs3063683	2	160009707	C	A	0.199016	-0.406598	0.0203444	1.56E-86	7213

APOA1BP	rs4661190	1	156594814	G	A	0.0286289	-1.46466	0.0465662	1.31E-203	7213
NPPB	rs198375	1	11853700	T	C	0.403993	0.0956982	0.0170112	1.92E-08	7213
COL6A2	rs2839107	21	46082477	G	A	0.44198	0.128349	0.0165668	1.07E-14	7213
COL6A2	rs35548026	21	46132295	G	A	0.0869957	-0.548396	0.0288366	1.01E-78	7213
COL6A2	rs148337125	21	46132608	T	G	0.0250936	0.511975	0.0517792	6.58E-23	7213
HDGF	rs150063652	1	156656083	C	T	0.0250243	-0.288445	0.0523697	3.76E-08	7213
HDGF	rs3806417	1	156741831	C	A	0.346666	-0.777225	0.0148475	0	7213
LECT2	rs31263	5	135633272	G	C	0.281228	0.120002	0.0182442	5.12E-11	7213
LECT2	rs4272127	5	135867824	A	G	0.336129	-0.183687	0.0174219	8.39E-26	7213
LECT2	rs248167	5	135956428	A	G	0.367046	0.708789	0.0152479	0	7213
LECT2	rs142743574	5	136237738	C	G	0.011715	-0.459621	0.0776348	3.36E-09	7213
REG1B	rs11126696	2	79096762	A	G	0.384167	-0.324922	0.0168078	3.22E-81	7213
REG1B	rs12614387	2	79132617	C	G	0.170248	-0.184541	0.0218256	3.34E-17	7213
REG1B	rs17016642	2	79249249	A	G	0.115694	-0.147681	0.0259579	1.33E-08	7213
SCUBE3	rs942374	6	35176115	T	C	0.160613	-0.356218	0.022472	1.18E-55	7213
ENGASE	rs116735850	17	79041459	C	T	0.016498	-0.759179	0.0644427	9.58E-32	7213
ENGASE	rs56107536	17	79074958	C	A	0.133509	0.681166	0.0231459	7.15E-180	7213
ENGASE	rs148222910	17	79142808	C	A	0.0155275	0.457122	0.0673373	1.22E-11	7213
ENGASE	rs78418700	17	79157247	C	T	0.0343824	-0.276526	0.045811	1.66E-09	7213
SIGLEC5	rs2864096	19	51488518	T	C	0.24234	-0.177178	0.0194598	1.10E-19	7213
SIGLEC5	rs7250849	19	51655063	G	T	0.113822	-0.951534	0.0232534	0	7213
SIGLEC5	rs12974855	19	51685899	G	A	0.129558	0.461729	0.024216	4.09E-79	7213
SIGLEC5	rs8110040	19	51849623	G	A	0.0986413	0.220021	0.0277341	2.46E-15	7213
PDE5A	rs58583086	4	119635207	A	G	0.37273	0.502202	0.0163889	5.88E-194	7213
PDE5A	rs114110723	4	119728725	T	C	0.0436711	-0.242156	0.0409578	3.53E-09	7213
PDE5A	rs143179501	4	119836365	C	A	0.0170525	-0.499991	0.0637844	5.20E-15	7213
NME4	rs6600214	16	394814	C	T	0.319077	0.173731	0.0176289	9.08E-23	7213
NME4	rs7202319	16	439985	A	G	0.0566339	-0.255287	0.0358931	1.25E-12	7213
CDCP1	rs17077267	3	45097023	C	T	0.147026	-0.303925	0.0232302	1.13E-38	7213
CDCP1	rs2276862	3	45146293	C	G	0.178705	-0.37749	0.0211676	1.20E-69	7213
APOL3	rs147323157	22	36002905	C	T	0.0229447	-0.407921	0.0555246	2.26E-13	7213
APOL3	rs132631	22	36146146	A	C	0.165742	-1.0582	0.0185308	0	7213
ATXN3	rs1133441	14	91968721	T	A	0.33093	-0.59418	0.0162469	8.90E-269	7213
ATXN3	rs10150636	14	92031851	T	C	0.214266	-0.226359	0.0202057	6.84E-29	7213
ATXN3	rs1465126	14	92221563	T	G	0.105573	0.233942	0.0269558	4.89E-18	7213
COL6A1	rs4819142	21	45702121	G	A	0.466865	-0.350272	0.0159741	3.16E-103	7213
COL6A1	rs7283989	21	46000847	G	A	0.449189	-0.727731	0.0142031	0	7213
COL6A1	rs915808	21	46056787	A	C	0.404478	0.235796	0.0168771	8.65E-44	7213
SCO2	rs11479	22	50525807	G	A	0.08859	-0.278112	0.0289253	9.34E-22	7213
MAPRE2	rs1443641	18	35110792	A	T	0.443089	-0.371504	0.016118	1.76E-113	7213
GSTZ1	rs7160188	14	77320018	G	C	0.299182	0.708867	0.0161192	0	7213
GSTZ1	rs28516852	14	77519054	T	G	0.0916401	-0.203128	0.0288869	2.23E-12	7213
GSTZ1	rs28433606	14	77665261	T	A	0.0804797	-0.275438	0.0303325	1.37E-19	7213

PHPT1	rs872463	9	136850355	A	G	0.262096	0.558795	0.0177074	7.41E-205	7213
ADAMTSL1	rs117766170	9	18406352	G	A	0.0624567	0.355752	0.0339049	1.43E-25	7213
ENPP2	rs13267597	8	119641985	T	G	0.323582	-0.191541	0.0176274	2.72E-27	7213
MDGA1	rs79144597	6	37323009	G	T	0.0576043	-0.237994	0.0357512	3.00E-11	7213
MDGA1	rs72857324	6	37564376	C	T	0.0208651	-0.383144	0.0576969	3.35E-11	7213
MDGA1	rs183635273	6	37579691	A	G	0.013032	0.597447	0.0731479	3.68E-16	7213
MDGA1	rs6937280	6	37704146	T	G	0.475322	0.985091	0.011813	0	7213
CADM2	rs9880919	3	85412152	G	A	0.239983	0.154652	0.0195288	2.75E-15	7213
OMG	rs72813607	17	31134507	G	A	0.131568	-0.195295	0.0245913	2.30E-15	7213
RNASET2	rs9457245	6	166927227	C	T	0.0745182	-0.841235	0.0299113	3.16E-165	7213
RNASET2	rs6456141	6	166949107	A	C	0.248024	-0.260467	0.0191144	9.11E-42	7213
CD14	rs114928388	5	140383553	G	A	0.0233606	0.335137	0.0552954	1.42E-09	7213
CD14	rs5744441	5	140637262	G	A	0.237627	-0.309891	0.0188958	2.25E-59	7213
SEMA4A	rs12401997	1	156178005	C	A	0.392209	0.268619	0.0167405	5.85E-57	7213
SLITRK6	rs9547187	13	85358375	C	A	0.221475	-0.346282	0.0193512	4.20E-70	7213
SLITRK6	rs688445	13	85752817	G	A	0.260848	0.112493	0.0188616	2.58E-09	7213
TLR3	rs66624661	4	186073978	G	A	0.33405	0.234095	0.0173647	6.31E-41	7213
TLR3	rs34357460	4	186090335	G	A	0.344309	-0.689436	0.0155856	0	7213
TLR3	rs7667777	4	186204923	C	G	0.417163	-0.11634	0.0167026	3.56E-12	7213
DBI	rs6714264	2	119390517	T	C	0.193609	-0.529076	0.0200986	6.70E-146	7213
DBI	rs79258303	2	119443461	A	C	0.0140718	0.433993	0.0695915	4.73E-10	7213
GSTA1	rs9474314	6	52737562	T	C	0.104187	0.313572	0.0272082	1.82E-30	7213
GSTA1	rs9395826	6	52814892	C	T	0.47553	-0.645362	0.0149081	0	7213
PDGFD	rs1917446	11	103627236	T	C	0.150076	-0.21104	0.0231672	1.06E-19	7213
PDGFD	rs10895596	11	104161315	G	A	0.300846	-0.52836	0.0171323	2.74E-196	7213
PDGFD	rs144268268	11	104525727	G	A	0.0271732	0.273472	0.0497172	3.92E-08	7213
BLVRB	rs268695	19	40455079	G	T	0.297033	0.133347	0.0182058	2.66E-13	7213
IRF3	rs34165679	19	49510136	T	C	0.130251	-0.21329	0.0247316	7.85E-18	7213
IRF3	rs10415576	19	49661133	T	C	0.372591	-0.452409	0.016293	2.61E-161	7213
KIR2DL3	rs11672845	19	54570466	C	T	0.0725773	0.20032	0.0320971	4.59E-10	7213
KIR2DL3	rs1654659	19	54683871	T	C	0.131637	0.173444	0.0245953	1.93E-12	7213
KIR2DL3	rs4806594	19	54843895	A	G	0.187439	0.245625	0.0207917	6.53E-32	7213
DCLK1	rs7339267	13	36073931	G	A	0.217455	0.309996	0.0200156	3.00E-53	7213
DDOST	rs150466875	1	20651922	T	G	0.0811729	-0.369582	0.0301824	3.90E-34	7213
ANXA4	rs2228203	2	69806452	C	T	0.196659	-0.51625	0.0200114	1.89E-140	7213
CALCB	rs7104596	11	15057506	T	C	0.274643	-0.300296	0.0184656	1.98E-58	7213
RAB5A	rs4241540	3	19941480	G	C	0.371274	-0.116966	0.0172473	1.28E-11	7213
TCL1A	rs78986913	14	95696081	G	A	0.0456121	-0.492879	0.039601	3.37E-35	7213
OGN	rs10992291	9	92270727	T	C	0.367947	0.567692	0.0158004	3.29E-260	7213
LCP1	rs12858505	13	46124637	G	A	0.218494	-0.16006	0.0201661	2.38E-15	7213
GUK1	rs11580599	1	228148258	C	T	0.0422848	1.32568	0.0381448	8.00E-245	7213
GUK1	rs7533319	1	228451441	A	G	0.196659	-0.150622	0.0207569	4.39E-13	7213
CNPY3	rs9471969	6	42938646	G	T	0.250173	-0.185098	0.0190482	3.48E-22	7213

BDH2	rs223507	4	102712658	G	A	0.339803	0.132852	0.0173056	1.85E-14	7213
BDH2	rs78336913	4	103190257	C	T	0.174823	0.356888	0.0212814	5.97E-62	7213
KREMEN1	rs2205771	22	29098605	G	A	0.151116	0.427977	0.0228926	3.37E-76	7213
ACADM	rs7534754	1	75719623	G	A	0.304242	0.316964	0.017587	4.61E-71	7213
ACAT2	rs25683	6	159775311	A	G	0.418688	0.344014	0.0164861	6.13E-94	7213
ZADH2	rs12969004	18	75208110	G	A	0.420352	-0.165514	0.0168061	9.68E-23	7213
AIDA	rs28709375	1	222649657	A	G	0.277277	0.260654	0.0184418	9.26E-45	7213
CHMP2B	rs300978	3	87274184	G	T	0.40933	0.556863	0.0159171	7.98E-248	7213
STMN1	rs807060	1	25914964	G	T	0.430611	-0.0984897	0.0167824	4.59E-09	7213
ABHD10	rs57797651	3	111994785	A	G	0.20061	-0.125478	0.0207362	1.51E-09	7213
AKR1C3	rs4880709	10	5109163	G	A	0.339387	0.135515	0.017398	7.69E-15	7213
UBE2K	rs58339883	4	39781456	A	C	0.129974	0.265989	0.0245065	3.10E-27	7213
STARD5	rs4392019	15	81324218	C	T	0.0322335	0.634692	0.0465856	9.42E-42	7213
STARD5	rs28555408	15	81345531	C	T	0.0641897	-0.530498	0.0330579	5.72E-57	7213
PFKM	rs3742074	12	48063602	C	T	0.168099	0.143149	0.0224181	1.82E-10	7213
RBM17	rs11256726	10	6100347	T	C	0.0375711	-0.745598	0.0429864	4.65E-66	7213
ADH1A	rs189407772	4	99225517	A	G	0.0202412	-1.01286	0.0578836	3.56E-67	7213
ADH1A	rs1154446	4	99367790	C	A	0.303688	0.125919	0.018054	3.34E-12	7213
SFRP4	rs75207237	7	37932788	T	A	0.124289	0.402321	0.0249045	1.08E-57	7213
HARS	rs112352450	5	140782616	G	A	0.0196867	0.463388	0.0600616	1.37E-14	7213
CP	rs13089226	3	149080893	C	T	0.0432552	0.272277	0.0406211	2.20E-11	7213
GOLM1	rs11141212	9	86074933	G	A	0.0173991	1.159	0.0627663	2.00E-74	7213
GOLM1	rs10868368	9	86085724	A	G	0.0607237	0.340189	0.034908	2.65E-22	7213
MX1	rs430948	21	41413952	A	G	0.135311	0.158829	0.0243807	7.78E-11	7213
MX1	rs464783	21	41424553	C	T	0.406835	-0.413879	0.0163459	1.40E-135	7213
SH3BGRL3	rs111486799	1	26251494	G	A	0.197976	-0.365761	0.0203917	2.03E-70	7213
SH3BGRL3	rs4659423	1	26296483	A	C	0.0703591	-1.17867	0.029352	2.12580143239176e-318	7213
NMI	rs6713190	2	151302945	T	C	0.484195	-0.195191	0.01646	3.86E-32	7213
ANXA11	rs79434154	10	80009759	A	C	0.155483	0.183908	0.0229717	1.37E-15	7213
ANXA11	rs2573346	10	80158285	G	A	0.438167	-0.61607	0.015184	0	7213
RAB21	rs2864808	12	71746926	G	A	0.1507	0.20531	0.0231769	1.01E-18	7213
HSPA13	rs2822638	21	14372785	C	T	0.251213	0.26387	0.0188482	5.86E-44	7213
GIF	rs150884181	11	59843108	A	G	0.0106059	-0.5299	0.0814599	8.29E-11	7213
ACOT13	rs4544900	6	24677760	C	T	0.254194	0.276588	0.0189966	2.37E-47	7213
TRIP10	rs62125134	19	6724433	G	A	0.224387	0.112996	0.0198151	1.23E-08	7213
TRIP10	rs1054060	19	6752423	T	C	0.18945	-0.152566	0.0211647	6.23E-13	7213
TRIP10	rs8106212	19	6802560	C	T	0.0142798	0.561283	0.0700127	1.26E-15	7213
TRIP10	rs201831100	19	6804082	T	C	0.0648135	-0.231856	0.0342279	1.35E-11	7213
EPHB1	rs9862761	3	134783855	G	T	0.426937	-0.136938	0.0165895	1.80E-16	7213
EPHB1	rs10935143	3	134946317	G	A	0.447525	0.398707	0.0161256	1.27E-129	7213
CD46	rs60467252	1	207651037	G	A	0.147581	0.208736	0.023161	2.54E-19	7213
CD46	rs2488252	1	207764883	C	T	0.410024	0.593582	0.0153005	3.43E-299	7213
APOA4	rs180365	11	116725458	C	T	0.450853	0.125898	0.0168505	8.86E-14	7213

APOA4	rs187929675	11	117240652	C	T	0.0165673	-0.589817	0.0648363	1.18E-19	7213
TBCB	rs2231569	19	36114973	G	T	0.0415223	0.822433	0.0405437	5.12E-89	7213
TBCB	rs45471297	19	36115262	C	G	0.0133786	-0.686723	0.0724987	3.64E-21	7213
TPP1	rs11040938	11	6633382	A	G	0.422432	0.229556	0.016762	3.65E-42	7213
BTN3A3	rs13212534	6	25982782	G	A	0.0704284	-0.984923	0.0304233	8.46E-215	7213
BTN3A3	rs114124677	6	25992305	G	A	0.0125468	0.507153	0.0746018	1.14E-11	7213
PSME2	rs2236352	14	24141524	T	C	0.252599	0.312178	0.0186124	5.72E-62	7213
PSME2	rs7146672	14	24145152	T	G	0.0128934	-0.606768	0.0723321	5.86E-17	7213
OVCA2	rs76946443	17	2033909	G	C	0.0835297	-0.405145	0.0298805	2.25E-41	7213
OVCA2	rs145234879	17	2042060	C	A	0.0142105	1.40274	0.0681679	1.77E-91	7213
UGT1A1	rs114252547	2	233402625	T	G	0.0335505	-0.296349	0.0461065	1.38E-10	7213
UGT1A1	rs887829	2	233759924	C	T	0.329821	-0.607257	0.0162861	1.99E-278	7213
HSPH1	rs9538558	13	31143416	T	A	0.225912	0.199646	0.0199115	1.66E-23	7213
PPP1R1A	rs10876568	12	54590778	G	C	0.210453	0.148809	0.020392	3.25E-13	7213
IDI1	rs4880760	10	1048969	C	G	0.0304312	-0.350284	0.047909	2.93E-13	7213
FKBP4	rs56196860	12	2799164	C	A	0.0289755	-1.06599	0.0480182	1.12E-105	7213
FKBP4	rs145074222	12	2854186	C	T	0.0122002	0.51139	0.0760598	1.91E-11	7213
SAR1A	rs4746970	10	70146394	T	C	0.419867	0.396475	0.0160422	1.62E-129	7213
CHMP2A	rs11673465	19	58527528	T	C	0.203244	0.140955	0.0207526	1.19E-11	7213
IVD	rs12440453	15	40399596	C	T	0.317968	0.219407	0.0177	6.23E-35	7213
HADH	rs72890511	4	107967387	T	C	0.0790933	0.260889	0.0306342	1.98E-17	7213
FIS1	rs74662330	7	101222585	C	T	0.0516429	-0.70054	0.0368752	1.45E-78	7213
TP53I3	rs145582435	2	23680301	G	A	0.0402052	0.241463	0.0421012	1.01E-08	7213
TP53I3	rs1134516	2	24119662	G	A	0.160821	-0.9954	0.0194667	0	7213
TP53I3	rs3731626	2	24204995	G	A	0.28109	-0.12266	0.0182595	1.99E-11	7213
TP53I3	rs11884143	2	24490061	C	G	0.180369	-0.134763	0.0216385	4.99E-10	7213
CRYBB1	rs5761621	22	26603394	A	G	0.325662	-0.201325	0.0177064	1.05E-29	7213
UGDH	rs1138891	4	39532402	T	G	0.366699	-0.455336	0.0163977	2.53E-161	7213
CAB39	rs7604335	2	230754380	T	C	0.244836	-0.232338	0.0191964	2.14E-33	7213
DCXR	rs7207947	17	82187415	A	T	0.415777	0.140266	0.0166362	4.09E-17	7213
NUDT5	rs7099650	10	12159177	C	T	0.489741	-0.100384	0.0167265	2.05E-09	7213
RHOC	rs7415820	1	112626827	G	A	0.475461	0.220126	0.0165674	8.11E-40	7213
SNUPN	rs7170787	15	75593089	G	A	0.250659	0.359476	0.0187648	7.95E-80	7213
SNUPN	rs77237106	15	75676994	C	T	0.173021	-0.183351	0.0220612	1.12E-16	7213
NCF1	rs148400673	7	74645788	A	G	0.0395813	-0.669261	0.041983	2.97E-56	7213
NCF1	rs148581667	7	75044775	G	A	0.0551782	0.734063	0.0354647	1.68E-92	7213
PCNP	rs13060373	3	101631980	A	G	0.499445	-0.346945	0.0159757	2.37E-101	7213
ACAA1	rs2229528	3	38125604	A	G	0.0438791	-0.714983	0.0396133	2.92E-71	7213
MMAB	rs11065640	12	109132145	C	T	0.184597	-0.16696	0.0214829	8.81E-15	7213
MMAB	rs7970557	12	109564328	C	T	0.178913	-0.67284	0.0200454	1.21E-229	7213
RCL	rs114371775	6	43231992	T	C	0.105296	-0.276876	0.0265871	3.23E-25	7213
HPCAL1	rs7581642	2	10337292	C	T	0.162623	0.130405	0.0224445	6.51E-09	7213
PMM2	rs34258285	16	8813057	A	C	0.028005	-0.660606	0.049547	4.43E-40	7213

IMPA1	rs2142316	8	81652967	A	G	0.30854	-0.147208	0.0178079	1.63E-16	7213
PGLS	rs62125238	19	17439138	C	T	0.145917	0.16018	0.0235508	1.12E-11	7213
PGLS	rs73020480	19	17518363	G	A	0.202482	0.4129	0.0199256	1.06E-92	7213
NANS	rs7855984	9	98056008	G	A	0.244143	0.201298	0.0191437	1.13E-25	7213
NANS	rs7025781	9	98226228	G	A	0.309025	0.105311	0.0181242	6.49E-09	7213
BPHL	rs3799225	6	3152008	A	G	0.049286	0.217967	0.0387458	1.92E-08	7213
BPNT1	rs7530481	1	220063279	T	C	0.29225	0.23611	0.0181907	4.25E-38	7213
SUGT1	rs185192949	13	52645515	C	T	0.0406211	-0.263108	0.0421956	4.76E-10	7213
FAHD1	rs3743853	16	1827557	G	A	0.158741	-0.326481	0.0223296	9.90E-48	7213
NMT2	rs80260329	10	15155677	C	T	0.113822	-0.184919	0.0259656	1.17E-12	7213
NMT2	rs11259539	10	15197030	T	C	0.402814	-0.227925	0.0166714	5.00E-42	7213
S100A14	rs9330298	1	153617778	C	A	0.475184	-0.171167	0.0165494	6.73E-25	7213
IDI2	rs1044261	10	1019770	C	T	0.0770137	-0.556295	0.0306761	6.63E-72	7213
IDI2	rs7915650	10	1103450	C	A	0.139817	0.209887	0.0242013	5.16E-18	7213
S100A16	rs9729962	1	153596510	T	C	0.0878275	0.181925	0.0291697	4.72E-10	7213
S100A16	rs9729773	1	153622147	A	G	0.482116	-0.319067	0.0160774	2.21E-85	7213
S100A16	rs7543790	1	153697837	C	T	0.0616942	-0.192875	0.0347087	2.84E-08	7213
PPCS	rs4660635	1	42439366	G	A	0.367877	-0.45315	0.0162303	5.55E-163	7213
PPCS	rs7354966	1	42546161	G	A	0.486344	-0.102708	0.0166759	7.71E-10	7213
PPME1	rs76970567	11	74205493	C	G	0.0535838	-0.298051	0.0366809	5.20E-16	7213
NT5M	rs16961516	17	17304918	G	A	0.0424234	0.264647	0.0409698	1.12E-10	7213
ASF1A	rs4946366	6	118811617	C	T	0.180854	0.130473	0.0215711	1.54E-09	7213
AKR7A3	rs72645633	1	18925408	C	A	0.0381256	-0.450127	0.0432664	3.60E-25	7213
AKR7A3	rs72645644	1	18937833	C	T	0.0939969	-0.171124	0.0281939	1.35E-09	7213
AKR7A3	rs55717136	1	19246561	A	T	0.373215	-0.483248	0.0163333	1.12E-181	7213
AKR7A3	rs114458471	1	19443436	T	A	0.0340358	0.330897	0.0461708	8.44E-13	7213
PDCD6IP	rs116488831	3	33366277	G	T	0.0330653	-0.43072	0.0462642	1.67E-20	7213
PDCD6IP	rs150059308	3	33453499	G	A	0.0131013	-0.506032	0.0730554	4.68E-12	7213
PDCD6IP	rs1141054	3	33798622	T	C	0.267018	-0.669194	0.0170219	1.85E-306	7213
VAT1	rs4239148	17	43077840	G	A	0.338209	-0.0959998	0.0175695	4.81E-08	7213
RTN4IP1	rs17565019	6	106610517	A	C	0.3554	0.0942912	0.0172722	4.94E-08	7213
PCK1	rs2070756	20	57564347	T	C	0.253778	-0.259345	0.0188441	1.47E-42	7213
LDLRAP1	rs4523506	1	25566730	G	T	0.459102	-0.358204	0.0162908	9.04E-104	7213
TARS	rs3777083	5	33442018	A	G	0.191876	-0.216131	0.0208396	5.03E-25	7213
TARS	rs72737345	5	33450267	A	C	0.0210037	-0.474841	0.0578146	2.53E-16	7213
ADH6	rs17595424	4	99072718	G	T	0.105435	0.50593	0.0266514	1.88E-78	7213
ADH6	rs28730611	4	99079107	T	C	0.0381949	-1.16016	0.0415317	3.87E-163	7213
UBE2F	rs2326005	2	238008106	G	A	0.451684	0.118121	0.0166994	1.65E-12	7213
GCLM	rs7521934	1	93939485	T	C	0.493831	0.234163	0.0165186	5.19E-45	7213
GCLM	rs486879	1	94012869	C	T	0.195203	0.21781	0.0206765	9.24E-26	7213
THG1L	rs6896064	5	157548008	T	G	0.154097	0.236574	0.0229024	7.70E-25	7213
THG1L	rs7700500	5	157765194	T	A	0.34313	-0.802919	0.0147158	0	7213
THG1L	rs559165782	5	158026335	A	G	0.0153889	-0.421718	0.0667406	2.79E-10	7213

IL11RA	rs11575578	9	34656482	G	A	0.0639817	0.943428	0.0319229	2.71E-181	7213
CNRIP1	rs7604489	2	68352779	C	T	0.153681	-0.815743	0.0210461	1.05E-298	7213
CNRIP1	rs17035375	2	68371771	C	T	0.282823	0.127838	0.0186139	7.06E-12	7213
CNRIP1	rs75085051	2	68757741	C	G	0.0220435	-0.402003	0.0565878	1.33E-12	7213
SRA1	rs801460	5	140552345	C	T	0.461043	0.461083	0.0156559	4.02E-180	7213
SH3GLB2	rs11537529	9	129028252	C	T	0.455636	-0.576641	0.0153569	5.31E-282	7213
HEBP1	rs79211223	12	12939849	T	C	0.109109	-0.415762	0.0260328	1.88E-56	7213
HEBP1	rs1941	12	12975329	C	G	0.0337585	-1.61272	0.0419106	8.25E-295	7213
HEBP1	rs1291356	12	13005006	T	A	0.354568	-0.234866	0.0170045	7.55E-43	7213
PGP	rs116977380	16	2213835	C	T	0.0368085	0.884351	0.0432124	1.58E-90	7213
PGP	rs62040664	16	2275108	C	T	0.0115763	-0.565318	0.0770473	2.42E-13	7213
HSPC159	rs76392037	2	64341766	A	C	0.0528906	0.57949	0.0366218	1.82E-55	7213
CPOX	rs1675513	3	98589854	A	G	0.155552	-0.267199	0.0226839	9.75E-32	7213
CMPK1	rs35687416	1	47368537	G	T	0.0466519	-0.653522	0.0387567	1.34E-62	7213
ANXA7	rs117332875	10	73370976	C	G	0.0203106	-0.439824	0.0587773	8.13E-14	7213
DTD2	rs17097898	14	31449817	C	T	0.127825	-0.460686	0.024394	1.11E-77	7213
DTD2	rs10135385	14	31481137	T	C	0.0104672	-0.972884	0.0808681	5.08E-33	7213
DTD2	rs77004407	14	31646022	C	A	0.0500485	0.333523	0.0382597	3.49E-18	7213
CCL15	rs1617347	17	35959517	T	C	0.0384722	0.321812	0.0430864	9.03E-14	7213
CCL15	rs1734954	17	36010848	G	C	0.0638431	1.38857	0.0296152	0	7213
GRHPR	rs4878690	9	37422659	C	T	0.312838	0.367175	0.0173003	5.11E-97	7213
GRHPR	rs7021678	9	37504523	C	T	0.213573	0.114761	0.0202074	1.41E-08	7213
SEPW1	rs11881893	19	47777295	G	A	0.492306	-0.311165	0.0163912	1.86E-78	7213
ASRGL1	rs3017096	11	62410486	T	C	0.454041	0.140228	0.0166841	5.11E-17	7213
RTP4	rs1878861	3	187375904	G	A	0.40628	0.506623	0.0157481	2.65E-212	7213
PDHX	rs286879	11	34672106	C	G	0.11403	-0.188052	0.0261091	6.51E-13	7213
PDHX	rs2915221	11	34892933	C	A	0.316096	0.335834	0.0173887	4.51E-81	7213
PDHX	rs3903687	11	35310095	G	A	0.391169	-0.11506	0.0169469	1.22E-11	7213
CPLX1	rs11248039	4	772737	C	T	0.335228	-0.144202	0.0176599	3.75E-16	7213
IDH1	rs34599179	2	208243577	T	C	0.0108831	-1.82125	0.0772311	1.65E-118	7213
IDH1	rs3928183	2	208247493	T	C	0.0562873	-0.845273	0.0348089	2.84E-125	7213
PSMB4	rs1045628	1	150964427	T	C	0.0432552	-0.22575	0.0412143	4.46E-08	7213
PSMB4	rs4603	1	151401549	T	C	0.180923	-0.710493	0.0196809	1.74E-262	7213
PSMB4	rs191519987	1	151688522	C	T	0.0158741	-0.443419	0.0666367	3.06E-11	7213
PSAT1	rs2277148	9	78297199	G	C	0.215098	-0.390402	0.0196511	1.49E-85	7213
DECR2	rs1204505	16	412881	A	T	0.478164	0.276776	0.0161617	1.78E-64	7213
RDX	rs7935163	11	110336594	T	C	0.356024	-0.0946592	0.0172507	4.22E-08	7213
GLRX	rs6556884	5	95825636	G	A	0.24844	-0.121341	0.0191124	2.30E-10	7213
ST13	rs138337	22	40835049	A	G	0.467559	0.28252	0.0164284	5.46E-65	7213
AKR1C4	rs6601924	10	5205339	T	C	0.154998	-1.0443	0.019441	0	7213
AKR1D1	rs2306847	7	138113847	A	T	0.210523	-0.186947	0.0204084	6.63E-20	7213
ALKBH3	rs2292889	11	43920227	C	G	0.266394	-0.216182	0.0186154	6.66E-31	7213
ARF4	rs149547076	3	57571717	T	C	0.011715	0.45401	0.077171	4.20E-09	7213

ARF4	rs141818286	3	57935360	A	G	0.0524747	0.242484	0.0372137	7.71E-11	7213
AS3MT	rs79254677	10	102931810	A	G	0.111812	-0.169067	0.0264914	1.86E-10	7213
DDX19A	rs61757207	16	70324592	A	G	0.0140025	-1.03263	0.0703066	3.82E-48	7213
PPIC	rs6867714	5	123046364	T	G	0.3014	-0.6663	0.0161161	0	7213
PPIC	rs7715941	5	123173258	G	A	0.292735	0.141183	0.0181151	7.42E-15	7213
ITLN1	rs61803286	1	160861601	C	T	0.0778456	0.181304	0.0309858	5.09E-09	7213
LRIG1	rs2306272	3	66384219	T	C	0.281922	-0.753717	0.0163214	0	7213
LRIG1	rs147127798	3	66444300	G	T	0.0320948	0.26755	0.0471681	1.46E-08	7213
LRIG1	rs138006212	3	66825044	A	G	0.0348676	-0.28395	0.0452311	3.63E-10	7213
SAA2	rs11024589	11	18256876	A	C	0.104741	-0.578967	0.0261726	5.77E-105	7213
SAA2	rs76204461	11	18493515	C	T	0.0752114	0.180595	0.031359	8.81E-09	7213
SERPINB13	rs77157727	18	63586727	G	A	0.0305005	-0.498763	0.0481303	5.48E-25	7213
PRSS3	rs142302680	9	33566625	G	A	0.0137252	-0.470035	0.0710951	4.08E-11	7213
PRSS3	rs83921	9	33797058	A	T	0.37273	-0.550732	0.0160807	2.58E-238	7213
PRSS3	rs11506602	9	34129873	A	G	0.404755	-0.122711	0.0167934	3.02E-13	7213
AIF1L	rs11244284	9	131098400	T	C	0.286566	-0.544168	0.0173408	9.78E-203	7213
AIF1L	rs2767651	9	131269532	A	G	0.255164	0.129886	0.0188372	5.84E-12	7213
CEACAM8	rs10420796	19	42569106	A	G	0.0900458	-0.178236	0.0289912	8.27E-10	7213
COL2A1	rs719008	12	48022269	G	T	0.390683	-0.449708	0.0163818	6.96E-158	7213
COL2A1	rs12423404	12	48314785	A	G	0.22203	-0.141257	0.019828	1.15E-12	7213
CHST4	rs7200431	16	71492891	T	A	0.297934	0.203281	0.0179653	1.95E-29	7213
GREM1	rs10318	15	32733778	C	T	0.179537	0.840389	0.0192587	0	7213
GREM1	rs12594918	15	32833282	T	G	0.194579	-0.138028	0.0208353	3.73E-11	7213
GREM1	rs17816447	15	32843327	C	T	0.023222	0.421742	0.0546913	1.41E-14	7213
CD97	rs3786656	19	14395852	C	T	0.282892	0.122519	0.0182822	2.22E-11	7213
CLSTN2	rs9863263	3	139767054	A	G	0.0610703	0.592833	0.0344858	6.07E-65	7213
CLSTN2	rs2114164	3	139884656	A	G	0.438375	-0.182282	0.0166339	1.00E-27	7213
DNAJB4	rs7514180	1	77995251	G	A	0.206779	-0.34905	0.0199062	1.92E-67	7213
GBP2	rs10922556	1	89054960	C	T	0.215791	-0.161393	0.0201694	1.42E-15	7213
GPR56	rs1801257	16	57655473	C	G	0.488909	0.162996	0.0165	7.20E-23	7213
GSTM4	rs493972	1	109626266	C	G	0.372591	-0.218772	0.0171653	8.28E-37	7213
GSTM4	rs687643	1	109669912	T	C	0.482532	-0.391214	0.0160881	1.33E-125	7213
GSTM4	rs11102002	1	109757655	C	T	0.0519895	-0.23108	0.0376896	9.19E-10	7213
HS6ST3	rs12860877	13	96036886	T	C	0.460627	0.128073	0.016601	1.37E-14	7213
HDAC2	rs13212298	6	113921735	T	C	0.260918	-0.10362	0.0189301	4.55E-08	7213
HDGFRP3	rs28479338	15	83071593	T	C	0.0768751	0.213411	0.0309825	6.13E-12	7213
ITPA	rs6139031	20	3209336	G	A	0.226882	-0.491933	0.0189817	1.14E-141	7213
ITPA	rs6139042	20	3243067	C	T	0.183072	-0.82735	0.0188608	0	7213
ITPA	rs676141	20	3659124	T	A	0.100236	-0.304003	0.0277573	1.07E-27	7213
AMY2A	rs12076610	1	103521319	G	T	0.093789	0.689138	0.0274343	1.47E-133	7213
AMY2A	rs114500706	1	103849546	A	G	0.0469985	-0.682034	0.0388132	1.02E-67	7213
AMY2A	rs4847148	1	103907224	C	A	0.0154582	-0.561542	0.0670638	6.67E-17	7213
CD68	rs9901673	17	7580783	C	A	0.163108	0.587568	0.0213561	1.51E-158	7213

SLIT2	rs6833662	4	19825635	A	G	0.305143	-0.0984644	0.0178112	3.35E-08	7213
SLIT2	rs587668	4	20189349	C	T	0.297241	0.156569	0.0181881	9.00E-18	7213
TGM4	rs6650899	3	44900485	A	G	0.418688	-0.11859	0.0167544	1.60E-12	7213
TGM2	rs13039547	20	38169451	C	G	0.374532	-0.209571	0.0169885	1.29E-34	7213
SH3BGRL2	rs2245410	6	79616140	A	G	0.442188	-0.426901	0.0160201	2.67E-149	7213
SH3BGRL2	rs6923178	6	79684023	C	G	0.129558	-0.182569	0.024741	1.77E-13	7213
UBLCP1	rs13158921	5	159222864	C	T	0.0165673	-0.464805	0.0649771	9.30E-13	7213
MTHFSD	rs9937599	16	86527683	A	C	0.39609	-0.436248	0.0162186	5.98E-152	7213
SERPINB8	rs55700832	18	63959273	A	G	0.203175	-0.231685	0.0205677	3.44E-29	7213
SERPINB8	rs1944270	18	63979835	G	A	0.289061	0.131052	0.0183451	9.97E-13	7213
SERPINB8	rs3826616	18	63987229	A	G	0.425828	0.428375	0.0160303	4.32E-150	7213
NT5C3L	rs57672173	17	41837233	C	T	0.238389	0.211134	0.0192345	8.19E-28	7213
CYB5R2	rs11041455	11	7561572	A	C	0.202482	-0.159805	0.0205639	8.86E-15	7213
CYB5R2	rs7942293	11	7656420	G	C	0.362263	-0.535655	0.0161877	1.14E-223	7213
PRPSAP2	rs80251691	17	18859663	C	A	0.0691113	-0.190592	0.0329719	7.76E-09	7213
SERPINE2	rs3187727	2	223875615	A	T	0.0956606	-0.21485	0.0278741	1.45E-14	7213
SERPINE2	rs13412535	2	224010157	G	A	0.234784	-0.92683	0.0162228	0	7213
SERPINE2	rs149016715	2	224299529	C	G	0.120685	0.179058	0.0252024	1.32E-12	7213
USP15	rs11174314	12	62095959	C	T	0.157008	0.158802	0.0227893	3.49E-12	7213
USP15	rs11612349	12	62323777	G	A	0.112921	0.851842	0.0246521	2.99E-242	7213
USP15	rs117307151	12	62598879	T	A	0.0332039	-0.492009	0.0458906	1.28E-26	7213
ZFAND1	rs2912805	8	81689005	A	G	0.14661	0.72832	0.0217759	3.92E-228	7213
ZFAND1	rs7842738	8	81741600	G	C	0.452378	-0.164104	0.0166456	8.75E-23	7213
STAMBP	rs148932286	2	73829050	C	G	0.0142105	0.406138	0.0692955	4.81E-09	7213
NAP1L4	rs3213614	11	2952510	A	G	0.217455	-0.839386	0.0178412	0	7213
NAP1L4	rs139082408	11	3204413	C	T	0.0135866	-0.515221	0.0713983	5.89E-13	7213
DTD1	rs1555353	20	18525973	G	A	0.365105	-0.741219	0.014808	0	7213
DTD1	rs6081458	20	18998891	G	A	0.0539304	-0.212568	0.0365168	6.10E-09	7213
ACAT1	rs12577790	11	108115500	C	A	0.238736	-0.135155	0.0194316	3.82E-12	7213
ADK	rs12571531	10	73967891	C	G	0.198877	0.123916	0.0207421	2.42E-09	7213
SPINK4	rs1630171	9	33234041	A	G	0.154166	0.80272	0.0212888	3.80E-284	7213
SPINK4	rs11789719	9	33304803	A	C	0.0695966	0.24355	0.0327249	1.10E-13	7213
RAB1A	rs7607039	2	65096216	A	G	0.258076	-0.251404	0.0187385	1.48E-40	7213
ATOX1	rs1549921	5	151758650	A	G	0.425135	-0.194396	0.0166606	3.54E-31	7213
DDT	rs4820571	22	23900786	A	G	0.385762	0.218832	0.0168016	2.40E-38	7213
GLUL	rs61805076	1	182185855	T	C	0.332247	0.11505	0.0177447	9.54E-11	7213
PDAP1	rs28495024	7	99419060	G	C	0.123458	-0.178251	0.0252809	1.94E-12	7213
SRGN	rs2229498	10	69097096	G	A	0.157771	-0.50051	0.0220557	3.53E-110	7213
GMPR	rs9370842	6	15908411	C	T	0.18633	-0.158922	0.0215701	1.93E-13	7213
GMPR	rs909559	6	16071868	C	T	0.198877	0.171294	0.0207044	1.54E-16	7213
GMPR	rs4716053	6	16249603	G	A	0.384722	0.904946	0.0132929	0	7213
GMPR	rs9464899	6	16392861	G	A	0.128865	0.149204	0.0247938	1.85E-09	7213
GCDH	rs2238641	19	12895871	T	C	0.386455	-0.170847	0.016967	1.08E-23	7213

ACADVL	rs446994	17	7213534	C	A	0.414113	0.0969528	0.0168895	9.83E-09	7213
G3BP1	rs2964584	5	151763317	T	C	0.25052	-0.190465	0.0188935	9.63E-24	7213
HAGH	rs143902781	16	1510039	C	T	0.0196867	-0.431259	0.0592326	3.67E-13	7213
HAGH	rs13166	16	1827912	C	G	0.141758	0.561284	0.0231017	2.12E-125	7213
GSR	rs2551710	8	30699094	T	C	0.328851	-0.174943	0.0173837	1.15E-23	7213
TSTD1	rs143989520	1	160977761	G	C	0.0350062	-0.476638	0.0444644	1.31E-26	7213
TSTD1	rs10908821	1	161038745	C	G	0.126369	0.998822	0.0222301	0	7213
RBP1	rs2071387	3	139538761	A	G	0.181547	0.20106	0.0213309	5.64E-21	7213
UROD	rs10127584	1	44721867	G	A	0.028005	-0.463429	0.0498562	1.91E-20	7213
UROD	rs12126314	1	44992009	G	A	0.23423	-0.734801	0.017544	0	7213
VPS26A	rs35773981	10	69185042	T	C	0.225149	-0.487651	0.0191891	1.61E-136	7213
ACPP	rs61793793	3	132338990	G	A	0.0532372	0.225928	0.0371068	1.20E-09	7213
TXNDC12	rs6686632	1	52117939	T	C	0.0736864	-0.640626	0.0312302	6.24E-91	7213
MATN3	rs78806344	2	19789731	G	A	0.0153889	-0.460022	0.0676267	1.11E-11	7213
MATN3	rs6760839	2	20056139	C	T	0.366352	0.646021	0.0155005	0	7213
MATN3	rs116697184	2	20415721	A	G	0.0443643	0.265166	0.0402528	4.79E-11	7213
BCAT2	rs62125920	19	48803019	A	G	0.199778	-0.58207	0.0196472	3.97E-182	7213
DCI	rs55650311	16	2251268	C	T	0.162484	-0.175538	0.0224517	6.11E-15	7213
MDGA2	rs75436557	14	47019389	T	G	0.0408984	-0.271599	0.041544	6.68E-11	7213
MDGA2	rs145891385	14	47374910	A	G	0.0584362	0.758481	0.0346071	3.93E-103	7213
OLFM2	rs62104305	19	9948235	G	A	0.33911	0.840317	0.0146166	0	7213
OLFM2	rs3745598	19	9998503	A	G	0.423125	-0.162476	0.0168432	6.90E-22	7213
DDAH1	rs233071	1	85340322	C	T	0.366768	-0.124186	0.0172744	7.18E-13	7213
VEGFA	rs6921438	6	43957870	G	A	0.478719	-0.216499	0.0166333	2.67E-38	7213
GMPR2	rs34354104	14	24238273	G	A	0.0439484	-0.967493	0.0387651	6.14E-132	7213
UPP1	rs10278152	7	48089060	T	A	0.461666	-0.144709	0.0167251	6.16E-18	7213
PDCD6	rs34511054	5	264041	A	C	0.0542077	-0.209217	0.0370949	1.76E-08	7213
PDCD6	rs56075848	5	308237	T	C	0.0649522	0.234281	0.0336805	3.81E-12	7213
NPL	rs116699595	1	182951103	A	G	0.0359074	0.3465	0.0440477	4.18E-15	7213
DTYMK	rs145825209	2	241675032	G	A	0.0167753	-0.438323	0.0648916	1.54E-11	7213
DTYMK	rs145739634	2	241692133	C	T	0.0237765	-0.469782	0.054205	5.43E-18	7213
DTYMK	rs142497237	2	241721906	C	T	0.0431859	0.595972	0.0398942	1.02E-49	7213
HK3	rs61749653	5	176889558	C	T	0.0216276	-0.521669	0.0571537	8.95E-20	7213
PARK7	rs17523802	1	7961680	G	A	0.168723	0.79023	0.0198562	2.71002999999797e-313	7213
PARK7	rs9434612	1	8128395	C	A	0.0428393	-0.239838	0.0410266	5.26E-09	7213
PARK7	rs61774167	1	8129546	A	G	0.0614169	-0.280513	0.0343543	3.75E-16	7213
CR1	rs76124527	1	207494204	A	G	0.0372245	-0.449741	0.043227	3.57E-25	7213
CR1	rs679515	1	207577223	T	C	0.18224	0.618221	0.0202172	3.32E-193	7213
CR1	rs75625374	1	207866086	G	C	0.0605851	-0.242062	0.035186	6.51E-12	7213
KLB	rs7678241	4	39421498	C	T	0.260086	0.184815	0.0188768	1.71E-22	7213
KLB	rs13103023	4	39455997	G	A	0.334119	-0.635276	0.0157342	1.59089137960881e-321	7213
KLB	rs16995354	4	39522684	T	G	0.09878	0.19833	0.0277221	9.24E-13	7213
KLB	rs149922670	4	39651901	T	C	0.0229447	-0.444112	0.0556608	1.71E-15	7213

LRP4	rs2306029	11	46871557	T	C	0.450229	0.459609	0.016059	9.97E-171	7213
PLXNA4	rs62622406	7	132198552	C	T	0.0165673	-0.662224	0.0647393	2.15E-24	7213
PLXNA4	rs744767	7	132330527	T	C	0.236171	-0.124272	0.0195234	2.07E-10	7213
PLXND1	rs146167147	3	129451369	C	T	0.0125468	-0.679734	0.0744112	8.37E-20	7213
PLXND1	rs3774769	3	129493428	G	A	0.0545543	-0.265485	0.0360561	2.00E-13	7213
PLXND1	rs1108584	3	129601458	G	A	0.105365	-0.554971	0.026273	3.80E-96	7213
PLXND1	rs142846687	3	129854591	G	A	0.0268959	0.35	0.0514548	1.11E-11	7213
SEZ6L	rs7284984	22	25975245	A	C	0.111535	0.200005	0.0261671	2.39E-14	7213
SEZ6L	rs76688646	22	26268623	G	A	0.124151	-0.137197	0.0249713	4.06E-08	7213
SEZ6L	rs590671	22	26355557	C	G	0.410509	0.188905	0.0167671	3.37E-29	7213
SEZ6L	rs738399	22	26369587	A	G	0.37682	-0.166168	0.0169604	1.60E-22	7213
DOK2	rs17615788	8	21957228	C	T	0.429364	0.203088	0.0166541	7.16E-34	7213
DOK2	rs12541385	8	22031395	C	T	0.161445	-0.163966	0.0226696	5.22E-13	7213
SFTPD	rs17881517	10	79559768	A	C	0.0209344	-0.412712	0.0575797	8.39E-13	7213
SFTPD	rs528453694	10	79848460	C	T	0.0828365	0.238535	0.0299172	1.79E-15	7213
SFTPD	rs4515911	10	79994752	T	C	0.344586	0.521653	0.0162301	6.03E-212	7213
JUND	rs12610373	19	18287474	A	T	0.362956	0.167005	0.0173267	7.44E-22	7213
TCN1	rs117785973	11	59715368	G	A	0.0183003	0.357165	0.0618206	7.90E-09	7213
TCN1	rs34324219	11	59855905	C	A	0.106405	-0.571434	0.0262174	4.71E-102	7213
TCN1	rs146080691	11	60177545	G	A	0.0122002	-0.517979	0.0751773	6.05E-12	7213
NT5C3A	rs12155117	7	33016438	A	G	0.299459	0.185298	0.0178274	3.97E-25	7213
PTGR1	rs56292970	9	111251468	G	A	0.0155968	-0.458317	0.066585	6.35E-12	7213
PTGR1	rs144875739	9	111431329	G	A	0.0201719	-1.43718	0.055463	1.25E-141	7213
PTGR1	rs73533566	9	111504656	G	A	0.0305698	0.291004	0.0482024	1.65E-09	7213
PTGR1	rs186668635	9	111536358	A	C	0.0189935	-0.488295	0.0606186	9.22E-16	7213
PTGR1	rs7036102	9	111564267	A	G	0.0456121	1.12077	0.0373832	3.25E-186	7213
PTGR1	rs112344479	9	111616359	A	G	0.0451962	-0.511393	0.0393403	3.31E-38	7213
INHBA	rs1122291	7	41708910	C	T	0.243796	-0.112634	0.0193977	6.65E-09	7213
KNG1	rs5030062	3	186736391	A	C	0.37682	0.193292	0.0168854	4.41E-30	7213
F11	rs142316601	4	186148420	G	T	0.0188548	-0.336241	0.0614347	4.57E-08	7213
F11	rs2289252	4	186286227	C	T	0.410786	0.418577	0.0162834	1.66E-139	7213
F11	rs62350309	4	186356512	A	G	0.0542077	-0.234546	0.0370224	2.51E-10	7213
COL18A1	rs9983783	21	45481089	C	T	0.197075	-0.302867	0.0206956	8.18E-48	7213
COL18A1	rs17004785	21	45512704	G	C	0.102315	-0.261808	0.0273872	1.59E-21	7213
PLAT	rs77346091	8	42162640	T	C	0.0214197	-0.52766	0.0568372	2.13E-20	7213
TIMP2	rs11077399	17	78868093	A	G	0.295647	-0.128605	0.0181444	1.49E-12	7213
C5	rs1035029	9	120980540	G	A	0.398586	-0.227462	0.0167937	2.72E-41	7213
CXCL16	rs7214635	17	4697725	G	A	0.249411	-0.214211	0.0190846	5.39E-29	7213
CXCL16	rs9890937	17	4757213	G	A	0.361708	-0.11242	0.0173821	1.06E-10	7213
APCS	rs12727188	1	159516538	T	G	0.0876889	0.370305	0.0290142	6.61E-37	7213
APCS	rs77672383	1	159608353	C	T	0.0237765	-0.919614	0.0533996	3.65E-65	7213
TIMP3	rs10854627	22	32707932	A	G	0.0928878	-0.176551	0.0287254	8.36E-10	7213
TIMP3	rs5749506	22	32771237	C	T	0.267988	0.667918	0.0169097	4.52315e-309	7213

TIMP3	rs137490	22	32868600	C	A	0.2291	-0.198711	0.0198235	1.70E-23	7213
GFRA2	rs11781875	8	21506787	C	T	0.131568	0.171482	0.0246008	3.44E-12	7213
GFRA2	rs15881	8	21693256	A	C	0.460557	0.359501	0.0163353	6.06E-104	7213
GFRA2	rs150868293	8	21826786	G	A	0.177943	-0.285074	0.021666	4.33E-39	7213
GFRA2	rs192923146	8	21897507	G	T	0.0167753	-0.397662	0.0649279	9.56E-10	7213
CCL21	rs10972201	9	34707376	G	A	0.338625	-0.121072	0.0174076	3.83E-12	7213
CFI	rs13117504	4	109737700	C	G	0.417787	0.386073	0.0162638	5.31E-120	7213
IGFBP3	rs148562589	7	45956826	T	C	0.0159434	1.12319	0.064779	5.16E-66	7213
IGFBP3	rs1723959	7	45980023	C	T	0.154582	-0.141766	0.0231808	1.01E-09	7213
MMP9	rs8113877	20	46006406	G	T	0.396229	0.276869	0.0166904	1.11E-60	7213
MPO	rs75394768	17	58336274	C	A	0.0628726	-0.523014	0.033472	3.75E-54	7213
MPO	rs117981636	17	58737704	G	C	0.0370165	0.244041	0.043899	2.81E-08	7213
ROR1	rs6691240	1	63704032	G	A	0.206641	0.121025	0.0207302	5.51E-09	7213
ROR1	rs67884112	1	63828984	C	A	0.159226	0.174764	0.0228713	2.43E-14	7213
ROR1	rs1408416	1	64148812	G	T	0.17205	-0.653146	0.0206531	1.13E-205	7213
ROR1	rs825186	1	64235821	T	C	0.380078	-0.130853	0.017074	2.04E-14	7213
ANGPT2	rs1968586	8	6421990	C	T	0.365243	-0.218101	0.017176	1.49E-36	7213
CST3	rs73610709	20	23561222	C	G	0.482809	0.111426	0.0165387	1.74E-11	7213
CST3	rs2405367	20	23642243	G	A	0.203799	-0.612633	0.0194452	3.10E-204	7213
TYRO3	rs2588323	15	41549880	C	G	0.305559	0.25169	0.0179676	5.23E-44	7213
EFNA4	rs3806256	1	155063135	C	T	0.488008	0.101451	0.0165766	9.84E-10	7213
EFNA5	rs2484103	5	107560916	T	C	0.279565	-0.130069	0.0184064	1.74E-12	7213
EFNA5	rs975690	5	107596105	C	G	0.476847	-0.281576	0.0163759	5.69E-65	7213
ERBB3	rs2292238	12	56100038	A	C	0.412172	-0.19803	0.0165449	1.05E-32	7213
IL6ST	rs13183319	5	55912729	G	A	0.257175	-0.107655	0.0188253	1.12E-08	7213
IL6ST	rs13183065	5	55977599	G	A	0.119437	0.654538	0.0245699	3.24E-149	7213
IL10RB	rs8178462	21	33273129	C	G	0.0203799	-0.406995	0.0587156	4.52E-12	7213
IL10RB	rs2515717	21	33289977	G	A	0.448634	0.233191	0.0164944	8.80E-45	7213
IL12RB1	rs375947	19	18069641	A	G	0.317482	-0.2348	0.0175694	2.94E-40	7213
LAYN	rs4938792	11	111550211	T	C	0.391515	-0.249856	0.0166524	3.92E-50	7213
MRC1	rs11595574	10	17428163	G	A	0.222862	0.112574	0.020016	1.93E-08	7213
MRC1	rs147043080	10	17803476	C	T	0.227783	0.250845	0.0196998	9.57E-37	7213
MRC1	rs111957681	10	17849558	G	A	0.20775	0.704389	0.0188071	1.08E-280	7213
MRC1	rs200928899	10	17887539	T	C	0.24539	-0.169964	0.0192061	1.09E-18	7213
PRKCA	rs61762372	17	66302564	G	A	0.399002	0.305938	0.0167667	9.35E-73	7213
GDI2	rs7091234	10	5658432	A	C	0.335297	0.122863	0.0175831	3.05E-12	7213
GDI2	rs55913768	10	5707486	G	A	0.326078	-0.471464	0.0167435	1.35E-165	7213
GDI2	rs6602290	10	5855172	A	G	0.173506	0.173806	0.0219625	2.87E-15	7213
PLAUR	rs2302524	19	43652320	T	C	0.162484	-0.415355	0.0216989	1.05E-79	7213
PLAUR	rs36229204	19	43671830	C	T	0.0387495	-0.473289	0.0428358	3.73E-28	7213
TNFRSF1A	rs1800693	12	6330843	T	C	0.404963	-0.191908	0.0166692	2.10E-30	7213
NTRK3	rs28714295	15	88027853	G	A	0.128795	0.450297	0.0243349	1.01E-74	7213
NTRK3	rs1948066	15	88047262	C	T	0.308055	-0.174399	0.0180826	7.02E-22	7213

TNFRSF17	rs387871	16	11958626	G	A	0.494039	-0.167918	0.0164414	2.53E-24	7213
DCN	rs3138190	12	91171750	T	G	0.0655067	-0.227633	0.0337274	1.60E-11	7213
EGFR	rs75059484	7	54868647	C	T	0.141619	-0.251438	0.0237542	5.42E-26	7213
EGFR	rs73418703	7	54990815	C	A	0.0103286	0.660676	0.082398	1.24E-15	7213
EGFR	rs12718945	7	55125270	T	G	0.449605	0.171695	0.0166156	7.42E-25	7213
HGF	rs5745695	7	81728759	G	A	0.240191	-0.240622	0.0193617	4.22E-35	7213
C3	rs11569479	19	6696783	T	C	0.140025	-0.155755	0.0238405	6.88E-11	7213
MIA	rs2233154	19	40775441	C	T	0.0707057	1.36981	0.0282651	0	7213
MIA	rs2644899	19	40797044	G	T	0.284348	0.266076	0.0182952	2.98E-47	7213
PLA2G2A	rs12117083	1	19921937	G	A	0.0503258	-0.302963	0.0376485	9.83E-16	7213
PLA2G2A	rs2307246	1	19978364	G	A	0.233953	0.791119	0.0173656	0	7213
PF4	rs3756074	4	73982348	G	C	0.0461666	-0.455359	0.0395815	2.31E-30	7213
PROS1	rs9826711	3	93917625	G	C	0.0251629	-0.439497	0.05283	1.05E-16	7213
PROS1	rs7615840	3	93948926	A	G	0.108346	0.148924	0.026698	2.52E-08	7213
CCL25	rs8112595	19	8027142	G	A	0.425135	-0.296414	0.0165047	1.38E-70	7213
CCL25	rs74959615	19	8056212	G	A	0.0751421	-1.17087	0.0279835	0	7213
POR	rs59882870	7	76009103	G	A	0.156246	0.172892	0.022633	2.47E-14	7213
NOV	rs17793097	8	119453024	A	G	0.102038	0.270322	0.0273796	7.60E-23	7213
SIGLEC6	rs4802807	19	51527194	A	G	0.407528	0.603958	0.0153691	3.20E-306	7213
SIGLEC6	rs34342096	19	51544719	A	G	0.0121309	-0.496308	0.0758411	6.40E-11	7213
SIGLEC7	rs1039405	19	51122980	A	G	0.42569	0.193705	0.016613	3.89E-31	7213
SIGLEC7	rs140185670	19	51142883	G	C	0.0682102	-0.812856	0.0315832	7.97E-140	7213
SHH	rs872723	7	155813050	C	T	0.156384	-0.193114	0.0227008	2.15E-17	7213
IGHG1	rs11621145	14	105706543	G	A	0.328435	-0.178848	0.0177985	1.33E-23	7213
ULBP3	rs72501730	6	150053632	G	A	0.119645	-0.255101	0.0253913	1.36E-23	7213
C3	rs11569479	19	6696783	T	C	0.140025	-0.162316	0.0238344	1.05E-11	7213
IL16	rs17875492	15	81294745	A	C	0.114862	0.206585	0.0258181	1.42E-15	7213
IL16	rs11857713	15	81298924	C	T	0.070775	-1.3708	0.0285247	0	7213
IL16	rs12905152	15	81363127	C	T	0.168862	-0.160866	0.0221789	4.50E-13	7213
CCL3L1	rs854683	17	35986971	G	C	0.204353	0.116574	0.0206527	1.72E-08	7213
CCL3L1	rs1102934	17	36062554	A	G	0.140233	0.464274	0.0233955	2.26E-85	7213
MMP7	rs11568819	11	102530902	G	A	0.0620408	0.795426	0.0329473	6.69E-124	7213
MMP7	rs12796179	11	102559478	T	C	0.235616	-0.241078	0.019652	2.97E-34	7213
ANGPT1	rs1461993	8	107072760	G	A	0.359074	0.138851	0.0170883	5.20E-16	7213
AGRP	rs114322795	16	67476655	C	T	0.03972	0.663465	0.0423294	1.80E-54	7213
AGRP	rs74900413	16	67709521	G	A	0.0129627	-0.456826	0.0738821	6.63E-10	7213
BCAM	rs12461127	19	44549282	C	T	0.473659	0.112504	0.0166522	1.53E-11	7213
BCAM	rs28399656	19	44813447	T	A	0.011507	1.51946	0.0764403	1.21E-85	7213
CDH5	rs6499080	16	66397516	T	C	0.318383	-0.221214	0.0175812	6.28E-36	7213
CX3CL1	rs62037115	16	57424884	G	A	0.0103979	1.11873	0.0814352	2.06E-42	7213
SPINT1	rs17658212	15	40853721	C	T	0.0620408	-0.480648	0.0337008	1.56E-45	7213
KLK11	rs62115743	19	51011618	C	T	0.0824206	-1.05334	0.027856	1.28E-285	7213
KLK8	rs10410942	19	51000397	T	C	0.0664772	-0.796823	0.0320261	3.64E-131	7213

MET	rs40239	7	116677823	G	A	0.155483	0.133153	0.0226124	4.07E-09	7213
MET	rs41748	7	116806519	T	G	0.444267	-0.195354	0.016611	1.21E-31	7213
SPINT2	rs1821284	19	38292941	C	T	0.298766	-0.712418	0.0162571	0	7213
SPINT2	rs12151131	19	38365341	G	A	0.165396	0.27166	0.0221216	2.54E-34	7213
SPINT2	rs78341134	19	38515208	A	G	0.0330653	-0.30377	0.0460043	4.31E-11	7213
TIE1	rs1556580	1	43304250	T	C	0.386178	-0.255506	0.0166141	1.54E-52	7213
C5	rs17220750	9	121025721	G	A	0.0991266	0.298043	0.0276522	6.98E-27	7213
MAPK3	rs9932416	16	29671173	G	T	0.364134	-0.116747	0.0171228	9.96E-12	7213
MAPK3	rs9932466	16	30130526	C	T	0.338763	0.6286	0.0160309	5.00E-305	7213
MAP2K1	rs77428206	15	66484142	C	A	0.0954527	0.179974	0.0284267	2.58E-10	7213
RAC1	rs4724795	7	6338205	G	A	0.290032	0.151851	0.0182425	1.01E-16	7213
UBE2I	rs2401949	16	1296685	A	G	0.275683	-0.261959	0.0184711	4.75E-45	7213
SERPINA3	rs56361564	14	94627677	G	C	0.189727	-0.249953	0.0211581	6.53E-32	7213
C7	rs35867196	5	40798506	C	A	0.0256481	-0.290645	0.0525071	3.22E-08	7213
C7	rs74480769	5	40972109	A	G	0.0340358	-1.50019	0.0423047	4.77E-254	7213
C7	rs79534924	5	40976370	T	G	0.0290448	0.355954	0.049647	8.26E-13	7213
C7	rs112889316	5	40990099	C	T	0.0151116	0.4697	0.0672685	3.16E-12	7213
C7	rs433224	5	41267623	C	T	0.336199	0.40825	0.0168911	3.65E-124	7213
CCL28	rs7710570	5	43378715	T	C	0.397061	0.108661	0.0168285	1.14E-10	7213
CCL14	rs62078063	17	35959448	T	C	0.024539	0.537152	0.0533261	1.04E-23	7213
CCL14	rs7222922	17	36008654	C	T	0.110218	-1.0676	0.0236482	0	7213
MDK	rs61882743	11	46527204	C	G	0.176626	-0.149262	0.0218198	8.53E-12	7213
CCL23	rs7225405	17	35813995	G	T	0.0284902	-0.673694	0.0495092	1.18E-41	7213
CCL23	rs117639761	17	35868176	A	C	0.0159434	0.421391	0.065641	1.45E-10	7213
CCL23	rs712048	17	35999179	C	A	0.129835	-0.563536	0.0238212	3.18E-119	7213
CCL23	rs555642576	17	36059368	C	T	0.0110911	0.562764	0.0796559	1.76E-12	7213
SERPINE1	rs2227631	7	101126257	A	G	0.410439	-0.185799	0.0167899	3.09E-28	7213
APOE	rs35136575	19	44935906	C	G	0.232843	-0.134428	0.0195404	6.51E-12	7213
NBL1	rs12091047	1	19445431	C	T	0.344933	0.136891	0.0174814	5.55E-15	7213
NBL1	rs3020595	1	19644233	C	G	0.244212	-0.223588	0.0190372	1.45E-31	7213
CFD	rs72984031	19	872089	A	G	0.252946	-0.196222	0.0187693	2.12E-25	7213
GHR	rs4866942	5	42641860	T	C	0.463053	0.338674	0.0162371	7.29E-94	7213
GHR	rs62372091	5	42866517	T	C	0.0971856	-0.180791	0.0279571	1.07E-10	7213
PROC	rs1799809	2	127418299	G	A	0.430473	-0.231169	0.0167043	5.25E-43	7213
CLEC11A	rs193222348	19	50720044	C	T	0.0277277	1.25656	0.0487011	1.73E-140	7213
VCAM1	rs141294855	1	100681309	T	C	0.216207	-0.128724	0.0201487	1.78E-10	7213
TNFSF15	rs56339337	9	114798680	A	G	0.145155	-0.157907	0.0234517	1.79E-11	7213
BMP7	rs6123685	20	57260984	G	A	0.25454	0.111039	0.0191018	6.40E-09	7213
CNTN1	rs10878949	12	40643054	A	C	0.163871	0.163644	0.0226332	5.32E-13	7213
CNTN1	rs11177604	12	40651583	C	A	0.386732	0.289771	0.016737	7.91E-66	7213
CNTN1	rs812152	12	41112051	G	C	0.0973936	-0.217555	0.0279005	7.20E-15	7213
EDAR	rs260696	2	108972629	C	T	0.224248	-0.35469	0.0194084	5.69E-73	7213
EDAR	rs115793269	2	108986608	C	T	0.0126854	1.5456	0.0713484	7.19E-101	7213

LGALS4	rs111262200	19	38537190	G	A	0.0296687	0.464442	0.0487952	2.35E-21	7213
LGALS4	rs9304578	19	38759697	G	A	0.471995	-0.273497	0.0161829	7.17E-63	7213
CXCL1	rs74451052	4	73720891	A	G	0.0695966	-0.240792	0.0322064	8.53E-14	7213
CXCL1	rs1366946	4	73872752	T	C	0.215652	0.626211	0.0188078	3.08E-226	7213
CXCL1	rs114862057	4	74102925	C	T	0.0214197	0.385422	0.0562594	7.95E-12	7213
IL1R1	rs2287047	2	102157594	G	A	0.299875	-0.230468	0.0179302	2.11E-37	7213
IL17RA	rs3827278	22	17115025	C	A	0.229724	0.848015	0.0171434	0	7213
IL17RA	rs5748923	22	17166972	T	G	0.331346	-0.210818	0.0174643	3.12E-33	7213
IL17RA	rs3788277	22	17198659	C	G	0.0398586	-0.299611	0.0422852	1.52E-12	7213
IL18RAP	rs4851010	2	102439667	A	T	0.295577	0.290845	0.0180882	3.49E-57	7213
IL1RL2	rs1960510	2	102205805	T	C	0.399626	0.332749	0.0163469	1.32E-89	7213
JAM3	rs655627	11	134151964	G	A	0.446832	-0.215657	0.016636	5.24E-38	7213
LSAMP	rs1835674	3	116319644	T	C	0.176071	-0.348407	0.0214725	3.53E-58	7213
MBL2	rs12573118	10	52743332	G	A	0.423679	-0.657848	0.0148396	0	7213
MBL2	rs2204344	10	52912424	C	T	0.161167	-0.201501	0.0226479	7.15E-19	7213
PDCD1LG2	rs16923189	9	5510644	A	G	0.297102	0.676326	0.0162134	0	7213
PDCD1LG2	rs7854413	9	5557708	T	C	0.0930958	-0.356581	0.0282791	4.50E-36	7213
PDCD1LG2	rs6477000	9	5761493	T	G	0.36968	0.134197	0.0171445	5.69E-15	7213
SIGLEC9	rs73051038	19	51083701	G	C	0.0620408	-0.193461	0.0344215	1.98E-08	7213
SIGLEC9	rs2691249	19	51104817	A	C	0.477125	0.71832	0.0145057	0	7213
SIGLEC9	rs2280806	19	51129600	A	G	0.247816	0.126085	0.0191794	5.24E-11	7213
TGFBR3	rs75735322	1	91587247	C	T	0.0196867	-0.362197	0.0597227	1.39E-09	7213
TGFBR3	rs79859654	1	91620183	A	G	0.214405	0.189129	0.0201309	7.52E-21	7213
TGFBR3	rs2799526	1	91854020	G	A	0.0784001	-0.23211	0.030533	3.29E-14	7213
SERPINF2	rs11078596	17	1714968	C	T	0.183211	-0.349685	0.0212753	1.27E-59	7213
FGF2	rs308403	4	122836593	C	T	0.327672	-0.221641	0.0175047	2.34E-36	7213
CCL23	rs7225405	17	35813995	G	T	0.0284902	-0.736889	0.0493842	1.31E-49	7213
CCL23	rs117639761	17	35868176	A	C	0.0159434	0.427365	0.0656357	7.96E-11	7213
CCL23	rs712048	17	35999179	C	A	0.129835	-0.891227	0.0223906	2.22082000000029e-313	7213
CD209	rs2335525	19	7720827	A	G	0.250659	-0.399727	0.0185423	6.09E-100	7213
CD209	rs4804804	19	7748977	G	A	0.357826	-0.178118	0.0170688	2.59E-25	7213
LGALS2	rs2235338	22	37569873	G	A	0.412796	0.290398	0.0165779	2.59E-67	7213
LGALS2	rs5995489	22	37636908	A	G	0.432622	-0.091607	0.0166026	3.55E-08	7213
CXCL11	rs6827617	4	75994993	G	A	0.390683	0.192949	0.0169386	8.32E-30	7213
CCL3	rs62078244	17	35934014	G	A	0.022806	0.417909	0.0554984	5.69E-14	7213
CCL3	rs2015086	17	36064257	A	G	0.138639	0.609656	0.0230465	2.97E-147	7213
MRC2	rs145495890	17	62499184	C	T	0.0431859	0.330333	0.040253	2.68E-16	7213
MRC2	rs146385050	17	62559897	C	A	0.196382	-0.414284	0.0205227	3.39E-88	7213
MRC2	rs2460290	17	62691485	T	C	0.0156662	0.732544	0.0670185	1.36E-27	7213
SPARC	rs13182103	5	151672199	A	G	0.380563	-0.105409	0.0170322	6.39E-10	7213
SPARC	rs59311424	5	152030389	G	A	0.0311244	-0.386049	0.0477169	6.92E-16	7213
CCL18	rs854682	17	35987298	T	A	0.204353	0.172849	0.0206069	5.90E-17	7213
CCL18	rs2015086	17	36064257	A	G	0.138639	0.855831	0.0219342	2.60E-302	7213

PTN	rs322329	7	137333710	C	G	0.420421	0.293789	0.0165828	8.83E-69	7213
PTN	rs2882591	7	137413339	T	C	0.348884	0.109415	0.0174599	3.90E-10	7213
RETN	rs117761837	19	7643172	C	T	0.014973	-0.464557	0.0685231	1.30E-11	7213
RETN	rs34124816	19	7668790	A	C	0.0286289	-0.748559	0.0493745	3.92E-51	7213
PRSS1	rs56092812	7	142901703	C	T	0.0786081	0.193832	0.0310423	4.50E-10	7213
HP	rs112791888	16	71828759	C	G	0.128449	-0.286696	0.0247578	9.72E-31	7213
HP	rs77303550	16	72045758	C	T	0.190836	0.680945	0.019634	6.77E-244	7213
HP	rs212177	16	72544485	C	A	0.0173298	-0.351813	0.0631802	2.66E-08	7213
TNFSF13B	rs374039502	13	108308037	T	A	0.0250936	0.345389	0.0526957	5.97E-11	7213
C9	rs835210	5	39350369	T	C	0.0331346	0.408037	0.0458495	7.00E-19	7213
C9	rs696766	5	39356024	A	G	0.0549009	0.463091	0.0360227	2.06E-37	7213
LGALS3	rs75464260	14	55049687	C	T	0.0111604	-0.497036	0.0794728	4.22E-10	7213
LGALS3	rs118083722	14	55129494	G	A	0.0865105	-0.80857	0.027847	2.09E-175	7213
IL18BP	rs17884883	11	72097010	T	C	0.401983	0.10036	0.0167364	2.11E-09	7213
LBP	rs2232587	20	38354467	T	C	0.0105365	1.40893	0.0802642	1.40E-67	7213
LBP	rs6025169	20	38361813	G	A	0.0559407	0.456615	0.0357518	5.91E-37	7213
LBP	rs2232613	20	38369011	C	T	0.0797172	-1.21273	0.0271417	0	7213
LBP	rs150091761	20	38385086	C	A	0.0219742	0.328963	0.0563718	5.59E-09	7213
F10	rs547138	13	113137856	T	A	0.396229	-0.2812	0.0166703	1.21E-62	7213
F10	rs2480948	13	113163223	T	C	0.18737	-0.222957	0.0210461	4.95E-26	7213
RARRES2	rs3735167	7	150342466	C	T	0.262928	0.50049	0.0181808	1.02E-158	7213
VEGFC	rs10001755	4	176811946	A	T	0.0190628	-0.350216	0.0606466	8.03E-09	7213
VEGFC	rs112782083	4	176834019	G	C	0.0110911	1.07707	0.0789183	6.86E-42	7213
CD4	rs73053728	12	6787380	A	G	0.305698	-0.148931	0.0178135	7.42E-17	7213
CXCL2	rs1893319	4	74107412	C	T	0.370927	0.113199	0.0172653	5.89E-11	7213
IL2RA	rs12722497	10	6053965	C	A	0.0954527	0.228191	0.028402	1.09E-15	7213
TNFRSF1B	rs5745987	1	12180665	T	G	0.0202412	0.388178	0.0581111	2.57E-11	7213
TNFRSF1B	rs519064	1	12182039	C	T	0.0334812	-0.746031	0.0452391	5.30E-60	7213
CD33	rs60650416	19	51175335	A	G	0.0528906	0.406247	0.0370484	9.29E-28	7213
CD33	rs7245846	19	51227920	G	A	0.357064	-0.839747	0.014136	0	7213
CD33	rs273630	19	51307999	G	A	0.104464	0.157601	0.0268477	4.55E-09	7213
CD33	rs558625031	19	51355657	G	A	0.0260641	-0.370659	0.0519057	1.02E-12	7213
ADAMTS5	rs80176691	21	26852957	T	C	0.0190628	0.4799	0.0605234	2.54E-15	7213
ADAMTS5	rs2830585	21	26932893	C	T	0.160959	-0.912609	0.0198583	0	7213
ADAMTS5	rs2027708	21	27105407	G	A	0.0895605	0.179739	0.0292709	8.66E-10	7213
IDUA	rs3796622	4	989272	T	C	0.360322	-0.623674	0.0158131	3.03E-308	7213
IDUA	rs74921869	4	1019594	G	A	0.181409	0.257283	0.0211406	9.60E-34	7213
IDUA	rs6814865	4	1357369	T	C	0.295023	0.190012	0.0181936	2.37E-25	7213
APP	rs455047	21	26151670	G	C	0.441772	0.16472	0.0166338	5.68E-23	7213
ARSB	rs11743362	5	78880898	G	A	0.436434	-0.262654	0.0165591	1.01E-55	7213
ARSB	rs6877621	5	79018556	G	A	0.286913	0.154734	0.0182548	2.79E-17	7213
NAAA	rs72864159	4	75917068	T	C	0.065576	0.810981	0.0325646	2.13E-131	7213
NAAA	rs28818603	4	76050753	G	T	0.255095	-0.745788	0.0169982	0	7213

NAAA	rs6532181	4	76093596	A	G	0.447317	0.148206	0.0165206	3.69E-19	7213
ADAMTS13	rs7047076	9	133249942	A	T	0.0689727	0.306739	0.0325536	5.81E-21	7213
ADAMTS13	rs28647808	9	133440409	C	G	0.0937197	-0.891989	0.0266192	6.29E-229	7213
ADAMTS13	rs4962150	9	133446270	A	G	0.418896	-0.166753	0.0167023	2.54E-23	7213
CTSA	rs191805	20	45880903	T	A	0.367947	0.22453	0.0170547	3.92E-39	7213
CTSS	rs116173394	1	150636953	G	A	0.0535145	0.378381	0.0364972	5.23E-25	7213
CTSS	rs41271951	1	150764744	A	G	0.0745182	-1.09777	0.0287695	2.40E-290	7213
ENTPD1	rs11188480	10	95774019	T	C	0.336406	0.161665	0.0176497	6.67E-20	7213
F7	rs117926700	13	112991459	A	T	0.0189242	-0.650103	0.0612112	3.73E-26	7213
F7	rs117989138	13	113043357	G	A	0.018231	0.557358	0.0617262	2.18E-19	7213
F7	rs112944884	13	113101528	C	T	0.0122002	-0.475444	0.0756505	3.47E-10	7213
F7	rs776905	13	113127628	A	C	0.0992652	-1.18304	0.0241027	0	7213
WFIKKN1	rs55798945	16	619708	T	G	0.275406	0.170856	0.0185579	4.33E-20	7213
GP6	rs8099942	19	55022110	C	G	0.183488	-0.891725	0.0189148	0	7213
GP6	rs73059038	19	55053377	T	C	0.103563	0.593548	0.0263755	1.93E-108	7213
GP6	rs12981737	19	55115264	C	T	0.0279357	-0.300433	0.0503412	2.52E-09	7213
GNLY	rs78809601	2	85561353	A	T	0.031263	0.302702	0.0473778	1.77E-10	7213
GNLY	rs12714148	2	85632712	T	A	0.278802	-0.134068	0.018591	6.10E-13	7213
GNLY	rs12151752	2	85707529	T	C	0.22612	0.758921	0.0179195	0	7213
HAPLN1	rs6886442	5	83677391	C	A	0.249064	-0.172545	0.0191431	2.52E-19	7213
LTA4H	rs2247570	12	96028599	T	C	0.312422	0.154682	0.0179083	7.00E-18	7213
LYVE1	rs114758648	11	10593808	G	A	0.0426314	0.490929	0.0410168	1.05E-32	7213
LYVE1	rs10840449	11	10630492	C	G	0.446693	-0.123578	0.0166577	1.32E-13	7213
LYVE1	rs34873421	11	10648648	G	C	0.0200333	-0.36057	0.0592346	1.21E-09	7213
METAP1	rs6822589	4	98980016	A	G	0.38902	0.122328	0.0169662	6.16E-13	7213
ASAH2	rs36032010	10	49744055	C	G	0.0454735	0.322956	0.0400258	8.26E-16	7213
ASAH2	rs146622748	10	49780556	G	A	0.0293914	-0.786859	0.0479509	1.93E-59	7213
ASAH2	rs552986493	10	50134341	A	G	0.350201	-0.317646	0.0170854	2.13E-75	7213
ASAH2	rs201395210	10	50218730	A	G	0.117288	-0.447907	0.0251726	2.38E-69	7213
ASAH2	rs2842126	10	50258722	A	G	0.207057	-0.639068	0.019043	1.47E-229	7213
NID1	rs2734807	1	236098248	T	C	0.397962	-0.226066	0.0166358	1.50E-41	7213
PIGR	rs6540730	1	206945992	T	C	0.465063	-0.205398	0.0165742	6.49E-35	7213
RET	rs144670775	10	42839564	G	A	0.0372938	0.404297	0.0435894	2.30E-20	7213
RET	rs2506008	10	43084776	C	A	0.254263	-0.509789	0.0182252	1.51E-163	7213
SFRP1	rs62642270	8	41140689	A	G	0.170595	-0.175349	0.0221304	2.66E-15	7213
SFRP1	rs147918758	8	41310043	C	G	0.392139	-0.209407	0.0167765	2.16E-35	7213
ACP5	rs2305799	19	11576536	C	T	0.122279	-0.444933	0.0247267	7.56E-71	7213
ACP5	rs33949590	19	11780106	C	T	0.0893526	0.270819	0.0286283	4.09E-21	7213
CCDC80	rs9870933	3	112653470	A	G	0.372799	0.147132	0.0170478	7.43E-18	7213
WFIKKN2	rs11079936	17	50830473	T	C	0.329683	0.706933	0.0157228	0	7213
ACAN	rs12594617	15	88715387	C	G	0.19756	0.189913	0.0207819	8.10E-20	7213
ACAN	rs34949187	15	88843421	G	A	0.172397	-0.219758	0.0216866	5.68E-24	7213
TGFBI	rs1990199	5	136059854	G	C	0.474421	0.623481	0.0149424	0	7213

TGFBI	rs11748198	5	136240141	C	A	0.108831	0.214848	0.026636	8.44E-16	7213
CD109	rs10943126	6	73767566	A	T	0.459518	0.667105	0.0147141	0	7213
CD109	rs13211001	6	74058516	A	G	0.107722	0.168609	0.0268477	3.58E-10	7213
FCER2	rs62110713	19	7689659	C	T	0.241924	-0.364279	0.0191969	2.14E-78	7213
FCER2	rs150301323	19	7728748	T	A	0.0159434	0.372263	0.0662618	2.00E-08	7213
CD48	rs352684	1	160711985	A	G	0.423679	-0.353888	0.0163453	9.17E-101	7213
CD48	rs35304526	1	160712795	G	A	0.0253015	-0.375044	0.052914	1.49E-12	7213
CD5L	rs12565518	1	157692347	T	C	0.0503951	-0.234067	0.0378037	6.28E-10	7213
CD5L	rs2765501	1	157834858	G	A	0.420144	0.376988	0.0164016	6.98E-113	7213
CNTN2	rs2068666	1	204948452	T	G	0.0828365	0.19569	0.0301527	9.15E-11	7213
CNTN2	rs3753847	1	205045411	T	C	0.155968	0.84936	0.0207613	0	7213
CNTN2	rs7530217	1	205139891	T	A	0.227506	-0.281	0.0195254	2.55E-46	7213
CNTN4	rs12490123	3	2080171	T	C	0.0356301	0.401915	0.0449459	4.78E-19	7213
CNTN4	rs13071423	3	2098694	C	A	0.181201	0.370997	0.0209993	2.05E-68	7213
CNTN4	rs112526457	3	2254885	T	G	0.144877	-0.225988	0.0235043	9.35E-22	7213
CNTN4	rs1178537	3	2334075	A	C	0.0447109	0.227697	0.0406149	2.14E-08	7213
CNTN5	rs7109365	11	99125224	C	T	0.197629	-0.129656	0.0207994	4.81E-10	7213
CNTN5	rs4528296	11	99222947	C	T	0.0718148	0.731417	0.0309376	4.45E-119	7213
CNTN5	rs181469023	11	99300634	C	T	0.0129627	-0.529701	0.0726196	3.32E-13	7213
CST7	rs4462848	20	24922814	C	A	0.226189	-0.561801	0.0186981	5.67E-187	7213
CST7	rs140988383	20	25003777	A	G	0.0206571	0.972099	0.0576144	1.13E-62	7213
DLL4	rs117454282	15	40847504	C	T	0.0200333	0.375018	0.0578036	9.29E-11	7213
FCGR2A	rs4326638	1	161419170	T	C	0.460627	0.251668	0.0165953	3.69E-51	7213
FCGR2A	rs7522794	1	161505035	T	C	0.339318	-0.728459	0.0153798	0	7213
FCGR2A	rs34754216	1	161605748	C	T	0.396576	0.231783	0.0169086	3.07E-42	7213
FCGR2B	rs11799744	1	161428624	T	C	0.0562873	-0.334256	0.0362308	3.63E-20	7213
FCGR2B	rs115264949	1	161532142	G	A	0.0118536	0.779797	0.0759138	1.39E-24	7213
FCGR2B	rs72700095	1	161597399	T	C	0.228546	0.956422	0.0161145	0	7213
FCGR2B	rs16827592	1	161649251	T	G	0.0751421	-0.22874	0.0310796	2.05E-13	7213
FCGR3B	rs10800573	1	161538134	A	G	0.32483	0.462213	0.0169506	6.58E-156	7213
FCGR3B	rs11578979	1	161688868	G	A	0.249896	0.128756	0.0192483	2.41E-11	7213
FCN2	rs57136797	9	134860694	A	T	0.143283	-0.723795	0.0221636	2.75E-218	7213
FCN2	rs11103557	9	134864975	C	A	0.0761819	0.405727	0.0311961	3.05E-38	7213
GFRA1	rs10885876	10	116202766	C	G	0.290864	0.339095	0.0178832	2.77E-78	7213
SERPIND1	rs73160986	22	20752297	T	C	0.11202	0.150383	0.0262143	1.00E-08	7213
IGFBP7	rs881382	4	57051180	G	T	0.278802	0.160197	0.0184523	4.77E-18	7213
IGFBP7	rs1718860	4	57083351	G	A	0.256412	0.529674	0.0181789	1.52E-176	7213
LRIG3	rs140704863	12	58752217	C	A	0.0169139	0.625784	0.0636123	1.08E-22	7213
LRIG3	rs142216336	12	58785207	C	T	0.0251629	0.408782	0.0530151	1.42E-14	7213
LRIG3	rs61096689	12	58873782	T	C	0.0222515	0.363592	0.0554889	6.05E-11	7213
LRIG3	rs11172791	12	58879191	T	C	0.0444337	-0.846134	0.0389619	2.38E-101	7213
LRIG3	rs61921897	12	58898770	A	G	0.0159434	0.419877	0.066518	2.91E-10	7213
LRIG3	rs61936480	12	59282990	G	A	0.0287675	0.611805	0.0495226	1.04E-34	7213

LRP8	rs7542607	1	53270941	A	G	0.418827	0.255027	0.0166331	3.07E-52	7213
LRP8	rs4926974	1	53348560	G	A	0.205393	0.128579	0.0206575	5.11E-10	7213
LY9	rs12128261	1	160792994	T	G	0.21593	0.607541	0.018855	6.22E-213	7213
LY9	rs11577230	1	161083854	C	T	0.115555	-0.163679	0.0260608	3.57E-10	7213
LY9	rs12721035	1	161221549	C	T	0.0576736	-0.208036	0.0355306	4.98E-09	7213
MATN2	rs17831160	8	98033638	G	A	0.0344517	0.566124	0.0446356	1.79E-36	7213
MATN2	rs28522929	8	98052385	C	T	0.22099	0.238337	0.020007	2.04E-32	7213
CADM1	rs17564430	11	115172854	T	G	0.253015	0.292195	0.0187274	5.31E-54	7213
NTN4	rs17288108	12	95738117	A	G	0.176002	-0.372722	0.0213431	6.40E-67	7213
NTN4	rs9919756	12	95786367	T	G	0.469153	-0.126322	0.0166724	3.99E-14	7213
NTN4	rs7486703	12	95944219	A	G	0.435741	0.111731	0.0167571	2.79E-11	7213
PGLYRP1	rs2072563	19	46023390	G	A	0.316235	-0.213745	0.0178008	6.66E-33	7213
RGMB	rs2368553	5	98446049	C	T	0.452655	-0.339456	0.0161518	3.17E-95	7213
RGMB	rs25743	5	98622050	G	A	0.196243	0.164289	0.0210077	6.01E-15	7213
TFPI	rs116011690	2	187459732	C	T	0.0219742	-0.557414	0.0566753	1.10E-22	7213
THBS2	rs9346611	6	169070944	C	T	0.175794	-0.166611	0.0218211	2.54E-14	7213
THBS2	rs1556454	6	169158880	G	A	0.0354915	-0.393212	0.04513	3.63E-18	7213
THBS2	rs74507247	6	169224675	G	A	0.0982947	0.945145	0.0255704	4.93E-274	7213
THBS2	rs10945406	6	169238696	A	C	0.0530293	-0.200198	0.0366803	4.98E-08	7213
THBS4	rs2451934	5	79902130	T	A	0.244143	0.176576	0.0193015	7.40E-20	7213
THBS4	rs2404710	5	79960739	C	A	0.433731	0.211661	0.0165669	5.60E-37	7213
THBS4	rs13167730	5	80074424	G	T	0.0934424	0.654834	0.0277116	5.70E-119	7213
ACY1	rs148804382	3	52078217	C	A	0.0125468	-0.679851	0.0739927	5.14E-20	7213
BMP1	rs1126931	8	22164420	C	T	0.16907	-0.198104	0.0220545	3.33E-19	7213
CA6	rs35370789	1	8563519	C	G	0.0135866	-0.522604	0.0721413	4.80E-13	7213
CA6	rs942969	1	8947497	G	C	0.325038	0.184862	0.017712	2.54E-25	7213
CA6	rs3765963	1	8974539	A	G	0.404478	0.648589	0.0151819	0	7213
CSK	rs34933034	15	74787133	G	A	0.164564	0.489076	0.0215858	7.53E-110	7213
CTSV	rs4743059	9	97137031	C	T	0.0754887	0.390775	0.0311744	1.13E-35	7213
CTSV	rs10981323	9	97241897	C	G	0.446832	-0.18849	0.0165674	9.74E-30	7213
ECM1	rs11806879	1	150131106	C	T	0.0858866	0.260473	0.0294755	1.22E-18	7213
ECM1	rs1260388	1	150399540	T	G	0.400596	-0.832824	0.0137301	0	7213
FETUB	rs74751406	3	186656426	C	T	0.0469292	0.562493	0.0384248	7.68E-48	7213
FETUB	rs184036273	3	186696470	G	A	0.0184389	0.811577	0.061	6.42E-40	7213
FETUB	rs5030103	3	186731139	T	C	0.0138639	-1.40629	0.0693812	6.97E-89	7213
IL17RD	rs6776722	3	57108631	G	A	0.302787	0.624575	0.0164486	8.74E-288	7213
IL17RD	rs768713	3	57124309	G	C	0.380702	-0.123408	0.0173475	1.24E-12	7213
IL17RD	rs4282030	3	57166552	C	A	0.333218	-0.114512	0.0175195	6.74E-11	7213
KLK7	rs1654523	19	50981938	C	T	0.0829752	-0.590329	0.0292149	2.32E-88	7213
KLK7	rs268898	19	50988912	A	G	0.438722	0.0964507	0.0167517	8.88E-09	7213
LYN	rs1050855	8	55880027	G	A	0.490226	-0.146682	0.0166053	1.26E-18	7213
SERPINA5	rs10133793	14	94584813	A	C	0.331	0.155602	0.0175565	9.69E-19	7213
REN	rs113430136	1	204289080	C	T	0.0643976	0.213041	0.0336216	2.49E-10	7213

TPSB2	rs9925427	16	1225896	G	A	0.485651	0.634792	0.0149309	0	7213
TPSB2	rs11641981	16	1263636	T	C	0.339872	0.200345	0.0172321	5.72E-31	7213
TPSB2	rs7190606	16	1434612	G	A	0.0973936	0.216044	0.027858	1.00E-14	7213
UFC1	rs79923112	1	161158854	G	C	0.0126854	0.453104	0.0742539	1.10E-09	7213
IBSP	rs2616262	4	87802058	G	T	0.497643	0.116683	0.0165558	1.98E-12	7213
CAMK2D	rs3822295	4	113748360	A	C	0.124913	-0.140797	0.0252927	2.69E-08	7213
CA13	rs2403082	8	85166967	T	A	0.261056	-0.386942	0.0184143	3.39E-95	7213
CA13	rs113014331	8	85272310	G	T	0.0625953	-1.22592	0.0311956	3.09E-306	7213
TNFSF8	rs3181348	9	114931904	G	A	0.426452	-0.244436	0.0165723	1.57E-48	7213
EPHA1	rs56242860	7	143371794	C	T	0.232289	0.201874	0.0197491	2.32E-24	7213
EPHA1	rs41277428	7	143388729	G	A	0.0772217	-1.26054	0.0274232	0	7213
EPHA1	rs117978799	7	143395749	C	T	0.0108138	-0.450318	0.0807525	2.54E-08	7213
FN1	rs1250258	2	215435462	C	T	0.266602	-0.285448	0.0185882	2.15E-52	7213
FSTL3	rs3787004	19	657666	C	T	0.474768	-0.138794	0.016505	4.93E-17	7213
GZMA	rs4865901	5	54993327	A	G	0.265354	0.111919	0.0187994	2.75E-09	7213
GSK3A	rs61088131	19	42196795	T	C	0.161722	0.124651	0.022779	4.59E-08	7213
IL15RA	rs8177643	10	5974657	C	T	0.317482	0.15904	0.0175913	1.97E-19	7213
INSR	rs3745545	19	7211830	T	C	0.154651	-0.139215	0.0229695	1.42E-09	7213
SERPINA4	rs116862882	14	94500380	T	C	0.0354915	-0.268485	0.044705	2.00E-09	7213
SERPINA4	rs10135681	14	94541407	T	C	0.377513	0.271951	0.0169685	7.89E-57	7213
SERPINA4	rs10147337	14	94559223	A	T	0.210592	0.782931	0.0182044	0	7213
LYN	rs1050855	8	55880027	G	A	0.490226	-0.137026	0.0166168	1.93E-16	7213
POSTN	rs7329947	13	37524954	G	A	0.381603	0.240615	0.0168504	1.23E-45	7213
PDGFRB	rs7709258	5	150108347	T	C	0.363857	-0.196454	0.0170168	1.46E-30	7213
PDGFRB	rs246395	5	150120109	T	C	0.32684	-0.496531	0.0167465	2.12E-182	7213
PDGFRB	rs3828610	5	150156062	A	C	0.367323	-0.649991	0.0156066	0	7213
BCAN	rs2365715	1	156645322	A	G	0.37779	-0.31023	0.0165442	1.22E-76	7213
SELE	rs4987388	1	169689445	A	G	0.279842	0.156461	0.0182664	1.30E-17	7213
XPNPEP1	rs3862006	10	109991006	G	A	0.147927	0.386843	0.0229056	8.69E-63	7213
AGT	rs11568018	1	230715449	G	C	0.105435	0.458801	0.0264426	4.22E-66	7213
AGT	rs1111051	1	230742742	T	C	0.0503951	0.253534	0.037841	2.24E-11	7213
CAT	rs208674	11	34420905	T	C	0.405518	0.19021	0.016655	5.96E-30	7213
CAT	rs4756148	11	34465650	C	T	0.229031	-0.349438	0.0194518	1.26E-70	7213
CXCL6	rs16850073	4	73838282	C	T	0.371621	0.624649	0.0156906	1.78451000000282e-313	7213
CXCL6	rs191180456	4	73960599	C	T	0.0108138	-0.734233	0.0804632	9.15E-20	7213
IL17B	rs6862301	5	149404127	C	A	0.293221	0.189295	0.0181004	2.04E-25	7213
CCL22	rs4784790	16	57316119	G	C	0.0682795	0.341436	0.0325279	1.37E-25	7213
CCL22	rs170364	16	57376022	T	G	0.251144	-0.227769	0.019172	2.99E-32	7213
CXCL12	rs10793518	10	44015012	A	G	0.180785	-0.131641	0.0215109	9.86E-10	7213
CXCL12	rs1023264	10	44398308	C	T	0.325939	0.176212	0.017863	8.26E-23	7213
CPB2	rs2806894	13	45897526	C	T	0.288507	0.165098	0.0182406	1.79E-19	7213
CPB2	rs9316181	13	46071605	G	A	0.328435	0.766275	0.0152554	0	7213
CPB2	rs7323653	13	46128395	G	A	0.268127	0.117807	0.0186375	2.75E-10	7213

CCL17	rs223895	16	57406984	T	C	0.346042	0.179432	0.0174447	1.21E-24	7213
CCL17	rs28695417	16	57410006	G	A	0.0849161	0.510802	0.0292765	8.36E-67	7213
DKK1	rs1863730	10	52332892	T	C	0.45626	-0.0915349	0.016741	4.71E-08	7213
DKK1	rs1159798	10	52652733	A	C	0.232636	-0.402413	0.019055	4.18E-96	7213
DKK1	rs11003047	10	52674751	T	G	0.105435	0.570006	0.0263877	2.51E-100	7213
ADIPOQ	rs73185669	3	186746268	G	C	0.0200333	0.352302	0.0592415	2.86E-09	7213
ADIPOQ	rs76071583	3	186840083	A	G	0.0277277	-0.88664	0.0496828	9.66E-70	7213
ADIPOQ	rs17366653	3	186853027	T	C	0.0125468	-0.835872	0.0741903	3.36E-29	7213
SERPINA1	rs4905172	14	94233801	C	G	0.0439484	0.535752	0.0400434	2.41E-40	7213
SERPINA1	rs55704412	14	94282356	G	A	0.0322335	0.306852	0.0470428	7.37E-11	7213
SERPINA1	rs79247904	14	94309697	G	A	0.027797	0.355845	0.0508004	2.70E-12	7213
SERPINA1	rs143038446	14	94363153	C	T	0.0155275	0.413104	0.0670697	7.70E-10	7213
SERPINA1	rs28929474	14	94378610	C	T	0.0187855	-1.8065	0.0576364	2.77E-202	7213
SERPINA1	rs17580	14	94380925	T	A	0.0402745	-0.86525	0.0410714	1.16E-95	7213
AHSG	rs2518134	3	186614393	C	T	0.357133	-0.517787	0.0163458	2.64E-206	7213
AHSG	rs78048160	3	186656837	A	G	0.0189935	0.37959	0.0605012	3.72E-10	7213
BMP10	rs34008398	2	68866281	G	A	0.00949674	-0.579365	0.085333	1.22E-11	7213
CASP3	rs55880126	4	184670083	T	G	0.124567	-0.728277	0.0238045	2.20E-193	7213
CASP3	rs12644905	4	184755529	C	T	0.0350756	0.318867	0.0448023	1.21E-12	7213
CHIT1	rs11586183	1	203214444	A	T	0.0177457	-0.843316	0.0623302	3.31E-41	7213
CHIT1	rs2486070	1	203226561	G	A	0.179814	-0.919383	0.0189702	0	7213
MASP1	rs3774275	3	187247480	A	G	0.309303	-0.213235	0.0176305	2.37E-33	7213
DKK3	rs10734190	11	11980040	T	C	0.155552	-0.311376	0.0224849	4.62E-43	7213
DKK3	rs11022114	11	12017327	G	A	0.310481	0.47715	0.0170313	4.91E-164	7213
GNS	rs1690278	12	64739864	C	G	0.473659	-0.109823	0.0168402	7.43E-11	7213
HGFAC	rs4690013	4	3434287	C	A	0.397338	0.277502	0.0166425	2.80E-61	7213
HGFAC	rs1203107	4	3458122	C	T	0.10391	-1.09157	0.0244083	0	7213
LGMN	rs148659834	14	92709697	G	A	0.0135866	-0.853805	0.0702144	1.08E-33	7213
LGMN	rs2236264	14	92732735	C	T	0.169278	-0.187174	0.0220514	2.53E-17	7213
LY86	rs977785	6	6588648	A	C	0.261472	-0.187434	0.0188693	4.20E-23	7213
LRPAP1	rs16844464	4	3493229	C	T	0.0309857	-0.979512	0.0465103	1.33E-95	7213
KDR	rs34231037	4	55106779	A	G	0.0307778	-1.11205	0.0465962	3.16E-121	7213
KDR	rs2305948	4	55113391	C	T	0.0990573	0.526536	0.0272717	5.07E-81	7213
KDR	rs138595949	4	55155678	G	A	0.0142798	-0.39294	0.0701716	2.23E-08	7213
KDR	rs78498771	4	55240580	G	A	0.0200333	-0.353798	0.0590314	2.15E-09	7213
PLG	rs58432601	6	160463068	G	A	0.127478	0.15153	0.024914	1.25E-09	7213
PLG	rs11751347	6	160671406	C	T	0.101414	-0.539672	0.0267928	7.89E-88	7213
PLG	rs3823055	6	160717885	A	G	0.290309	-0.146284	0.0182261	1.17E-15	7213
PLG	rs150683617	6	160784251	T	C	0.0264106	1.00581	0.0506899	2.36E-85	7213
PLG	rs62435485	6	161045983	T	A	0.0311937	-0.278562	0.0477724	5.75E-09	7213
PYY	rs12449853	17	44049191	A	C	0.196312	0.138133	0.0208194	3.48E-11	7213
TEK	rs511619	9	27196496	A	C	0.0357688	0.63166	0.0439491	3.33E-46	7213
TEK	rs35030851	9	27197488	G	T	0.0438098	0.859212	0.0395199	1.45E-101	7213

ANGPTL4	rs116843064	19	8364439	G	A	0.0209344	-0.59482	0.0571648	3.53E-25	7213
CA3	rs1543852	8	85440529	A	G	0.387218	-0.288002	0.0168434	2.77E-64	7213
CST5	rs6138152	20	23869493	A	G	0.174199	0.729782	0.019966	1.68E-268	7213
CST5	rs67927430	20	23942700	C	T	0.271038	0.136142	0.0185268	2.23E-13	7213
ESM1	rs4242051	5	54902947	T	C	0.248926	-0.181186	0.0191393	3.83E-21	7213
ESM1	rs1895413	5	54962065	G	T	0.498822	0.101468	0.0165901	1.01E-09	7213
EPHA5	rs2122720	4	65385355	A	C	0.476154	-0.114321	0.0164133	3.57E-12	7213
EPHA5	rs6849874	4	65415013	A	G	0.318383	-0.114263	0.0177109	1.18E-10	7213
EPHA5	rs28663440	4	65595644	G	T	0.0743103	0.403083	0.0313429	1.93E-37	7213
EPHA5	rs13149202	4	66118613	G	A	0.227298	0.114864	0.0199403	8.74E-09	7213
FYN	rs9487731	6	111833645	T	C	0.183627	-0.142789	0.0213955	2.68E-11	7213
MAPKAPK2	rs4256810	1	206717104	T	C	0.317205	0.330034	0.0174927	1.54E-77	7213
PPIA	rs56119332	7	44773770	A	G	0.0725773	-0.387659	0.0319598	1.56E-33	7213
MDH1	rs262505	2	63634652	C	T	0.281714	0.340077	0.0182467	9.37E-76	7213
PRDX1	rs7537361	1	45029523	G	A	0.313392	-0.110403	0.0179464	8.07E-10	7213
PRDX1	rs11211133	1	45525407	G	A	0.232081	0.357692	0.0194818	1.28E-73	7213
ACP1	rs300750	2	208794	T	G	0.496049	-0.63417	0.0147967	0	7213
ACP1	rs7419262	2	273621	C	G	0.311937	-0.582657	0.0165734	4.73E-250	7213
ACP1	rs2203063	2	338364	C	T	0.422778	0.0982209	0.0167877	5.11E-09	7213
SBDS	rs79344818	7	66988489	A	G	0.0356994	-1.18714	0.0427437	2.22E-161	7213
TPT1	rs2234222	13	45339402	G	C	0.0489394	0.274344	0.0377888	4.28E-13	7213
NAGK	rs11680831	2	71065392	T	C	0.349231	-0.513707	0.0163099	3.96E-204	7213
NAGK	rs62143760	2	71472697	C	T	0.313046	-0.101785	0.0179615	1.51E-08	7213
UBE2N	rs2291266	12	93426357	C	A	0.16595	-0.400189	0.0216423	1.27E-74	7213
BPI	rs6098926	20	38275548	A	G	0.115763	-0.200722	0.0256253	5.45E-15	7213
BPI	rs4287822	20	38293108	A	G	0.351449	0.421909	0.0167843	9.78E-134	7213
BPI	rs6127742	20	38322148	G	A	0.100097	-0.749556	0.02612	1.38E-171	7213
BPI	rs220546	20	38617395	C	T	0.129211	-0.164289	0.0246247	2.72E-11	7213
C6	rs77444140	5	41171293	G	A	0.0183696	-0.971168	0.0610293	4.59E-56	7213
C6	rs4957379	5	41206940	T	C	0.0938583	0.510501	0.0277033	3.91E-74	7213
C6	rs138054038	5	41243435	T	C	0.0218356	-0.454247	0.0567865	1.45E-15	7213
NTF3	rs73039984	12	5440290	C	T	0.097047	-0.190756	0.028254	1.58E-11	7213
NTF3	rs61908059	12	5568422	G	A	0.0998198	0.156801	0.0275556	1.32E-08	7213
PLG	rs9355291	6	160568256	C	T	0.163039	-0.321649	0.0221637	4.64E-47	7213
PLG	rs12211977	6	160831738	G	A	0.102523	-0.54473	0.0268489	4.77E-89	7213
KLKB1	rs4253304	4	186252417	G	C	0.420006	0.36051	0.0162797	3.53E-105	7213
SERPINA3	rs10149025	14	94594699	A	G	0.105157	-0.224922	0.026842	6.33E-17	7213
SERPINA3	rs17753556	14	94629097	C	A	0.189172	-0.525621	0.0204451	1.55E-139	7213
SELP	rs6136	1	169594713	T	G	0.10793	-0.643764	0.0255934	6.70E-134	7213
TNC	rs11794797	9	115052994	G	A	0.231249	0.748249	0.0177076	0	7213
TNC	rs112637280	9	115111431	G	A	0.1202	-0.204772	0.0254363	9.58E-16	7213
TNC	rs10982670	9	115320267	A	C	0.419174	-0.137839	0.0166504	1.47E-16	7213
PLAU	rs2633322	10	73934174	T	C	0.260641	0.413473	0.018235	5.21E-110	7213

PLAU	rs138437684	10	74012183	C	T	0.0198253	-0.367793	0.0588911	4.47E-10	7213
CFH	rs145462852	1	196350092	A	C	0.0221822	0.332529	0.0563025	3.66E-09	7213
CFH	rs1048663	1	196705852	G	A	0.162138	-0.601286	0.0212371	2.60E-167	7213
MMP2	rs243837	16	55498847	A	T	0.38902	-0.11658	0.0169817	7.20E-12	7213
TF	rs8177245	3	133760386	A	G	0.335505	0.171696	0.0175016	1.41E-22	7213
YWHAG	rs73140051	7	76335899	G	A	0.280466	-0.118233	0.0184035	1.41E-10	7213
PGD	rs113612161	1	10403531	C	T	0.0198253	-0.947738	0.0590627	5.83E-57	7213
AKR7A2	rs67178903	1	19044833	G	A	0.0532372	0.209896	0.0370171	1.48E-08	7213
AKR7A2	rs74430522	1	19262680	G	A	0.0943435	-0.521227	0.0277181	4.72E-77	7213
AKR7A2	rs10917454	1	19469357	T	C	0.408083	0.199063	0.0169642	1.64E-31	7213
AKR1A1	rs7520156	1	45446590	A	T	0.0607237	-1.3829	0.0307011	0	7213
AKR1A1	rs74849674	1	45489242	C	A	0.0596839	0.229721	0.0353279	8.42E-11	7213
AKR1A1	rs61751012	1	45566635	G	A	0.0110218	-0.958253	0.0788624	1.21E-33	7213
VTA1	rs225628	6	142151799	T	C	0.474283	-0.266426	0.0164987	1.19E-57	7213
VTA1	rs2077836	6	142600924	T	C	0.457091	-0.10284	0.0167984	9.73E-10	7213
FER	rs71592765	5	108755299	C	G	0.0849854	0.367916	0.0292711	7.39E-36	7213
IGF1R	rs62022255	15	98637139	A	C	0.0776376	-0.171795	0.0309943	3.08E-08	7213
IGF1R	rs7162314	15	98946687	G	A	0.206849	0.239139	0.0203011	9.71E-32	7213
IL1RL1	rs2241132	2	102187575	C	A	0.128449	-0.351803	0.0243456	1.11E-46	7213
IL1RL1	rs1420101	2	102341256	C	T	0.370442	-0.651533	0.0152811	0	7213
IL1RL1	rs13011360	2	102614929	C	T	0.0993345	-0.24228	0.0271798	6.15E-19	7213
NME2	rs115848216	17	51166498	G	A	0.0409677	-1.37989	0.0388935	2.84E-254	7213
NME2	rs11650047	17	51304202	A	T	0.387356	0.203729	0.0170057	9.24E-33	7213
NSFL1C	rs4814391	20	1622497	C	T	0.133786	0.1842	0.0243424	4.29E-14	7213
NUDCD3	rs73105364	7	44440865	C	T	0.0354222	0.376946	0.0445447	3.15E-17	7213
PEBP1	rs6490169	12	118131829	C	T	0.0364619	-0.5314	0.0441975	5.53E-33	7213
PEBP1	rs1051077	12	118145103	C	T	0.0612089	0.520718	0.0336393	3.40E-53	7213
P4HB	rs56112103	17	82336294	A	G	0.478026	-0.198497	0.0164492	3.28E-33	7213
NAPA	rs71363738	19	47515000	G	T	0.0347983	0.582813	0.0453537	2.20E-37	7213
SPHK1	rs12941069	17	76464503	T	C	0.344586	-0.152241	0.0172714	1.49E-18	7213
SPON1	rs10832164	11	14026933	C	T	0.493623	0.498155	0.0155695	3.60E-210	7213
SPON1	rs3815984	11	14253979	C	T	0.0598919	0.320595	0.0347411	3.56E-20	7213
TPI1	rs7138740	12	6874600	T	A	0.284972	-0.346424	0.0180141	2.06E-80	7213
CST2	rs6036492	20	23721534	C	A	0.430126	0.128237	0.0166666	1.61E-14	7213
CST2	rs4260306	20	23749699	C	T	0.282961	-0.512363	0.0175648	6.40E-177	7213
BOC	rs3856718	3	113277371	G	A	0.17108	0.355246	0.0215859	9.05E-60	7213
CLEC1B	rs676397	12	9955834	A	G	0.101345	0.401401	0.0271337	8.35E-49	7213
CLEC1B	rs521040	12	9995251	T	C	0.259809	0.426407	0.0183401	2.29E-115	7213
CLEC1B	rs73054650	12	10027242	C	G	0.110703	-0.173408	0.026055	3.03E-11	7213
CRP	rs2794520	1	159709026	C	T	0.325177	-0.273903	0.0174272	9.31E-55	7213
ICAM1	rs2228615	19	10292692	G	A	0.381949	-0.965838	0.0131561	0	7213
ICAM1	rs378395	19	10315746	A	C	0.468044	-0.146177	0.0166264	1.82E-18	7213
DAPK2	rs55986634	15	63983446	G	A	0.427839	0.160119	0.01671	1.28E-21	7213

GDF15	rs1058587	19	18388612	C	G	0.262166	0.672832	0.0169581	1.42281999999996e-311	7213
GDF15	rs74953716	19	18587889	C	T	0.0387495	0.307817	0.0427244	6.41E-13	7213
MST1	rs3197999	3	49684099	G	A	0.284417	-1.04869	0.0137809	0	7213
MST1	rs111744323	3	50183594	C	T	0.0159434	-0.386885	0.0659584	4.67E-09	7213
SLPI	rs6104034	20	45137017	G	C	0.47858	-0.224728	0.0164709	7.23E-42	7213
COLEC11	rs6542680	2	3592552	C	T	0.190905	0.657269	0.0196628	6.79E-228	7213
COLEC11	rs11123637	2	3606600	T	C	0.363233	0.190796	0.0170923	1.07E-28	7213
ENPP7	rs28502318	17	79730489	C	G	0.369472	0.902698	0.0138289	0	7213
ENPP7	rs11658405	17	79824192	C	T	0.0907389	0.187752	0.0289045	8.82E-11	7213
ENTPD5	rs150027599	14	73827792	T	C	0.0169832	0.511823	0.0644327	2.26E-15	7213
ENTPD5	rs73301475	14	73996165	C	T	0.0565645	-1.25585	0.0328625	3.78E-291	7213
ENTPD5	rs118186707	14	74219872	G	A	0.0325801	0.303387	0.0464082	6.69E-11	7213
FCRL3	rs6681271	1	157696854	T	C	0.465618	0.699858	0.0143977	0	7213
FCRL3	rs74121555	1	157846607	C	T	0.0589907	0.24898	0.0351683	1.58E-12	7213
MFGE8	rs8024934	15	88893254	A	C	0.44808	-0.127742	0.0166201	1.72E-14	7213
MFGE8	rs34239095	15	88912537	C	G	0.279703	0.442825	0.0178388	1.33E-130	7213
PCSK7	rs7121898	11	116926130	A	T	0.352489	-0.667038	0.015631	0	7213
PCSK7	rs7934690	11	117140583	T	A	0.293359	0.224294	0.0181824	1.30E-34	7213
SPARCL1	rs1408	4	87136201	G	A	0.42978	-0.134798	0.0168472	1.43E-15	7213
SPARCL1	rs6814797	4	87525976	A	G	0.219673	0.737211	0.0181077	0	7213
SPARCL1	rs62315973	4	87569196	A	G	0.136906	0.27292	0.0240327	1.23E-29	7213
CHST15	rs7078438	10	124109086	C	T	0.303618	-0.193143	0.0179469	8.31E-27	7213
SERPING1	rs142322855	11	57319582	G	A	0.0530293	0.242589	0.0371016	6.64E-11	7213
SERPING1	rs117635824	11	57415425	C	T	0.0153889	-0.447649	0.0670201	2.58E-11	7213
SERPING1	rs11601449	11	57756838	G	A	0.32989	-0.653005	0.0158003	0	7213
C3	rs2230199	19	6718376	G	C	0.201442	-0.143409	0.0205581	3.31E-12	7213
FGF7	rs12439429	15	49442437	C	T	0.460627	-0.183085	0.01652	2.58E-28	7213
FGF7	rs2413977	15	49916375	A	G	0.320671	-0.163605	0.0177057	3.18E-20	7213
MMP12	rs2276109	11	102875061	T	C	0.129696	-0.749039	0.0231256	5.36E-215	7213
MMP12	rs72987587	11	103037030	T	G	0.0564952	0.20854	0.0360184	7.34E-09	7213
NCAM1	rs7951615	11	112768825	G	A	0.484057	-0.224288	0.0164461	7.86E-42	7213
NCAM1	rs11214489	11	113105212	C	T	0.190143	-0.359937	0.0207921	8.17E-66	7213
NCAM1	rs2288158	11	113262954	T	G	0.134063	0.459764	0.0239533	4.02E-80	7213
CLEC11A	rs116924815	19	50727476	C	T	0.028005	1.28774	0.048344	3.48E-149	7213
PRSS22	rs7204669	16	2860735	G	A	0.319354	0.30323	0.0173419	4.42E-67	7213
BST1	rs4279202	4	15573801	C	T	0.127201	-1.138	0.0211708	0	7213
BST1	rs114667404	4	15660215	C	T	0.0246777	0.337121	0.0534135	2.93E-10	7213
BST1	rs16892370	4	15753476	G	A	0.0451269	-0.89664	0.0383992	2.59E-116	7213
BST1	rs7699101	4	15902819	T	A	0.456606	-0.092498	0.0166328	2.78E-08	7213
CDON	rs1047070	11	125957177	G	A	0.197144	0.14037	0.0208975	2.00E-11	7213
CDON	rs112978541	11	126018717	T	C	0.0876196	-1.03696	0.0267429	6.45E-299	7213
CDON	rs562672	11	126113569	C	T	0.4155	-0.155372	0.0167928	2.84E-20	7213
CDON	rs240551	11	126333862	G	A	0.0394427	0.31431	0.0421576	9.99E-14	7213

ADGRE2	rs67023356	19	14396821	A	G	0.232081	0.490378	0.018833	6.28E-143	7213
ADGRE2	rs12461521	19	14780598	C	T	0.433315	0.213313	0.0167319	7.89E-37	7213
FUT3	rs708686	19	5840608	C	T	0.292042	-0.682224	0.0165747	0	7213
FUT3	rs117284005	19	5926398	A	C	0.0183003	-0.40452	0.0617802	6.24E-11	7213
FUT5	rs778809	19	5830291	G	A	0.330306	-0.509359	0.0166862	1.40E-192	7213
FUT5	rs8101385	19	5838584	C	T	0.0786774	-0.353554	0.0309891	6.80E-30	7213
FUT5	rs11666151	19	5871651	G	A	0.412866	-0.336084	0.0162727	4.07E-92	7213
KYNU	rs6734849	2	142985443	T	G	0.408984	0.11918	0.0167402	1.19E-12	7213
KYNU	rs16858549	2	143043227	T	C	0.171704	-0.877743	0.0194135	0	7213
PLCG1	rs753381	20	41168825	T	C	0.450437	-0.153841	0.0165639	2.05E-20	7213
PLXNC1	rs2291331	12	94227410	T	A	0.424373	-0.540059	0.0154975	4.80E-246	7213
PLXNC1	rs11107483	12	94254327	C	G	0.200957	0.177226	0.0206433	1.10E-17	7213
SLITRK5	rs9525140	13	87250230	C	T	0.342299	0.118253	0.0174349	1.27E-11	7213
SLITRK5	rs12856670	13	88034278	C	T	0.291141	-0.22543	0.0181012	3.07E-35	7213
EPB41	rs204074	1	28865051	T	C	0.0716068	-0.295365	0.0319945	3.44E-20	7213
EPB41	rs12403568	1	29131266	G	T	0.482116	-0.100374	0.0166658	1.80E-09	7213
YWHAH	rs5994442	22	31875946	C	T	0.257175	-0.106774	0.0188597	1.56E-08	7213
PDIA3	rs12900924	15	43737329	C	T	0.179398	-0.148016	0.0216159	8.13E-12	7213
AFM	rs72856690	4	73507241	G	A	0.0210037	-1.14918	0.0564859	1.63E-89	7213
AFM	rs2091583	4	73550995	T	C	0.0262027	0.420784	0.0518675	5.77E-16	7213
SMPDL3A	rs28385609	6	122801319	C	T	0.144323	-0.482748	0.0228083	1.64E-96	7213
GSN	rs10513364	9	121249890	T	C	0.0097047	-1.0449	0.0838157	2.62E-35	7213
GSN	rs76463933	9	121327414	C	T	0.0304312	-0.481878	0.0480899	1.77E-23	7213
GSN	rs306788	9	121357729	C	T	0.178982	0.150492	0.0217986	5.50E-12	7213
LPO	rs11656836	17	58257173	G	T	0.203175	-0.275333	0.0203232	2.61E-41	7213
ITIH4	rs3617	3	52799789	C	A	0.457993	0.16143	0.016547	2.39E-22	7213
SELL	rs4987353	1	169697846	G	A	0.31062	-0.583599	0.0166611	2.24E-248	7213
SELL	rs3917435	1	169725521	A	T	0.13039	0.170992	0.0247543	5.35E-12	7213
EPHA2	rs924204	1	16187431	A	G	0.408291	0.371275	0.0162093	3.85E-112	7213
NTRK2	rs1624327	9	84814375	A	G	0.267572	-0.133728	0.0188136	1.29E-12	7213
VEGFA	rs6921438	6	43957870	G	A	0.478719	-0.177079	0.0166979	4.40E-26	7213
ANG	rs140254447	14	20664500	C	G	0.0391654	0.434493	0.0422936	1.37E-24	7213
ANG	rs12433832	14	20665025	C	T	0.123388	0.220851	0.0250275	1.36E-18	7213
ANG	rs4982325	14	20688677	C	T	0.117219	0.831794	0.0238412	1.42E-246	7213
ANG	rs17242790	14	20698068	G	A	0.0270345	-0.4466	0.051645	6.41E-18	7213
F10	rs547138	13	113137856	T	A	0.396229	-0.286206	0.0166585	7.14E-65	7213
F10	rs2480948	13	113163223	T	C	0.18737	-0.240833	0.0210188	3.90E-30	7213
CCL7	rs17614093	17	34182237	C	G	0.430195	0.126308	0.0168571	7.54E-14	7213
CCL7	rs3138037	17	34320725	T	C	0.167267	-0.659528	0.0211211	7.00E-201	7213
F5	rs1591734	1	169302664	C	T	0.142105	-0.18865	0.0237728	2.41E-15	7213
F5	rs61808983	1	169505159	C	T	0.0490087	-0.831266	0.0370778	1.14E-107	7213
F5	rs6685578	1	169566115	C	G	0.485027	-0.115112	0.0164985	3.28E-12	7213
ENG	rs11794565	9	127838998	C	T	0.456329	0.241888	0.0163827	1.26E-48	7213

GSTP1	rs140960667	11	67168860	C	T	0.0173991	0.45092	0.0624738	5.83E-13	7213
GSTP1	rs117149657	11	67580109	T	A	0.0153196	0.522659	0.0673977	1.01E-14	7213
GSTP1	rs1695	11	67585218	A	G	0.33807	-0.582943	0.0161336	6.59E-263	7213
CCL16	rs117559122	17	35750440	C	T	0.0449189	-0.222671	0.0402696	3.32E-08	7213
CCL16	rs10445391	17	35979070	A	G	0.0919174	-1.29322	0.0244563	0	7213
MMP1	rs57701631	11	102786249	T	C	0.150631	-0.186026	0.0228782	4.97E-16	7213
MMP1	rs1144396	11	102808321	C	A	0.499237	-0.309328	0.0162245	4.22E-79	7213
MMP13	rs655316	11	102961635	T	C	0.317344	0.132737	0.0177097	7.41E-14	7213
SHBG	rs11651783	17	7544593	C	T	0.214751	-0.306614	0.0200799	7.83E-52	7213
SHBG	rs1050541	17	7657517	T	G	0.459032	-0.131251	0.0167358	5.05E-15	7213
STC1	rs4872196	8	23834554	C	T	0.110703	0.188057	0.0261855	7.57E-13	7213
ANXA1	rs2795108	9	73150562	T	C	0.127755	0.255113	0.0246105	5.30E-25	7213
ANXA2	rs11638139	15	60284709	C	G	0.23118	0.118148	0.0197456	2.29E-09	7213
ANXA2	rs8033800	15	60396980	T	A	0.386178	0.23546	0.016997	4.34E-43	7213
CDNF	rs11259349	10	14800948	A	G	0.467351	0.1042	0.0166919	4.55E-10	7213
CDNF	rs61738953	10	14820083	C	G	0.0352835	-1.09924	0.0432188	9.74E-137	7213
ERAP1	rs7736193	5	96720415	G	T	0.348399	-0.331193	0.0168703	1.23E-83	7213
ERAP1	rs245471	5	96837640	G	A	0.487245	0.784879	0.0139729	0	7213
ERAP1	rs72777613	5	97143546	T	A	0.262512	0.130585	0.0186488	2.74E-12	7213
ERAP1	rs1421741	5	97151273	T	A	0.0395813	0.36643	0.0421877	4.60E-18	7213
CAPG	rs11684231	2	85400188	C	T	0.217871	0.321955	0.020029	3.76E-57	7213
CAPG	rs62623452	2	85402143	C	T	0.0128934	-1.7656	0.0700509	2.06E-134	7213
CAPG	rs79923841	2	85428082	A	T	0.0266879	-0.418173	0.0514311	4.98E-16	7213
CA1	rs116866430	8	85333298	T	G	0.00915015	-1.58931	0.0851238	5.22E-76	7213
CA1	rs2645050	8	85358432	A	G	0.161375	0.233627	0.0224457	3.42E-25	7213
CTSZ	rs75940688	20	59220098	G	A	0.0119229	-0.925535	0.0754721	3.12E-34	7213
CRK	rs12936625	17	1434769	T	C	0.367808	0.20039	0.0170939	1.87E-31	7213
DPT	rs142979375	1	168550512	G	A	0.0251629	-0.326059	0.0530945	8.63E-10	7213
DPT	rs1018454	1	168728523	A	C	0.423125	-0.431681	0.01601	1.21E-152	7213
DPT	rs9633332	1	168950339	G	A	0.36656	0.156276	0.0171255	9.13E-20	7213
DSC3	rs35630063	18	31024408	T	C	0.0211424	-0.338506	0.0577683	4.84E-09	7213
PI3	rs56168207	20	45175330	C	T	0.182379	0.376689	0.0211969	3.49E-69	7213
ESD	rs9778	13	46779966	C	T	0.100028	-0.843938	0.0261843	4.84E-213	7213
ESD	rs17069005	13	46849983	A	G	0.103078	-0.185778	0.0273638	1.22E-11	7213
FGG	rs2227399	4	154564701	T	G	0.209344	0.218955	0.0202549	4.97E-27	7213
GP1BA	rs1060431	17	4937573	G	A	0.0783308	0.368647	0.0306218	4.61E-33	7213
GPC5	rs1330064	13	91405002	G	A	0.345834	-0.314598	0.0171684	2.41E-73	7213
GPC5	rs342706	13	91764804	T	C	0.277	-0.686856	0.0168175	0	7213
GRN	rs5848	17	44352876	C	T	0.281575	-0.24927	0.0182402	5.40E-42	7213
HRG	rs61004104	3	186659662	C	T	0.46132	0.654765	0.0145083	0	7213
HRG	rs1656909	3	186721079	A	G	0.190143	0.214455	0.0211756	6.03E-24	7213
HRG	rs73185681	3	186771052	G	A	0.0511576	0.310238	0.0375978	1.84E-16	7213
LGALS3BP	rs3826311	17	78975444	T	C	0.178982	0.218327	0.0215831	6.79E-24	7213

MAPK12	rs3817818	22	50260902	C	G	0.459795	0.206294	0.0165713	3.26E-35	7213
MAPK13	rs12210904	6	36130414	C	A	0.282823	0.146546	0.0184461	2.24E-15	7213
MAPK14	rs12200998	6	36114905	G	A	0.465548	-0.146343	0.0166563	1.91E-18	7213
AK1	rs78181560	9	127878806	G	T	0.0351449	-0.714771	0.0446887	1.30E-56	7213
PLA2G7	rs953062	6	46658616	A	G	0.324553	0.130824	0.0178242	2.38E-13	7213
PRDX6	rs33951697	1	173489187	C	T	0.184112	0.513529	0.0204699	3.13E-133	7213
PPA1	rs12570967	10	70212594	C	T	0.0485928	-0.227206	0.0383921	3.41E-09	7213
ADSL	rs8192456	22	40350208	C	T	0.142312	0.129038	0.0235775	4.58E-08	7213
FAP	rs12477178	2	162209363	G	A	0.0133093	1.11477	0.0711618	2.05E-54	7213
PRSS2	rs1799886	7	142800839	T	C	0.430265	0.390392	0.0161979	1.67E-123	7213
PRSS2	rs148648889	7	143123547	G	A	0.0155968	-0.489777	0.0668553	2.63E-13	7213
TNFAIP6	rs35233519	2	151141532	C	T	0.352974	-0.112727	0.0175378	1.38E-10	7213
TNFAIP6	rs2278089	2	151290158	G	T	0.402052	0.45787	0.0159246	3.28E-172	7213
CD274	rs822342	9	5453973	T	C	0.258284	-0.36538	0.0186618	3.28E-83	7213
ICOSLG	rs2838537	21	44263692	G	T	0.351033	0.159332	0.0173271	4.80E-20	7213
CD244	rs114594620	1	160731600	T	C	0.0174685	-0.356236	0.0639727	2.66E-08	7213
CD300C	rs62087200	17	74463637	G	A	0.049286	-0.764918	0.0373564	1.28E-90	7213
CD300C	rs9912017	17	74487717	T	G	0.270692	-0.12267	0.018636	4.95E-11	7213
CD55	rs3748671	1	207079686	C	T	0.438167	-0.11054	0.0167301	4.19E-11	7213
CD55	rs1583091	1	207262693	C	T	0.242271	-0.793445	0.0168954	0	7213
CD55	rs147774007	1	207655896	G	A	0.0243311	0.317078	0.054259	5.33E-09	7213
TNFRSF6B	rs8957	20	63742354	G	T	0.266325	0.151545	0.0185781	4.01E-16	7213
EPHA10	rs12074120	1	37731729	T	G	0.309719	0.155682	0.018069	8.43E-18	7213
EPHB6	rs7789303	7	142854785	G	A	0.280882	-0.59113	0.0171843	2.96E-240	7213
EPHB6	rs6943031	7	142939774	G	A	0.018023	0.364761	0.0620194	4.25E-09	7213
IL20RA	rs1775296	6	137005667	T	G	0.282545	0.126066	0.0184463	8.92E-12	7213
IL22RA2	rs6917836	6	137132089	G	A	0.105781	-0.223758	0.0268908	1.03E-16	7213
IL22RA2	rs2234711	6	137219383	A	G	0.380355	0.135001	0.0169594	1.98E-15	7213
IL23R	rs12569203	1	67132664	A	C	0.450575	0.101877	0.0165265	7.45E-10	7213
IL23R	rs11581607	1	67242007	G	A	0.0683488	-0.201988	0.0327826	7.60E-10	7213
IL7R	rs10058572	5	35880755	G	T	0.404617	0.147757	0.0168059	1.81E-18	7213
LILRB1	rs2781771	19	54630849	A	G	0.051227	-1.48787	0.0335634	0	7213
LILRB1	rs1654657	19	54679190	T	G	0.452794	0.105803	0.0166194	2.06E-10	7213
LILRB2	rs393665	19	54251997	T	A	0.282476	0.222997	0.0182229	4.28E-34	7213
LILRB2	rs399657	19	54289650	G	A	0.216276	-0.806524	0.0178899	0	7213
JAG1	rs2423507	20	10633691	C	G	0.36656	0.108984	0.0173585	3.62E-10	7213
JAG1	rs1051412	20	10673915	A	C	0.498822	0.0943502	0.0166376	1.48E-08	7213
KIR2DL4	rs111505776	19	54729287	C	A	0.0321641	0.262283	0.0470208	2.52E-08	7213
KIR2DL4	rs10500318	19	54809324	G	A	0.158533	-0.554202	0.021729	1.85E-137	7213
KIR2DL4	rs652188	19	54850487	G	T	0.383544	0.627318	0.0153878	0	7213
KIR2DL4	rs35043300	19	54865069	C	T	0.252877	-0.53385	0.0180318	6.76E-182	7213
CD200R1	rs62263730	3	112859161	G	A	0.30244	0.245101	0.0178419	2.07E-42	7213
CD200R1	rs13081854	3	113094528	A	G	0.264384	0.106437	0.0188752	1.78E-08	7213

RTN4R	rs75766	22	20187330	C	A	0.253015	0.401726	0.0184868	1.80E-101	7213
RTN4R	rs685667	22	20202099	C	T	0.279773	0.203499	0.0181087	4.64E-29	7213
RTN4R	rs1642173	22	20222596	G	A	0.308956	-0.220931	0.0179847	2.42E-34	7213
NOTCH1	rs9411207	9	136514099	C	T	0.346458	0.188335	0.0174161	4.77E-27	7213
NOTCH3	rs4808238	19	15192918	G	A	0.14973	0.217443	0.0232101	9.65E-21	7213
CD200	rs146515723	3	112190140	C	T	0.0485235	-0.235553	0.0382375	7.65E-10	7213
CD200	rs1879514	3	112308988	G	T	0.23527	-0.219756	0.0194931	3.11E-29	7213
SEMA6B	rs4353572	19	4563925	T	C	0.491751	-0.138043	0.0163982	4.57E-17	7213
ICAM5	rs901886	19	10291455	T	C	0.479828	0.636706	0.0151848	0	7213
ICAM5	rs58852827	19	10342333	G	A	0.0926799	-0.223075	0.0286791	8.37E-15	7213
ICAM5	rs118167193	19	10577400	A	G	0.0406211	-0.56127	0.0420999	4.49E-40	7213
SLAMF6	rs1041067	1	160487337	C	T	0.201858	-0.388563	0.0203182	1.45E-79	7213
SCARF1	rs35678363	17	1633588	G	A	0.358727	0.826142	0.014348	0	7213
SCARF1	rs72820397	17	1700051	T	C	0.242756	0.246693	0.019167	1.70E-37	7213
TNFRSF19	rs3814787	13	23578231	G	C	0.299529	0.191643	0.0178833	1.35E-26	7213
IL27RA	rs149677133	19	13925319	G	A	0.0127547	0.417367	0.0740874	1.83E-08	7213
IL27RA	rs35026308	19	14042481	T	C	0.163386	-1.01263	0.0191315	0	7213
HAVCR2	rs4704819	5	156906967	A	G	0.222723	-0.148715	0.0199196	9.26E-14	7213
HAVCR2	rs6891966	5	157099320	A	G	0.22813	0.755431	0.0176898	0	7213
HAVCR2	rs140388519	5	157552083	G	A	0.109317	0.190004	0.026231	4.83E-13	7213
UNC5C	rs1994292	4	95521608	C	T	0.349369	0.313902	0.0170329	3.79E-74	7213
UNC5C	rs11931784	4	95573655	C	T	0.22612	-0.111809	0.0199011	2.00E-08	7213
UNC5C	rs987383	4	95626201	A	T	0.164703	-0.201707	0.0220551	7.59E-20	7213
PPID	rs28571231	4	158229723	G	A	0.484958	0.112213	0.0166234	1.59E-11	7213
PPID	rs56172511	4	158704501	G	A	0.221683	0.831982	0.0175877	0	7213
PPID	rs12508886	4	158967051	G	A	0.186122	0.171203	0.0211254	6.19E-16	7213
GCKR	rs1260326	2	27508073	T	C	0.406558	-0.197844	0.0168361	1.35E-31	7213
IMPDH1	rs1053124	7	128501811	T	C	0.200541	0.120469	0.0207683	6.89E-09	7213
PCSK9	rs11591147	1	55039974	G	T	0.0144877	-1.04454	0.0687454	2.39E-51	7213
PCSK9	rs693668	1	55055436	G	A	0.356994	-0.165634	0.0172284	9.42E-22	7213
PPIE	rs1046988	1	39753393	C	T	0.364273	-0.592572	0.0157718	2.63E-282	7213
PPIF	rs12252016	10	79324912	T	A	0.0168446	-0.453721	0.0639377	1.40E-12	7213
PDE5A	rs58583086	4	119635207	A	G	0.37273	0.456714	0.0165724	4.82E-159	7213
PDE5A	rs143179501	4	119836365	C	A	0.0170525	-0.365053	0.0639112	1.16E-08	7213
SHC1	rs61751623	1	154965738	C	G	0.0478303	-0.295485	0.0391208	4.77E-14	7213
VAV1	rs8106212	19	6802560	C	T	0.0142798	0.38532	0.0701774	4.14E-08	7213
VAV1	rs36097961	19	6850756	C	T	0.0765285	-0.580725	0.0303145	7.99E-80	7213
CCL11	rs6505397	17	34190977	T	A	0.419104	-0.147153	0.0168147	2.59E-18	7213
APOE	rs429358	19	44908684	T	C	0.14973	0.299981	0.023212	8.70E-38	7213
APOE	rs35136575	19	44935906	C	G	0.232843	-0.125203	0.0195489	1.60E-10	7213
F2	rs78807356	11	46575120	G	T	0.0261334	0.505516	0.0519699	3.16E-22	7213
F2	rs113954134	11	47181131	G	A	0.0352142	0.275843	0.0447637	7.56E-10	7213
S100A9	rs724781	1	153363542	C	G	0.266325	-0.154398	0.0187673	2.26E-16	7213

CPNE1	rs6058227	20	35308144	C	T	0.0806183	-0.202066	0.0307108	5.05E-11	7213
CPNE1	rs2050729	20	35706487	A	G	0.135519	-0.919494	0.0214825	0	7213
DLL1	rs3012384	6	170153137	T	C	0.38694	0.101338	0.0171497	3.60E-09	7213
DLL1	rs4710790	6	170279144	A	C	0.427145	0.175398	0.0166574	9.68E-26	7213
IL1RN	rs6728769	2	113050934	T	C	0.307639	0.277154	0.0175425	2.66E-55	7213
IL1RN	rs55709272	2	113109711	T	C	0.440385	-0.37788	0.0162943	8.23E-115	7213
IL1RN	rs117768367	2	113307014	C	T	0.0198946	0.415158	0.0593795	2.96E-12	7213
OMD	rs10992426	9	92703641	G	A	0.222584	-0.155488	0.0197077	3.48E-15	7213
SEMA3E	rs111685565	7	83085098	A	G	0.0408291	0.283947	0.0417829	1.16E-11	7213
SEMA3E	rs73707827	7	83410464	T	A	0.122556	-1.07026	0.0219461	0	7213
SEMA3E	rs302121	7	83644459	T	A	0.216484	0.347594	0.0197257	4.44E-68	7213
SEMA3E	rs11764301	7	83793904	A	T	0.330861	-0.12371	0.0177863	3.82E-12	7213
FAS	rs1389	10	88942948	G	A	0.18945	-0.137198	0.0212179	1.07E-10	7213
FAS	rs982764	10	89010241	T	C	0.304242	-0.407148	0.0174679	6.47E-116	7213
FAS	rs61854881	10	89119147	A	C	0.0187855	-0.338531	0.0613076	3.47E-08	7213
LEPR	rs78007650	1	65235626	G	A	0.0433246	-0.248804	0.0408875	1.22E-09	7213
LEPR	rs1171279	1	65522810	C	T	0.261403	0.185882	0.0187482	5.04E-23	7213
LEPR	rs72925337	1	65640019	C	T	0.190143	-1.15352	0.0164522	0	7213
TNFRSF21	rs11754478	6	47168389	G	A	0.415846	0.141037	0.0167556	4.60E-17	7213
TNFRSF21	rs6458555	6	47309833	G	A	0.319908	0.286235	0.0172871	1.82E-60	7213
SIRPA	rs56278099	20	1913961	A	G	0.377513	-1.04769	0.0118266	0	7213
SIRPA	rs6045428	20	1919121	G	A	0.0178151	0.445558	0.062781	1.40E-12	7213
SIRPA	rs4566427	20	2071168	C	G	0.0579509	-0.236403	0.0357101	3.85E-11	7213
ALCAM	rs78470897	3	105368773	G	A	0.0210037	0.481275	0.0578072	9.95E-17	7213
ALCAM	rs751554	3	105418999	C	T	0.116526	-0.350204	0.0257729	1.53E-41	7213
ASGR1	rs434325	17	7108030	A	C	0.437613	-0.10127	0.0165289	9.44E-10	7213
ASGR1	rs62061425	17	7169772	A	G	0.193054	-0.243888	0.0209295	4.21E-31	7213
CNDP1	rs17817077	18	74542308	G	A	0.395674	0.244799	0.016778	1.53E-47	7213
CNDP1	rs58692747	18	74565502	T	C	0.200957	-0.160138	0.020753	1.36E-14	7213
COLEC12	rs2846666	18	469797	C	A	0.276099	-0.222127	0.018572	1.17E-32	7213
COLEC12	rs12955797	18	589353	G	C	0.278456	0.109916	0.0184062	2.46E-09	7213
CST1	rs6036492	20	23721534	C	A	0.430126	0.147461	0.0166446	1.00E-18	7213
CST1	rs4260306	20	23749699	C	T	0.282961	-0.506384	0.0175888	1.24E-172	7213
DDX19B	rs61757207	16	70324592	A	G	0.0140025	-0.675044	0.0709063	2.31E-21	7213
FCN3	rs111257457	1	27389173	C	A	0.0946901	-0.542905	0.0276708	1.53E-83	7213
GAS1	rs2150719	9	86584460	T	C	0.471995	0.153376	0.0165616	2.63E-20	7213
GAS1	rs4878043	9	87072939	C	T	0.45418	-0.155893	0.0166356	9.42E-21	7213
GRB2	rs9889402	17	75368135	G	A	0.283031	-0.133859	0.0183155	2.99E-13	7213
HS6ST1	rs4662790	2	128294778	A	G	0.413905	0.251496	0.016521	1.57E-51	7213
PRKCB	rs2023671	16	23838518	G	T	0.25149	0.29896	0.0187119	1.71E-56	7213
PRKCB	rs12919956	16	23850005	C	T	0.210869	-0.143415	0.020359	2.04E-12	7213
FOLH1	rs55728336	11	49156256	T	C	0.0506724	0.341771	0.0378777	2.31E-19	7213
CCL5	rs2107538	17	35880776	C	T	0.178844	-0.612013	0.0205638	1.10E-183	7213

RGMA	rs10852188	15	93064200	A	G	0.427631	0.241869	0.0166359	3.18E-47	7213
RGMA	rs6497022	15	93109593	G	C	0.264384	0.12631	0.0189609	2.91E-11	7213
RGMA	rs8036731	15	93483598	G	A	0.237211	0.108814	0.0194883	2.44E-08	7213
SLAMF7	rs489286	1	160752760	A	G	0.33703	-0.671949	0.015752	0	7213
SLAMF7	rs3905346	1	160904497	T	A	0.261888	0.125924	0.0190102	3.75E-11	7213
SLAMF7	rs72714915	1	161166553	C	G	0.0792319	-0.403805	0.0305767	2.31E-39	7213
SPOCK2	rs3312	10	72097226	A	G	0.464162	0.251867	0.0163783	1.56E-52	7213
EGF	rs10029654	4	109940771	G	A	0.332525	0.383437	0.0170452	2.31E-108	7213
EGF	rs17041301	4	110055602	C	T	0.0499792	-0.227763	0.0381073	2.38E-09	7213
FGFR1	rs62505473	8	38466247	C	G	0.405864	0.144198	0.0166978	7.10E-18	7213
FGFR1	rs11997244	8	38701808	C	T	0.453625	-0.148486	0.0166296	5.38E-19	7213
NRP1	rs117514859	10	32998517	G	A	0.0183003	-0.416867	0.0615314	1.34E-11	7213
NRP1	rs734186	10	33195901	G	A	0.378691	-0.394068	0.0165709	2.08E-120	7213
FGL1	rs34807569	8	17881720	G	C	0.31062	-0.720298	0.0158888	0	7213
FGL1	rs2517343	8	18114531	T	C	0.0691806	-0.247896	0.0324103	2.29E-14	7213
FGL1	rs2220734	8	18373510	T	C	0.448773	0.105032	0.0168558	4.89E-10	7213
TCN2	rs9606716	22	30332464	G	A	0.0207958	0.418181	0.0581476	7.04E-13	7213
TCN2	rs2528456	22	30568916	A	G	0.0291834	0.499215	0.0493658	7.01E-24	7213
TCN2	rs12169610	22	30626603	C	T	0.119853	-0.828155	0.0236756	9.52E-248	7213
TCN2	rs34524925	22	30666043	T	C	0.134202	-0.398729	0.0240224	9.52E-61	7213
TCN2	rs13058171	22	30701437	A	G	0.10086	0.26415	0.0274737	9.36E-22	7213
MINPP1	rs59980852	10	87515989	C	T	0.458339	-0.150282	0.0165993	1.75E-19	7213
PDIA5	rs72974405	3	123058028	G	C	0.0275891	0.578451	0.0504269	3.37E-30	7213
PDIA5	rs9812472	3	123077614	T	G	0.289963	-0.248282	0.0179957	9.28E-43	7213
PDIA5	rs836853	3	123122947	G	T	0.204423	0.830882	0.0180791	0	7213
PDIA5	rs73188536	3	123506924	A	G	0.0830445	-0.169889	0.0301743	1.87E-08	7213
GREM2	rs116135446	1	240794633	C	T	0.0411063	0.229128	0.0417067	4.07E-08	7213
PGLYRP2	rs10164310	19	15475134	G	A	0.149314	-0.423254	0.0228809	1.12E-74	7213
MFNG	rs563027675	22	37426797	C	G	0.0201719	-0.573642	0.0592303	4.76E-22	7213
FAM19A5	rs5768649	22	48471219	C	T	0.112297	-0.169841	0.0263984	1.32E-10	7213
FAM3B	rs57529409	21	41346335	C	T	0.0526133	-0.659898	0.0360974	5.13E-73	7213
FAM3B	rs2838023	21	41351572	G	C	0.0494246	-0.260318	0.0380899	8.91E-12	7213
PAM	rs72779935	5	102378177	G	C	0.049286	0.225045	0.0377245	2.55E-09	7213
PAM	rs7704999	5	102685049	G	C	0.0237765	-0.369679	0.0544755	1.24E-11	7213
PAM	rs12656561	5	102989988	T	C	0.354083	-0.574245	0.0163209	2.01E-250	7213
THSD1	rs12397524	13	52223221	C	T	0.0490087	-0.38206	0.0386643	7.00E-23	7213
THSD1	rs41292808	13	52397382	C	T	0.0246083	1.52287	0.050543	5.92E-188	7213
THSD1	rs76490335	13	52732745	G	A	0.0897685	-0.178113	0.0289982	8.57E-10	7213
SEMA3G	rs2016575	3	52443064	T	C	0.166921	0.362468	0.022024	8.97E-60	7213
CD300A	rs72852270	17	74394226	G	A	0.0244697	-0.372537	0.054065	6.03E-12	7213
CD300A	rs2670839	17	74458195	G	C	0.184389	-0.815836	0.0188499	0	7213
CD300A	rs148420928	17	74483513	C	T	0.031055	0.293169	0.047857	9.49E-10	7213
CD300A	rs11868846	17	74555287	C	G	0.0644669	0.359344	0.0335505	1.44E-26	7213

CRTAC1	rs684225	10	97882605	C	A	0.414807	-0.36741	0.0161697	1.90E-110	7213
CRTAC1	rs117781346	10	98150009	C	T	0.0133093	-0.646224	0.0727358	8.00E-19	7213
POFUT1	rs76143353	20	32227952	C	T	0.0619021	-1.03236	0.032229	1.30E-210	7213
MFAP4	rs139356332	17	19385973	G	C	0.0215583	-0.866868	0.0562735	1.05E-52	7213
MFAP4	rs1129696	17	19578472	A	G	0.122279	-0.148124	0.0256302	7.81E-09	7213
NTNG1	rs115668827	1	107135646	G	C	0.0502565	1.29214	0.034064	2.96E-287	7213
COLGALT1	rs3746317	19	17556110	C	T	0.438722	-0.141092	0.0166935	3.43E-17	7213
RNASE4	rs76543014	14	20673471	A	G	0.110911	-0.265411	0.0264309	1.43E-23	7213
RNASE4	rs1888560	14	20696086	C	T	0.313947	-0.51182	0.0168986	8.65E-190	7213
RNASE6	rs10133492	14	20716517	C	T	0.436642	0.15534	0.0167996	3.00E-20	7213
RNASE6	rs7152772	14	20776343	T	C	0.228199	0.15647	0.0200422	6.68E-15	7213
RNASE6	rs4981332	14	20793858	T	C	0.270622	0.769512	0.0162442	0	7213
CTRB2	rs12922905	16	75138186	G	A	0.47553	-0.159544	0.016465	4.54E-22	7213
CTRB2	rs8048371	16	75241649	C	T	0.191321	0.924826	0.0183349	0	7213
CTRB2	rs7192155	16	75348296	A	G	0.230071	-0.306268	0.0193043	9.59E-56	7213
PSG4	rs4078629	19	43187772	T	G	0.380909	0.660375	0.0149853	0	7213
PSG4	rs111712264	19	43343853	T	G	0.0768751	-0.1831	0.0308896	3.22E-09	7213
PSG7	rs117338918	19	42873169	C	G	0.0140718	0.530955	0.069842	3.28E-14	7213
PSG7	rs1810386	19	43028388	G	A	0.436989	0.0995258	0.0164516	1.52E-09	7213
ENDOU	rs1153977	12	47641073	T	A	0.466449	-0.114451	0.0165771	5.48E-12	7213
ST3GAL1	rs9643300	8	133490905	C	T	0.434147	0.357289	0.0164201	1.00E-101	7213
ST3GAL1	rs10956709	8	133539043	A	G	0.195688	-0.131117	0.0211526	6.01E-10	7213
F13B	rs10922096	1	196693329	T	C	0.458339	-0.246961	0.0166011	2.52E-49	7213
F13B	rs1332668	1	197036606	C	T	0.470054	-0.504522	0.0156926	4.65E-212	7213
SOD3	rs2536512	4	24799693	G	A	0.337169	-0.328159	0.0172178	4.72E-79	7213
PF4V1	rs872914	4	73852384	G	A	0.290101	0.122594	0.0182735	2.11E-11	7213
CTRB1	rs75562502	16	75027004	T	C	0.0183696	0.596729	0.0616928	5.36E-22	7213
CTRB1	rs8057145	16	75222353	A	G	0.192777	-0.614443	0.0199739	2.29E-195	7213
ASIP	rs6059655	20	34077942	A	G	0.0793706	0.926197	0.0283812	5.26E-218	7213
OBP2B	rs4454354	9	133214142	T	C	0.21288	-0.35204	0.0199241	1.98E-68	7213
VASN	rs757593	16	4391616	A	C	0.270692	-0.133906	0.0187377	9.79E-13	7213
PRKCSH	rs11557488	19	11447460	G	A	0.204631	0.287358	0.0201693	1.92E-45	7213
CBLN4	rs4811612	20	55694664	C	T	0.355608	0.236976	0.0172555	2.18E-42	7213
CBLN4	rs6014495	20	55778059	G	A	0.303688	-0.200602	0.0180325	1.63E-28	7213
CBLN4	rs2870718	20	56179321	A	G	0.291488	-0.187168	0.0180905	6.50E-25	7213
CRISPLD2	rs8055092	16	84803874	A	T	0.33502	0.11134	0.0173579	1.50E-10	7213
CRISPLD2	rs72799587	16	84910384	G	C	0.0873423	-0.194008	0.0293946	4.40E-11	7213
CLEC3B	rs10865936	3	44995308	G	A	0.216484	-0.356188	0.0196213	4.79E-72	7213
CLEC3B	rs76951547	3	44996018	A	G	0.109178	-0.20868	0.0267511	7.02E-15	7213
OLFM1	rs7871721	9	134859278	C	T	0.0712602	-0.212576	0.0320784	3.68E-11	7213
OLFM1	rs10858334	9	135097939	C	G	0.141203	0.198987	0.0237176	5.80E-17	7213
GZMM	rs8107361	19	538160	A	G	0.0734784	-0.209342	0.0316115	3.79E-11	7213
LEAP2	rs60545802	5	132868885	T	G	0.171843	0.41656	0.0215684	4.50E-81	7213

IFNL3	rs62120535	19	39270392	C	G	0.189242	0.119778	0.0211687	1.59E-08	7213
PRCP	rs2229437	11	82853252	T	G	0.176487	0.344918	0.0214074	2.09E-57	7213
B3GALTL	rs4943306	13	31320378	C	T	0.226951	-0.346313	0.0194944	3.88E-69	7213
FCRL1	rs4971154	1	157802090	C	T	0.481908	0.407539	0.015905	1.16E-138	7213
SPINK6	rs11742183	5	147947036	C	T	0.0786081	0.210601	0.0307878	8.54E-12	7213
SPINK6	rs111849861	5	147989676	T	C	0.0567725	0.293002	0.0357236	2.78E-16	7213
SPINK6	rs11741093	5	148221967	C	T	0.0736171	-1.01238	0.0294948	2.74E-239	7213
TREML2	rs2223592	6	40997467	C	A	0.483849	-0.153153	0.0167317	7.06E-20	7213
TREML2	rs4714431	6	41200599	A	C	0.358727	0.505081	0.0162223	8.54E-200	7213
SEMA4D	rs12115280	9	89026648	C	T	0.133093	-0.139921	0.0243588	9.61E-09	7213
SEMA4D	rs3211650	9	89310312	C	T	0.0932344	-1.07813	0.0258892	0	7213
SEMA4D	rs74871955	9	89395132	T	C	0.0518508	0.386476	0.0375558	1.15E-24	7213
ROBO1	rs3773232	3	78725105	C	T	0.198184	0.340381	0.0206271	4.46E-60	7213
ROBO1	rs328049	3	78936655	G	A	0.475392	0.101549	0.0165017	7.97E-10	7213
ACP6	rs12022413	1	147525421	A	C	0.285942	0.162323	0.0182892	8.68E-19	7213
ACP6	rs12045115	1	147646379	T	C	0.425828	-0.684357	0.0148945	0	7213
CLPS	rs9380534	6	35783795	G	A	0.395744	-0.415573	0.0164582	7.11E-135	7213
CLPS	rs111968111	6	35930341	C	T	0.0156662	-0.377593	0.0674249	2.22E-08	7213
CLPS	rs112065360	6	36014652	C	A	0.138223	-0.217068	0.0238954	1.33E-19	7213
AFP	rs667	4	73414548	C	T	0.237141	-0.129298	0.0195086	3.65E-11	7213
C3	rs2230199	19	6718376	G	C	0.201442	0.225902	0.0204551	3.94E-28	7213
TDGF1	rs6794103	3	46082110	T	C	0.445792	-0.110983	0.0165811	2.34E-11	7213
TDGF1	rs11710224	3	46544788	C	G	0.274851	0.697821	0.0168158	0	7213
TDGF1	rs4465978	3	46596733	G	T	0.0263413	0.837699	0.0511764	3.69E-59	7213
TDGF1	rs142106468	3	46863682	T	C	0.0322335	0.310376	0.0469345	4.04E-11	7213
TDGF1	rs143851020	3	47014506	C	T	0.0203799	0.450515	0.0576706	6.43E-15	7213
IL9	rs1799962	5	135896167	T	C	0.0719534	-0.181859	0.0323899	2.04E-08	7213
LIFR	rs3729741	5	38496677	C	T	0.340496	0.252336	0.0172538	9.36E-48	7213
LIFR	rs6884185	5	38723708	G	C	0.465826	0.142377	0.0166503	1.47E-17	7213
S100A12	rs3014874	1	153365467	G	A	0.251698	-0.278032	0.0188555	1.67E-48	7213
S100A12	rs61803119	1	153417726	G	T	0.109039	0.1913	0.0265302	6.14E-13	7213
EIF5A	rs11658072	17	7311505	C	T	0.37883	0.195134	0.0169853	2.77E-30	7213
HINT1	rs12332117	5	131157469	C	T	0.247886	0.314202	0.0189067	6.87E-61	7213
TNFSF12	rs62059804	17	7550188	C	A	0.256412	0.180916	0.0189874	2.14E-21	7213
DMKN	rs7408798	19	35512067	G	A	0.107098	-0.212346	0.0271965	6.64E-15	7213
BOLA3	rs1620482	2	74160099	T	C	0.415084	-0.119896	0.0168622	1.27E-12	7213
CRHBP	rs72775791	5	76513461	T	C	0.0998198	0.160481	0.0275527	5.97E-09	7213
CRHBP	rs13186835	5	76914295	G	T	0.356301	0.649294	0.015584	0	7213
CRHBP	rs874228	5	76929100	A	G	0.213642	-0.131897	0.0202257	7.44E-11	7213
PIP	rs73170678	7	143139110	A	T	0.132816	0.580026	0.0232585	9.64E-132	7213
CECR1	rs2401071	22	17174498	A	G	0.35942	0.118025	0.0172752	9.05E-12	7213
CECR1	rs2231495	22	17188416	T	C	0.337377	-0.837861	0.0144238	0	7213
CECR1	rs2058120	22	17241165	A	G	0.459518	-0.156316	0.0165827	5.60E-21	7213

PCOLCE2	rs371824634	3	142879798	C	T	0.0349369	0.33672	0.0456152	1.74E-13	7213
PCOLCE2	rs11716897	3	142886714	G	A	0.344378	0.491823	0.016507	4.55E-184	7213
PCOLCE2	rs116459600	3	142934677	C	T	0.0244697	0.445493	0.0541495	2.26E-16	7213
CHRDL2	rs6592590	11	74669984	C	T	0.405726	0.165628	0.0167778	7.69E-23	7213
CHRDL2	rs61389091	11	74716876	C	T	0.0420075	-0.751669	0.04074	2.61E-74	7213
PSAP	rs4747200	10	71814648	A	G	0.279911	0.222778	0.0183487	1.35E-33	7213
GUCA2B	rs1047047	1	42153468	A	G	0.153473	0.213397	0.0228446	1.24E-20	7213
KLK10	rs80209459	19	50971789	A	G	0.0521974	0.510261	0.036973	8.77E-43	7213
KLK10	rs2569454	19	51019947	T	C	0.433869	-0.658504	0.0149777	0	7213
VIT	rs79782259	2	36704753	C	G	0.228615	0.337765	0.0193473	7.01E-67	7213
VIT	rs34331280	2	36709305	A	G	0.218286	-0.133544	0.0199891	2.55E-11	7213
VIT	rs1468810	2	36766797	C	A	0.412242	0.520041	0.0158662	9.07E-220	7213
PVRL2	rs440277	19	44857967	G	A	0.318245	-0.141434	0.0177557	1.90E-15	7213
SIRPB1	rs112912530	20	1307318	C	T	0.104533	-0.265706	0.0269621	9.09E-23	7213
SIRPB1	rs17791824	20	1376802	C	T	0.018231	0.670692	0.0623061	8.09E-27	7213
SIRPB1	rs6042710	20	1511424	C	T	0.362471	-0.438437	0.0166252	2.12E-146	7213
SIRPB1	rs2250091	20	1558224	G	A	0.469153	-0.620705	0.0151634	0	7213
SCGB3A1	rs307802	5	180592237	T	C	0.404201	0.117237	0.017022	6.16E-12	7213
CPXM1	rs6051395	20	2789004	T	A	0.219188	-0.251792	0.0198401	1.63E-36	7213
CPXM1	rs6037416	20	2804279	G	A	0.415084	-0.672426	0.0149103	0	7213
CPXM1	rs4813623	20	2989744	A	G	0.0787467	-0.270812	0.0304681	7.73E-19	7213
NPNT	rs34712979	4	105897896	G	A	0.245182	-0.387159	0.018757	5.19E-92	7213
POMGNT2	rs12639294	3	43109244	C	G	0.469915	-0.404904	0.0160639	1.99E-134	7213
TAPBPL	rs10744705	12	6389529	C	G	0.0305005	0.375006	0.0478341	5.17E-15	7213
TAPBPL	rs12369967	12	6518190	G	A	0.288715	0.876633	0.0152628	0	7213
TAPBPL	rs117012163	12	6652727	G	A	0.0201719	0.683202	0.0584372	2.70E-31	7213
TXNDC15	rs3733897	5	134887903	A	G	0.132746	0.472112	0.0236864	4.34E-86	7213
FMOD	rs4971252	1	203351280	G	C	0.12727	-0.440737	0.024365	1.46E-71	7213
DLK1	rs12881545	14	100709875	G	C	0.329613	-0.484803	0.0166383	1.48E-176	7213
XXYLT1	rs7635512	3	195072715	G	C	0.149522	0.292501	0.0228996	5.81E-37	7213
XXYLT1	rs13072883	3	195274025	G	A	0.420006	-0.302452	0.0165909	1.25E-72	7213
ADAMTSL2	rs10120207	9	133547919	C	A	0.0876196	0.178086	0.0296085	1.89E-09	7213
MANBA	rs223507	4	102712658	G	A	0.339803	0.660755	0.0155365	0	7213
MANBA	rs56412006	4	103082836	A	C	0.193817	0.244097	0.0206836	7.52E-32	7213
TLL1	rs1903176	4	165742950	T	A	0.331415	-0.0989996	0.0176353	2.05E-08	7213
VWA1	rs1153094	1	1445240	A	G	0.185984	0.144315	0.0213011	1.34E-11	7213
VWA1	rs116279124	1	1525927	C	T	0.0137252	-1.60513	0.0695232	7.64E-114	7213
CCDC126	rs35121828	7	23595366	G	A	0.256967	0.497549	0.0181575	2.40E-157	7213
WISP2	rs6065746	20	44693361	G	C	0.445584	-0.348585	0.016325	3.82E-98	7213
WISP2	rs753740	20	44726014	G	A	0.411479	-0.164656	0.016742	1.11E-22	7213
HSP90B1	rs111979420	12	103749558	G	A	0.236864	0.138382	0.0194408	1.20E-12	7213
HSP90B1	rs1177457	12	103942349	C	T	0.303203	1.06258	0.0130267	0	7213
HSP90B1	rs117449629	12	104275114	G	A	0.0183696	0.518751	0.0620325	7.30E-17	7213

PILRA	rs1623264	7	100213581	G	C	0.263829	0.795326	0.0162641	0	7213
PILRA	rs314298	7	100773491	C	T	0.44191	-0.252404	0.0165953	1.91E-51	7213
C1QL1	rs7225162	17	44958892	C	T	0.47657	0.327862	0.0160219	1.64E-90	7213
C1QL1	rs138144764	17	45109853	G	A	0.128518	-0.23503	0.0249082	5.13E-21	7213
INHBC	rs6581138	12	57351081	G	A	0.23825	0.110789	0.0194319	1.24E-08	7213
ADGRF5	rs13196132	6	46687866	C	T	0.324761	-0.110488	0.0178574	6.46E-10	7213
ADGRF5	rs657340	6	46855958	G	A	0.0658533	-1.36852	0.0294075	0	7213
OAF	rs494965	11	120182942	C	T	0.43075	0.275795	0.0165533	3.47E-61	7213
OAF	rs2845705	11	120230383	A	T	0.202828	0.706956	0.01897	3.70E-278	7213
CPN2	rs6783228	3	194339757	G	A	0.25967	-0.694947	0.016968	0	7213
CPN2	rs6789147	3	194368182	C	T	0.379454	0.165878	0.0171015	4.13E-22	7213
CPN2	rs73067280	3	194387342	G	C	0.0464439	0.216969	0.0395064	4.11E-08	7213
GKN2	rs12990985	2	68944477	T	C	0.0131013	-0.634771	0.072523	2.56E-18	7213
GKN2	rs62133344	2	68950137	C	A	0.0539304	-0.964384	0.0350041	5.90E-159	7213
GKN2	rs59397125	2	68956828	C	A	0.0876889	0.224333	0.0295301	3.42E-14	7213
MMP19	rs56180965	12	55847804	T	C	0.0482462	-0.317774	0.0386522	2.37E-16	7213
PCYOX1	rs72841129	2	70183395	C	G	0.0648135	0.471823	0.0333625	8.24E-45	7213
PCYOX1	rs2706762	2	70261338	C	T	0.146194	-0.952514	0.0206032	0	7213
FAM20A	rs12453086	17	68535757	C	T	0.230209	-0.283788	0.01965	1.25E-46	7213
FAM20A	rs62088364	17	68650892	A	G	0.0912935	-0.326768	0.028597	5.57E-30	7213
PSG3	rs12462231	19	43043330	G	A	0.157909	0.192124	0.0226296	2.49E-17	7213
PSG3	rs2355433	19	43174936	A	G	0.454388	0.711818	0.0141537	0	7213
SEMA3C	rs917191	7	80941555	G	C	0.421461	-0.17953	0.0166676	7.53E-27	7213
SEMA3C	rs62467052	7	81012590	T	G	0.355123	0.139196	0.0171739	6.14E-16	7213
ASPN	rs2516568	9	92425098	A	T	0.326425	-0.510872	0.0165811	6.13E-196	7213
ASPN	rs10992479	9	92816209	G	T	0.0276584	-0.331066	0.0500304	3.92E-11	7213
HAPLN4	rs55762233	19	19256510	C	G	0.15576	0.281069	0.0228504	1.99E-34	7213
APOC3	rs5141	11	116831407	T	C	0.092264	0.198931	0.0286294	4.02E-12	7213
TIMP4	rs184262	3	12093240	A	G	0.167753	-0.560213	0.0213015	1.21E-145	7213
TIMP4	rs143888770	3	12274853	C	T	0.0254402	-0.370286	0.0526356	2.18E-12	7213
TIMP4	rs56316314	3	12557101	A	G	0.0330653	-0.286362	0.0458239	4.36E-10	7213
POGLUT1	rs17203139	3	119486370	T	G	0.0609317	1.19901	0.0321216	5.65E-279	7213
POGLUT1	rs6438537	3	119558999	T	C	0.11604	-0.204101	0.0258811	3.58E-15	7213
FBLN1	rs11090631	22	45450490	C	T	0.19347	-0.343656	0.0207568	1.86E-60	7213
FBLN1	rs136744	22	45532871	C	G	0.453625	0.140674	0.0166156	3.04E-17	7213
CFHR4	rs10801548	1	196560700	C	T	0.382227	-0.187353	0.0171352	1.31E-27	7213
CFHR4	rs111579215	1	196657050	G	A	0.0150423	0.587395	0.068566	1.29E-17	7213
CFHR4	rs77919582	1	196730965	G	A	0.019756	0.638545	0.0593013	7.81E-27	7213
CFHR4	rs149403976	1	196731888	C	T	0.0233606	-0.51006	0.0547699	1.62E-20	7213
CFHR4	rs151321650	1	196918083	T	G	0.0227367	0.605653	0.0556909	2.46E-27	7213
CFHR4	rs10494745	1	196918327	G	A	0.104811	-0.936087	0.0247348	4.76E-286	7213
CFHR4	rs142834452	1	196948409	G	T	0.0338278	0.626773	0.0460792	1.27E-41	7213
PMEL	rs3213122	12	55969882	C	A	0.0779149	0.451595	0.0308985	1.07E-47	7213

IGLON5	rs7246422	19	51309159	T	C	0.46437	0.173209	0.0166143	2.87E-25	7213
IGLON5	rs10410947	19	51362982	C	T	0.184389	-0.126888	0.0214248	3.32E-09	7213
IGLL1	rs34293649	22	23542427	A	G	0.0166366	-0.367425	0.0652123	1.82E-08	7213
IGLL1	rs9624216	22	23580365	G	A	0.087273	-0.5173	0.0287253	5.89E-71	7213
KLK15	rs73048483	19	50837287	G	A	0.0650908	0.389093	0.0335305	7.37E-31	7213
CPZ	rs6845969	4	8400738	T	A	0.340219	-0.144787	0.0176966	3.28E-16	7213
CPZ	rs115802606	4	8592662	C	A	0.197421	0.129778	0.0207478	4.20E-10	7213
DLK1	rs12881545	14	100709875	G	C	0.329613	-0.477699	0.0166667	3.39E-171	7213
NCAM2	rs11911765	21	21007727	T	C	0.490087	-0.245809	0.0163858	4.08E-50	7213
NCAM2	rs2826851	21	21463626	A	G	0.293359	0.549438	0.0172495	1.98E-208	7213
MGP	rs2900342	12	14876664	A	G	0.384791	-0.428371	0.0163372	8.02E-145	7213
NPTX2	rs12673464	7	98655243	C	T	0.289616	-0.300661	0.0178084	9.50E-63	7213
NPTX2	rs28540061	7	98725577	G	A	0.0955913	-0.219379	0.0282355	8.96E-15	7213
DUSP13	rs6480771	10	75101922	T	C	0.406904	-0.307987	0.0165728	2.45E-75	7213
TRIL	rs740250	7	28957328	T	C	0.127755	0.299451	0.0246966	1.64E-33	7213
PROL1	rs77611689	4	70181247	G	A	0.0660613	0.211482	0.0333244	2.34E-10	7213
PROL1	rs115063133	4	70302141	T	C	0.0176764	-0.361456	0.0630901	1.05E-08	7213
NELL1	rs8176786	11	20937848	C	T	0.0481076	1.09483	0.0367428	4.27E-184	7213
NELL1	rs12416999	11	21087685	G	A	0.186469	0.172655	0.0211852	4.26E-16	7213
SERPINA12	rs8006968	14	94515198	T	A	0.168169	0.284212	0.0219248	5.26E-38	7213
SERPINA12	rs4900236	14	94538816	C	T	0.227783	0.530021	0.0187849	3.13E-166	7213
ENPP5	rs9395125	6	45826235	T	C	0.211562	-0.122476	0.0200787	1.12E-09	7213
ENPP5	rs76412942	6	46106405	C	T	0.0520588	-1.47892	0.0332102	0	7213
ENPP5	rs6918355	6	46187917	C	T	0.18335	-0.185246	0.0214256	6.50E-18	7213
ENPP5	rs74918287	6	46190360	C	A	0.0180923	0.596458	0.0623926	1.58E-21	7213
ENPP5	rs1881030	6	46518333	A	G	0.168238	-0.121518	0.0221527	4.26E-08	7213
LRRC15	rs113691495	3	194362424	G	A	0.0223901	-0.850302	0.0558441	1.49E-51	7213
LRRC15	rs923930	3	194364494	G	A	0.109732	0.841539	0.0246354	3.49E-237	7213
COLEC10	rs2465383	8	119067509	A	G	0.448011	-0.121627	0.0166199	2.79E-13	7213
FAIM3	rs72758947	1	206913708	A	G	0.124012	-0.187416	0.0251412	1.01E-13	7213
ART4	rs77584909	12	14716923	A	G	0.0621101	-0.316782	0.0342378	2.84E-20	7213
ART4	rs7301428	12	14864322	T	C	0.384514	0.960042	0.0128	0	7213
ART4	rs10744085	12	15042070	G	A	0.160336	0.185894	0.0224113	1.29E-16	7213
GALNT3	rs7590559	2	165915153	C	T	0.391169	0.0936173	0.0167336	2.29E-08	7213
NDNF	rs6840113	4	121015352	A	G	0.404686	-0.102137	0.0168254	1.34E-09	7213
IGFALS	rs11641257	16	1822751	T	C	0.0711909	0.268751	0.0318326	3.71E-17	7213
CNP	rs4432296	17	41966789	C	T	0.263205	-0.284189	0.0184656	1.30E-52	7213
FCRL6	rs6656979	1	159812391	C	A	0.192638	-0.470415	0.0203736	7.36E-114	7213
LINGO1	rs2667764	15	77588897	C	T	0.373631	0.105472	0.0171004	7.30E-10	7213
LINGO1	rs62007781	15	77734259	G	A	0.414044	0.134419	0.0167412	1.14E-15	7213
CHST12	rs884566	7	2374915	C	A	0.207265	0.356557	0.0200827	4.63E-69	7213
PNLIPRP1	rs7906926	10	116597627	C	T	0.339041	-0.218421	0.0173082	3.96E-36	7213
DEFB1	rs59164840	8	6862244	A	G	0.12734	-0.156014	0.0252693	7.02E-10	7213

DEFB1	rs2741117	8	6868447	T	G	0.49494	-0.416227	0.0159058	2.81E-144	7213
DEFB1	rs11990152	8	6880941	G	A	0.388188	0.105834	0.0168285	3.38E-10	7213
NTN1	rs9897200	17	9082193	G	T	0.114585	-0.664774	0.0252318	3.88E-146	7213
NTN1	rs8071180	17	9193641	G	A	0.390129	0.322513	0.0164752	3.50E-83	7213
ALPPL2	rs35458538	2	232439642	G	A	0.23728	0.1377	0.0194927	1.77E-12	7213
LRP11	rs117153438	6	149462729	G	C	0.0189935	-0.363401	0.0611981	3.02E-09	7213
LRP11	rs11155682	6	149772845	G	A	0.370927	1.01002	0.0126214	0	7213
LRP11	rs6918437	6	150175829	A	G	0.330653	0.149003	0.0176828	4.26E-17	7213
ALPPL2	rs11681585	2	232315500	A	G	0.120477	-0.193175	0.0253815	3.07E-14	7213
ALPPL2	rs35458538	2	232439642	G	A	0.23728	0.325131	0.0191816	3.18E-63	7213
B3GAT3	rs7122950	11	62617896	C	T	0.290309	-0.416871	0.0177655	2.12E-117	7213
LRRTM2	rs10071381	5	138937159	C	T	0.295231	0.181441	0.0181703	2.50E-23	7213
MGAT2	rs12147470	14	49561954	T	C	0.453695	-0.274587	0.0164699	2.91E-61	7213
HBZ	rs2562164	16	126744	A	G	0.349023	0.104766	0.0173329	1.57E-09	7213
HBZ	rs2541638	16	157612	T	C	0.326494	0.668398	0.0159851	0	7213
HBZ	rs6600189	16	245715	A	C	0.0244697	-0.372498	0.0535907	3.95E-12	7213
HBZ	rs369440	16	280925	A	G	0.097255	-0.198853	0.0277148	7.95E-13	7213
HBZ	rs45619831	16	563729	T	G	0.0334119	0.475707	0.0455887	2.60E-25	7213
GFRAL	rs2270989	6	54840713	G	A	0.304104	-0.0990065	0.0178529	3.03E-08	7213
GFRAL	rs73439277	6	55393672	G	A	0.140233	0.29143	0.0237795	3.43E-34	7213
GFRAL	rs9396134	6	55689525	C	G	0.0808263	0.256093	0.03	1.67E-17	7213
PLOD2	rs16857612	3	145939653	G	A	0.192569	0.124749	0.0210632	3.31E-09	7213
SNX8	rs28514741	7	2249281	T	C	0.0111604	-0.908082	0.0774769	1.92E-31	7213
SNX8	rs34519622	7	2314707	A	G	0.158048	0.170261	0.0227327	7.72E-14	7213
NDST1	rs2569103	5	150422548	A	G	0.0711216	-0.259373	0.032292	1.11E-15	7213
NDST1	rs11746198	5	150543647	C	T	0.403577	0.228075	0.0167665	1.25E-41	7213
ST8SIA6	rs11254541	10	17348944	G	A	0.188895	0.14004	0.0211	3.43E-11	7213
ST8SIA6	rs11595070	10	17448760	T	C	0.0355608	-0.371752	0.0446541	9.98E-17	7213
ST3GAL6	rs138585571	3	98469736	C	T	0.0359074	-0.29163	0.0443657	5.26E-11	7213
ST3GAL6	rs114965972	3	98735036	G	A	0.0134479	0.423498	0.0718335	3.90E-09	7213
ST3GAL6	rs278365	3	98767147	A	G	0.0389574	0.387548	0.0424557	8.89E-20	7213
ST3GAL6	rs72934641	3	98801495	G	A	0.0455428	-1.6391	0.034946	0	7213
ST3GAL6	rs35644578	3	99045128	C	T	0.0718148	0.796545	0.0308532	1.19E-140	7213
SNX1	rs138194169	15	64107669	A	C	0.021489	0.679498	0.0567159	9.16E-33	7213
CNTNAP2	rs850493	7	145616845	G	A	0.274782	0.703988	0.0165634	0	7213
CNTNAP2	rs2462603	7	146116762	G	A	0.499376	0.205417	0.0165737	6.37E-35	7213
WFDC5	rs6124684	20	45092852	C	T	0.283516	-0.133065	0.0184199	5.57E-13	7213
IGSF8	rs12401728	1	160092169	C	T	0.217247	0.293985	0.0200166	3.85E-48	7213
HS3ST3B1	rs62056073	17	14345059	A	G	0.0218356	-0.464273	0.0565896	2.73E-16	7213
ASPH	rs11775077	8	61685128	G	A	0.177319	-0.331558	0.0216419	3.69E-52	7213
CD72	rs75679360	9	35563364	G	A	0.0516429	0.526913	0.0373286	1.19E-44	7213
LILRB5	rs17841903	19	54230970	G	T	0.276029	-0.23696	0.0183815	1.31E-37	7213
LILRB5	rs12976868	19	54252060	A	G	0.239567	-0.725221	0.0175493	0	7213

LILRB5	rs527420832	19	54296769	T	C	0.0172605	0.534917	0.0641728	9.15E-17	7213
SEMA7A	rs78994380	15	74400071	C	A	0.113268	-0.179892	0.0260272	5.20E-12	7213
ADAM23	rs114956296	2	206103006	G	A	0.0346596	-0.4768	0.0454147	1.34E-25	7213
ADAM23	rs115234016	2	206412331	A	T	0.147858	0.181885	0.0235452	1.27E-14	7213
ADAM23	rs1448903	2	206444237	A	G	0.0899764	0.857603	0.0272122	2.36E-204	7213
ADAM23	rs12472480	2	206533833	C	A	0.369056	-0.426872	0.0164077	1.05E-142	7213
NEGR1	rs78738825	1	71587342	C	T	0.0435325	-0.274298	0.0406428	1.60E-11	7213
NEGR1	rs10493488	1	71927102	C	G	0.139748	-0.164655	0.0239133	6.24E-12	7213
LILRA6	rs289177	19	54196064	A	G	0.0797865	0.31252	0.0305844	2.41E-24	7213
LILRA6	rs34810796	19	54244555	T	C	0.0884514	1.07816	0.0260765	0	7213
BTN3A1	rs3884025	6	25929860	G	A	0.0703591	-0.218309	0.0325048	2.01E-11	7213
MATN4	rs17322289	20	44971310	T	C	0.119714	0.183292	0.0255909	8.71E-13	7213
MATN4	rs11906694	20	45299492	C	T	0.249965	-0.3569	0.0187716	1.10E-78	7213
RMDN1	rs112418766	8	86009897	C	T	0.0129627	0.495418	0.0722676	7.70E-12	7213
RMDN1	rs79995713	8	86122166	C	G	0.0726466	0.24441	0.0318112	1.76E-14	7213
RMDN1	rs74817514	8	86426137	G	A	0.0290448	0.524615	0.0485882	5.70E-27	7213
RMDN1	rs10100247	8	86519816	C	T	0.279981	-0.871536	0.0155041	0	7213
RMDN1	rs17683923	8	86618352	A	G	0.0306391	0.442479	0.0485687	1.05E-19	7213
DNAJB11	rs56227532	3	186576030	T	C	0.0766671	-0.570804	0.030505	2.49E-76	7213
VWA2	rs35060624	10	114139781	C	T	0.0811729	0.463603	0.0298303	1.34E-53	7213
VWA2	rs67035778	10	114283667	T	A	0.0657147	-0.354665	0.0331118	1.43E-26	7213
CELA2A	rs2473355	1	15423721	A	G	0.38389	-0.112078	0.0169899	4.50E-11	7213
MGAT4B	rs113756550	5	179801377	G	A	0.0206571	-1.57189	0.0557478	5.07E-166	7213
ITIH3	rs2710331	3	52803839	T	C	0.357272	0.66869	0.0154933	0	7213
ITIH3	rs187933377	3	52814850	C	T	0.0121309	-1.63674	0.0735834	4.53E-106	7213
ITIH3	rs11716612	3	52910629	C	T	0.12623	0.140159	0.0251358	2.55E-08	7213
FUT10	rs76324030	8	33032814	G	A	0.0308471	0.412652	0.0476586	5.83E-18	7213
FUT10	rs146410548	8	33033969	G	A	0.0259254	0.392958	0.0521541	5.50E-14	7213
FUT10	rs7817580	8	33168153	A	T	0.0317482	0.366979	0.0469885	6.53E-15	7213
FUT10	rs75200062	8	33204003	T	A	0.0151809	-0.369459	0.0675192	4.60E-08	7213
FUT10	rs117419172	8	33348807	G	A	0.0183696	0.814924	0.0615894	1.66E-39	7213
FUT10	rs16880907	8	33411137	G	C	0.0116456	-0.859095	0.0769152	9.90E-29	7213
FUT10	rs28558391	8	33438597	G	A	0.402953	0.751327	0.0142793	0	7213
H6PD	rs34603401	1	9245386	A	C	0.15278	0.712904	0.0214457	1.42E-225	7213
H6PD	rs916379	1	9281069	C	T	0.414876	-0.196966	0.016742	1.15E-31	7213
TMEM132C	rs9669390	12	128277877	C	T	0.349577	-0.32637	0.0170527	1.11E-79	7213
TMEM132C	rs1518370	12	128418024	C	T	0.458062	-0.195954	0.0165065	3.32E-32	7213
TMEM132C	rs66805629	12	128587627	T	C	0.1568	-0.124299	0.0226362	4.13E-08	7213
NFASC	rs2093539	1	204816087	C	T	0.0898378	0.497329	0.0285455	1.27E-66	7213
NFASC	rs6663324	1	204977636	T	C	0.106474	0.823901	0.0251358	8.72E-220	7213
NFASC	rs7533611	1	205028800	A	G	0.0419382	0.253894	0.0415408	1.04E-09	7213
GP5	rs1466733	3	194400269	A	G	0.256689	-0.265821	0.0188276	1.14E-44	7213
IFNLR1	rs75884190	1	24165968	A	G	0.223347	-0.128953	0.0198551	8.87E-11	7213

IFNLR1	rs139958347	1	24187233	G	C	0.0368085	-0.907799	0.0426988	2.44E-97	7213
FAM20B	rs12568291	1	179017420	G	A	0.370512	0.166602	0.017229	5.51E-22	7213
FBP1	rs79813405	9	94589267	C	T	0.0960072	0.183357	0.0283434	1.05E-10	7213
FBP1	rs2987899	9	94596959	A	G	0.0650908	-0.461261	0.0333641	6.33E-43	7213
MGAT4C	rs61929372	12	85923916	A	G	0.109178	0.237014	0.0267574	1.01E-18	7213
APLP1	rs58291714	19	35891884	T	C	0.0328573	0.320672	0.0461173	3.88E-12	7213
RNASE1	rs17254387	14	20812519	G	A	0.313392	-0.423488	0.0172462	6.06E-128	7213
RNASE1	rs56216219	14	20819064	A	G	0.0346596	-0.768045	0.0447602	1.02E-64	7213
ATP1B2	rs1642762	17	7651454	C	T	0.430958	0.431073	0.0160323	7.68E-152	7213
ATP1B2	rs1050541	17	7657517	T	G	0.459032	0.214355	0.0166164	1.17E-37	7213
COCH	rs141551312	14	30728783	T	A	0.0173991	0.361124	0.0640921	1.82E-08	7213
COCH	rs12888555	14	30777652	C	T	0.347775	-0.136795	0.0175797	8.17E-15	7213
COCH	rs28400019	14	30874288	G	A	0.118328	0.711235	0.0240692	3.00E-181	7213
COCH	rs1108282	14	30884041	A	C	0.210176	-0.209045	0.0202697	9.12E-25	7213
IGFLR1	rs148381377	19	35506479	G	A	0.0136559	-0.651786	0.0714394	9.27E-20	7213
IGFLR1	rs11549030	19	35755517	C	G	0.151671	-0.996974	0.0203398	0	7213
IGFLR1	rs139141772	19	35838764	T	C	0.0671704	-0.217989	0.0332117	5.62E-11	7213
FREM2	rs9603416	13	38755978	T	A	0.305421	0.155925	0.01782	2.63E-18	7213
C1QTNF3	rs840390	5	34018518	G	A	0.129419	-0.213205	0.0247919	9.69E-18	7213
SERPINA9	rs11160181	14	94461852	G	T	0.418342	-0.193143	0.0166451	7.42E-31	7213
SERPINA9	rs1998207	14	94491377	G	T	0.288368	-0.132821	0.018063	2.15E-13	7213
LRRC32	rs12275758	11	76638585	G	A	0.0762512	-0.172226	0.0313585	4.11E-08	7213
CRELD1	rs41277465	3	9683316	T	A	0.0214197	0.451858	0.057307	3.61E-15	7213
CRELD1	rs111239586	3	9861067	T	A	0.155968	0.152714	0.0229871	3.29E-11	7213
CRELD1	rs35741213	3	10045977	A	G	0.162415	-0.857489	0.0201869	0	7213
CRELD1	rs34810856	3	10078244	A	G	0.0293221	0.516569	0.0488669	6.29E-26	7213
CRELD1	rs370230	3	10259257	A	G	0.0266879	-0.599372	0.050769	7.15E-32	7213
MYBPC1	rs80087033	12	101654669	G	A	0.1568	-0.202723	0.0226046	3.79E-19	7213
NPPB	rs198379	1	11855410	T	C	0.424026	0.204607	0.0168613	1.47E-33	7213
HTATIP2	rs7103934	11	20368197	T	A	0.34008	-0.117003	0.0174584	2.21E-11	7213
CHKB	rs5770922	22	50584979	A	G	0.0484542	-0.503567	0.038365	6.54E-39	7213
SH3BP2	rs7695151	4	2770803	C	T	0.467628	0.176218	0.0164943	1.92E-26	7213
NFU1	rs74637005	2	69423598	G	A	0.037779	-0.302771	0.0435373	3.85E-12	7213
CHST11	rs1704878	12	104577480	G	A	0.295716	-0.574916	0.0170273	3.75E-232	7213
CHST11	rs1353191	12	104771121	C	T	0.313878	0.145453	0.0179405	6.02E-16	7213
KNG1	rs5030044	3	186731333	A	G	0.109247	0.506048	0.0262686	1.11E-80	7213
KNG1	rs76438938	3	186743735	C	T	0.0273118	-1.51067	0.047643	1.11E-206	7213
LILRA5	rs397600	19	54287934	G	A	0.344586	-0.239724	0.0173604	7.90E-43	7213
CHST1	rs753198	11	45733411	T	C	0.282337	-0.109412	0.0184852	3.39E-09	7213
B4GALT7	rs112824307	5	177588250	T	A	0.0417995	0.473376	0.0413344	4.16E-30	7213
GLCE	rs11635000	15	69170334	T	A	0.21995	-0.774062	0.0179457	0	7213
GLCE	rs148769347	15	69450333	G	A	0.0512963	-0.240906	0.037518	1.44E-10	7213
C1QTNF5	rs9640	11	119339269	T	A	0.12318	0.273382	0.0250648	1.75E-27	7213

C1QTNF5	rs587985	11	119373385	C	T	0.390337	0.132684	0.0170017	6.84E-15	7213
ALPP	rs11681585	2	232315500	A	G	0.120477	-0.246873	0.0253168	2.50E-22	7213
ALPP	rs35458538	2	232439642	G	A	0.23728	0.393209	0.019004	1.95E-92	7213
ESAM	rs12541	11	124753596	T	C	0.227506	-0.392821	0.0192003	1.78E-90	7213
QPCT	rs4648158	2	37148175	T	C	0.044503	0.329025	0.0403322	3.99E-16	7213
QPCT	rs75573663	2	37345976	T	C	0.0106059	-1.2178	0.0793432	2.42E-52	7213
QPCT	rs72792854	2	37365901	G	A	0.0612782	0.309755	0.0341452	1.49E-19	7213
MTIF3	rs1218825	13	27435783	G	C	0.0468598	-0.312736	0.0393278	2.11E-15	7213
FAM151A	rs17393916	1	54549812	T	A	0.164356	-0.171356	0.0221773	1.25E-14	7213
FAM151A	rs2060385	1	54623799	A	T	0.343616	0.302959	0.0170118	1.83E-69	7213
ROR2	rs4604505	9	91740556	G	C	0.415638	0.227109	0.0165902	3.94E-42	7213
ROR2	rs10118816	9	91929196	C	T	0.327118	0.34275	0.017433	7.11E-84	7213
DNAJC30	rs9647712	7	73682207	C	A	0.0351449	1.04623	0.0437737	1.45E-121	7213
TMEM132A	rs41504746	11	60854384	T	G	0.0300153	-0.999532	0.0478898	5.59E-94	7213
TMEM132A	rs1789780	11	60859758	G	A	0.236171	0.730677	0.0177039	0	7213
TMEM132A	rs175132	11	61069202	C	G	0.163178	0.133842	0.0224715	2.71E-09	7213
PLEK	rs1867312	2	68392849	C	A	0.432414	-0.443531	0.0160892	3.92E-159	7213
PLEK	rs7570797	2	68645939	A	G	0.0472758	-0.281332	0.0392109	7.96E-13	7213
UGT1A6	rs887829	2	233759924	C	T	0.329821	-0.378993	0.0172179	5.58E-104	7213
HP	rs4788817	16	71607492	T	C	0.35942	-0.142542	0.0173286	2.28E-16	7213
HP	rs34042070	16	72067626	C	G	0.197144	-0.547949	0.0198295	7.53E-160	7213
HP	rs7202323	16	72183214	T	G	0.237765	0.330837	0.0191439	1.34E-65	7213
S100A7	rs3014837	1	153458930	C	G	0.0595453	1.48186	0.0303707	0	7213
AMY1A	rs150076551	1	103534341	C	T	0.0208651	0.409885	0.057867	1.54E-12	7213
AMY1A	rs78811372	1	103579772	G	A	0.0396506	-0.712255	0.0421722	8.61E-63	7213
AMY1A	rs114922930	1	103588835	C	T	0.0107445	-0.635002	0.0797818	2.00E-15	7213
AMY1A	rs74486377	1	103780382	A	C	0.0122695	0.581554	0.0753399	1.33E-14	7213
AMY1A	rs61816500	1	103963556	A	G	0.0469292	-0.320957	0.0394803	5.03E-16	7213
AMY1A	rs72683765	1	104021219	C	G	0.0320255	-0.306793	0.0470752	7.65E-11	7213
FJX1	rs474415	11	35621175	G	A	0.321503	0.124433	0.0175971	1.68E-12	7213
FJX1	rs10768174	11	36057706	G	A	0.431721	0.273935	0.0163845	1.36E-61	7213
FJX1	rs10501144	11	36118384	T	C	0.326078	0.108923	0.0177045	8.05E-10	7213
SPINT3	rs6073773	20	45519311	G	C	0.472758	-0.409398	0.0160488	1.67E-137	7213
TPST1	rs578114150	7	65779453	G	A	0.0170525	0.489631	0.0643301	3.06E-14	7213
TPST1	rs313829	7	66087510	A	G	0.309303	-0.372838	0.0174782	5.86E-98	7213
TPST1	rs778714	7	66384415	A	T	0.150423	-0.217583	0.0232742	1.16E-20	7213
ADAM22	rs2279542	7	87935182	C	G	0.487176	0.446487	0.0157528	1.20E-167	7213
ADAM22	rs73202393	7	88110717	A	C	0.0844309	-0.214977	0.029709	5.09E-13	7213
SEMA6A	rs17139604	5	116371615	A	G	0.0511576	-0.406644	0.0378005	8.71E-27	7213
SEMA6A	rs3733724	5	116444277	C	T	0.0849854	0.385829	0.0295859	1.94E-38	7213
SEMA6A	rs34965	5	116469633	C	T	0.0269652	-0.318088	0.0504857	3.14E-10	7213
SEMA6A	rs633654	5	116614393	C	A	0.188133	-0.115544	0.0211525	4.85E-08	7213
GLTPD2	rs138959040	17	4597092	G	A	0.0122695	-0.47551	0.075443	3.09E-10	7213

GLTPD2	rs34460487	17	4781933	G	A	0.341883	0.284825	0.0171163	4.82E-61	7213
SLAMF1	rs6704124	1	160632458	C	T	0.331901	0.116482	0.0176157	4.05E-11	7213
ITIH1	rs2535629	3	52799203	G	A	0.344517	-0.647941	0.0157859	0	7213
SCG3	rs2606139	15	51674514	A	G	0.234299	-0.806803	0.0170823	0	7213
CRTAM	rs10790526	11	122757323	T	C	0.445307	0.09376	0.0165786	1.61E-08	7213
CRTAM	rs2370794	11	122844074	A	G	0.327118	0.307747	0.0175703	2.68E-67	7213
ART3	rs4859610	4	76079288	A	G	0.225912	0.397073	0.0191334	5.77E-93	7213
ART3	rs10009108	4	76106512	C	A	0.395952	0.129041	0.0169568	3.09E-14	7213
B3GNT2	rs80161321	2	62186659	C	T	0.0143491	-0.525274	0.0698873	6.33E-14	7213
B3GNT2	rs34361686	2	62405057	G	A	0.479897	0.234086	0.0165559	8.62E-45	7213
ERO1LB	rs2463201	1	236242197	T	C	0.406558	0.328498	0.0164158	9.66E-87	7213
ENTPD1	rs4512761	10	95865342	G	A	0.340774	0.292389	0.017236	2.54E-63	7213
MXRA7	rs146105750	17	76568973	C	T	0.0153196	0.42212	0.068126	6.10E-10	7213
MXRA7	rs4789345	17	76692234	C	T	0.369264	0.496034	0.0161882	6.04E-194	7213
DNAJB12	rs9415063	10	72326894	C	T	0.357202	0.199928	0.0171603	4.32E-31	7213
CTSB	rs709821	8	11845085	G	C	0.259185	0.73774	0.0170299	0	7213
CTSB	rs4841602	8	11871913	A	G	0.102454	-0.366993	0.0270805	2.47E-41	7213
LMAN2L	rs58361269	2	96737373	G	A	0.335228	0.248066	0.0174316	2.41E-45	7213
MANEA	rs2380214	6	95508738	C	T	0.108069	-1.04012	0.0235344	0	7213
MANEA	rs144362594	6	95567940	G	A	0.0370858	0.237819	0.0434478	4.56E-08	7213
MANEA	rs147307634	6	95644258	A	T	0.0226674	0.315746	0.055926	1.71E-08	7213
MANEA	rs55708294	6	95917671	T	C	0.0352142	-0.427513	0.0450602	3.14E-21	7213
MANEA	rs6926151	6	96028152	A	G	0.0668238	0.218621	0.033242	5.15E-11	7213
TPST2	rs4275	22	26525759	A	G	0.424581	0.268985	0.0166545	1.14E-57	7213
TPST2	rs2071862	22	26625437	G	A	0.140233	-0.17591	0.0239225	2.15E-13	7213
SPINK5	rs17599675	5	148158915	T	C	0.259531	0.181518	0.0186031	2.36E-22	7213
FAM177A1	rs1210973	14	34898055	C	T	0.223416	-0.158275	0.0198731	1.92E-15	7213
FAM177A1	rs10151531	14	34923843	T	C	0.153473	0.861946	0.0208802	0	7213
FAM177A1	rs77528002	14	35169548	G	A	0.156939	0.141672	0.0227594	5.09E-10	7213
COMP	rs12974746	19	18795739	A	G	0.0256481	-0.698886	0.0521191	1.62E-40	7213
PATE4	rs7939499	11	125748836	T	C	0.468252	0.109895	0.0166198	4.05E-11	7213
PATE4	rs665677	11	125882266	A	G	0.29225	0.277842	0.0179524	3.55E-53	7213
LIPN	rs139794964	10	88546049	G	T	0.018023	-0.377485	0.0627446	1.87E-09	7213
LIPN	rs391683	10	88760926	A	G	0.322612	0.666425	0.016007	0	7213
LIPN	rs61854005	10	88767075	G	C	0.0302925	-0.32206	0.0480348	2.17E-11	7213
LIPN	rs7909575	10	88772259	T	C	0.0158048	0.681406	0.0664989	1.80E-24	7213
SPON2	rs3736084	4	883837	G	A	0.404339	0.109983	0.0168015	6.31E-11	7213
SPON2	rs2279279	4	1170489	C	G	0.334258	0.584408	0.0160884	1.79E-265	7213
SPON2	rs73793112	4	1204810	A	C	0.0282129	0.317444	0.0498469	2.03E-10	7213
MIF	rs2330634	22	23908608	C	G	0.402745	0.274135	0.0166472	7.72E-60	7213
EPHB2	rs2043970	1	22735465	A	C	0.0802717	-0.8304	0.0290775	4.88E-170	7213
EPHB2	rs6686906	1	22829631	G	A	0.354637	0.156922	0.0171451	7.12E-20	7213
GXYLT1	rs10880228	12	42048650	G	A	0.152988	-0.325103	0.0229161	4.46E-45	7213

GXYLT1	rs190407775	12	42126028	A	G	0.0171912	1.4109	0.0613434	5.03E-113	7213
ITIH5	rs7897049	10	7571512	G	A	0.0469292	-0.444479	0.0388802	5.24E-30	7213
ITIH5	rs77377944	10	7575484	C	T	0.0196174	0.342333	0.0596232	9.76E-09	7213
ITIH5	rs11255234	10	7607656	A	G	0.27395	-0.333597	0.0183355	2.35E-72	7213
ITIH5	rs6602258	10	7656775	G	A	0.252322	-0.427218	0.0183495	1.14E-115	7213
CHGB	rs36054946	20	5886044	T	C	0.0177457	-0.410316	0.0631849	8.92E-11	7213
SPINK1	rs6580502	5	147828480	T	C	0.396645	0.16339	0.0167536	2.47E-22	7213
FUT8	rs148659526	14	65277722	C	T	0.0341051	-0.53246	0.0453724	1.62E-31	7213
FUT8	rs78461609	14	65306742	A	C	0.0407597	-0.23172	0.0423097	4.48E-08	7213
FUT8	rs8020305	14	65370785	C	T	0.0607237	0.728754	0.03372	2.02E-100	7213
ICAM5	rs281440	19	10289628	G	A	0.234161	-0.787543	0.0175097	0	7213
ICAM5	rs55898302	19	10463579	T	C	0.164148	0.16345	0.0225551	4.71E-13	7213
ICAM5	rs117707627	19	10615630	A	G	0.046028	-0.471954	0.0399342	6.18E-32	7213
SIGLEC14	rs62115071	19	51481402	G	A	0.182171	-0.151742	0.0216995	2.93E-12	7213
SIGLEC14	rs1106476	19	51627384	T	A	0.119506	-1.00953	0.0227222	0	7213
SIGLEC14	rs4802892	19	52024891	A	G	0.348745	-0.0978146	0.0173501	1.79E-08	7213
PTPRJ	rs1566734	11	48123823	A	C	0.15992	0.232257	0.0224332	6.04E-25	7213
NOTUM	rs111343749	17	81966908	G	A	0.0440871	-0.267293	0.04036	3.78E-11	7213
MRVI1	rs1075768	11	10678202	A	G	0.397477	0.214955	0.0169081	1.23E-36	7213
MRVI1	rs12792130	11	10724382	T	C	0.032996	-0.312888	0.0461379	1.28E-11	7213
UXS1	rs3888361	2	106173309	C	G	0.123388	0.358402	0.024887	2.23E-46	7213
TMPO	rs61932018	12	98573827	T	G	0.0447803	-0.230861	0.0401204	9.06E-09	7213
HS3ST3A1	rs139707331	17	13657100	G	A	0.0140025	0.419599	0.0708177	3.27E-09	7213
ARSK	rs956274	5	95611429	G	T	0.362055	-0.133637	0.017117	6.67E-15	7213
STX7	rs3813356	6	132513379	C	T	0.440871	-0.222839	0.0166116	1.51E-40	7213
PEAR1	rs12041331	1	156899922	G	A	0.0863025	-0.541775	0.0289941	3.98E-76	7213
PEAR1	rs12137505	1	156913754	A	G	0.39713	-0.244259	0.0167549	1.82E-47	7213
APOH	rs140526702	17	66150520	C	T	0.0431166	0.475676	0.0406944	2.78E-31	7213
APOH	rs1801689	17	66214462	A	C	0.032788	-1.53413	0.0433056	1.42E-253	7213
APOH	rs114844906	17	66349902	G	A	0.0149036	-0.406302	0.0687281	3.54E-09	7213
GPNMB	rs74937181	7	23180927	A	G	0.0276584	-0.364635	0.0502537	4.41E-13	7213
GPNMB	rs28458177	7	23255253	G	A	0.379315	-0.30341	0.0168612	7.49E-71	7213
KDELC2	rs141379009	11	108278480	T	G	0.0294607	-1.38995	0.0460827	2.53E-188	7213
KDELC2	rs4278482	11	108551662	G	A	0.285595	-0.174545	0.018123	7.99E-22	7213
DNAJC10	rs288334	2	182757820	T	G	0.364966	-0.211479	0.0171142	1.00E-34	7213
LILRA4	rs12976217	19	54338192	C	T	0.356162	0.4063	0.0168104	3.64E-124	7213
LILRA4	rs2004431	19	54341339	T	G	0.175516	-0.14102	0.0217579	9.69E-11	7213
TNFRSF11B	rs12156430	8	118430658	A	C	0.347775	-0.114765	0.0174114	4.66E-11	7213
TNFRSF11B	rs2468184	8	119185624	A	G	0.414876	-0.141371	0.0167859	4.42E-17	7213
C11orf68	rs554169857	11	65919002	C	A	0.0186469	0.924467	0.0604472	5.47E-52	7213
HYAL1	rs116482870	3	50302191	C	T	0.0656454	-0.362857	0.033573	5.08E-27	7213
ADH4	rs6858148	4	99144766	T	C	0.364758	-0.431801	0.0165317	9.01E-144	7213
DPEP2	rs76359116	16	67963800	G	C	0.041245	-0.603741	0.0414212	1.89E-47	7213

DPEP2	rs73615919	16	68364959	C	T	0.122626	0.211337	0.0250999	4.50E-17	7213
GRAMD1C	rs9883363	3	113829383	G	A	0.241231	-0.105974	0.01929	4.07E-08	7213
GRAMD1C	rs61634901	3	113907112	G	A	0.331831	0.336878	0.0172682	1.22E-82	7213
PTPRU	rs2179795	1	29315806	G	T	0.282407	-0.253223	0.0182568	3.44E-43	7213
PTPRU	rs9426365	1	29470846	C	A	0.0768751	-0.181306	0.0311932	6.42E-09	7213
GPX7	rs6669154	1	52609880	A	C	0.167961	0.719894	0.0206675	7.73E-246	7213
DPP7	rs4880198	9	137084985	C	T	0.221822	-0.523638	0.0190956	1.46E-157	7213
PRSS57	rs2301741	19	675106	C	T	0.491751	-0.332643	0.0161556	1.40E-91	7213
OXT	rs6084206	20	2937674	A	G	0.43484	0.148765	0.0167132	6.91E-19	7213
OXT	rs67110989	20	3054497	A	G	0.483294	-0.155939	0.016592	7.29E-21	7213
OXT	rs2875830	20	3073387	C	T	0.292666	-0.368173	0.017465	8.88E-96	7213
PRDX3	rs1810159	10	119195930	A	C	0.117427	0.168914	0.0256845	5.15E-11	7213
NCR1	rs73619967	19	54912120	T	C	0.140718	-0.191337	0.0240388	1.99E-15	7213
NCR1	rs2915993	19	54921974	G	A	0.385138	-0.17854	0.0169101	7.19E-26	7213
UST	rs6926406	6	148598424	T	A	0.324345	0.130085	0.0175308	1.30E-13	7213
UST	rs7764228	6	148737672	A	C	0.446278	0.15576	0.0167134	1.52E-20	7213
TNFRSF1B	rs519064	1	12182039	C	T	0.0334812	-1.14125	0.044081	2.14E-141	7213
TNFRSF1B	rs4845897	1	12185675	T	A	0.159781	-0.304256	0.0225795	6.85E-41	7213
LHB	rs3795050	19	49017606	A	C	0.0775683	-0.546216	0.0303364	6.22E-71	7213
RNASE2	rs2233859	14	20891649	C	A	0.426244	0.304277	0.0162001	7.03E-77	7213
QSOX2	rs10858248	9	136216478	A	G	0.495979	-0.456393	0.0159071	1.59E-171	7213
QSOX2	rs67827752	9	136245358	G	A	0.336129	-0.141728	0.0177544	1.65E-15	7213
RARRES1	rs4680458	3	158724365	A	G	0.292805	0.652282	0.0165104	3.45961e-309	7213
RARRES1	rs1604644	3	158843617	A	G	0.284001	-0.114554	0.0183146	4.21E-10	7213
FASN	rs62078746	17	82095714	G	A	0.456814	0.179629	0.0163781	9.06E-28	7213
RSPO3	rs853974	6	126747838	T	C	0.270415	0.140848	0.0186566	4.91E-14	7213
RSPO3	rs1892172	6	127155371	G	A	0.462498	-0.492365	0.0153929	4.64E-210	7213
NTM	rs12800878	11	131246794	G	A	0.385138	-0.366275	0.0166356	4.93E-104	7213
NTM	rs2511504	11	131326501	T	C	0.427839	0.617461	0.0152104	0	7213
RSPO4	rs75904281	20	963194	C	T	0.0146264	-0.685955	0.0693884	6.71E-23	7213
RSPO4	rs879012	20	1029145	C	T	0.315403	0.143471	0.0176435	4.94E-16	7213
CTSH	rs34843303	15	78942128	T	C	0.108831	-1.22849	0.0222149	0	7213
CTSH	rs62013235	15	78953824	G	A	0.0956606	0.330086	0.0279643	7.32E-32	7213
CTSH	rs143141218	15	78968399	G	C	0.0232913	-0.364332	0.0551766	4.32E-11	7213
IGFBP2	rs4674091	2	216535725	C	T	0.420421	-0.104725	0.0167362	4.14E-10	7213
CHGA	rs729940	14	92932756	C	T	0.145155	-0.326871	0.0232347	2.29E-44	7213
MMP10	rs11225354	11	102639315	T	C	0.126577	-0.29893	0.0246845	1.97E-33	7213
MMP10	rs17860955	11	102778751	T	C	0.0173991	-1.07655	0.0624352	2.56E-65	7213
MMP10	rs3025066	11	102839752	T	C	0.0697352	0.271085	0.0323193	5.91E-17	7213
EFEMP1	rs145713418	2	55738460	C	A	0.0223901	-0.319513	0.0560683	1.26E-08	7213
EFEMP1	rs3791679	2	55869757	A	G	0.240191	0.427352	0.0189252	3.78E-109	7213
GPNMB	rs858285	7	23209899	C	T	0.0399279	-0.416887	0.0420383	4.94E-23	7213
GPNMB	rs2268748	7	23273552	T	C	0.0406904	0.683774	0.0411988	9.72E-61	7213

OLFML3	rs3811018	1	113970317	A	T	0.301816	-0.173273	0.0180727	1.21E-21	7213
PKDCC	rs2424	2	42058435	T	A	0.217801	0.38507	0.0196436	2.11E-83	7213
PKDCC	rs7582283	2	42068308	G	A	0.0400665	0.491391	0.0420929	3.30E-31	7213
TMEM106B	rs5011432	7	12229042	A	C	0.409746	-0.25715	0.0165455	1.33E-53	7213
GPC1	rs1126920	2	240466062	C	T	0.110218	0.799601	0.0248492	2.13E-212	7213
A4GALT	rs2413707	22	42645483	G	A	0.345279	0.0974933	0.0172995	1.81E-08	7213
A4GALT	rs8138197	22	42718545	G	A	0.4817	-0.372678	0.0161511	1.02E-113	7213
LILRA5	rs397600	19	54287934	G	A	0.344586	-0.275889	0.0172858	2.21E-56	7213
EMILIN3	rs61739314	20	41361737	G	C	0.0323721	-1.02302	0.0454153	1.21E-108	7213
EMILIN3	rs6029662	20	41407325	G	C	0.211978	0.304156	0.0201713	1.32E-50	7213
NOG	rs227715	17	56705153	A	G	0.232843	-0.17296	0.0195899	1.31E-18	7213
FAM171B	rs12612335	2	186701929	T	A	0.183003	-0.264785	0.021176	1.65E-35	7213
DPEP1	rs409170	16	89615942	A	G	0.40025	-0.526387	0.0157061	5.34E-229	7213
DPEP1	rs62054658	16	90074286	A	G	0.061001	-0.355402	0.0350847	5.89E-24	7213
TFRC	rs62282694	3	196109704	T	C	0.245598	-0.111702	0.0192796	7.17E-09	7213
PSAPL1	rs35791045	4	7425380	A	G	0.170872	0.578542	0.0209331	6.70E-160	7213
PSAPL1	rs62277606	4	7445534	G	A	0.433038	-0.123339	0.0168208	2.50E-13	7213
C1S	rs146111161	12	6800807	C	T	0.0176071	-0.364701	0.0637218	1.09E-08	7213
CILP2	rs4808205	19	19534684	T	G	0.165049	0.184904	0.0224024	1.81E-16	7213
GRAMD1C	rs9990079	3	113838843	G	A	0.24137	-0.127949	0.0192713	3.38E-11	7213
GRAMD1C	rs61634901	3	113907112	G	A	0.331831	0.575464	0.0163708	5.39E-250	7213
CA11	rs62130341	19	48675495	C	T	0.100929	0.167622	0.0271784	7.31E-10	7213
QPCTL	rs143356508	19	45545009	A	G	0.064259	-0.194922	0.0338597	8.93E-09	7213
QPCTL	rs17850756	19	45703004	G	A	0.322057	-0.47655	0.016974	1.09E-164	7213
CLN5	rs17067027	13	76965650	C	T	0.0258561	-0.525036	0.0514864	2.97E-24	7213
CLN5	rs9573988	13	77029428	G	A	0.0641897	-0.282629	0.0339469	9.95E-17	7213
CACNA2D3	rs9882539	3	54129177	C	G	0.401151	0.273787	0.0164143	2.57E-61	7213
CACNA2D3	rs74943810	3	54603265	C	T	0.041245	0.251084	0.0417747	1.94E-09	7213
TMEM132B	rs2007225	12	125328341	T	C	0.370165	0.104475	0.0170349	9.08E-10	7213
TMEM132B	rs3825381	12	125652317	C	T	0.256828	-0.327018	0.018609	1.01E-67	7213
PEAR1	rs12041331	1	156899922	G	A	0.0863025	-0.233399	0.0295603	3.31E-15	7213
HNRNPAB	rs72648830	5	178181541	C	T	0.122834	0.170735	0.025195	1.33E-11	7213
NEO1	rs13379875	15	73050440	T	C	0.411063	-0.352341	0.0165059	4.35E-98	7213
STIM1	rs1869085	11	3883134	A	C	0.30757	-0.122929	0.0179342	7.75E-12	7213
GALNT16	rs11621049	14	69279532	G	A	0.467836	-0.162949	0.016608	1.39E-22	7213
GALNT16	rs34996930	14	69325779	C	T	0.0996811	-0.154095	0.0275493	2.31E-08	7213
GALNT16	rs12100668	14	69326758	G	A	0.399972	-0.314204	0.0165682	2.65E-78	7213
RRM2B	rs11785514	8	102191915	A	C	0.0685568	0.411628	0.0324345	1.63E-36	7213
ENTPD6	rs6050446	20	25214873	A	G	0.028005	-0.612944	0.0500189	3.48E-34	7213
HDGF	rs4399146	1	156743766	G	A	0.346596	-0.628061	0.015795	8.29703000000813e-313	7213
SCARF2	rs5763025	22	20432201	A	C	0.195758	-0.362552	0.0205814	4.91E-68	7213
ERLEC1	rs2692523	2	53823074	C	T	0.175031	-0.247753	0.0217208	7.01E-30	7213
CHL1	rs990284	3	63289	A	G	0.374879	-0.306673	0.0169679	1.91E-71	7213

CHL1	rs13061475	3	229245	G	C	0.154998	0.223276	0.0229894	3.66E-22	7213
CHL1	rs143330129	3	383600	A	G	0.0142105	-0.447658	0.0702912	2.02E-10	7213
ERAP2	rs156015	5	96495489	G	A	0.311867	0.154112	0.0180036	1.36E-17	7213
ERAP2	rs34847574	5	96704933	G	A	0.350478	-0.180947	0.0171626	8.41E-26	7213
ERAP2	rs12655342	5	96893838	T	G	0.146541	-0.729165	0.021858	4.86E-227	7213
ERAP2	rs1230381	5	96918505	A	G	0.486067	0.682887	0.0145168	0	7213
ERAP2	rs1593042	5	97258374	C	T	0.352003	-0.111148	0.0174693	2.11E-10	7213
VTI1B	rs10483801	14	67650289	C	A	0.185221	0.167751	0.0211454	2.46E-15	7213
CD14	rs114928388	5	140383553	G	A	0.0233606	0.311275	0.0553148	1.90E-08	7213
CD14	rs5744441	5	140637262	G	A	0.237627	-0.308749	0.0188984	6.07E-59	7213
RIPK2	rs34211510	8	89771518	C	T	0.0804104	0.18844	0.0303209	5.42E-10	7213
FCRL4	rs11582663	1	157589332	C	T	0.159088	-1.00443	0.0197164	0	7213
FCRL4	rs111628484	1	157903343	G	A	0.0241924	0.307243	0.0545764	1.87E-08	7213
COL15A1	rs71501884	9	98941784	T	C	0.260641	0.209968	0.0185439	1.79E-29	7213
COL15A1	rs7867960	9	98999434	T	C	0.368293	0.350249	0.01685	3.00E-93	7213
THBS3	rs72704117	1	155205298	C	T	0.0194787	0.411296	0.059546	5.36E-12	7213
GOLM1	rs138447426	9	86060717	A	G	0.0173991	0.74455	0.0636318	2.42E-31	7213
SCUBE1	rs139004	22	43221033	A	C	0.308609	-0.128679	0.0178056	5.45E-13	7213
SCUBE1	rs5759290	22	43337746	T	A	0.150561	0.254109	0.0230512	4.92E-28	7213
TMEM2	rs1410988	9	71650180	G	A	0.288853	-0.239721	0.0180966	1.36E-39	7213
SERPINA11	rs55911632	14	94452451	A	G	0.159157	-0.346229	0.0225275	1.78E-52	7213
SERPINA11	rs12434141	14	94480981	G	A	0.164772	-0.139498	0.0220654	2.74E-10	7213
PLXNA1	rs9822602	3	126976937	A	G	0.314016	-0.431118	0.0170776	8.10E-135	7213
PRG3	rs12575356	11	57375990	T	G	0.113753	-0.146239	0.0259692	1.86E-08	7213
LCT	rs138530693	2	135587827	T	C	0.0123388	-0.629643	0.0746522	3.98E-17	7213
LCT	rs6760329	2	135787182	A	G	0.222931	-0.74486	0.0175946	0	7213
LCT	rs76496496	2	135985086	G	A	0.02953	-0.643367	0.0489845	5.87E-39	7213
LCT	rs150225621	2	136273744	T	G	0.0135173	-0.396632	0.0716783	3.25E-08	7213
PCDH10	rs62313606	4	133070877	C	A	0.408776	-0.210354	0.0166733	4.13E-36	7213
PCDH10	rs4864200	4	133546774	T	G	0.371343	0.235852	0.0167474	1.87E-44	7213
HAVCR1	rs6862569	5	157039537	G	C	0.281922	-0.466529	0.0177613	2.66E-145	7213
HAVCR1	rs76749041	5	157157316	C	T	0.0365313	0.293155	0.0442018	3.55E-11	7213
BTNL8	rs2387717	5	180938103	A	G	0.479412	-0.374847	0.0159801	2.53E-117	7213
PENK	rs2089984	8	56456607	A	G	0.464716	-0.535131	0.0152696	1.31E-248	7213
PENK	rs57401734	8	56570970	T	C	0.1507	0.274431	0.023052	2.24E-32	7213
PENK	rs4521751	8	56896436	G	A	0.416193	0.102186	0.0167644	1.15E-09	7213
MAN1A2	rs114957881	1	117207069	G	T	0.0298073	-0.446851	0.0487133	5.90E-20	7213
MAN1A2	rs75836610	1	117280122	G	T	0.0231526	0.380581	0.0549764	4.82E-12	7213
MAN1A2	rs4659047	1	117370451	A	G	0.115278	-0.569835	0.0252203	2.85E-109	7213
SNTB1	rs6982091	8	120534562	G	A	0.322473	-0.120568	0.017752	1.20E-11	7213
MMP8	rs11225395	11	102725749	A	G	0.439554	0.232217	0.0164592	1.31E-44	7213
MMP8	rs2155240	11	102966588	G	C	0.0266186	-0.290458	0.0514766	1.74E-08	7213
PGM1	rs1126728	1	63631761	C	T	0.221891	0.226637	0.0197827	3.98E-30	7213

PGM1	rs2269247	1	63641613	C	T	0.183142	0.224205	0.0212879	9.45E-26	7213
DSCAM	rs78111814	21	40688572	T	C	0.046028	0.467451	0.0389219	6.48E-33	7213
FAM3B	rs3746889	21	41280019	T	C	0.0335505	0.36381	0.0465442	6.21E-15	7213
FAM3B	rs77751326	21	41344765	G	C	0.0350062	-0.590823	0.0443653	5.44E-40	7213
FAM3B	rs57529409	21	41346335	C	T	0.0526133	-1.08511	0.0346428	4.86E-202	7213
IFNAR1	rs2248412	21	33233226	A	G	0.157355	-0.158794	0.0227304	3.08E-12	7213
IFNAR1	rs2254315	21	33355181	C	T	0.176764	-0.82496	0.0196485	0	7213
IFNAR1	rs2070388	21	33504401	G	C	0.108346	-0.255608	0.0266437	1.15E-21	7213
TFF1	rs3761376	21	42366929	G	A	0.239914	-0.365113	0.0193328	1.10E-77	7213
TFF1	rs117396825	21	42389187	T	C	0.0121309	0.440485	0.0758889	6.74E-09	7213
CXCL9	rs884304	4	76014110	G	A	0.289131	-0.115804	0.0183849	3.17E-10	7213
TFF2	rs58206891	21	42354303	A	G	0.179814	-0.125366	0.0214456	5.26E-09	7213
LGALS9	rs4239242	17	27647232	T	C	0.365798	-0.300878	0.0168424	7.06E-70	7213
TAGLN2	rs2789422	1	159922298	G	A	0.423957	-0.238889	0.0164801	5.88E-47	7213
SERPINF1	rs62088172	17	1762959	C	T	0.347359	-0.563544	0.0160783	1.14E-248	7213
CTSF	rs1044522	11	66568361	G	A	0.237349	0.298029	0.0192348	2.72E-53	7213
PLXNB2	rs75107793	22	50190508	G	A	0.0604464	0.340075	0.0347138	1.61E-22	7213
PLXNB2	rs17248301	22	50233668	T	C	0.0104672	0.444753	0.0809667	4.09E-08	7213
PLXNB2	rs28379706	22	50289633	T	C	0.397962	0.644354	0.0150419	0	7213
PLXNB2	rs131780	22	50544110	C	G	0.249619	-0.109229	0.0190521	1.03E-08	7213
PLXNB2	rs140753100	22	50552374	C	T	0.0208651	0.39895	0.0576821	5.03E-12	7213
MANF	rs4611808	3	51431657	T	C	0.137807	-0.356673	0.0238522	8.21E-50	7213
IMPAD1	rs78144087	8	56908855	G	A	0.0131707	0.407919	0.0729541	2.33E-08	7213
IMPAD1	rs57870584	8	57033935	C	T	0.0192708	0.374757	0.0607653	7.32E-10	7213
IMPAD1	rs112174080	8	57064678	C	A	0.0165673	-1.32566	0.0635985	9.59E-94	7213
IMPAD1	rs4738570	8	57092912	A	G	0.0678636	0.272872	0.0332057	2.44E-16	7213
IMPAD1	rs146504322	8	57193883	A	G	0.0155968	-0.503714	0.066841	5.44E-14	7213
TFPI2	rs17165852	7	93896196	C	G	0.0443643	-0.278301	0.0403732	5.92E-12	7213
TWSG1	rs62087477	18	9372548	A	T	0.077499	0.423284	0.0306483	7.65E-43	7213
SIRPG	rs6042354	20	1448490	C	T	0.400458	-0.102599	0.0169191	1.39E-09	7213
SIRPG	rs6043409	20	1635560	A	G	0.339387	-0.492955	0.016581	2.53E-183	7213
PPT1	rs4308964	1	39979216	C	T	0.255996	0.176682	0.0190488	2.30E-20	7213
PPT1	rs7533094	1	40094014	G	A	0.0469985	-1.11779	0.0367399	2.18E-191	7213
MAN2B2	rs2301788	4	6605137	A	G	0.48475	-0.843306	0.0136162	0	7213
MAN2B2	rs145066233	4	6625089	T	C	0.0417302	-0.374849	0.0405848	3.30E-20	7213
ABO	rs11244024	9	133183544	T	C	0.0157355	0.437098	0.066925	6.97E-11	7213
ABO	rs34764475	9	133186128	G	T	0.061001	-0.20474	0.0345222	3.16E-09	7213
ABO	rs8176632	9	133277095	C	T	0.137391	1.11785	0.0204887	0	7213
NPTX1	rs12949110	17	80460053	A	G	0.424858	-0.111313	0.0167546	3.28E-11	7213
NPTX1	rs62068270	17	80566625	C	G	0.0833911	0.670141	0.0288912	7.48E-115	7213
CTSO	rs2334114	4	155935041	C	A	0.210176	-0.214937	0.0203823	8.23E-26	7213
GLIPR1	rs7975447	12	75413201	G	C	0.394427	-0.0957356	0.0170003	1.85E-08	7213
TREM1	rs12204570	6	40959010	C	A	0.469846	0.169197	0.016537	2.10E-24	7213

TREM1	rs2234243	6	41276253	C	T	0.0849161	-0.890395	0.02796	2.42E-208	7213
TREM1	rs55857816	6	41298295	A	T	0.0923333	-0.241287	0.0286965	4.97E-17	7213
TREM1	rs56295949	6	41348816	G	A	0.0987107	0.165901	0.0278232	2.60E-09	7213
CPA4	rs10259610	7	130298871	C	G	0.0944129	-0.381637	0.0277729	1.96E-42	7213
CPA4	rs6467296	7	130307504	T	C	0.337377	-0.732744	0.0156198	0	7213
CPA4	rs117810108	7	130749672	T	G	0.0468598	0.233533	0.0389783	2.18E-09	7213
CXCL12	rs2818912	10	44087579	C	T	0.341397	-0.143758	0.0177209	5.79E-16	7213
CXCL12	rs17156193	10	44363991	A	G	0.202204	-0.151945	0.0209015	3.99E-13	7213
CRISP2	rs3997172	6	49505193	C	T	0.334604	-0.177735	0.0176032	8.22E-24	7213
CRISP2	rs555247	6	49742392	G	A	0.444059	0.502713	0.0157245	7.35E-210	7213
CRISP2	rs142915124	6	50162705	C	T	0.018231	-0.42158	0.0618753	1.03E-11	7213
FKBP7	rs1863671	2	178477603	T	C	0.169347	0.767546	0.0202922	9.04E-286	7213
FKBP7	rs114100829	2	178934849	T	C	0.080757	-0.286427	0.0299162	1.38E-21	7213
MFAP2	rs761422	1	16975285	A	G	0.47657	0.391934	0.0159198	1.42E-128	7213
B3GNT8	rs13345456	19	41301013	C	T	0.245945	0.161645	0.0191508	3.78E-17	7213
B3GNT8	rs11083618	19	41396406	C	T	0.3972	-0.794988	0.0140112	0	7213
B3GNT8	rs17267725	19	41690224	G	A	0.128102	0.154877	0.0246465	3.49E-10	7213
AZGP1	rs1981550	7	99944329	G	A	0.291626	-0.51842	0.0170957	3.20E-190	7213
CBLN1	rs12929787	16	48845548	T	C	0.0731318	0.221804	0.0320496	4.88E-12	7213
CBLN1	rs12597798	16	48960355	G	A	0.145016	-0.994999	0.0204511	0	7213
CBLN1	rs874466	16	49299378	C	T	0.316235	0.118517	0.0178367	3.26E-11	7213
PSG5	rs4536565	19	42874003	A	G	0.461389	0.152435	0.0163784	1.71E-20	7213
PSG5	rs1968068	19	43133663	C	T	0.366352	0.301252	0.0167579	1.02E-70	7213
PSG5	rs7253874	19	43140467	G	A	0.368363	-0.612888	0.0155557	1.14E-307	7213
PSG5	rs10421824	19	43363197	G	A	0.179953	-0.117537	0.0213993	4.10E-08	7213
WFDC1	rs400345	16	84294888	C	T	0.182795	-0.845478	0.0189855	0	7213
WFDC1	rs72804697	16	84414782	C	G	0.351102	0.11323	0.0175019	1.05E-10	7213
NMB	rs35127183	15	84672508	G	A	0.34424	-0.238447	0.0171572	2.36E-43	7213
ITIH2	rs80056062	10	7657587	C	T	0.0205878	-0.34315	0.0580917	3.64E-09	7213
ITIH2	rs112958019	10	7704871	C	T	0.0658533	1.35246	0.0295103	0	7213
PSG9	rs4802159	19	43189921	G	T	0.059268	-0.550983	0.0343132	4.90E-57	7213
PSG9	rs4299260	19	43294881	T	C	0.228199	0.183591	0.0196664	1.32E-20	7213
TAC1	rs17168679	7	97479442	T	C	0.0203799	0.408356	0.0585102	3.23E-12	7213
TAC1	rs10275909	7	97669509	G	A	0.207473	0.146062	0.0204339	9.67E-13	7213
TAC1	rs2072099	7	97733023	G	T	0.169971	-0.267924	0.0218742	3.74E-34	7213
FKBP2	rs72920394	11	64222830	G	A	0.0951061	-0.232746	0.0282704	2.15E-16	7213
PDGFD	rs10791660	11	104000311	C	A	0.183765	-0.39678	0.020944	3.77E-78	7213
IL2RB	rs228953	22	37135396	G	A	0.437335	0.154667	0.0166036	1.59E-20	7213
C1RL	rs1047776	12	6839528	A	G	0.368224	0.0941951	0.0171508	4.11E-08	7213
FSTL4	rs3749816	5	133199967	A	G	0.347428	-0.120028	0.0174711	6.95E-12	7213
FSTL4	rs111914149	5	133617238	G	A	0.267503	0.144165	0.0187355	1.61E-14	7213
CREG1	rs7513428	1	167546035	T	C	0.153542	0.334788	0.0229664	1.85E-47	7213
DLK2	rs1114357	6	43295191	C	T	0.198184	0.140836	0.0209474	1.91E-11	7213

DLK2	rs2125739	6	43445127	T	C	0.259254	-0.413378	0.0180676	6.65E-112	7213
EDIL3	rs10073371	5	83920960	T	C	0.121863	-0.149468	0.0254198	4.29E-09	7213
LRRC4C	rs10837377	11	40283726	C	T	0.434632	0.27077	0.0165135	2.37E-59	7213
GGH	rs116996805	8	62864588	G	A	0.0124775	-0.812127	0.0740059	8.47E-28	7213
GGH	rs4739037	8	62990765	G	A	0.0676556	0.910339	0.0315951	6.92E-173	7213
GGH	rs181070159	8	62996243	G	A	0.0236379	-0.703846	0.0536741	7.67E-39	7213
GGH	rs57906368	8	63213672	C	A	0.0167059	0.460309	0.0647233	1.25E-12	7213
PLA2G12B	rs12257692	10	72944091	T	C	0.0457507	-0.323922	0.0394722	2.68E-16	7213
CHI3L2	rs11556868	1	111235703	C	T	0.121239	0.294902	0.0252314	2.81E-31	7213
GAA	rs11658424	17	80009654	C	A	0.0318869	0.276564	0.0471935	4.83E-09	7213
GAA	rs2304849	17	80101663	G	C	0.251074	-0.376537	0.0185736	6.50E-89	7213
CPQ	rs72680187	8	96632223	A	G	0.0161514	-0.659551	0.0658321	1.80E-23	7213
CPQ	rs142439242	8	96713932	A	G	0.0165673	-1.12076	0.0630236	2.83E-69	7213
CPQ	rs76898837	8	96720777	G	T	0.0176764	-0.485275	0.0627223	1.16E-14	7213
CPQ	rs78562693	8	97038054	C	T	0.0262027	1.02155	0.0504073	7.39E-89	7213
TRABD2A	rs7598011	2	84863432	C	G	0.126022	0.141713	0.024847	1.22E-08	7213
TPSAB1	rs35116045	16	1257403	C	T	0.370789	0.358361	0.0166439	1.04E-99	7213
TPSAB1	rs4984785	16	1258908	C	T	0.276445	-0.328728	0.0180953	3.79E-72	7213
TPSAB1	rs34361866	16	1290631	G	A	0.111257	0.274207	0.0260574	1.04E-25	7213
TPSAB1	rs13336594	16	1482864	G	A	0.0500485	0.226051	0.0380307	2.91E-09	7213
CPM	rs12303166	12	68975897	A	G	0.0600305	0.280596	0.0345027	4.91E-16	7213
CPM	rs8181716	12	69024494	G	T	0.179884	-0.221862	0.0215798	1.27E-24	7213
C4BPA	rs11120218	1	207105106	G	A	0.130459	0.776306	0.0227487	9.58E-237	7213
C4BPA	rs45574833	1	207126725	G	A	0.0122695	0.49011	0.0754301	8.71E-11	7213
C4BPA	rs72742944	1	207127988	G	A	0.0562179	-0.217409	0.0358494	1.39E-09	7213
FAS	rs1389	10	88942948	G	A	0.18945	-0.139699	0.0212156	4.88E-11	7213
FAS	rs982764	10	89010241	T	C	0.304242	-0.433387	0.0173801	1.02E-131	7213
LMAN2	rs28419182	5	177301399	G	A	0.212672	-0.138173	0.0202791	1.03E-11	7213
METTL24	rs12193221	6	110040513	C	G	0.046028	-0.237375	0.0395804	2.10E-09	7213
METTL24	rs7767851	6	110356906	T	G	0.0915708	-0.291075	0.0284999	2.53E-24	7213
PRPSAP1	rs9907526	17	76319096	G	C	0.186053	0.12222	0.021193	8.40E-09	7213
NUDT9	rs28805573	4	87408035	C	A	0.137391	-0.459196	0.0235114	8.06E-83	7213
NUDT9	rs6531989	4	87414416	A	C	0.0180923	0.380085	0.0623802	1.16E-09	7213
DSG2	rs1460602	18	31501112	C	T	0.385485	-0.350854	0.0165438	7.05E-97	7213
DSG2	rs147145691	18	31526091	C	T	0.0227367	-0.596912	0.055704	1.36E-26	7213
PTK7	rs141855538	6	43087119	G	A	0.0359767	0.549598	0.0438105	9.99E-36	7213
EDDM3A	rs34552133	14	20747764	G	T	0.107999	-0.220957	0.0266561	1.35E-16	7213
GZMK	rs2407827	5	55011593	T	G	0.420421	-0.168677	0.0166971	7.78E-24	7213
MANSC1	rs3741798	12	12343152	C	T	0.11812	0.634892	0.0246054	1.68E-140	7213
BIN1	rs35103166	2	127124606	T	C	0.379107	0.189816	0.0169837	9.16E-29	7213
MANSC4	rs12367102	12	27767068	C	T	0.226605	0.889698	0.0166564	0	7213
MANSC4	rs11049166	12	27819909	G	A	0.401983	0.114448	0.0171316	2.56E-11	7213
MANSC4	rs140552413	12	27974093	C	G	0.0141411	0.38955	0.0701544	2.91E-08	7213

LAMC2	rs2276543	1	183186170	G	A	0.276445	0.733742	0.0165155	0	7213
LAMC2	rs4465156	1	183362725	C	T	0.101761	0.219283	0.0273629	1.29E-15	7213
LAMC2	rs7556032	1	183438511	C	G	0.448218	-0.187492	0.0165497	1.67E-29	7213
B4GALT2	rs1859728	1	43981741	G	C	0.0505338	-0.982315	0.0362223	2.62E-154	7213
B4GALT2	rs78538864	1	44042883	G	A	0.0383336	0.356529	0.0432819	2.07E-16	7213
PIANP	rs11064321	12	6700730	G	C	0.376958	0.345008	0.0165543	1.02E-93	7213
TIGIT	rs6792290	3	114294347	C	G	0.296825	0.125569	0.0180845	4.16E-12	7213
PDGFRL	rs2517259	8	17575073	T	C	0.175031	0.168557	0.0217836	1.15E-14	7213
PDGFRL	rs77670928	8	17596804	A	T	0.0361847	-0.825112	0.0435415	3.43E-78	7213
IGSF3	rs655735	1	116592497	G	A	0.404963	-0.204503	0.0166005	1.59E-34	7213
MMP16	rs4961090	8	88337442	A	G	0.316997	0.144903	0.0175147	1.54E-16	7213
DNAJA4	rs11639195	15	78255151	A	T	0.228546	0.115394	0.0197832	5.68E-09	7213
GSTM3	rs56380049	1	109463486	C	T	0.0659226	0.270932	0.0335202	7.38E-16	7213
GSTM3	rs1292096	1	109710758	A	G	0.206017	0.540104	0.0195113	2.23E-160	7213
NQO2	rs116107402	6	2940400	T	G	0.0385415	0.886585	0.0421059	1.45E-95	7213
NQO2	rs9378755	6	3000744	G	A	0.243588	-0.694315	0.0178625	3.07E-300	7213
NQO2	rs28383602	6	3002956	A	G	0.0496326	-0.556336	0.0378067	2.57E-48	7213
DNER	rs7577541	2	229344020	T	C	0.470193	-0.109036	0.0165756	5.10E-11	7213
DNER	rs35032874	2	229444644	T	G	0.296548	-0.159852	0.0181416	1.53E-18	7213
DNER	rs4972908	2	229657075	T	C	0.444337	0.129864	0.0166826	7.98E-15	7213
DNER	rs35975053	2	229729774	T	A	0.33398	-0.246397	0.0174123	7.33E-45	7213
NLGN2	rs150452493	17	7400489	C	T	0.0839457	-0.403137	0.0297727	2.87E-41	7213
BRSK2	rs4255564	11	1384495	C	T	0.265493	0.121941	0.0188383	1.02E-10	7213
IGDCC4	rs191350199	15	65421670	C	G	0.0141411	1.0898	0.0694791	1.51E-54	7213
IGDCC4	rs189124650	15	65603629	T	A	0.0271732	-0.380498	0.0502524	4.14E-14	7213
IGDCC4	rs76702386	15	65620544	T	C	0.0938583	-0.452024	0.0278438	3.09E-58	7213
CEL	rs2075733	9	133072397	A	G	0.0409677	0.442834	0.041603	2.90E-26	7213
ISOC1	rs35767068	5	128933241	T	C	0.298073	0.249558	0.0179339	1.86E-43	7213
FHIT	rs13070475	3	59983610	G	A	0.349092	-0.15566	0.0173392	3.49E-19	7213
SULT2A1	rs296384	19	47867143	G	T	0.151393	-0.364053	0.0228156	2.35E-56	7213
ADH1B	rs1229984	4	99318162	T	C	0.0325107	-0.29095	0.0465651	4.39E-10	7213
NQO1	rs10454066	16	69665102	T	A	0.190212	-0.77937	0.0190676	0	7213
SMAD1	rs2118438	4	145507232	A	G	0.195411	0.12324	0.0208727	3.70E-09	7213
TIRAP	rs8177398	11	126287999	C	T	0.0202412	0.340655	0.0587575	7.01E-09	7213
TIRAP	rs8177399	11	126290931	C	T	0.0237765	-1.35517	0.0519286	1.55E-143	7213
TIRAP	rs117827160	11	126337205	C	T	0.0105365	0.622056	0.0810855	1.92E-14	7213
ACTN1	rs76640185	14	69092813	G	C	0.0345903	-0.36557	0.0453121	8.32E-16	7213
ARHGDIB	rs10444404	12	14961817	T	G	0.408013	-0.151537	0.0168711	3.34E-19	7213
ARHGDIB	rs10772837	12	15123908	G	T	0.316165	0.147258	0.0178583	1.93E-16	7213
CCNH	rs2230641	5	87399457	A	G	0.22196	-0.165792	0.0198338	7.51E-17	7213
APEX1	rs1130409	14	20456995	T	G	0.468182	0.203126	0.0164676	1.31E-34	7213
APEX1	rs1760927	14	20488285	T	C	0.355331	0.127778	0.0172936	1.65E-13	7213
WARS	rs4905957	14	100370893	C	T	0.251352	-0.632638	0.0178442	6.12E-254	7213

ALDOC	rs141921160	17	28576910	C	T	0.0101206	-0.841634	0.0824206	2.58E-24	7213
CRKL	rs192565874	22	20688681	G	A	0.0754887	-0.219487	0.0313747	2.88E-12	7213
CRKL	rs117858197	22	20784951	T	C	0.0152502	-0.639355	0.068042	7.42E-21	7213
GLO1	rs12209477	6	38702023	C	G	0.38188	-0.194455	0.0169331	2.92E-30	7213
PPIL1	rs12194408	6	36871822	C	G	0.0291141	-1.35786	0.0468717	1.18E-174	7213
XRCC4	rs1056503	5	83353158	T	G	0.11403	0.31539	0.026048	2.02E-33	7213
SPOCK3	rs11736135	4	166925896	T	A	0.245598	-0.126725	0.0192884	5.38E-11	7213
SPOCK3	rs35065151	4	167127749	A	G	0.439969	-0.670458	0.0146632	0	7213
KRT1	rs117006546	12	52865871	A	T	0.0328573	0.30971	0.046633	3.33E-11	7213
DUSP28	rs73108005	2	240563949	G	A	0.0767364	0.193158	0.0311739	6.10E-10	7213
DUSP28	rs112548426	2	240660995	G	A	0.0339664	-0.860957	0.0452562	8.95E-79	7213
LAG3	rs3782735	12	6775910	G	A	0.399418	-0.170626	0.0168354	5.59E-24	7213
CDHR5	rs12421646	11	578844	C	G	0.226813	0.210369	0.0196519	1.53E-26	7213
KIAA1467	rs117798423	12	13056357	A	G	0.0192708	0.824693	0.05947	3.57E-43	7213
NPW	rs111276210	16	2016341	G	A	0.0632885	-0.305531	0.0335982	1.22E-19	7213
NPW	rs139482112	16	2023892	T	C	0.0122002	-0.439405	0.0756807	6.67E-09	7213
DUT	rs6493314	15	48273292	C	T	0.136212	-0.15394	0.0242834	2.45E-10	7213
