# Supplementary material for: Multicenter proteome-wide Mendelian randomization study identifies causal plasma proteins in melanoma and non-melanoma skin cancers
Source: Commun Biol. 2024 Jul 13;7:857. doi: 10.1038/s42003-024-06538-2 (PMC11246481; doi:10.1038/s42003-024-06538-2)
Supplement: Supplementary file 4 — Supplementary Data 2 [file 42003_2024_6538_MOESM4_ESM.pdf]

Supplementary Data 2. Investigating the Previous Genome-Wide Significant Associations of SNPs as Genetic Instruments for Potential Causal Proteins

Protein	SNP	Position	Year	nSample	nStudies	trait	effect_allele	other_allele	EAF	beta	se	pval	pmid	ancestry
ACADVL	rs377212765	chr17:7121672	2017	7637	1	Cause of death: cervix uteri, unspecified	A	G	0.002	0.05565	0.008153	9.43E-12	UKBB	European
ACADVL	rs377212765	chr17:7121672	2017	7637	1	Cause of death: urinary organ, unspecified	A	G	0.002	0.05508	0.00817	1.67E-11	UKBB	European
ACADVL	rs377212765	chr17:7121672	2017	7637	1	Cause of death: cerebrum, except lobes and ventricles	A	G	0.002	0.05595	0.006659	5.16E-17	UKBB	European
ACADVL	rs377212765	chr17:7121672	2017	7637	1	Cause of death: lobar pneumonia, unspecified	A	G	0.002	0.05498	0.008609	1.79E-10	UKBB	European
ACADVL	rs377212765	chr17:7121672	2017	7637	1	Cause of death: cholangitis	A	G	0.002	0.05213	0.008612	1.49E-09	UKBB	European
ACADVL	rs2521985	chr17:7121785	2017	193063	1	Birth weight	T	C	0.3638	-0.03433	0.003269	8.55E-26	UKBB	European
ACADVL	rs739669	chr17:7122377	2017	193063	1	Birth weight	A	G	0.6382	0.03463	0.003268	3.12E-26	UKBB	European
ACADVL	rs2017365	chr17:7122624	2017	193063	1	Birth weight	A	G	0.6213	0.03335	0.00326	1.45E-24	UKBB	European
ACADVL	rs146379816	chr17:7127698	2017	7637	1	Cause of death: follicular non-hodgkins lymphoma, unspecified	T	C	0	0.06051	0.0107	1.59E-08	UKBB	European
ACADVL	rs17671352	chr17:7127718	2017	193063	1	Birth weight	T	C	0.3777	-0.03351	0.003258	8.28E-25	UKBB	European
ASIP	rs2065911	chr20:32783596	2017	337159	1	Treatment with alphagan 0.2% eye drops	T	G	0.995	-0.001408	0.0002558	3.74E-08	UKBB	European
ASIP	rs117194239	chr20:32786564	2017	332021	1	Comparative height size at age 10	T	C	0.9811	0.04273	0.005202	2.165E-16	UKBB	European
ASIP	rs117194239	chr20:32786564	2017	336172	1	Sitting height	T	C	0.9811	0.05042	0.005893	1.172E-17	UKBB	European
ASIP	rs117194239	chr20:32786564	2017	336474	1	Height	T	C	0.9811	0.04427	0.005417	3.022E-16	UKBB	European
ASIP	rs118066733	chr20:32788052	2017	7637	1	Cause of death: multiple sclerosis	T	C	0.9901	-0.04046	0.006933	5.602E-09	UKBB	European
ASIP	rs1205306	chr20:32791469	2017	7637	1	Cause of death: cerebrum, except lobes and ventricles	T	C	0.002	0.03595	0.00536	2.139E-11	UKBB	European
ASIP	rs1205306	chr20:32791469	2017	337199	1	Other inflammatory spondylopathies	T	C	0.002	0.004073	0.0005848	3.285E-12	UKBB	European
ASIP	rs181246620	chr20:32793193	2017	7637	1	Cause of death: car occupant injured in unspecified traffic accident	A	C	0.002	0.05898	0.00796	1.405E-13	UKBB	European
ASIP	rs77600146	chr20:32796230	2017	332021	1	Comparative height size at age 10	A	G	0.0239	-0.03841	0.004958	9.527E-15	UKBB	European
ASIP	rs77600146	chr20:32796230	2017	336172	1	Sitting height	A	G	0.0239	-0.04675	0.005616	8.573E-17	UKBB	European
ASIP	rs77600146	chr20:32796230	2017	336474	1	Height	A	G	0.0239	-0.04158	0.005162	7.976E-16	UKBB	European
ASIP	rs181232383	chr20:32796370	2017	337159	1	Treatment with phenobarbitone	A	G	0.001	0.003336	0.0006034	3.229E-08	UKBB	European
ASIP	rs75420814	chr20:32796742	2017	332021	1	Comparative height size at age 10	A	G	0.9811	0.04271	0.005202	2.197E-16	UKBB	European
ASIP	rs75420814	chr20:32796742	2017	336172	1	Sitting height	A	G	0.9811	0.0504	0.005892	1.201E-17	UKBB	European
ASIP	rs75420814	chr20:32796742	2017	336474	1	Height	A	G	0.9811	0.04426	0.005416	3.044E-16	UKBB	European
ASIP	rs6088428	chr20:32797039	2017	336172	1	Sitting height	C	G	0.9523	-0.02482	0.004547	4.821E-08	UKBB	European
ASIP	rs6088428	chr20:32797039	2017	336474	1	Height	C	G	0.9523	-0.03263	0.004179	5.886E-15	UKBB	European
ASIP	rs1205313	chr20:32798832	2017	7637	1	Cause of death: cerebrum, except lobes and ventricles	T	G	0.999	-0.03451	0.005252	5.323E-11	UKBB	European
ASIP	rs1205313	chr20:32798832	2017	337199	1	Other inflammatory spondylopathies	T	G	0.999	-0.003893	0.0005716	9.756E-12	UKBB	European
ASIP	rs181620798	chr20:32800395	2017	7637	1	Cause of death: subarachnoid haemorrhage from intracranial artery, unspecified	A	G	0.998	-0.03878	0.007046	3.829E-08	UKBB	European
ASIP	rs181620798	chr20:32800395	2017	7637	1	Cause of death: calculus of gallbladder without cholecystitis	A	G	0.998	-0.05283	0.00799	4.037E-11	UKBB	European
ASIP	rs79839738	chr20:32800849	2017	332021	1	Comparative height size at age 10	A	T	0.0189	-0.04271	0.005202	2.212E-16	UKBB	European
ASIP	rs79839738	chr20:32800849	2017	336172	1	Sitting height	A	T	0.0189	-0.05039	0.005892	1.214E-17	UKBB	European
ASIP	rs79839738	chr20:32800849	2017	336474	1	Height	A	T	0.0189	-0.04426	0.005416	3.061E-16	UKBB	European
ASIP	rs116872416	chr20:32802416	2017	7637	1	Cause of death: follicular non-hodgkins lymphoma, unspecified	A	G	0.0278	0.01094	0.001867	4.856E-09	UKBB	European
ASIP	rs150972928	chr20:32803043	2017	7637	1	Cause of death: emphysema, unspecified	A	G	0.994	-0.03397	0.003697	5.101E-20	UKBB	European
ASIP	rs80021255	chr20:32803831	2017	332021	1	Comparative height size at age 10	A	G	0.0189	-0.0426	0.005214	3.072E-16	UKBB	European
ASIP	rs80021255	chr20:32803831	2017	336172	1	Sitting height	A	G	0.0189	-0.0504	0.005905	1.404E-17	UKBB	European
ASIP	rs80021255	chr20:32803831	2017	336474	1	Height	A	G	0.0189	-0.04423	0.005428	3.735E-16	UKBB	European
ASIP	rs77831801	chr20:32803832	2017	332021	1	Comparative height size at age 10	A	G	0.9821	0.0426	0.005214	3.072E-16	UKBB	European
ASIP	rs77831801	chr20:32803832	2017	336172	1	Sitting height	A	G	0.9821	0.0504	0.005905	1.404E-17	UKBB	European
ASIP	rs77831801	chr20:32803832	2017	336474	1	Height	A	G	0.9821	0.04423	0.005428	3.735E-16	UKBB	European
ASIP	rs192566501	chr20:32806043	2017	337159	1	Treatment with estradot 25micrograms patch	T	C	0.004	0.001291	0.0002361	4.568E-08	UKBB	European
ASIP	rs192566501	chr20:32806043	2017	7637	1	Cause of death: home	T	C	0.004	0.02517	0.003896	1.104E-10	UKBB	European
ASIP	rs6088437	chr20:32810291	2017	7637	1	Cause of death: subarachnoid haemorrhage from intracranial artery, unspecified	A	G	0.004	0.02239	0.00268	7.766E-17	UKBB	European
ASIP	rs117113297	chr20:32813689	2017	7637	1	Cause of death: follicular non-hodgkins lymphoma, unspecified	A	G	0.0209	0.01444	0.002383	1.45E-09	UKBB	European
ASIP	rs754897444	chr20:32821591	2017	7637	1	Cause of death: chronic or unspecified with haemorrhage	A	G	-	0.05051	0.008658	5.629E-09	UKBB	European
ASIP	rs754897444	chr20:32821591	2017	7637	1	Cause of death: alcoholic hepatitis	A	G	-	0.05093	0.006903	1.788E-13	UKBB	European
ASIP	rs561592714	chr20:32822970	2017	7637	1	Cause of death: acute and subacute infective endocarditis	A	G	0.002	0.04749	0.005597	2.554E-17	UKBB	European
ASIP	rs561592714	chr20:32822970	2017	7637	1	Cause of death: chronic or unspecified with haemorrhage	A	G	0.002	0.04571	0.008314	3.957E-08	UKBB	European
ASIP	rs139608260	chr20:32823326	2017	337159	1	Treatment with progesterone product	T	C	0.998	-0.004034	0.0007103	1.355E-08	UKBB	European
ASIP	rs139608260	chr20:32823326	2017	7637	1	Cause of death: alcoholic hepatitis	T	C	0.998	-0.0643	0.01099	5.022E-09	UKBB	European
ASIP	rs117915681	chr20:32828786	2017	336172	1	Sitting height	A	G	0.0368	0.03859	0.006211	5.204E-10	UKBB	European
ASIP	rs117915681	chr20:32828786	2017	336474	1	Height	A	G	0.0368	0.04488	0.00571	3.861E-15	UKBB	European
ASIP	rs113050381	chr20:32832722	2017	337159	1	Self-reported fibrosing alveolitis or unspecified alveolitis	A	G	0.001	0.00167	0.0002975	2.007E-08	UKBB	European
ASIP	rs113050381	chr20:32832722	2017	337159	1	Treatment with imdur 60mg durule	A	G	0.001	0.002502	0.0004132	1.398E-09	UKBB	European

ASIP	rs147396659	chr20:32838123	2017	7637	1	Cause of death: bronchiectasis	T	C	0.996	-0.1668	0.0162	1.073E-24	UKBB	European
ASIP	rs140437531	chr20:32841757	2017	7637	1	Cause of death: endocarditis, valve unspecified	T	C	0.999	-0.03323	0.005988	2.97E-08	UKBB	European
ASIP	rs187553326	chr20:32841876	2017	332021	1	Comparative height size at age 10	A	G	0.0159	-0.04335	0.005231	1.157E-16	UKBB	European
ASIP	rs187553326	chr20:32841876	2017	336172	1	Sitting height	A	G	0.0159	-0.05076	0.005925	1.059E-17	UKBB	European
ASIP	rs187553326	chr20:32841876	2017	336474	1	Height	A	G	0.0159	-0.04463	0.005446	2.504E-16	UKBB	European
ASIP	rs80149300	chr20:32842880	2017	336474	1	Height	T	C	0.0398	-0.02942	0.004951	2.815E-09	UKBB	European
ASIP	rs182686523	chr20:32847642	2017	337159	1	Treatment with strontium product	A	G	0.999	-0.002338	0.0004187	2.354E-08	UKBB	European
ASIP	rs182686523	chr20:32847642	2017	7637	1	Cause of death: secondary malignant neoplasm of retroperitoneum and peritoneum	A	G	0.999	-0.04035	0.005908	9.135E-12	UKBB	European
ASIP	rs6059743	chr20:32847711	2017	194398	1	Heel bone mineral density	A	G	0.4294	-0.01931	0.003155	9.284E-10	UKBB	European
ASIP	rs819135	chr20:32847767	2017	194398	1	Heel bone mineral density	A	G	0.5686	0.01939	0.003157	8.116E-10	UKBB	European
ASIP	rs1205312	chr20:32849416	2017	328725	1	Nervous feelings	A	G	0.0427	0.009889	0.001795	3.63E-08	UKBB	European
ASIP	rs1205312	chr20:32849416	2017	337159	1	Self-reported malignant melanoma	A	G	0.0427	0.002279	0.0003717	8.804E-10	UKBB	European
ASIP	rs1205312	chr20:32849416	2017	337159	1	Self-reported basal cell carcinoma	A	G	0.0427	0.002957	0.0004033	2.286E-13	UKBB	European
ASIP	rs1205312	chr20:32849416	2017	336172	1	Sitting height	A	G	0.0427	-0.03602	0.003226	6.097E-29	UKBB	European
ASIP	rs1205312	chr20:32849416	2017	336227	1	Weight	A	G	0.0427	-0.02392	0.003647	5.456E-11	UKBB	European
ASIP	rs1205312	chr20:32849416	2017	331291	1	Whole body fat-free mass	A	G	0.0427	-0.0249	0.002639	4.012E-21	UKBB	European
ASIP	rs1205312	chr20:32849416	2017	331315	1	Whole body water mass	A	G	0.0427	-0.02522	0.002643	1.396E-21	UKBB	European
ASIP	rs1205312	chr20:32849416	2017	331307	1	Basal metabolic rate	A	G	0.0427	-0.025	0.002768	1.674E-19	UKBB	European
ASIP	rs1205312	chr20:32849416	2017	331284	1	Impedance of whole body	A	G	0.0427	0.01957	0.003183	7.837E-10	UKBB	European
ASIP	rs1205312	chr20:32849416	2017	331279	1	Impedance of arm right	A	G	0.0427	0.01843	0.002935	3.391E-10	UKBB	European
ASIP	rs1205312	chr20:32849416	2017	331292	1	Impedance of arm left	A	G	0.0427	0.01831	0.002948	5.239E-10	UKBB	European
ASIP	rs1205312	chr20:32849416	2017	331285	1	Leg fat-free mass right	A	G	0.0427	-0.0227	0.002747	1.444E-16	UKBB	European
ASIP	rs1205312	chr20:32849416	2017	331285	1	Leg predicted mass right	A	G	0.0427	-0.02261	0.00273	1.21E-16	UKBB	European
ASIP	rs1205312	chr20:32849416	2017	331258	1	Leg fat-free mass left	A	G	0.0427	-0.02194	0.002748	1.428E-15	UKBB	European
ASIP	rs1205312	chr20:32849416	2017	331253	1	Leg predicted mass left	A	G	0.0427	-0.02183	0.00273	1.269E-15	UKBB	European
ASIP	rs1205312	chr20:32849416	2017	331221	1	Arm fat-free mass right	A	G	0.0427	-0.02497	0.002601	7.989E-22	UKBB	European
ASIP	rs1205312	chr20:32849416	2017	331216	1	Arm predicted mass right	A	G	0.0427	-0.02504	0.002592	4.344E-22	UKBB	European
ASIP	rs1205312	chr20:32849416	2017	331159	1	Arm fat-free mass left	A	G	0.0427	-0.02387	0.002654	2.387E-19	UKBB	European
ASIP	rs1205312	chr20:32849416	2017	331146	1	Arm predicted mass left	A	G	0.0427	-0.02475	0.002644	7.864E-21	UKBB	European
ASIP	rs1205312	chr20:32849416	2017	331030	1	Trunk fat-free mass	A	G	0.0427	-0.0255	0.002629	3.049E-22	UKBB	European
ASIP	rs1205312	chr20:32849416	2017	330995	1	Trunk predicted mass	A	G	0.0427	-0.02561	0.00262	1.5E-22	UKBB	European
ASIP	rs1205312	chr20:32849416	2017	336474	1	Height	A	G	0.0427	-0.02695	0.002966	1.02E-19	UKBB	European
ASIP	rs1205312	chr20:32849416	2017	337199	1	Other malignant neoplasms of skin	A	G	0.0427	0.003298	0.0004974	3.332E-11	UKBB	European
ASIP	rs568690809	chr20:32852394	2017	337159	1	Self-reported fibrosing alveolitis or unspecified alveolitis	T	C	0.999	-0.003084	0.0005324	6.971E-09	UKBB	European
ASIP	rs568690809	chr20:32852394	2017	7637	1	Cause of death: subarachnoid haemorrhage from intracranial artery, unspecified	T	C	0.999	-0.049	0.007478	6.041E-11	UKBB	European
ASIP	rs568690809	chr20:32852394	2017	337199	1	Secondary and unspecified malignant neoplasm of lymph nodes	T	C	0.999	-0.007298	0.001118	6.731E-11	UKBB	European
BOLA1	rs17581597	chr1:149860372	2014	242340	79	Height	T	C	0.0845	-0.037	0.0056	4.8E-11	3E+07	European
BOLA1	rs17581597	chr1:149860372	2017	332021	1	Comparative height size at age 10	T	C	0.0845	-0.01929	0.002855	1.418E-11	UKBB	European
BOLA1	rs17581597	chr1:149860372	2017	331284	1	Impedance of whole body	T	C	0.0845	-0.02213	0.003189	3.934E-12	UKBB	European
BOLA1	rs17581597	chr1:149860372	2017	331279	1	Impedance of arm right	T	C	0.0845	-0.02561	0.002941	3.089E-18	UKBB	European
BOLA1	rs17581597	chr1:149860372	2017	331292	1	Impedance of arm left	T	C	0.0845	-0.02352	0.002953	1.653E-15	UKBB	European
BOLA1	rs17581597	chr1:149860372	2017	336474	1	Height	T	C	0.0845	-0.02579	0.002971	3.985E-18	UKBB	European
BOLA1	rs6696191	chr1:149862367	2017	332021	1	Comparative height size at age 10	A	G	0.1252	-0.01705	0.002716	3.441E-10	UKBB	European
BOLA1	rs6696191	chr1:149862367	2017	336474	1	Height	A	G	0.1252	-0.02091	0.002827	1.418E-13	UKBB	European
BOLA1	rs186893352	chr1:149869245	2017	7637	1	Cause of death: creutzfeldt-jakob disease	A	G	0.003	0.05156	0.005974	7.365E-18	UKBB	European
BOLA1	rs143441849	chr1:149869573	2017	331279	1	Impedance of arm right	A	G	0.0139	-0.0466	0.007125	6.122E-11	UKBB	European
BOLA1	rs6700818	chr1:149870480	2017	337199	1	Procreative management	T	C	0.0089	0.001804	0.0002973	1.297E-09	UKBB	European
BOLA1	rs1355237	chr1:149871278	2017	336474	1	Height	T	C	0.0189	-0.04357	0.006396	9.631E-12	UKBB	European
CLMP	rs528279368	chr11:122943496	2017	7637	1	Cause of death: heart failure, unspecified	A	G	0.006	0.03349	0.006033	2.936E-08	UKBB	European
CLMP	rs118065564	chr11:122944106	2017	7637	1	Cause of death: peritoneum, unspecified	A	G	0.0089	0.0297	0.005428	4.609E-08	UKBB	European
CLMP	rs142239120	chr11:122944416	2017	7637	1	Cause of death: endocarditis, valve unspecified	A	G	0.003	0.01825	0.003287	2.934E-08	UKBB	European
CLMP	rs4936771	chr11:122944739	2016	173480	2	Red cell distribution width	A	T	0.9672	-0.05526	0.008917	5.736E-10	3E+07	European
CLMP	rs11218953	chr11:122945908	2016	173480	2	Red cell distribution width	A	C	0.9672	-0.05509	0.008925	6.702E-10	3E+07	European
CLMP	rs535018840	chr11:122946118	2017	7637	1	Cause of death: lobar pneumonia, unspecified	A	G	0.001	0.04202	0.007506	2.232E-08	UKBB	European
CLMP	rs76381550	chr11:122946401	2016	173480	2	Red cell distribution width	T	C	0.9672	-0.05528	0.008905	5.37E-10	3E+07	European
CLMP	rs117854976	chr11:122946417	2017	337159	1	Treatment with forceval capsule	A	G	0.999	-0.003057	0.0005523	3.101E-08	UKBB	European
CLMP	rs553450675	chr11:122948899	2017	337199	1	Dislocation, sprain and strain of joints and ligaments at neck level	A	T	0.001	0.002691	0.0004567	3.811E-09	UKBB	European
CLMP	rs7115313	chr11:122949414	2016	173480	2	Red cell distribution width	A	G	0.0328	0.05518	0.008904	5.753E-10	3E+07	European

CLMP	rs7115329	chr11:122949545	2016	173480	2	Red cell distribution width	C	G	0.9672	-0.05598	0.008949	3.967E-10	3E+07	European
CLMP	rs7115329	chr11:122949545	2016	-	-	Red cell distribution width	C	G	0.9672	-0.05598	0.008951	4E-10	3E+07	European
CLMP	rs11218960	chr11:122949658	2016	173480	2	Red cell distribution width	A	G	0.9672	-0.05541	0.00893	5.446E-10	3E+07	European
CLMP	rs571517658	chr11:122950619	2017	7637	1	Cause of death: asthma, unspecified	A	G	-	0.0531	0.008846	2.024E-09	UKBB	European
CLMP	rs189041237	chr11:122950988	2017	7637	1	Cause of death: appendix	A	G	0.003	0.02591	0.004191	6.614E-10	UKBB	European
CLMP	rs189041237	chr11:122950988	2017	337199	1	Mental disorder, not otherwise specified	A	G	0.003	0.002143	0.000374	1.008E-08	UKBB	European
CLMP	rs536392875	chr11:122953119	2017	7637	1	Cause of death: unspecified dementia	C	G	0.999	-0.1089	0.01801	1.557E-09	UKBB	European
CLMP	rs536392875	chr11:122953119	2017	7637	1	Cause of death: bronchiectasis	C	G	0.999	-0.1205	0.01392	5.858E-18	UKBB	European
CLMP	rs536392875	chr11:122953119	2017	7637	1	Cause of death: diverticular disease of intestine, part unspecified, with perforation and abscess	C	G	0.999	-0.06071	0.00902	1.805E-11	UKBB	European
CLMP	rs192139593	chr11:122955172	2017	7637	1	Cause of death: malignant neoplasm of thyroid gland	A	G	0.998	-0.0205	0.0033	5.555E-10	UKBB	European
CLMP	rs554669889	chr11:122955695	2017	7637	1	Cause of death: vascular dementia, unspecified	T	C	-	0.06181	0.01128	4.363E-08	UKBB	European
CLMP	rs554669889	chr11:122955695	2017	7637	1	Cause of death: lobar pneumonia, unspecified	T	C	-	0.06469	0.01075	1.84E-09	UKBB	European
CLMP	rs116923515	chr11:122956946	2017	7637	1	Cause of death: other specified respiratory disorders	A	G	0.994	-0.01654	0.002985	3.141E-08	UKBB	European
CLMP	rs11218968	chr11:122958478	2016	173480	2	Red cell distribution width	A	G	0.9682	-0.05527	0.008959	6.862E-10	3E+07	European
CLMP	rs146611698	chr11:122961589	2017	337159	1	Treatment with normacol granules	A	G	0.999	-0.006015	0.0009041	2.879E-11	UKBB	European
CLMP	rs146611698	chr11:122961589	2017	337199	1	Other cardiac arrhythmias	A	G	0.999	-0.009203	0.001618	1.281E-08	UKBB	European
CLMP	rs191500452	chr11:122961753	2017	7637	1	Cause of death: cerebral infarction, unspecified	T	C	0.999	-0.07121	0.009219	1.261E-14	UKBB	European
CLMP	rs7131609	chr11:122963584	2017	7637	1	Cause of death: other and unspecified intestinal obstruction	A	G	0.0835	0.005357	0.000909	3.941E-09	UKBB	European
CLMP	rs117071839	chr11:122965115	2017	7637	1	Cause of death: caecum	A	G	0.003	0.05652	0.009601	4.1E-09	UKBB	European
CLMP	rs187279065	chr11:122966840	2017	7637	1	Cause of death: heart failure, unspecified	A	G	0.003	0.04947	0.005128	6.736E-22	UKBB	European
CLMP	rs148200915	chr11:122967699	2017	337159	1	Treatment with clonazepam	A	G	0.001	0.006545	0.0009952	4.809E-11	UKBB	European
CLMP	rs148200915	chr11:122967699	2017	7637	1	Cause of death: duodenum	A	G	0.001	0.06531	0.009312	2.523E-12	UKBB	European
CLMP	rs191261785	chr11:122967856	2017	7637	1	Cause of death: gastro-intestinal haemorrhage, unspecified	A	G	0.003	0.04053	0.006273	1.108E-10	UKBB	European
CLMP	rs543316624	chr11:122972069	2017	7637	1	Cause of death: unspecified place	T	C	0.002	0.01877	0.003306	1.416E-08	UKBB	European
CLMP	rs569024773	chr11:122974770	2017	7637	1	Cause of death: cerebrovascular disease, unspecified	T	C	0.999	-0.05439	0.008979	1.452E-09	UKBB	European
CLMP	rs147798838	chr11:122975519	2017	7637	1	Cause of death: appendix	A	G	0.003	0.02537	0.004153	1.062E-09	UKBB	European
CLMP	rs148896935	chr11:122982177	2017	7637	1	Cause of death: hodgkins disease, unspecified	T	G	0.004	0.03715	0.005462	1.116E-11	UKBB	European
CLMP	rs190436785	chr11:122984279	2017	337159	1	Treatment with ventolin 2mg tablet	A	G	0.995	-0.003087	0.0005321	6.561E-09	UKBB	European
CLMP	rs190436785	chr11:122984279	2017	337159	1	Treatment with tenofovir	A	G	0.995	-0.002581	0.0004319	2.294E-09	UKBB	European
CLMP	rs190436785	chr11:122984279	2017	7637	1	Cause of death: hodgkins disease, unspecified	A	G	0.995	-0.03313	0.005139	1.207E-10	UKBB	European
CLMP	rs190436785	chr11:122984279	2017	7637	1	Cause of death: subarachnoid haemorrhage from intracranial artery, unspecified	A	G	0.995	-0.03292	0.00555	3.153E-09	UKBB	European
CLMP	rs190436785	chr11:122984279	2017	24008	1	Knee surgery or amputation above the knee	A	G	0.995	-0.03307	0.004783	4.852E-12	UKBB	European
CLMP	rs567646127	chr11:122990271	2017	337159	1	Treatment with sustanon 100 oily injection	A	G	0	0.003906	0.00056	3.044E-12	UKBB	European
CLMP	rs567646127	chr11:122990271	2017	337159	1	Treatment with zapain caplet	A	G	0	0.004294	0.0007314	4.355E-09	UKBB	European
CLMP	rs567646127	chr11:122990271	2017	333997	1	Home area population density: postcode not linkable	A	G	0	0.00154	0.000238	9.802E-11	UKBB	European
CLMP	rs567646127	chr11:122990271	2017	7637	1	Cause of death: cholangitis	A	G	0	0.06378	0.009213	4.769E-12	UKBB	European
CLMP	rs567646127	chr11:122990271	2017	337199	1	Other inflammatory spondylopathies	A	G	0	0.005599	0.0008801	1.993E-10	UKBB	European
CLMP	rs191440463	chr11:122991864	2017	7637	1	Cause of death: endometrium	A	G	0.998	-0.09339	0.0168	0.000000028	UKBB	European
CLMP	rs191440463	chr11:122991864	2017	7637	1	Cause of death: acute lymphoblastic leukaemia	A	G	0.998	-0.03521	0.006243	1.77E-08	UKBB	European
CLMP	rs191440463	chr11:122991864	2017	7637	1	Cause of death: thoracic aortic aneurysm, ruptured	A	G	0.998	-0.04214	0.00674	4.269E-10	UKBB	European
CLMP	rs191440463	chr11:122991864	2017	7637	1	Cause of death: chronic or unspecified with haemorrhage	A	G	0.998	-0.04756	0.00845	1.89E-08	UKBB	European
CLMP	rs182404694	chr11:122991871	2017	7637	1	Cause of death: intracerebral haemorrhage, unspecified	A	G	0.002	0.1352	0.01933	2.921E-12	UKBB	European
CLMP	rs182404694	chr11:122991871	2017	337199	1	Other disorders of conjunctiva	A	G	0.002	0.004766	0.0007761	8.193E-10	UKBB	European
CLMP	rs182404694	chr11:122991871	2017	337199	1	Crushing injury of wrist and hand	A	G	0.002	0.002517	0.0004142	1.232E-09	UKBB	European
CLMP	rs75474776	chr11:122997154	2017	7637	1	Cause of death: oropharynx, unspecified	T	C	0.0089	0.02119	0.003838	3.465E-08	UKBB	European
CLMP	rs554902536	chr11:122997562	2017	7637	1	Cause of death: secondary malignant neoplasm of retroperitoneum and peritoneum	A	G	0.003	0.02967	0.00429	4.99E-12	UKBB	European
CLMP	rs183637828	chr11:122998081	2017	7637	1	Cause of death: duodenum	T	C	-	0.04773	0.008496	1.994E-08	UKBB	European
CLMP	rs183637828	chr11:122998081	2017	7637	1	Cause of death: fatty liver	T	C	-	0.05296	0.008043	4.857E-11	UKBB	European
CLMP	rs183637828	chr11:122998081	2017	337199	1	Malignant neoplasm of rectosigmoid junction	T	C	-	0.006348	0.001044	1.209E-09	UKBB	European
CLMP	rs190170315	chr11:122999088	2017	7637	1	Cause of death: cerebral infarction, unspecified	A	G	0.001	0.05888	0.0084	2.59E-12	UKBB	European
CLMP	rs188130979	chr11:123006164	2017	337159	1	Treatment with respiratory mometasone	T	G	0.003	0.003666	0.0006005	1.026E-09	UKBB	European
CLMP	rs188130979	chr11:123006164	2017	7637	1	Cause of death: other and unspecified t-cell lymphomas	T	G	0.003	0.06078	0.006003	6.077E-24	UKBB	European
CLMP	rs188130979	chr11:123006164	2017	7637	1	Cause of death: acute vascular disorders of intestine	T	G	0.003	0.05994	0.006332	3.778E-21	UKBB	European
CLMP	rs11219015	chr11:123008115	2017	317756	1	Pulse rate	A	G	0.2356	-0.01617	0.002934	3.555E-08	UKBB	European
CLMP	rs7925085	chr11:123008209	2017	7637	1	Cause of death: cerebrovascular disease, unspecified	T	C	1	-0.04516	0.008192	3.646E-08	UKBB	European
CLMP	rs12283343	chr11:123008349	2017	317756	1	Pulse rate	C	G	0.2336	-0.0162	0.002942	3.71E-08	UKBB	European
CLMP	rs12283311	chr11:123008467	2017	317756	1	Pulse rate	C	G	0.7664	0.01624	0.002942	3.38E-08	UKBB	European
CLMP	rs559092532	chr11:123011144	2017	7637	1	Cause of death: hodgkins disease, unspecified	A	G	0.999	-0.03335	0.005357	5.075E-10	UKBB	European

CLMP	rs559092532	chr11:123011144	2017	7637	1	Cause of death: interstitial pulmonary disease, unspecified	A	G	0.999	-0.06643	0.009524	3.299E-12	UKBB	European
CLMP	rs189325746	chr11:123012445	2017	337159	1	Treatment with respiratory mometasone	T	C	0.002	0.00324	0.0005682	1.183E-08	UKBB	European
CLMP	rs189325746	chr11:123012445	2017	7637	1	Cause of death: other and unspecified t-cell lymphomas	T	C	0.002	0.05497	0.005715	8.951E-22	UKBB	European
CLMP	rs189325746	chr11:123012445	2017	7637	1	Cause of death: acute vascular disorders of intestine	T	C	0.002	0.05474	0.006028	1.348E-19	UKBB	European
CLMP	rs183940951	chr11:123014775	2017	337159	1	Treatment with vitamin k product	A	G	0.001	0.003731	0.0004782	6.096E-15	UKBB	European
CLMP	rs183940951	chr11:123014775	2017	7637	1	Cause of death: cervix uteri, unspecified	A	G	0.001	0.0374	0.006743	3.014E-08	UKBB	European
CLMP	rs537924479	chr11:123015102	2017	7637	1	Cause of death: cerebrum, except lobes and ventricles	T	C	0.001	0.03627	0.006383	1.374E-08	UKBB	European
CLMP	rs537924479	chr11:123015102	2017	7637	1	Cause of death: fatty liver	T	C	0.001	0.05154	0.007801	4.18E-11	UKBB	European
CLMP	rs574079630	chr11:123023161	2017	7637	1	Cause of death: without complications	A	G	0.002	0.02789	0.00476	4.849E-09	UKBB	European
CLMP	rs574079630	chr11:123023161	2017	7637	1	Cause of death: alcoholic cirrhosis of liver	A	G	0.002	0.04919	0.007759	2.429E-10	UKBB	European
CLMP	rs182298584	chr11:123031850	2017	7637	1	Cause of death: other specified respiratory disorders	T	G	0.998	-0.02232	0.003476	1.435E-10	UKBB	European
CLMP	rs767140211	chr11:123035036	2017	7637	1	Cause of death: creutzfeldt-jakob disease	A	G	-	-0.06127	0.009731	3.227E-10	UKBB	European
CLMP	rs186558910	chr11:123038046	2017	7637	1	Cause of death: malignant neoplasm of thyroid gland	T	C	0.001	0.02543	0.004567	2.666E-08	UKBB	European
CLMP	rs186558910	chr11:123038046	2017	7637	1	Cause of death: heart failure, unspecified	T	C	0.001	0.03871	0.006455	2.101E-09	UKBB	European
CLMP	rs11607787	chr11:123044778	2017	7637	1	Cause of death: brain, unspecified	A	G	0.005	0.05258	0.009622	4.783E-08	UKBB	European
CLMP	rs144498880	chr11:123045616	2017	7637	1	Cause of death: cholangitis	A	G	0.999	-0.0631	0.009172	6.469E-12	UKBB	European
CLMP	rs144498880	chr11:123045616	2017	7637	1	Cause of death: gastro-intestinal haemorrhage, unspecified	A	G	0.999	-0.0566	0.007675	1.831E-13	UKBB	European
CLMP	rs10502266	chr11:123046519	2017	7637	1	Cause of death: other ill-defined and unspecified causes of mortality	C	G	0.005	0.09534	0.01637	5.917E-09	UKBB	European
CLMP	rs747801270	chr11:123046588	2017	7637	1	Cause of death: malignant neoplasm of thyroid gland	T	G	-	-0.02474	0.004519	4.542E-08	UKBB	European
CLMP	rs747801270	chr11:123046588	2017	7637	1	Cause of death: heart failure, unspecified	T	G	-	-0.03791	0.006388	3.08E-09	UKBB	European
CLMP	rs11219050	chr11:123049798	2015	442	1	NKeff:%314-158a+; NK: (CD314-CD158a+)	T	C	0.4215	0.1114	0.01926	7.305E-09	3E+07	European
CLMP	rs562249605	chr11:123050176	2017	337159	1	Treatment with nitrazepam	A	T	0.001	0.005513	0.0009055	1.139E-09	UKBB	European
CLMP	rs562249605	chr11:123050176	2017	337159	1	Treatment with inderal 10mg tablet	A	T	0.001	0.004494	0.0006323	1.187E-12	UKBB	European
CLMP	rs562249605	chr11:123050176	2017	337159	1	Treatment with clonazepam	A	T	0.001	0.005801	0.0009832	3.644E-09	UKBB	European
CLMP	rs562249605	chr11:123050176	2017	7637	1	Cause of death: other and unspecified t-cell lymphomas	A	T	0.001	0.09042	0.01037	3.443E-18	UKBB	European
CLMP	rs562249605	chr11:123050176	2017	7637	1	Cause of death: chronic myeloid leukaemia	A	T	0.001	0.06955	0.009797	1.372E-12	UKBB	European
CLMP	rs181503801	chr11:123050977	2017	7637	1	Cause of death: cervix uteri, unspecified	A	G	0.001	0.0423	0.007158	3.602E-09	UKBB	European
CLMP	rs181503801	chr11:123050977	2017	7637	1	Cause of death: cardiomegaly	A	G	0.001	0.07306	0.01013	5.907E-13	UKBB	European
CLMP	rs59327612	chr11:123051141	2017	7637	1	Cause of death: multisystem degeneration	A	G	0.9861	-0.07145	0.008878	9.69E-16	UKBB	European
CLMP	rs566994199	chr11:123051712	2017	7637	1	Cause of death: malignant neoplasm of thyroid gland	T	C	0.999	-0.02574	0.004613	2.498E-08	UKBB	European
CLMP	rs566994199	chr11:123051712	2017	7637	1	Cause of death: heart failure, unspecified	T	C	0.999	-0.03956	0.00652	1.368E-09	UKBB	European
CLMP	rs185759811	chr11:123053562	2017	337159	1	Treatment with nitrolingual 400micrograms spray	A	C	0	0.006468	0.0009977	8.977E-11	UKBB	European
CLMP	rs185759811	chr11:123053562	2017	7637	1	Cause of death: dilated cardiomyopathy	A	C	0	0.07796	0.01097	1.289E-12	UKBB	European
CLMP	rs6589971	chr11:123054531	2017	7637	1	Cause of death: brain, unspecified	C	G	0.995	-0.04697	0.008116	7.411E-09	UKBB	European
CLMP	rs138573855	chr11:123057291	2017	7637	1	Cause of death: endometrium	A	G	0.006	0.09837	0.01498	5.429E-11	UKBB	European
CLMP	rs6589972	chr11:123057496	2017	7637	1	Cause of death: brain, unspecified	A	G	0.005	0.04183	0.007514	2.671E-08	UKBB	European
CLMP	rs117916958	chr11:123060440	2017	7637	1	Cause of death: mesothelioma, unspecified	A	G	0.004	0.09404	0.01705	3.615E-08	UKBB	European
CLMP	rs117916958	chr11:123060440	2017	7637	1	Cause of death: cardiomyopathy, unspecified	A	G	0.004	0.05039	0.007929	2.197E-10	UKBB	European
CLMP	rs11219056	chr11:123060568	2017	7637	1	Cause of death: brain, unspecified	A	G	0.005	0.05796	0.008808	5.021E-11	UKBB	European
CLMP	rs878297	chr11:123061307	2017	7637	1	Cause of death: brain, unspecified	A	C	0.005	0.05167	0.008326	5.727E-10	UKBB	European
CLMP	rs878297	chr11:123061307	2017	337199	1	Systemic sclerosis	A	C	0.005	0.002733	0.0004688	5.578E-09	UKBB	European
CLMP	rs10790561	chr11:123061605	2017	7637	1	Cause of death: brain, unspecified	A	G	0.005	0.05174	0.008329	5.521E-10	UKBB	European
CLMP	rs10790561	chr11:123061605	2017	337199	1	Systemic sclerosis	A	G	0.005	0.002733	0.0004686	5.453E-09	UKBB	European
CLMP	rs10892980	chr11:123063025	2017	7637	1	Cause of death: brain, unspecified	T	C	0.995	-0.05594	0.00865	1.06E-10	UKBB	European
CLMP	rs11219059	chr11:123063082	2017	7637	1	Cause of death: brain, unspecified	C	G	0.995	-0.04893	0.00811	1.689E-09	UKBB	European
CLMP	rs11219059	chr11:123063082	2017	337199	1	Systemic sclerosis	C	G	0.995	-0.00266	0.0004627	9.01E-09	UKBB	European
CLMP	rs150962578	chr11:123063342	2017	337159	1	Self-reported plantar fascitis	T	C	0.005	0.001891	0.0003385	2.318E-08	UKBB	European
CLMP	rs572711838	chr11:123063360	2017	337159	1	Self-reported stomach cancer	T	G	0.999	-0.003983	0.0005833	8.656E-12	UKBB	European
CLMP	rs11219060	chr11:123064185	2017	7637	1	Cause of death: brain, unspecified	T	C	0.005	0.049	0.008117	1.649E-09	UKBB	European
CLMP	rs11219060	chr11:123064185	2017	337199	1	Systemic sclerosis	T	C	0.005	0.002638	0.000461	1.052E-08	UKBB	European
CNTN2	rs35261626	chr1:205013084	2016	173480	2	Basophil count	T	G	0.5706	-0.02173	0.003637	2.312E-09	3E+07	European
CNTN2	rs35261626	chr1:205013084	2016	173480	2	Mean corpuscular hemoglobin	T	G	0.5706	0.02304	0.003679	3.828E-10	3E+07	European
CNTN2	rs35261626	chr1:205013084	2016	173480	2	Mean platelet volume	T	G	0.5706	-0.05529	0.003753	3.85E-49	3E+07	European
CNTN2	rs575329111	chr1:205013623	2017	337199	1	Other disorders of ear	A	G	0.008	0.002496	0.0003859	9.89E-11	UKBB	European
CNTN2	rs546447393	chr1:205014085	2017	7637	1	Cause of death: ischaemic cardiomyopathy	T	C	-	0.06814	0.01003	1.196E-11	UKBB	European
CNTN2	rs546447393	chr1:205014085	2017	7637	1	Cause of death: pneumonitis due to food and vomit	T	C	-	0.05672	0.008976	2.779E-10	UKBB	European
CNTN2	rs546447393	chr1:205014085	2017	7637	1	Cause of death: home	T	C	-	0.05552	0.008983	6.703E-10	UKBB	European
CNTN2	rs3753847	chr1:205014539	2016	173480	2	Mean corpuscular hemoglobin	T	C	0.174	-0.02902	0.005002	6.561E-09	3E+07	European

CNTN2	rs3753847	chr1:205014539	2016	173480	2	Mean platelet volume	T	C	0.174	0.08546	0.005115	1.166E-62	3E+07	European
CNTN2	rs3753847	chr1:205014539	2016	173480	2	Platelet count	T	C	0.174	-0.03746	0.005156	3.72E-13	3E+07	European
CNTN2	rs3820337	chr1:205014930	2016	173480	2	Mean corpuscular hemoglobin	T	C	0.1531	-0.02998	0.005114	4.556E-09	3E+07	European
CNTN2	rs3820337	chr1:205014930	2016	173480	2	Mean platelet volume	T	C	0.1531	0.08363	0.005228	1.378E-57	3E+07	European
CNTN2	rs3820337	chr1:205014930	2016	173480	2	Platelet count	T	C	0.1531	-0.03428	0.005271	7.848E-11	3E+07	European
CNTN2	rs182439442	chr1:205015020	2017	7637	1	Cause of death: acute lymphoblastic leukaemia	A	G	0.002	0.08489	0.008188	5.112E-25	UKBB	European
CNTN2	rs182439442	chr1:205015020	2017	7637	1	Cause of death: perforation of intestine	A	G	0.002	0.08524	0.0106	1.016E-15	UKBB	European
CNTN2	rs11240341	chr1:205015284	2016	173480	2	Basophil percentage of white cells	T	C	0.338	0.02038	0.003721	4.331E-08	3E+07	European
CNTN2	rs11240341	chr1:205015284	2016	173480	2	High light scatter reticulocyte count	T	C	0.338	0.02325	0.00384	1.402E-09	3E+07	European
CNTN2	rs11240341	chr1:205015284	2016	173480	2	Immature fraction of reticulocytes	T	C	0.338	0.02553	0.003804	1.923E-11	3E+07	European
CNTN2	rs11240341	chr1:205015284	2016	173480	2	Mean corpuscular hemoglobin	T	C	0.338	-0.03281	0.003793	5.083E-18	3E+07	European
CNTN2	rs11240341	chr1:205015284	2016	173480	2	Mean corpuscular hemoglobin concentration	T	C	0.338	-0.02285	0.003712	7.401E-10	3E+07	European
CNTN2	rs11240341	chr1:205015284	2016	173480	2	Mean corpuscular volume	T	C	0.338	-0.02518	0.00378	2.692E-11	3E+07	European
CNTN2	rs11240341	chr1:205015284	2016	173480	2	Mean platelet volume	T	C	0.338	0.07507	0.003871	9.313E-84	3E+07	European
CNTN2	rs11240341	chr1:205015284	2016	173480	2	Platelet count	T	C	0.338	-0.02727	0.003905	2.909E-12	3E+07	European
CNTN2	rs11240341	chr1:205015284	2016	173480	2	Red cell distribution width	T	C	0.338	0.02173	0.003791	0.00000001	3E+07	European
CNTN2	rs3767278	chr1:205015699	2016	173480	2	High light scatter reticulocyte count	T	C	0.662	-0.02306	0.003837	1.85E-09	3E+07	European
CNTN2	rs3767278	chr1:205015699	2016	173480	2	Immature fraction of reticulocytes	T	C	0.662	-0.02548	0.003801	2.029E-11	3E+07	European
CNTN2	rs3767278	chr1:205015699	2016	173480	2	Mean corpuscular hemoglobin	T	C	0.662	0.0328	0.00379	5E-18	3E+07	European
CNTN2	rs3767278	chr1:205015699	2016	173480	2	Mean corpuscular hemoglobin concentration	T	C	0.662	0.02312	0.003709	4.534E-10	3E+07	European
CNTN2	rs3767278	chr1:205015699	2016	173480	2	Mean corpuscular volume	T	C	0.662	0.02497	0.003777	3.844E-11	3E+07	European
CNTN2	rs3767278	chr1:205015699	2016	173480	2	Mean platelet volume	T	C	0.662	-0.07478	0.003868	2.989E-83	3E+07	European
CNTN2	rs3767278	chr1:205015699	2016	173480	2	Platelet count	T	C	0.662	0.02736	0.003902	2.365E-12	3E+07	European
CNTN2	rs3767278	chr1:205015699	2016	173480	2	Red cell distribution width	T	C	0.662	-0.02134	0.003789	1.778E-08	3E+07	European
CNTN2	rs12724651	chr1:205015717	2016	173480	2	Basophil count	T	C	0.506	-0.02171	0.003517	6.742E-10	3E+07	European
CNTN2	rs12724651	chr1:205015717	2016	173480	2	Basophil percentage of white cells	T	C	0.506	-0.01975	0.00349	1.52E-08	3E+07	European
CNTN2	rs12724651	chr1:205015717	2016	173480	2	High light scatter reticulocyte count	T	C	0.506	-0.02236	0.003602	5.385E-10	3E+07	European
CNTN2	rs12724651	chr1:205015717	2016	173480	2	High light scatter percentage of red cells	T	C	0.506	-0.02007	0.003602	2.524E-08	3E+07	European
CNTN2	rs12724651	chr1:205015717	2016	173480	2	Immature fraction of reticulocytes	T	C	0.506	-0.02416	0.003568	1.288E-11	3E+07	European
CNTN2	rs12724651	chr1:205015717	2016	173480	2	Mean corpuscular hemoglobin	T	C	0.506	0.03055	0.003558	9E-18	3E+07	European
CNTN2	rs12724651	chr1:205015717	2016	173480	2	Mean corpuscular hemoglobin concentration	T	C	0.506	0.0211	0.003482	1.356E-09	3E+07	European
CNTN2	rs12724651	chr1:205015717	2016	173480	2	Mean corpuscular volume	T	C	0.506	0.02408	0.003546	1.111E-11	3E+07	European
CNTN2	rs12724651	chr1:205015717	2016	173480	2	Mean platelet volume	T	C	0.506	-0.06393	0.003628	1.724E-69	3E+07	European
CNTN2	rs12724651	chr1:205015717	2016	173480	2	Platelet count	T	C	0.506	0.02222	0.003661	1.273E-09	3E+07	European
CNTN2	rs3767279	chr1:205015799	2016	173480	2	High light scatter reticulocyte count	A	T	0.338	0.02293	0.003838	2.298E-09	3E+07	European
CNTN2	rs3767279	chr1:205015799	2016	173480	2	Immature fraction of reticulocytes	A	T	0.338	0.0254	0.003802	2.399E-11	3E+07	European
CNTN2	rs3767279	chr1:205015799	2016	173480	2	Mean corpuscular hemoglobin	A	T	0.338	-0.03287	0.003791	4.274E-18	3E+07	European
CNTN2	rs3767279	chr1:205015799	2016	173480	2	Mean corpuscular hemoglobin concentration	A	T	0.338	-0.02352	0.00371	2.324E-10	3E+07	European
CNTN2	rs3767279	chr1:205015799	2016	173480	2	Mean corpuscular volume	A	T	0.338	-0.02487	0.003779	4.628E-11	3E+07	European
CNTN2	rs3767279	chr1:205015799	2016	173480	2	Mean platelet volume	A	T	0.338	0.07497	0.00387	1.288E-83	3E+07	European
CNTN2	rs3767279	chr1:205015799	2016	173480	2	Platelet distribution width	A	T	0.338	0.02102	0.003848	4.728E-08	3E+07	European
CNTN2	rs3767279	chr1:205015799	2016	173480	2	Platelet count	A	T	0.338	-0.02723	0.003904	3.042E-12	3E+07	European
CNTN2	rs3767279	chr1:205015799	2016	173480	2	Red cell distribution width	A	T	0.338	0.02151	0.00379	1.377E-08	3E+07	European
CNTN2	rs3767281	chr1:205015920	2016	173480	2	Basophil count	T	C	0.5149	-0.02135	0.003517	1.261E-09	3E+07	European
CNTN2	rs3767281	chr1:205015920	2016	173480	2	Basophil percentage of white cells	T	C	0.5149	-0.01937	0.00349	2.852E-08	3E+07	European
CNTN2	rs3767281	chr1:205015920	2016	173480	2	High light scatter reticulocyte count	T	C	0.5149	-0.02202	0.003601	9.694E-10	3E+07	European
CNTN2	rs3767281	chr1:205015920	2016	173480	2	High light scatter percentage of red cells	T	C	0.5149	-0.01968	0.003602	4.618E-08	3E+07	European
CNTN2	rs3767281	chr1:205015920	2016	173480	2	Immature fraction of reticulocytes	T	C	0.5149	-0.02354	0.003568	4.171E-11	3E+07	European
CNTN2	rs3767281	chr1:205015920	2016	173480	2	Mean corpuscular hemoglobin	T	C	0.5149	0.03101	0.003557	2.845E-18	3E+07	European
CNTN2	rs3767281	chr1:205015920	2016	173480	2	Mean corpuscular hemoglobin concentration	T	C	0.5149	0.02139	0.003482	8.134E-10	3E+07	European
CNTN2	rs3767281	chr1:205015920	2016	173480	2	Mean corpuscular volume	T	C	0.5149	0.02451	0.003546	4.769E-12	3E+07	European
CNTN2	rs3767281	chr1:205015920	2016	173480	2	Mean platelet volume	T	C	0.5149	-0.06437	0.003627	1.862E-70	3E+07	European
CNTN2	rs3767281	chr1:205015920	2016	173480	2	Platelet count	T	C	0.5149	0.02268	0.00366	5.804E-10	3E+07	European
CNTN2	rs3753854	chr1:205016698	2016	173480	2	Basophil count	A	C	0.494	0.02184	0.003513	5.061E-10	3E+07	European
CNTN2	rs3753854	chr1:205016698	2016	173480	2	Basophil percentage of white cells	A	C	0.494	0.01986	0.003486	1.221E-08	3E+07	European
CNTN2	rs3753854	chr1:205016698	2016	173480	2	High light scatter reticulocyte count	A	C	0.494	0.0222	0.003598	6.852E-10	3E+07	European
CNTN2	rs3753854	chr1:205016698	2016	173480	2	High light scatter percentage of red cells	A	C	0.494	0.01989	0.003598	3.242E-08	3E+07	European
CNTN2	rs3753854	chr1:205016698	2016	173480	2	Immature fraction of reticulocytes	A	C	0.494	0.02385	0.003564	2.204E-11	3E+07	European

CNTN2	rs3753854	chr1:205016698	2016	173480	2	Mean corpuscular hemoglobin	A	C	0.494	-0.03071	0.003554	5.556E-18	3E+07	European
CNTN2	rs3753854	chr1:205016698	2016	173480	2	Mean corpuscular hemoglobin concentration	A	C	0.494	-0.02143	0.003478	7.151E-10	3E+07	European
CNTN2	rs3753854	chr1:205016698	2016	173480	2	Mean corpuscular volume	A	C	0.494	-0.02408	0.003542	1.071E-11	3E+07	European
CNTN2	rs3753854	chr1:205016698	2016	173480	2	Mean platelet volume	A	C	0.494	0.06375	0.003624	2.842E-69	3E+07	European
CNTN2	rs3753854	chr1:205016698	2016	173480	2	Platelet count	A	C	0.494	-0.02184	0.003657	2.349E-09	3E+07	European
CNTN2	rs112193230	chr1:205017488	2017	7637	1	Cause of death: fatty liver	T	C	0.005	0.02845	0.004169	9.497E-12	UKBB	European
CNTN2	rs113117159	chr1:205017539	2017	7637	1	Cause of death: fatty liver	A	G	0.995	-0.02845	0.004169	9.535E-12	UKBB	European
CNTN2	rs116082426	chr1:205017828	2017	7637	1	Cause of death: urinary organ, unspecified	A	G	0.006	0.03084	0.004366	1.755E-12	UKBB	European
CNTN2	rs79392273	chr1:205017854	2016	173480	2	Mean corpuscular hemoglobin	T	C	0.8439	-0.0263	0.00479	4.004E-08	3E+07	European
CNTN2	rs79392273	chr1:205017854	2016	173480	2	Mean platelet volume	T	C	0.8439	0.08515	0.004874	2.453E-68	3E+07	European
CNTN2	rs79392273	chr1:205017854	2016	173480	2	Platelet count	T	C	0.8439	-0.03777	0.004921	1.654E-14	3E+07	European
CNTN2	rs867513	chr1:205017962	2017	7637	1	Cause of death: fatty liver	A	G	0.005	0.02843	0.004167	9.709E-12	UKBB	European
CNTN2	rs560774149	chr1:205018038	2016	173480	2	Mean platelet volume	C	CGT	0.6262	-0.03318	0.004053	2.701E-16	3E+07	European
CNTN2	rs3767284	chr1:205018314	2017	7637	1	Cause of death: fatty liver	A	G	0.995	-0.02937	0.004228	4.069E-12	UKBB	European
CNTN2	rs4996932	chr1:205018622	2016	173480	2	Basophil count	A	G	0.506	-0.02165	0.003506	6.582E-10	3E+07	European
CNTN2	rs4996932	chr1:205018622	2016	173480	2	Basophil percentage of white cells	A	G	0.506	-0.01982	0.003479	1.213E-08	3E+07	European
CNTN2	rs4996932	chr1:205018622	2016	173480	2	High light scatter reticulocyte count	A	G	0.506	-0.02229	0.00359	5.362E-10	3E+07	European
CNTN2	rs4996932	chr1:205018622	2016	173480	2	High light scatter percentage of red cells	A	G	0.506	-0.02003	0.00359	2.413E-08	3E+07	European
CNTN2	rs4996932	chr1:205018622	2016	173480	2	Immature fraction of reticulocytes	A	G	0.506	-0.02402	0.003557	1.452E-11	3E+07	European
CNTN2	rs4996932	chr1:205018622	2016	173480	2	Mean corpuscular hemoglobin	A	G	0.506	0.03051	0.003546	7.772E-18	3E+07	European
CNTN2	rs4996932	chr1:205018622	2016	173480	2	Mean corpuscular hemoglobin concentration	A	G	0.506	0.02122	0.003471	9.778E-10	3E+07	European
CNTN2	rs4996932	chr1:205018622	2016	173480	2	Mean corpuscular volume	A	G	0.506	0.02397	0.003535	1.202E-11	3E+07	European
CNTN2	rs4996932	chr1:205018622	2016	173480	2	Mean platelet volume	A	G	0.506	-0.06358	0.003617	3.566E-69	3E+07	European
CNTN2	rs4996932	chr1:205018622	2016	173480	2	Platelet count	A	G	0.506	0.02175	0.003649	2.502E-09	3E+07	European
CNTN2	rs3767287	chr1:205020703	2017	7637	1	Cause of death: fatty liver	A	G	0.005	0.02917	0.004216	4.944E-12	UKBB	European
CNTN2	rs200411079	chr1:205021956	2016	173480	2	Mean platelet volume	C	CT	0.0547	-0.09073	0.007847	6.464E-31	3E+07	European
CNTN2	rs201887646	chr1:205022260	2017	7637	1	Cause of death: aortic valve disorder, unspecified	A	T	0.997	-0.0283	0.003779	7.707E-14	UKBB	European
CNTN2	rs4951162	chr1:205022977	2016	173480	2	Mean platelet volume	A	G	0.663	-0.02419	0.003798	1.897E-10	3E+07	European
CNTN2	rs895932	chr1:205023014	2016	173480	2	Basophil count	A	G	0.5676	-0.0217	0.003521	7.145E-10	3E+07	European
CNTN2	rs895932	chr1:205023014	2016	173480	2	Basophil percentage of white cells	A	G	0.5676	-0.02003	0.003495	0.00000001	3E+07	European
CNTN2	rs895932	chr1:205023014	2016	173480	2	High light scatter reticulocyte count	A	G	0.5676	-0.01987	0.003606	3.591E-08	3E+07	European
CNTN2	rs895932	chr1:205023014	2016	173480	2	Immature fraction of reticulocytes	A	G	0.5676	-0.02233	0.003573	4.083E-10	3E+07	European
CNTN2	rs895932	chr1:205023014	2016	173480	2	Mean corpuscular hemoglobin	A	G	0.5676	0.02866	0.003562	8.551E-16	3E+07	European
CNTN2	rs895932	chr1:205023014	2016	173480	2	Mean corpuscular hemoglobin concentration	A	G	0.5676	0.02016	0.003487	7.363E-09	3E+07	European
CNTN2	rs895932	chr1:205023014	2016	173480	2	Mean corpuscular volume	A	G	0.5676	0.02227	0.003551	3.593E-10	3E+07	European
CNTN2	rs895932	chr1:205023014	2016	173480	2	Mean platelet volume	A	G	0.5676	-0.05936	0.003633	5.316E-60	3E+07	European
CNTN2	rs895932	chr1:205023014	2016	173480	2	Platelet count	A	G	0.5676	0.02095	0.003667	1.106E-08	3E+07	European
CNTN2	rs139811280	chr1:205023028	2016	173480	2	Mean platelet volume	T	TCTC	0.1819	0.04163	0.00467	4.968E-19	3E+07	European
CNTN2	rs12134459	chr1:205023349	2016	173480	2	Basophil count	T	C	0.506	-0.02151	0.003496	7.638E-10	3E+07	European
CNTN2	rs12134459	chr1:205023349	2016	173480	2	Basophil percentage of white cells	T	C	0.506	-0.01957	0.00347	1.705E-08	3E+07	European
CNTN2	rs12134459	chr1:205023349	2016	173480	2	High light scatter reticulocyte count	T	C	0.506	-0.02164	0.003581	1.511E-09	3E+07	European
CNTN2	rs12134459	chr1:205023349	2016	173480	2	Immature fraction of reticulocytes	T	C	0.506	-0.02345	0.003547	3.823E-11	3E+07	European
CNTN2	rs12134459	chr1:205023349	2016	173480	2	Mean corpuscular hemoglobin	T	C	0.506	0.03007	0.003537	1.894E-17	3E+07	European
CNTN2	rs12134459	chr1:205023349	2016	173480	2	Mean corpuscular hemoglobin concentration	T	C	0.506	0.0212	0.003462	9.115E-10	3E+07	European
CNTN2	rs12134459	chr1:205023349	2016	173480	2	Mean corpuscular volume	T	C	0.506	0.02345	0.003526	2.893E-11	3E+07	European
CNTN2	rs12134459	chr1:205023349	2016	173480	2	Mean platelet volume	T	C	0.506	-0.06344	0.003607	3.09E-69	3E+07	European
CNTN2	rs12134459	chr1:205023349	2016	173480	2	Platelet count	T	C	0.506	0.02161	0.00364	2.887E-09	3E+07	European
CNTN2	rs4951163	chr1:205023764	2016	173480	2	Mean corpuscular hemoglobin	T	C	0.0885	-0.03546	0.00645	3.824E-08	3E+07	European
CNTN2	rs4951163	chr1:205023764	2016	173480	2	Mean platelet volume	T	C	0.0885	0.09594	0.006603	7.973E-48	3E+07	European
CNTN2	rs4951163	chr1:205023764	2016	173480	2	Platelet count	T	C	0.0885	-0.04351	0.006655	6.211E-11	3E+07	European
CNTN2	rs4951163	chr1:205023764	2016	173480	2	Red cell distribution width	T	C	0.0885	0.03857	0.006448	2.205E-09	3E+07	European
CNTN2	rs1572995	chr1:205024012	2016	173480	2	Mean corpuscular hemoglobin	T	C	0.8598	0.03374	0.005272	1.557E-10	3E+07	European
CNTN2	rs1572995	chr1:205024012	2016	173480	2	Mean platelet volume	T	C	0.8598	-0.0904	0.005391	4.122E-63	3E+07	European
CNTN2	rs1572995	chr1:205024012	2016	173480	2	Platelet count	T	C	0.8598	0.03988	0.005435	2.171E-13	3E+07	European
CNTN2	rs11240344	chr1:205024302	2016	173480	2	Basophil count	T	C	0.505	-0.02153	0.003495	7.223E-10	3E+07	European
CNTN2	rs11240344	chr1:205024302	2016	173480	2	Basophil percentage of white cells	T	C	0.505	-0.0195	0.003468	1.894E-08	3E+07	European
CNTN2	rs11240344	chr1:205024302	2016	173480	2	High light scatter reticulocyte count	T	C	0.505	-0.0218	0.003579	1.126E-09	3E+07	European
CNTN2	rs11240344	chr1:205024302	2016	173480	2	High light scatter percentage of red cells	T	C	0.505	-0.0196	0.003579	4.354E-08	3E+07	European

CNTN2	rs11240344	chr1:205024302	2016	173480	2	Immature fraction of reticulocytes	T	C	0.505	-0.02368	0.003546	2.393E-11	3E+07	European
CNTN2	rs11240344	chr1:205024302	2016	173480	2	Mean corpuscular hemoglobin	T	C	0.505	0.02991	0.003535	2.65E-17	3E+07	European
CNTN2	rs11240344	chr1:205024302	2016	173480	2	Mean corpuscular hemoglobin concentration	T	C	0.505	0.02112	0.00346	1.026E-09	3E+07	European
CNTN2	rs11240344	chr1:205024302	2016	173480	2	Mean corpuscular volume	T	C	0.505	0.02331	0.003524	3.693E-11	3E+07	European
CNTN2	rs11240344	chr1:205024302	2016	173480	2	Mean platelet volume	T	C	0.505	-0.06348	0.003605	2.223E-69	3E+07	European
CNTN2	rs11240344	chr1:205024302	2016	173480	2	Platelet count	T	C	0.505	0.02147	0.003638	3.603E-09	3E+07	European
CNTN2	rs151222889	chr1:205024394	2017	7637	1	Cause of death: rheumatoid arthritis, unspecified	T	C	0.001	0.04226	0.007558	2.332E-08	UKBB	European
CNTN2	rs151222889	chr1:205024394	2017	337199	1	Acute and subacute endocarditis	T	C	0.001	0.003249	0.0005874	3.204E-08	UKBB	European
CNTN2	rs151222889	chr1:205024394	2017	337199	1	Fistulae involving female genital tract	T	C	0.001	0.003098	0.0005638	3.908E-08	UKBB	European
CNTN2	rs151222889	chr1:205024394	2017	337199	1	Injury of muscle and tendon at forearm level	T	C	0.001	0.00354	0.0006288	1.809E-08	UKBB	European
CNTN2	rs4950978	chr1:205024573	2016	173480	2	Mean platelet volume	T	C	0.2435	0.04269	0.004323	5.363E-23	3E+07	European
CNTN2	rs6656507	chr1:205024857	2016	173480	2	Basophil count	T	C	0.5209	-0.02065	0.003503	3.756E-09	3E+07	European
CNTN2	rs6656507	chr1:205024857	2016	173480	2	High light scatter reticulocyte count	T	C	0.5209	-0.02006	0.003587	2.226E-08	3E+07	European
CNTN2	rs6656507	chr1:205024857	2016	173480	2	Immature fraction of reticulocytes	T	C	0.5209	-0.02188	0.003554	7.433E-10	3E+07	European
CNTN2	rs6656507	chr1:205024857	2016	173480	2	Mean corpuscular hemoglobin	T	C	0.5209	0.02892	0.003543	3.303E-16	3E+07	European
CNTN2	rs6656507	chr1:205024857	2016	173480	2	Mean corpuscular hemoglobin concentration	T	C	0.5209	0.0208	0.003468	2.013E-09	3E+07	European
CNTN2	rs6656507	chr1:205024857	2016	173480	2	Mean corpuscular volume	T	C	0.5209	0.02225	0.003532	2.982E-10	3E+07	European
CNTN2	rs6656507	chr1:205024857	2016	173480	2	Mean platelet volume	T	C	0.5209	-0.06221	0.003614	2.169E-66	3E+07	European
CNTN2	rs6656507	chr1:205024857	2016	173480	2	Platelet count	T	C	0.5209	0.02199	0.003647	1.648E-09	3E+07	European
CNTN2	rs568322146	chr1:205026267	2017	333997	1	Home area population density: England or Wales - village, sparse	T	C	-	0.001682	0.0002885	5.562E-09	UKBB	European
CNTN2	rs568322146	chr1:205026267	2017	7637	1	Cause of death: heart failure, unspecified	T	C	-	0.09047	0.009816	3.914E-20	UKBB	European
CNTN2	rs568322146	chr1:205026267	2017	337199	1	Donors of organs and tissues	T	C	-	0.005703	0.0009718	4.408E-09	UKBB	European
CNTN2	rs537494132	chr1:205026426	2017	7637	1	Cause of death: malignant neoplasm of thyroid gland	C	G	-	0.08826	0.006865	1.954E-37	UKBB	European
CNTN2	rs112511985	chr1:205026454	2017	7637	1	Cause of death: fatty liver	T	C	0.995	-0.02915	0.004213	4.911E-12	UKBB	European
CNTN2	rs142502980	chr1:205028229	2017	337199	1	Other disorders of ear	T	C	0.008	0.001918	0.0003113	7.174E-10	UKBB	European
CNTN2	rs2305278	chr1:205028581	2016	173480	2	Mean platelet volume	C	G	0.8191	-0.04108	0.004674	1.494E-18	3E+07	European
CNTN2	rs35182846	chr1:205028883	2016	173480	2	Basophil count	C	CAG	0.505	-0.02136	0.00349	9.247E-10	3E+07	European
CNTN2	rs35182846	chr1:205028883	2016	173480	2	Basophil percentage of white cells	C	CAG	0.505	-0.01944	0.003463	1.983E-08	3E+07	European
CNTN2	rs35182846	chr1:205028883	2016	173480	2	High light scatter reticulocyte count	C	CAG	0.505	-0.02187	0.003574	9.387E-10	3E+07	European
CNTN2	rs35182846	chr1:205028883	2016	173480	2	High light scatter percentage of red cells	C	CAG	0.505	-0.01987	0.003574	2.714E-08	3E+07	European
CNTN2	rs35182846	chr1:205028883	2016	173480	2	Immature fraction of reticulocytes	C	CAG	0.505	-0.02387	0.00354	1.554E-11	3E+07	European
CNTN2	rs35182846	chr1:205028883	2016	173480	2	Mean corpuscular hemoglobin	C	CAG	0.505	0.02866	0.00353	4.71E-16	3E+07	European
CNTN2	rs35182846	chr1:205028883	2016	173480	2	Mean corpuscular hemoglobin concentration	C	CAG	0.505	0.02061	0.003455	2.45E-09	3E+07	European
CNTN2	rs35182846	chr1:205028883	2016	173480	2	Mean corpuscular volume	C	CAG	0.505	0.02216	0.003518	2.988E-10	3E+07	European
CNTN2	rs35182846	chr1:205028883	2016	173480	2	Mean platelet volume	C	CAG	0.505	-0.06316	0.0036	6.66E-69	3E+07	European
CNTN2	rs35182846	chr1:205028883	2016	173480	2	Platelet count	C	CAG	0.505	0.02173	0.003632	2.184E-09	3E+07	European
CNTN2	rs10793725	chr1:205028915	2016	173480	2	Basophil count	T	C	0.505	-0.02117	0.003485	1.25E-09	3E+07	European
CNTN2	rs10793725	chr1:205028915	2016	173480	2	Basophil percentage of white cells	T	C	0.505	-0.01921	0.003459	2.79E-08	3E+07	European
CNTN2	rs10793725	chr1:205028915	2016	173480	2	High light scatter reticulocyte count	T	C	0.505	-0.02193	0.003569	8.048E-10	3E+07	European
CNTN2	rs10793725	chr1:205028915	2016	173480	2	High light scatter percentage of red cells	T	C	0.505	-0.01988	0.003569	2.551E-08	3E+07	European
CNTN2	rs10793725	chr1:205028915	2016	173480	2	Immature fraction of reticulocytes	T	C	0.505	-0.02379	0.003536	1.725E-11	3E+07	European
CNTN2	rs10793725	chr1:205028915	2016	173480	2	Mean corpuscular hemoglobin	T	C	0.505	0.02868	0.003526	4.097E-16	3E+07	European
CNTN2	rs10793725	chr1:205028915	2016	173480	2	Mean corpuscular hemoglobin concentration	T	C	0.505	0.02075	0.00345	1.817E-09	3E+07	European
CNTN2	rs10793725	chr1:205028915	2016	173480	2	Mean corpuscular volume	T	C	0.505	0.02209	0.003514	3.238E-10	3E+07	European
CNTN2	rs10793725	chr1:205028915	2016	173480	2	Mean platelet volume	T	C	0.505	-0.06292	0.003596	1.432E-68	3E+07	European
CNTN2	rs10793725	chr1:205028915	2016	173480	2	Platelet count	T	C	0.505	0.02164	0.003628	2.44E-09	3E+07	European
CNTN2	rs186445665	chr1:205029790	2017	7637	1	Cause of death: pharynx, unspecified	A	G	0.005	0.04497	0.006459	3.635E-12	UKBB	European
CNTN2	rs11240345	chr1:205029854	2016	173480	2	Basophil count	A	G	0.496	0.02133	0.00349	9.829E-10	3E+07	European
CNTN2	rs11240345	chr1:205029854	2016	173480	2	Basophil percentage of white cells	A	G	0.496	0.0195	0.003463	1.782E-08	3E+07	European
CNTN2	rs11240345	chr1:205029854	2016	173480	2	High light scatter reticulocyte count	A	G	0.496	0.02177	0.003574	1.118E-09	3E+07	European
CNTN2	rs11240345	chr1:205029854	2016	173480	2	High light scatter percentage of red cells	A	G	0.496	0.0197	0.003574	3.515E-08	3E+07	European
CNTN2	rs11240345	chr1:205029854	2016	173480	2	Immature fraction of reticulocytes	A	G	0.496	0.02363	0.00354	2.456E-11	3E+07	European
CNTN2	rs11240345	chr1:205029854	2016	173480	2	Mean corpuscular hemoglobin	A	G	0.496	-0.02873	0.00353	3.956E-16	3E+07	European
CNTN2	rs11240345	chr1:205029854	2016	173480	2	Mean corpuscular hemoglobin concentration	A	G	0.496	-0.02102	0.003454	1.172E-09	3E+07	European
CNTN2	rs11240345	chr1:205029854	2016	173480	2	Mean corpuscular volume	A	G	0.496	-0.02199	0.003518	4.139E-10	3E+07	European
CNTN2	rs11240345	chr1:205029854	2016	173480	2	Mean platelet volume	A	G	0.496	0.06315	0.0036	7.009E-69	3E+07	European
CNTN2	rs11240345	chr1:205029854	2016	173480	2	Platelet count	A	G	0.496	-0.02173	0.003632	2.206E-09	3E+07	European
CNTN2	rs41264869	chr1:205030862	2016	173480	2	Mean platelet volume	T	C	0.1759	0.04442	0.004701	3.435E-21	3E+07	European

CNTN2	rs41264869	chr1:205030862	2017	-	-	Blood protein levels	T	C	0.1759	0.3885	0.05883	4E-11	3E+07	European
CNTN2	rs2229866	chr1:205031116	2016	173480	2	Mean platelet volume	T	C	0.339	0.02838	0.003786	6.626E-14	3E+07	European
CNTN2	rs563250304	chr1:205031339	2017	7637	1	Cause of death: larynx, unspecified	A	G	0.998	-0.06999	0.01265	3.302E-08	UKBB	European
CNTN2	rs563250304	chr1:205031339	2017	7637	1	Cause of death: thoracic aortic aneurysm, ruptured	A	G	0.998	-0.07155	0.0081	1.252E-18	UKBB	European
CNTN2	rs2242001	chr1:205031744	2016	173480	2	Mean platelet volume	A	G	0.1759	0.04382	0.004694	1.015E-20	3E+07	European
CNTN2	rs2242000	chr1:205031769	2016	173480	2	Mean platelet volume	A	G	0.1759	0.0438	0.004695	1.069E-20	3E+07	European
CNTN2	rs2242000	chr1:205031769	2016	-	-	Cerebrospinal fluid biomarker levels	A	G	0.1759	NA	NA	2E-13	3E+07	East Asian
CNTN2	rs4017876	chr1:205032793	2016	173480	2	Mean platelet volume	A	AAAAG	0.3459	0.02671	0.003777	1.534E-12	3E+07	European
CNTN2	rs11329962	chr1:205033254	2016	173480	2	Mean platelet volume	A	AG	0.3529	0.02518	0.003767	2.299E-11	3E+07	European
CNTN2	rs1470637	chr1:205033283	2016	173480	2	Mean platelet volume	A	G	0.3519	0.02478	0.003767	4.759E-11	3E+07	European
CNTN2	rs2305277	chr1:205033374	2016	173480	2	Mean platelet volume	T	C	0.661	-0.02842	0.003786	6.036E-14	3E+07	European
CNTN2	rs537446494	chr1:205034124	2017	7637	1	Cause of death: b-cell lymphoma, unspecified	T	C	0.001	0.1102	0.01885	5.183E-09	UKBB	European
CNTN2	rs148801784	chr1:205034390	2017	7637	1	Cause of death: cervix uteri, unspecified	A	G	-	0.04487	0.007371	1.204E-09	UKBB	European
CNTN2	rs4460698	chr1:205034488	2016	173480	2	Mean platelet volume	A	G	0.7097	-0.02279	0.003897	4.964E-09	3E+07	European
CNTN2	rs11588412	chr1:205034723	2016	173480	2	Mean platelet volume	T	C	0.3032	0.02378	0.00385	6.555E-10	3E+07	European
CNTN2	rs143128501	chr1:205034852	2016	173480	2	Basophil count	A	ATGGCCTGCT	0.498	0.02128	0.003494	1.128E-09	3E+07	European
CNTN2	rs143128501	chr1:205034852	2016	173480	2	Basophil percentage of white cells	A	ATGGCCTGCT	0.498	0.01955	0.003467	1.721E-08	3E+07	European
CNTN2	rs143128501	chr1:205034852	2016	173480	2	High light scatter reticulocyte count	A	ATGGCCTGCT	0.498	0.02197	0.003578	8.174E-10	3E+07	European
CNTN2	rs143128501	chr1:205034852	2016	173480	2	High light scatter percentage of red cells	A	ATGGCCTGCT	0.498	0.01987	0.003578	2.817E-08	3E+07	European
CNTN2	rs143128501	chr1:205034852	2016	173480	2	Immature fraction of reticulocytes	A	ATGGCCTGCT	0.498	0.02379	0.003545	1.91E-11	3E+07	European
CNTN2	rs143128501	chr1:205034852	2016	173480	2	Mean corpuscular hemoglobin	A	ATGGCCTGCT	0.498	-0.02886	0.003534	3.163E-16	3E+07	European
CNTN2	rs143128501	chr1:205034852	2016	173480	2	Mean corpuscular hemoglobin concentration	A	ATGGCCTGCT	0.498	-0.02117	0.003459	9.232E-10	3E+07	European
CNTN2	rs143128501	chr1:205034852	2016	173480	2	Mean corpuscular volume	A	ATGGCCTGCT	0.498	-0.02205	0.003523	3.832E-10	3E+07	European
CNTN2	rs143128501	chr1:205034852	2016	173480	2	Mean platelet volume	A	ATGGCCTGCT	0.498	0.06405	0.003604	1.22E-70	3E+07	European
CNTN2	rs143128501	chr1:205034852	2016	173480	2	Platelet count	A	ATGGCCTGCT	0.498	-0.02188	0.003637	1.794E-09	3E+07	European
CNTN2	rs16937	chr1:205035455	2016	173480	2	Basophil percentage of white cells	A	G	0.3241	0.02054	0.003741	3.995E-08	3E+07	European
CNTN2	rs16937	chr1:205035455	2016	173480	2	High light scatter reticulocyte count	A	G	0.3241	0.02352	0.00386	1.116E-09	3E+07	European
CNTN2	rs16937	chr1:205035455	2016	173480	2	High light scatter percentage of red cells	A	G	0.3241	0.02124	0.00386	3.737E-08	3E+07	European
CNTN2	rs16937	chr1:205035455	2016	173480	2	Immature fraction of reticulocytes	A	G	0.3241	0.02622	0.003824	7.11E-12	3E+07	European
CNTN2	rs16937	chr1:205035455	2016	173480	2	Mean corpuscular hemoglobin	A	G	0.3241	-0.03423	0.003813	2.773E-19	3E+07	European
CNTN2	rs16937	chr1:205035455	2016	173480	2	Mean corpuscular hemoglobin concentration	A	G	0.3241	-0.0244	0.003731	6.146E-11	3E+07	European
CNTN2	rs16937	chr1:205035455	2016	173480	2	Mean corpuscular volume	A	G	0.3241	-0.02578	0.0038	1.163E-11	3E+07	European
CNTN2	rs16937	chr1:205035455	2016	173480	2	Mean platelet volume	A	G	0.3241	0.07811	0.003893	1.62E-89	3E+07	European
CNTN2	rs16937	chr1:205035455	2016	173480	2	Platelet distribution width	A	G	0.3241	0.02133	0.003872	3.614E-08	3E+07	European
CNTN2	rs16937	chr1:205035455	2016	173480	2	Platelet count	A	G	0.3241	-0.02826	0.003927	6.162E-13	3E+07	European
CNTN2	rs16937	chr1:205035455	2016	173480	2	Red cell distribution width	A	G	0.3241	0.02335	0.003812	9.055E-10	3E+07	European
CNTN2	rs3767297	chr1:205036826	2016	173480	2	Mean corpuscular hemoglobin	A	G	0.16	-0.03251	0.005049	1.197E-10	3E+07	European
CNTN2	rs3767297	chr1:205036826	2016	173480	2	Mean platelet volume	A	G	0.16	0.08662	0.005163	3.562E-63	3E+07	European
CNTN2	rs3767297	chr1:205036826	2016	173480	2	Platelet count	A	G	0.16	-0.03479	0.005204	2.323E-11	3E+07	European
CNTN2	rs3767297	chr1:205036826	2016	173480	2	Red cell distribution width	A	G	0.16	0.02921	0.005046	7.068E-09	3E+07	European
CNTN2	rs535136236	chr1:205036858	2017	337159	1	Treatment with clarithromycin	C	G	-	-0.003196	0.0005603	1.17E-08	UKBB	European
CNTN2	rs535136236	chr1:205036858	2017	337159	1	Treatment with co-amoxiclav	C	G	-	-0.003051	0.0005567	4.221E-08	UKBB	European
CNTN2	rs535136236	chr1:205036858	2017	337159	1	Treatment with cetraben emollient cream	C	G	-	-0.003657	0.000553	3.799E-11	UKBB	European
CNTN2	rs3767298	chr1:205037354	2016	173480	2	Mean platelet volume	A	G	0.1799	0.04416	0.004678	3.747E-21	3E+07	European
CNTN2	rs6593920	chr1:205037676	2016	173480	2	Basophil count	T	G	0.5318	0.02446	0.003497	2.661E-12	3E+07	European
CNTN2	rs6593920	chr1:205037676	2016	173480	2	Basophil percentage of white cells	T	G	0.5318	0.02341	0.00347	1.515E-11	3E+07	European
CNTN2	rs6593920	chr1:205037676	2016	173480	2	Basophil percentage of granulocytes	T	G	0.5318	0.02059	0.003501	4.093E-09	3E+07	European
CNTN2	rs6593920	chr1:205037676	2016	173480	2	High light scatter reticulocyte count	T	G	0.5318	0.0248	0.003581	4.313E-12	3E+07	European
CNTN2	rs6593920	chr1:205037676	2016	173480	2	High light scatter percentage of red cells	T	G	0.5318	0.0223	0.003581	4.731E-10	3E+07	European
CNTN2	rs6593920	chr1:205037676	2016	173480	2	Immature fraction of reticulocytes	T	G	0.5318	0.02683	0.003548	3.978E-14	3E+07	European
CNTN2	rs6593920	chr1:205037676	2016	173480	2	Mean corpuscular hemoglobin	T	G	0.5318	-0.03246	0.003536	4.332E-20	3E+07	European
CNTN2	rs6593920	chr1:205037676	2016	173480	2	Mean corpuscular hemoglobin concentration	T	G	0.5318	-0.02625	0.003461	3.305E-14	3E+07	European
CNTN2	rs6593920	chr1:205037676	2016	173480	2	Mean corpuscular volume	T	G	0.5318	-0.02335	0.003525	3.513E-11	3E+07	European
CNTN2	rs6593920	chr1:205037676	2016	173480	2	Mean platelet volume	T	G	0.5318	0.07248	0.003605	6.75E-90	3E+07	European
CNTN2	rs6593920	chr1:205037676	2016	173480	2	Platelet count	T	G	0.5318	-0.02571	0.003638	1.571E-12	3E+07	European
CNTN2	rs769120613	chr1:205038158	2016	173480	2	Basophil count	T	TTTGTTTGTT	-	-0.02515	0.003828	5.022E-11	3E+07	European
CNTN2	rs769120613	chr1:205038158	2016	173480	2	Basophil percentage of white cells	T	TTTGTTTGTT	-	-0.02309	0.003799	1.207E-09	3E+07	European
CNTN2	rs769120613	chr1:205038158	2016	173480	2	High light scatter reticulocyte count	T	TTTGTTTGTT	-	-0.02595	0.003921	3.673E-11	3E+07	European

CNTN2	rs769120613	chr1:205038158	2016	173480	2	High light scatter percentage of red cells	T	TTTGTTTGTT	-	-0.02274	0.003921	6.658E-09	3E+07	European
CNTN2	rs769120613	chr1:205038158	2016	173480	2	Immature fraction of reticulocytes	T	TTTGTTTGTT	-	-0.02742	0.003885	1.695E-12	3E+07	European
CNTN2	rs769120613	chr1:205038158	2016	173480	2	Mean corpuscular hemoglobin	T	TTTGTTTGTT	-	0.03393	0.003873	1.938E-18	3E+07	European
CNTN2	rs769120613	chr1:205038158	2016	173480	2	Mean corpuscular hemoglobin concentration	T	TTTGTTTGTT	-	0.02557	0.003791	1.537E-11	3E+07	European
CNTN2	rs769120613	chr1:205038158	2016	173480	2	Mean corpuscular volume	T	TTTGTTTGTT	-	0.02546	0.003861	4.306E-11	3E+07	European
CNTN2	rs769120613	chr1:205038158	2016	173480	2	Mean platelet volume	T	TTTGTTTGTT	-	-0.07165	0.003945	1.009E-73	3E+07	European
CNTN2	rs769120613	chr1:205038158	2016	173480	2	Platelet count	T	TTTGTTTGTT	-	0.02607	0.003982	5.818E-11	3E+07	European
CNTN2	rs769120613	chr1:205038158	2016	173480	2	Red blood cell count	T	TTTGTTTGTT	-	-0.02215	0.003891	1.251E-08	3E+07	European
CNTN2	rs551907089	chr1:205038471	2017	7637	1	Cause of death: connective and soft tissue, unspecified	C	G	-	-0.05967	0.01011	3.765E-09	UKBB	European
CNTN2	rs551907089	chr1:205038471	2017	7637	1	Cause of death: home	C	G	-	-0.03059	0.00496	7.263E-10	UKBB	European
CNTN2	rs144056952	chr1:205038648	2017	7637	1	Cause of death: malignant neoplasm of ureter	A	G	0.001	0.04252	0.007153	2.887E-09	UKBB	European
CNTN2	rs144056952	chr1:205038648	2017	7637	1	Cause of death: fatty liver	A	G	0.001	0.04219	0.007146	3.702E-09	UKBB	European
CNTN2	rs2305275	chr1:205038731	2016	173480	2	Basophil percentage of white cells	T	C	-	-0.0237	0.00387	9.121E-10	3E+07	European
CNTN2	rs2305275	chr1:205038731	2016	173480	2	Basophil percentage of granulocytes	T	C	-	-0.0216	0.003904	3.151E-08	3E+07	European
CNTN2	rs2305275	chr1:205038731	2016	173480	2	High light scatter reticulocyte count	T	C	-	-0.02205	0.003993	3.331E-08	3E+07	European
CNTN2	rs2305275	chr1:205038731	2016	173480	2	Immature fraction of reticulocytes	T	C	-	-0.02471	0.003956	4.214E-10	3E+07	European
CNTN2	rs2305275	chr1:205038731	2016	173480	2	Mean corpuscular hemoglobin	T	C	-	0.0282	0.003944	8.667E-13	3E+07	European
CNTN2	rs2305275	chr1:205038731	2016	173480	2	Mean corpuscular volume	T	C	-	0.02198	0.003931	2.258E-08	3E+07	European
CNTN2	rs2305275	chr1:205038731	2016	173480	2	Mean platelet volume	T	C	-	-0.07456	0.004029	1.912E-76	3E+07	European
CNTN2	rs2305275	chr1:205038731	2016	173480	2	Platelet count	T	C	-	0.0307	0.004063	4.183E-14	3E+07	European
CNTN2	rs12134724	chr1:205039288	2016	173480	2	Basophil count	T	C	0.5427	0.02416	0.0035	5.066E-12	3E+07	European
CNTN2	rs12134724	chr1:205039288	2016	173480	2	Basophil percentage of white cells	T	C	0.5427	0.0243	0.003473	2.661E-12	3E+07	European
CNTN2	rs12134724	chr1:205039288	2016	173480	2	Basophil percentage of granulocytes	T	C	0.5427	0.02096	0.003504	2.224E-09	3E+07	European
CNTN2	rs12134724	chr1:205039288	2016	173480	2	High light scatter reticulocyte count	T	C	0.5427	0.03189	0.003585	5.813E-19	3E+07	European
CNTN2	rs12134724	chr1:205039288	2016	173480	2	High light scatter percentage of red cells	T	C	0.5427	0.02821	0.003585	3.595E-15	3E+07	European
CNTN2	rs12134724	chr1:205039288	2016	173480	2	Immature fraction of reticulocytes	T	C	0.5427	0.03226	0.003552	1.048E-19	3E+07	European
CNTN2	rs12134724	chr1:205039288	2016	173480	2	Mean corpuscular hemoglobin	T	C	0.5427	-0.04065	0.00354	1.654E-30	3E+07	European
CNTN2	rs12134724	chr1:205039288	2016	173480	2	Mean corpuscular hemoglobin concentration	T	C	0.5427	-0.02811	0.003464	4.895E-16	3E+07	European
CNTN2	rs12134724	chr1:205039288	2016	173480	2	Mean corpuscular volume	T	C	0.5427	-0.03168	0.003529	2.784E-19	3E+07	European
CNTN2	rs12134724	chr1:205039288	2016	173480	2	Mean platelet volume	T	C	0.5427	0.08907	0.003609	1.75E-134	3E+07	European
CNTN2	rs12134724	chr1:205039288	2016	173480	2	Platelet count	T	C	0.5427	-0.03239	0.003642	5.863E-19	3E+07	European
CNTN2	rs12134724	chr1:205039288	2016	173480	2	Red blood cell count	T	C	0.5427	0.02468	0.003557	3.933E-12	3E+07	European
CNTN2	rs12134724	chr1:205039288	2016	173480	2	Reticulocyte count	T	C	0.5427	0.02377	0.00359	3.557E-11	3E+07	European
CNTN2	rs56283998	chr1:205039295	2016	173480	2	Mean corpuscular hemoglobin	A	C	0.8082	0.02557	0.004414	6.936E-09	3E+07	European
CNTN2	rs56283998	chr1:205039295	2016	173480	2	Mean platelet volume	A	C	0.8082	-0.04251	0.004505	3.812E-21	3E+07	European
CNTN2	rs4951166	chr1:205040228	2016	173480	2	Basophil percentage of white cells	A	G	0.1441	0.02933	0.005185	1.545E-08	3E+07	European
CNTN2	rs4951166	chr1:205040228	2016	173480	2	Mean corpuscular hemoglobin	A	G	0.1441	-0.03492	0.005287	3.989E-11	3E+07	European
CNTN2	rs4951166	chr1:205040228	2016	173480	2	Mean platelet volume	A	G	0.1441	0.09683	0.005406	9.272E-72	3E+07	European
CNTN2	rs4951166	chr1:205040228	2016	173480	2	Platelet count	A	G	0.1441	-0.03973	0.005449	3.073E-13	3E+07	European
CNTN2	rs4951166	chr1:205040228	2016	173480	2	Red cell distribution width	A	G	0.1441	0.0295	0.005284	2.356E-08	3E+07	European
CNTN2	rs3753855	chr1:205040726	2016	173480	2	Basophil percentage of white cells	A	G	0.1521	0.02939	0.00515	1.145E-08	3E+07	European
CNTN2	rs3753855	chr1:205040726	2016	173480	2	Basophil percentage of granulocytes	A	G	0.1521	0.02863	0.005194	3.552E-08	3E+07	European
CNTN2	rs3753855	chr1:205040726	2016	173480	2	Mean corpuscular hemoglobin	A	G	0.1521	-0.03376	0.005251	1.289E-10	3E+07	European
CNTN2	rs3753855	chr1:205040726	2016	173480	2	Mean platelet volume	A	G	0.1521	0.0947	0.005371	1.36E-69	3E+07	European
CNTN2	rs3753855	chr1:205040726	2016	173480	2	Platelet count	A	G	0.1521	-0.0375	0.005413	4.31E-12	3E+07	European
CNTN2	rs3753855	chr1:205040726	2016	173480	2	Red cell distribution width	A	G	0.1521	0.0293	0.005248	2.355E-08	3E+07	European
CNTN2	rs3820338	chr1:205040835	2016	173480	2	Basophil percentage of white cells	A	G	0.1511	0.02884	0.005139	2.008E-08	3E+07	European
CNTN2	rs3820338	chr1:205040835	2016	173480	2	Mean corpuscular hemoglobin	A	G	0.1511	-0.03392	0.00524	9.598E-11	3E+07	European
CNTN2	rs3820338	chr1:205040835	2016	173480	2	Mean platelet volume	A	G	0.1511	0.0949	0.005359	3.635E-70	3E+07	European
CNTN2	rs3820338	chr1:205040835	2016	173480	2	Platelet count	A	G	0.1511	-0.03782	0.005402	2.517E-12	3E+07	European
CNTN2	rs3820338	chr1:205040835	2016	173480	2	Red cell distribution width	A	G	0.1511	0.0295	0.005237	1.763E-08	3E+07	European
CNTN2	rs12117194	chr1:205040943	2016	173480	2	Basophil count	C	G	0.4831	0.02503	0.003498	8.477E-13	3E+07	European
CNTN2	rs12117194	chr1:205040943	2016	173480	2	Basophil percentage of white cells	C	G	0.4831	0.02536	0.003472	2.771E-13	3E+07	European
CNTN2	rs12117194	chr1:205040943	2016	173480	2	Basophil percentage of granulocytes	C	G	0.4831	0.02212	0.003503	2.718E-10	3E+07	European
CNTN2	rs12117194	chr1:205040943	2016	173480	2	High light scatter reticulocyte count	C	G	0.4831	0.03044	0.003583	1.98E-17	3E+07	European
CNTN2	rs12117194	chr1:205040943	2016	173480	2	High light scatter percentage of red cells	C	G	0.4831	0.02717	0.003583	3.344E-14	3E+07	European
CNTN2	rs12117194	chr1:205040943	2016	173480	2	Immature fraction of reticulocytes	C	G	0.4831	0.03204	0.00355	1.757E-19	3E+07	European
CNTN2	rs12117194	chr1:205040943	2016	173480	2	Mean corpuscular hemoglobin	C	G	0.4831	-0.04002	0.003539	1.188E-29	3E+07	European

CNTN2	rs12117194	chr1:205040943	2016	173480	2	Mean corpuscular hemoglobin concentration	C	G	0.4831	-0.02865	0.003463	1.29E-16	3E+07	European
CNTN2	rs12117194	chr1:205040943	2016	173480	2	Mean corpuscular volume	C	G	0.4831	-0.03063	0.003527	3.804E-18	3E+07	European
CNTN2	rs12117194	chr1:205040943	2016	173480	2	Mean platelet volume	C	G	0.4831	0.08897	0.003608	2.83E-134	3E+07	European
CNTN2	rs12117194	chr1:205040943	2016	173480	2	Platelet count	C	G	0.4831	-0.03202	0.003641	1.418E-18	3E+07	European
CNTN2	rs12117194	chr1:205040943	2016	173480	2	Red blood cell count	C	G	0.4831	0.0223	0.003555	3.548E-10	3E+07	European
CNTN2	rs12117194	chr1:205040943	2016	173480	2	Reticulocyte count	C	G	0.4831	0.02163	0.003588	1.662E-09	3E+07	European
CNTN2	rs11240349	chr1:205041015	2016	173480	2	Basophil count	A	G	0.4573	-0.02446	0.003499	2.723E-12	3E+07	European
CNTN2	rs11240349	chr1:205041015	2016	173480	2	Basophil percentage of white cells	A	G	0.4573	-0.02445	0.003472	1.905E-12	3E+07	European
CNTN2	rs11240349	chr1:205041015	2016	173480	2	Basophil percentage of granulocytes	A	G	0.4573	-0.02101	0.003504	2.014E-09	3E+07	European
CNTN2	rs11240349	chr1:205041015	2016	173480	2	High light scatter reticulocyte count	A	G	0.4573	-0.03203	0.003584	3.977E-19	3E+07	European
CNTN2	rs11240349	chr1:205041015	2016	173480	2	High light scatter percentage of red cells	A	G	0.4573	-0.02832	0.003584	2.75E-15	3E+07	European
CNTN2	rs11240349	chr1:205041015	2016	173480	2	Immature fraction of reticulocytes	A	G	0.4573	-0.03256	0.003551	4.696E-20	3E+07	European
CNTN2	rs11240349	chr1:205041015	2016	173480	2	Mean corpuscular hemoglobin	A	G	0.4573	0.04078	0.00354	1.022E-30	3E+07	European
CNTN2	rs11240349	chr1:205041015	2016	173480	2	Mean corpuscular hemoglobin concentration	A	G	0.4573	0.02823	0.003464	3.59E-16	3E+07	European
CNTN2	rs11240349	chr1:205041015	2016	173480	2	Mean corpuscular volume	A	G	0.4573	0.0318	0.003528	1.98E-19	3E+07	European
CNTN2	rs11240349	chr1:205041015	2016	173480	2	Mean platelet volume	A	G	0.4573	-0.08947	0.003608	9.53E-136	3E+07	European
CNTN2	rs11240349	chr1:205041015	2016	173480	2	Platelet count	A	G	0.4573	0.03263	0.003641	3.169E-19	3E+07	European
CNTN2	rs11240349	chr1:205041015	2016	173480	2	Red blood cell count	A	G	0.4573	-0.02488	0.003556	2.596E-12	3E+07	European
CNTN2	rs11240349	chr1:205041015	2016	173480	2	Reticulocyte count	A	G	0.4573	-0.02376	0.003589	3.587E-11	3E+07	European
CNTN2	rs201687043	chr1:205041063	2017	7637	1	Cause of death: bronchiectasis	T	C	0.001	0.05038	0.009158	3.885E-08	UKBB	European
CNTN2	rs201687043	chr1:205041063	2017	337199	1	Other disorders of ear	T	C	0.001	0.002983	0.0004312	4.608E-12	UKBB	European
CNTN2	rs2229868	chr1:205041158	2016	173480	2	Mean platelet volume	T	C	0.1988	0.0306	0.004564	2.031E-11	3E+07	European
CNTN2	rs6662930	chr1:205041465	2016	173480	2	Basophil count	T	C	0.5427	0.02423	0.0035	4.497E-12	3E+07	European
CNTN2	rs6662930	chr1:205041465	2016	173480	2	Basophil percentage of white cells	T	C	0.5427	0.02425	0.003474	2.948E-12	3E+07	European
CNTN2	rs6662930	chr1:205041465	2016	173480	2	Basophil percentage of granulocytes	T	C	0.5427	0.02082	0.003505	2.831E-09	3E+07	European
CNTN2	rs6662930	chr1:205041465	2016	173480	2	High light scatter reticulocyte count	T	C	0.5427	0.03223	0.003585	2.445E-19	3E+07	European
CNTN2	rs6662930	chr1:205041465	2016	173480	2	High light scatter percentage of red cells	T	C	0.5427	0.02852	0.003585	1.78E-15	3E+07	European
CNTN2	rs6662930	chr1:205041465	2016	173480	2	Immature fraction of reticulocytes	T	C	0.5427	0.03267	0.003552	3.669E-20	3E+07	European
CNTN2	rs6662930	chr1:205041465	2016	173480	2	Mean corpuscular hemoglobin	T	C	0.5427	-0.04066	0.003541	1.605E-30	3E+07	European
CNTN2	rs6662930	chr1:205041465	2016	173480	2	Mean corpuscular hemoglobin concentration	T	C	0.5427	-0.02835	0.003465	2.808E-16	3E+07	European
CNTN2	rs6662930	chr1:205041465	2016	173480	2	Mean corpuscular volume	T	C	0.5427	-0.03156	0.003529	3.782E-19	3E+07	European
CNTN2	rs6662930	chr1:205041465	2016	173480	2	Mean platelet volume	T	C	0.5427	0.08951	0.003609	8.77E-136	3E+07	European
CNTN2	rs6662930	chr1:205041465	2016	173480	2	Platelet count	T	C	0.5427	-0.03281	0.003642	2.106E-19	3E+07	European
CNTN2	rs6662930	chr1:205041465	2016	173480	2	Red blood cell count	T	C	0.5427	0.02492	0.003557	2.431E-12	3E+07	European
CNTN2	rs6662930	chr1:205041465	2016	173480	2	Reticulocyte count	T	C	0.5427	0.02399	0.00359	2.342E-11	3E+07	European
CNTN2	rs56106622	chr1:205041480	2016	173480	2	Mean platelet volume	T	C	0.1412	0.03607	0.005014	6.242E-13	3E+07	European
CNTN2	rs10664521	chr1:205041487	2016	173480	2	Basophil percentage of white cells	A	ATGGG	0.1521	0.02861	0.005135	2.525E-08	3E+07	European
CNTN2	rs10664521	chr1:205041487	2016	173480	2	Mean corpuscular hemoglobin	A	ATGGG	0.1521	-0.03409	0.005236	7.43E-11	3E+07	European
CNTN2	rs10664521	chr1:205041487	2016	173480	2	Mean platelet volume	A	ATGGG	0.1521	0.09473	0.005355	4.933E-70	3E+07	European
CNTN2	rs10664521	chr1:205041487	2016	173480	2	Platelet count	A	ATGGG	0.1521	-0.03724	0.005397	5.209E-12	3E+07	European
CNTN2	rs10664521	chr1:205041487	2016	173480	2	Red cell distribution width	A	ATGGG	0.1521	0.02971	0.005232	1.365E-08	3E+07	European
CNTN2	rs3903399	chr1:205041542	2016	173480	2	Mean platelet volume	T	C	0.7942	-0.03503	0.004396	1.62E-15	3E+07	European
CNTN2	rs6696846	chr1:205041952	2016	173480	2	Basophil count	T	C	0.4702	-0.02366	0.003494	1.281E-11	3E+07	European
CNTN2	rs6696846	chr1:205041952	2016	173480	2	Basophil percentage of white cells	T	C	0.4702	-0.02327	0.003467	1.937E-11	3E+07	European
CNTN2	rs6696846	chr1:205041952	2016	173480	2	Basophil percentage of granulocytes	T	C	0.4702	-0.02013	0.003498	8.643E-09	3E+07	European
CNTN2	rs6696846	chr1:205041952	2016	173480	2	High light scatter reticulocyte count	T	C	0.4702	-0.03286	0.003578	4.182E-20	3E+07	European
CNTN2	rs6696846	chr1:205041952	2016	173480	2	High light scatter percentage of red cells	T	C	0.4702	-0.02915	0.003578	3.753E-16	3E+07	European
CNTN2	rs6696846	chr1:205041952	2016	173480	2	Immature fraction of reticulocytes	T	C	0.4702	-0.03393	0.003545	1.059E-21	3E+07	European
CNTN2	rs6696846	chr1:205041952	2016	173480	2	Mean corpuscular hemoglobin	T	C	0.4702	0.04182	0.003534	2.592E-32	3E+07	European
CNTN2	rs6696846	chr1:205041952	2016	173480	2	Mean corpuscular hemoglobin concentration	T	C	0.4702	0.02972	0.003458	8.23E-18	3E+07	European
CNTN2	rs6696846	chr1:205041952	2016	173480	2	Mean corpuscular volume	T	C	0.4702	0.03218	0.003522	6.492E-20	3E+07	European
CNTN2	rs6696846	chr1:205041952	2016	173480	2	Mean platelet volume	T	C	0.4702	-0.09303	0.003602	4.38E-147	3E+07	European
CNTN2	rs6696846	chr1:205041952	2016	173480	2	Platelet count	T	C	0.4702	0.03338	0.003635	4.235E-20	3E+07	European
CNTN2	rs6696846	chr1:205041952	2016	173480	2	Red blood cell count	T	C	0.4702	-0.02493	0.00355	2.193E-12	3E+07	European
CNTN2	rs6696846	chr1:205041952	2016	173480	2	Reticulocyte count	T	C	0.4702	-0.02398	0.003583	2.22E-11	3E+07	European
CNTN2	rs6696846	chr1:205041952	2016	-	-	Red blood cell count	T	C	0.4702	0.02493	0.003543	2E-12	3E+07	European
CNTN2	rs56338794	chr1:205042115	2016	173480	2	Mean platelet volume	T	C	0.1193	0.041	0.005436	4.634E-14	3E+07	European
CNTN2	rs1002926	chr1:205042583	2016	173480	2	Mean corpuscular hemoglobin	T	C	0.1918	-0.02555	0.004416	7.196E-09	3E+07	European

CNTN2	rs1002926	chr1:205042583	2016	173480	2	Mean platelet volume	T	C	0.1918	0.0426	0.004507	3.347E-21	3E+07	European
CNTN2	rs17416088	chr1:205042939	2016	173480	2	Mean platelet volume	T	C	0.166	0.04225	0.005004	3.06E-17	3E+07	European
CNTN2	rs1042831	chr1:205043250	2016	173480	2	Basophil count	C	G	0.4583	-0.02432	0.003497	3.564E-12	3E+07	European
CNTN2	rs1042831	chr1:205043250	2016	173480	2	Basophil percentage of white cells	C	G	0.4583	-0.02443	0.003471	1.944E-12	3E+07	European
CNTN2	rs1042831	chr1:205043250	2016	173480	2	Basophil percentage of granulocytes	C	G	0.4583	-0.021	0.003502	2.021E-09	3E+07	European
CNTN2	rs1042831	chr1:205043250	2016	173480	2	High light scatter reticulocyte count	C	G	0.4583	-0.03159	0.003582	1.157E-18	3E+07	European
CNTN2	rs1042831	chr1:205043250	2016	173480	2	High light scatter percentage of red cells	C	G	0.4583	-0.02791	0.003582	6.642E-15	3E+07	European
CNTN2	rs1042831	chr1:205043250	2016	173480	2	Immature fraction of reticulocytes	C	G	0.4583	-0.03224	0.003549	1.025E-19	3E+07	European
CNTN2	rs1042831	chr1:205043250	2016	173480	2	Mean corpuscular hemoglobin	C	G	0.4583	0.04044	0.003538	2.923E-30	3E+07	European
CNTN2	rs1042831	chr1:205043250	2016	173480	2	Mean corpuscular hemoglobin concentration	C	G	0.4583	0.02805	0.003462	5.362E-16	3E+07	European
CNTN2	rs1042831	chr1:205043250	2016	173480	2	Mean corpuscular volume	C	G	0.4583	0.03152	0.003526	3.916E-19	3E+07	European
CNTN2	rs1042831	chr1:205043250	2016	173480	2	Mean platelet volume	C	G	0.4583	-0.08931	0.003606	2.03E-135	3E+07	European
CNTN2	rs1042831	chr1:205043250	2016	173480	2	Platelet count	C	G	0.4583	0.0326	0.003639	3.325E-19	3E+07	European
CNTN2	rs1042831	chr1:205043250	2016	173480	2	Red blood cell count	C	G	0.4583	-0.0246	0.003554	4.437E-12	3E+07	European
CNTN2	rs1042831	chr1:205043250	2016	173480	2	Reticulocyte count	C	G	0.4583	-0.02335	0.003587	7.483E-11	3E+07	European
CNTN2	rs143419712	chr1:205043263	2016	173480	2	Mean platelet volume	A	G	0.0109	-0.156	0.01473	3.294E-26	3E+07	European
CNTN2	rs11240351	chr1:205044339	2016	173480	2	High light scatter reticulocyte count	A	G	0.5944	-0.02325	0.003646	1.817E-10	3E+07	European
CNTN2	rs11240351	chr1:205044339	2016	173480	2	High light scatter percentage of red cells	A	G	0.5944	-0.02083	0.003647	1.125E-08	3E+07	European
CNTN2	rs11240351	chr1:205044339	2016	173480	2	Immature fraction of reticulocytes	A	G	0.5944	-0.0234	0.003613	9.336E-11	3E+07	European
CNTN2	rs11240351	chr1:205044339	2016	173480	2	Mean corpuscular hemoglobin	A	G	0.5944	0.0277	0.003603	1.472E-14	3E+07	European
CNTN2	rs11240351	chr1:205044339	2016	173480	2	Mean corpuscular hemoglobin concentration	A	G	0.5944	0.02286	0.003525	8.822E-11	3E+07	European
CNTN2	rs11240351	chr1:205044339	2016	173480	2	Mean corpuscular volume	A	G	0.5944	0.0203	0.003591	1.58E-08	3E+07	European
CNTN2	rs11240351	chr1:205044339	2016	173480	2	Mean platelet volume	A	G	0.5944	-0.05573	0.003673	5.578E-52	3E+07	European
CNTN2	rs10900443	chr1:205044354	2016	173480	2	Basophil percentage of white cells	A	G	0.8489	-0.02893	0.005155	2.005E-08	3E+07	European
CNTN2	rs10900443	chr1:205044354	2016	173480	2	Basophil percentage of granulocytes	A	G	0.8489	-0.02835	0.005199	4.927E-08	3E+07	European
CNTN2	rs10900443	chr1:205044354	2016	173480	2	Mean corpuscular hemoglobin	A	G	0.8489	0.03421	0.005257	7.589E-11	3E+07	European
CNTN2	rs10900443	chr1:205044354	2016	173480	2	Mean platelet volume	A	G	0.8489	-0.09638	0.005376	6.952E-72	3E+07	European
CNTN2	rs10900443	chr1:205044354	2016	173480	2	Platelet count	A	G	0.8489	0.03825	0.005419	1.674E-12	3E+07	European
CNTN2	rs10900443	chr1:205044354	2016	173480	2	Red cell distribution width	A	G	0.8489	-0.02981	0.005253	1.391E-08	3E+07	European
CNTN2	rs10900444	chr1:205044416	2016	173480	2	Basophil percentage of white cells	A	C	0.8559	-0.02942	0.005182	1.371E-08	3E+07	European
CNTN2	rs10900444	chr1:205044416	2016	173480	2	Basophil percentage of granulocytes	A	C	0.8559	-0.02857	0.005226	4.561E-08	3E+07	European
CNTN2	rs10900444	chr1:205044416	2016	173480	2	Mean corpuscular hemoglobin	A	C	0.8559	0.03463	0.005284	5.598E-11	3E+07	European
CNTN2	rs10900444	chr1:205044416	2016	173480	2	Mean platelet volume	A	C	0.8559	-0.09689	0.005403	6.748E-72	3E+07	European
CNTN2	rs10900444	chr1:205044416	2016	173480	2	Platelet count	A	C	0.8559	0.03951	0.005447	4.062E-13	3E+07	European
CNTN2	rs10900444	chr1:205044416	2016	173480	2	Red cell distribution width	A	C	0.8559	-0.0293	0.005281	2.87E-08	3E+07	European
CNTN2	rs28447128	chr1:205044842	2016	173480	2	Mean corpuscular hemoglobin	T	C	0.8072	0.02563	0.004428	7.159E-09	3E+07	European
CNTN2	rs28447128	chr1:205044842	2016	173480	2	Mean platelet volume	T	C	0.8072	-0.04282	0.004519	2.644E-21	3E+07	European
CNTN2	rs7518906	chr1:205044866	2016	173480	2	Basophil percentage of white cells	C	G	0.8499	-0.02885	0.005147	2.082E-08	3E+07	European
CNTN2	rs7518906	chr1:205044866	2016	173480	2	Mean corpuscular hemoglobin	C	G	0.8499	0.03429	0.005248	6.385E-11	3E+07	European
CNTN2	rs7518906	chr1:205044866	2016	173480	2	Mean platelet volume	C	G	0.8499	-0.09618	0.005367	8.039E-72	3E+07	European
CNTN2	rs7518906	chr1:205044866	2016	173480	2	Platelet count	C	G	0.8499	0.03798	0.00541	2.216E-12	3E+07	European
CNTN2	rs7518906	chr1:205044866	2016	173480	2	Red cell distribution width	C	G	0.8499	-0.02974	0.005245	1.424E-08	3E+07	European
CNTN2	rs1572993	chr1:205045087	2016	173480	2	High light scatter reticulocyte count	A	G	0.4175	0.02213	0.003631	1.101E-09	3E+07	European
CNTN2	rs1572993	chr1:205045087	2016	173480	2	Immature fraction of reticulocytes	A	G	0.4175	0.02183	0.003597	1.285E-09	3E+07	European
CNTN2	rs1572993	chr1:205045087	2016	173480	2	Mean corpuscular hemoglobin	A	G	0.4175	-0.02634	0.003587	2.094E-13	3E+07	European
CNTN2	rs1572993	chr1:205045087	2016	173480	2	Mean corpuscular hemoglobin concentration	A	G	0.4175	-0.02084	0.00351	2.904E-09	3E+07	European
CNTN2	rs1572993	chr1:205045087	2016	173480	2	Mean corpuscular volume	A	G	0.4175	-0.01981	0.003576	3.029E-08	3E+07	European
CNTN2	rs1572993	chr1:205045087	2016	173480	2	Mean platelet volume	A	G	0.4175	0.05164	0.003657	2.865E-45	3E+07	European
CNTN2	rs141197327	chr1:205045570	2017	337159	1	Treatment with co-careldopa	C	G	0.005	0.002801	0.0004823	6.302E-09	UKBB	European
CNTN2	rs191205135	chr1:205046738	2017	337159	1	Treatment with antihistamine 60mg tablet	T	C	0.001	0.00566	0.0009759	6.633E-09	UKBB	European
CNTN2	rs16855045	chr1:205046894	2016	173480	2	Mean corpuscular hemoglobin	T	C	0.8082	0.0254	0.004413	8.613E-09	3E+07	European
CNTN2	rs16855045	chr1:205046894	2016	173480	2	Mean platelet volume	T	C	0.8082	-0.04294	0.004504	1.511E-21	3E+07	European
CNTN2	rs554763607	chr1:205047525	2017	337159	1	Treatment with lipantil micro 67mg capsule	A	G	0.002	0.002033	0.0003617	1.914E-08	UKBB	European
CNTN2	rs554763607	chr1:205047525	2017	337199	1	Viral warts	A	G	0.002	0.004409	0.0007405	2.624E-09	UKBB	European
CTSS	rs1136808	chr1:150702717	2017	307638	1	Forced vital capacity	C	G	0.4294	0.01096	0.002009	4.863E-08	UKBB	European
CTSS	rs1136808	chr1:150702717	2016	173480	2	Eosinophil percentage of granulocytes	C	G	0.4294	0.02153	0.003641	3.331E-09	3E+07	European
CTSS	rs1136808	chr1:150702717	2016	173480	2	Granulocyte percentage of myeloid white cells	C	G	0.4294	-0.04022	0.003634	1.828E-28	3E+07	European
CTSS	rs1136808	chr1:150702717	2016	173480	2	Monocyte count	C	G	0.4294	0.0385	0.003634	3.175E-26	3E+07	European

CTSS	rs1136808	chr1:150702717	2016	173480	2	Monocyte percentage of white cells	C	G	0.4294	0.03935	0.003624	1.794E-27	3E+07	European
CTSS	rs1136808	chr1:150702717	2016	173480	2	Neutrophil percentage of white cells	C	G	0.4294	-0.02861	0.003631	3.283E-15	3E+07	European
CTSS	rs1136808	chr1:150702717	2016	173480	2	Neutrophil percentage of granulocytes	C	G	0.4294	-0.02122	0.003641	5.601E-09	3E+07	European
CTSS	rs55960943	chr1:150703034	2016	173480	2	Granulocyte percentage of myeloid white cells	C	CAGA	0.4085	-0.03833	0.003654	9.466E-26	3E+07	European
CTSS	rs55960943	chr1:150703034	2016	173480	2	Monocyte count	C	CAGA	0.4085	0.03511	0.003653	7.272E-22	3E+07	European
CTSS	rs55960943	chr1:150703034	2016	173480	2	Monocyte percentage of white cells	C	CAGA	0.4085	0.03687	0.003643	4.427E-24	3E+07	European
CTSS	rs55960943	chr1:150703034	2016	173480	2	Neutrophil percentage of white cells	C	CAGA	0.4085	-0.02819	0.00365	1.128E-14	3E+07	European
CTSS	rs74743630	chr1:150703291	2016	173480	2	Eosinophil count	T	C	0.9791	-0.1016	0.01682	1.494E-09	3E+07	European
CTSS	rs74743630	chr1:150703291	2016	173480	2	Sum eosinophil basophil counts	T	C	0.9791	-0.1058	0.01684	3.357E-10	3E+07	European
CTSS	rs74743630	chr1:150703291	2016	173480	2	Eosinophil percentage of white cells	T	C	0.9791	-0.09367	0.01682	2.547E-08	3E+07	European
CTSS	rs74743630	chr1:150703291	2016	173480	2	Neutrophil percentage of granulocytes	T	C	0.9791	0.09292	0.01691	3.888E-08	3E+07	European
CTSS	rs56303945	chr1:150703366	2016	173480	2	Granulocyte percentage of myeloid white cells	T	TAACA	0.4085	-0.03816	0.003653	1.545E-25	3E+07	European
CTSS	rs56303945	chr1:150703366	2016	173480	2	Monocyte count	T	TAACA	0.4085	0.03507	0.003653	7.979E-22	3E+07	European
CTSS	rs56303945	chr1:150703366	2016	173480	2	Monocyte percentage of white cells	T	TAACA	0.4085	0.03672	0.003643	6.817E-24	3E+07	European
CTSS	rs56303945	chr1:150703366	2016	173480	2	Neutrophil percentage of white cells	T	TAACA	0.4085	-0.02808	0.00365	1.426E-14	3E+07	European
CTSS	rs55954760	chr1:150704215	2016	173480	2	Eosinophil count	T	C	0.9791	-0.102	0.01682	1.336E-09	3E+07	European
CTSS	rs55954760	chr1:150704215	2016	173480	2	Sum eosinophil basophil counts	T	C	0.9791	-0.1061	0.01684	2.97E-10	3E+07	European
CTSS	rs55954760	chr1:150704215	2016	173480	2	Eosinophil percentage of white cells	T	C	0.9791	-0.09403	0.01682	2.255E-08	3E+07	European
CTSS	rs55954760	chr1:150704215	2016	173480	2	Neutrophil percentage of granulocytes	T	C	0.9791	0.09332	0.01691	0.000000034	3E+07	European
CTSS	rs111733660	chr1:150705656	2016	173480	2	Eosinophil count	T	C	0.0219	0.09678	0.01642	3.741E-09	3E+07	European
CTSS	rs111733660	chr1:150705656	2016	173480	2	Sum eosinophil basophil counts	T	C	0.0219	0.09948	0.01645	1.456E-09	3E+07	European
CTSS	rs1415148	chr1:150705755	2016	173480	2	Eosinophil percentage of granulocytes	A	G	0.4284	0.02151	0.00364	3.425E-09	3E+07	European
CTSS	rs1415148	chr1:150705755	2016	173480	2	Granulocyte percentage of myeloid white cells	A	G	0.4284	-0.04007	0.003634	2.828E-28	3E+07	European
CTSS	rs1415148	chr1:150705755	2016	173480	2	Monocyte count	A	G	0.4284	0.03828	0.003633	5.928E-26	3E+07	European
CTSS	rs1415148	chr1:150705755	2016	173480	2	Monocyte percentage of white cells	A	G	0.4284	0.03922	0.003623	2.622E-27	3E+07	European
CTSS	rs1415148	chr1:150705755	2016	173480	2	Neutrophil percentage of white cells	A	G	0.4284	-0.02841	0.00363	5.026E-15	3E+07	European
CTSS	rs1415148	chr1:150705755	2016	173480	2	Neutrophil percentage of granulocytes	A	G	0.4284	-0.02114	0.003641	6.344E-09	3E+07	European
CTSS	rs115539008	chr1:150707381	2017	337159	1	Self-reported eczema or dermatitis	T	C	0.9881	-0.007539	0.001298	6.283E-09	UKBB	European
CTSS	rs75966112	chr1:150707572	2016	173480	2	Eosinophil count	T	G	0.0209	0.1018	0.01682	1.396E-09	3E+07	European
CTSS	rs75966112	chr1:150707572	2016	173480	2	Sum eosinophil basophil counts	T	G	0.0209	0.106	0.01684	3.067E-10	3E+07	European
CTSS	rs75966112	chr1:150707572	2016	173480	2	Eosinophil percentage of white cells	T	G	0.0209	0.09407	0.01682	2.222E-08	3E+07	European
CTSS	rs75966112	chr1:150707572	2016	173480	2	Neutrophil percentage of granulocytes	T	G	0.0209	-0.09356	0.01691	3.133E-08	3E+07	European
CTSS	rs7521898	chr1:150707596	2017	255492	1	Forced vital capacity, best measure	A	G	0.4076	0.01229	0.002216	2.926E-08	UKBB	European
CTSS	rs7521898	chr1:150707596	2017	307638	1	Forced vital capacity	A	G	0.4076	0.01189	0.00202	3.9E-09	UKBB	European
CTSS	rs7521898	chr1:150707596	2016	173480	2	Granulocyte percentage of myeloid white cells	A	G	0.4076	-0.03817	0.003653	1.492E-25	3E+07	European
CTSS	rs7521898	chr1:150707596	2016	173480	2	Monocyte count	A	G	0.4076	0.03502	0.003653	9.101E-22	3E+07	European
CTSS	rs7521898	chr1:150707596	2016	173480	2	Monocyte percentage of white cells	A	G	0.4076	0.03679	0.003643	5.507E-24	3E+07	European
CTSS	rs7521898	chr1:150707596	2016	173480	2	Neutrophil percentage of white cells	A	G	0.4076	-0.02796	0.00365	1.841E-14	3E+07	European
CTSS	rs7511673	chr1:150707636	2017	255492	1	Forced vital capacity, best measure	A	T	0.5924	-0.01232	0.002216	2.759E-08	UKBB	European
CTSS	rs7511673	chr1:150707636	2017	307638	1	Forced vital capacity	A	T	0.5924	-0.01192	0.00202	3.654E-09	UKBB	European
CTSS	rs7511673	chr1:150707636	2016	173480	2	Granulocyte percentage of myeloid white cells	A	T	0.5924	0.03817	0.003654	1.523E-25	3E+07	European
CTSS	rs7511673	chr1:150707636	2016	173480	2	Monocyte count	A	T	0.5924	-0.03501	0.003653	9.385E-22	3E+07	European
CTSS	rs7511673	chr1:150707636	2016	173480	2	Monocyte percentage of white cells	A	T	0.5924	-0.03677	0.003643	5.977E-24	3E+07	European
CTSS	rs7511673	chr1:150707636	2016	173480	2	Neutrophil percentage of white cells	A	T	0.5924	0.02799	0.00365	1.753E-14	3E+07	European
CTSS	rs112092350	chr1:150707783	2016	173480	2	Eosinophil count	T	C	0.9791	-0.1068	0.01733	7.051E-10	3E+07	European
CTSS	rs112092350	chr1:150707783	2016	173480	2	Sum eosinophil basophil counts	T	C	0.9791	-0.1107	0.01736	1.832E-10	3E+07	European
CTSS	rs112092350	chr1:150707783	2016	173480	2	Eosinophil percentage of white cells	T	C	0.9791	-0.09861	0.01733	1.267E-08	3E+07	European
CTSS	rs112092350	chr1:150707783	2016	173480	2	Eosinophil percentage of granulocytes	T	C	0.9791	-0.09579	0.01742	3.81E-08	3E+07	European
CTSS	rs112092350	chr1:150707783	2016	173480	2	Neutrophil percentage of granulocytes	T	C	0.9791	0.0976	0.01742	2.119E-08	3E+07	European
CTSS	rs72704603	chr1:150707992	2017	255492	1	Forced vital capacity, best measure	T	C	0.4076	0.0123	0.002217	2.86E-08	UKBB	European
CTSS	rs72704603	chr1:150707992	2017	307638	1	Forced vital capacity	T	C	0.4076	0.01191	0.00202	3.71E-09	UKBB	European
CTSS	rs72704603	chr1:150707992	2016	173480	2	Granulocyte percentage of myeloid white cells	T	C	0.4076	-0.03814	0.003653	1.657E-25	3E+07	European
CTSS	rs72704603	chr1:150707992	2016	173480	2	Monocyte count	T	C	0.4076	0.03503	0.003653	8.853E-22	3E+07	European
CTSS	rs72704603	chr1:150707992	2016	173480	2	Monocyte percentage of white cells	T	C	0.4076	0.03674	0.003643	6.328E-24	3E+07	European
CTSS	rs72704603	chr1:150707992	2016	173480	2	Neutrophil percentage of white cells	T	C	0.4076	-0.02797	0.00365	1.825E-14	3E+07	European
CTSS	rs587731206	chr1:150708231	2016	173480	2	Granulocyte percentage of myeloid white cells	A	AT	0.334	-0.03575	0.004314	1.165E-16	3E+07	European
CTSS	rs587731206	chr1:150708231	2016	173480	2	Monocyte count	A	AT	0.334	0.03127	0.004314	4.196E-13	3E+07	European
CTSS	rs587731206	chr1:150708231	2016	173480	2	Monocyte percentage of white cells	A	AT	0.334	0.03317	0.004302	1.245E-14	3E+07	European

CTSS	rs587731206	chr1:150708231	2016	173480	2	Neutrophil percentage of white cells	A	AT	0.334	-0.02785	0.004311	1.039E-10	3E+07	European
CTSS	rs11204717	chr1:150709129	2017	255492	1	Forced vital capacity, best measure	A	T	0.4076	0.01229	0.002217	2.927E-08	UKBB	European
CTSS	rs11204717	chr1:150709129	2017	307638	1	Forced vital capacity	A	T	0.4076	0.0119	0.00202	3.806E-09	UKBB	European
CTSS	rs11204717	chr1:150709129	2016	173480	2	Granulocyte percentage of myeloid white cells	A	T	0.4076	-0.03816	0.003654	1.579E-25	3E+07	European
CTSS	rs11204717	chr1:150709129	2016	173480	2	Monocyte count	A	T	0.4076	0.03501	0.003653	9.383E-22	3E+07	European
CTSS	rs11204717	chr1:150709129	2016	173480	2	Monocyte percentage of white cells	A	T	0.4076	0.03677	0.003643	5.975E-24	3E+07	European
CTSS	rs11204717	chr1:150709129	2016	173480	2	Neutrophil percentage of white cells	A	T	0.4076	-0.02801	0.00365	1.677E-14	3E+07	European
CTSS	rs587621048	chr1:150709248	2016	173480	2	Eosinophil count	T	TA	0.9722	-0.08532	0.01494	1.128E-08	3E+07	European
CTSS	rs587621048	chr1:150709248	2016	173480	2	Sum eosinophil basophil counts	T	TA	0.9722	-0.08967	0.01496	2.051E-09	3E+07	European
CTSS	rs6587520	chr1:150709723	2017	255492	1	Forced vital capacity, best measure	T	C	0.4761	-0.01556	0.002165	6.495E-13	UKBB	European
CTSS	rs6587520	chr1:150709723	2017	307638	1	Forced vital capacity	T	C	0.4761	-0.01234	0.001972	3.887E-10	UKBB	European
CTSS	rs6587520	chr1:150709723	2016	173480	2	Eosinophil count	T	C	0.4761	-0.02046	0.003562	9.221E-09	3E+07	European
CTSS	rs6587520	chr1:150709723	2016	173480	2	Eosinophil percentage of granulocytes	T	C	0.4761	-0.02278	0.003574	1.82E-10	3E+07	European
CTSS	rs6587520	chr1:150709723	2016	173480	2	Granulocyte percentage of myeloid white cells	T	C	0.4761	0.04541	0.003567	3.951E-37	3E+07	European
CTSS	rs6587520	chr1:150709723	2016	173480	2	Lymphocyte count	T	C	0.4761	-0.02216	0.003596	7.192E-10	3E+07	European
CTSS	rs6587520	chr1:150709723	2016	173480	2	Monocyte count	T	C	0.4761	-0.04447	0.003567	1.109E-35	3E+07	European
CTSS	rs6587520	chr1:150709723	2016	173480	2	Monocyte percentage of white cells	T	C	0.4761	-0.04493	0.003557	1.388E-36	3E+07	European
CTSS	rs6587520	chr1:150709723	2016	173480	2	Neutrophil percentage of white cells	T	C	0.4761	0.03092	0.003564	4.056E-18	3E+07	European
CTSS	rs6587520	chr1:150709723	2016	173480	2	Neutrophil percentage of granulocytes	T	C	0.4761	0.02237	0.003574	3.865E-10	3E+07	European
CTSS	rs11204718	chr1:150709785	2017	255492	1	Forced vital capacity, best measure	C	G	0.5924	-0.01229	0.002217	2.913E-08	UKBB	European
CTSS	rs11204718	chr1:150709785	2017	307638	1	Forced vital capacity	C	G	0.5924	-0.01189	0.00202	3.914E-09	UKBB	European
CTSS	rs11204718	chr1:150709785	2016	173480	2	Granulocyte percentage of myeloid white cells	C	G	0.5924	0.03803	0.003653	2.219E-25	3E+07	European
CTSS	rs11204718	chr1:150709785	2016	173480	2	Monocyte count	C	G	0.5924	-0.03485	0.003653	1.408E-21	3E+07	European
CTSS	rs11204718	chr1:150709785	2016	173480	2	Monocyte percentage of white cells	C	G	0.5924	-0.03664	0.003643	8.516E-24	3E+07	European
CTSS	rs11204718	chr1:150709785	2016	173480	2	Neutrophil percentage of white cells	C	G	0.5924	0.02794	0.00365	1.936E-14	3E+07	European
CTSS	rs74828331	chr1:150710286	2016	173480	2	Eosinophil count	A	G	0.9791	-0.1017	0.01682	1.486E-09	3E+07	European
CTSS	rs74828331	chr1:150710286	2016	173480	2	Sum eosinophil basophil counts	A	G	0.9791	-0.1061	0.01685	3.042E-10	3E+07	European
CTSS	rs74828331	chr1:150710286	2016	173480	2	Eosinophil percentage of white cells	A	G	0.9791	-0.09379	0.01682	2.456E-08	3E+07	European
CTSS	rs74828331	chr1:150710286	2016	173480	2	Neutrophil percentage of granulocytes	A	G	0.9791	0.09334	0.01691	3.381E-08	3E+07	European
CTSS	rs113967546	chr1:150711557	2016	173480	2	Eosinophil count	T	C	0.9791	-0.102	0.01682	1.341E-09	3E+07	European
CTSS	rs113967546	chr1:150711557	2016	173480	2	Sum eosinophil basophil counts	T	C	0.9791	-0.1062	0.01685	2.942E-10	3E+07	European
CTSS	rs113967546	chr1:150711557	2016	173480	2	Eosinophil percentage of white cells	T	C	0.9791	-0.09416	0.01682	2.18E-08	3E+07	European
CTSS	rs113967546	chr1:150711557	2016	173480	2	Neutrophil percentage of granulocytes	T	C	0.9791	0.09364	0.01691	3.087E-08	3E+07	European
CTSS	rs181047033	chr1:150711804	2017	337199	1	Fracture of neck	A	T	1	-0.002925	0.0005235	2.301E-08	UKBB	European
CTSS	rs7418501	chr1:150711896	2016	173480	2	Eosinophil percentage of granulocytes	A	T	0.5716	-0.02159	0.00364	3.018E-09	3E+07	European
CTSS	rs7418501	chr1:150711896	2016	173480	2	Granulocyte percentage of myeloid white cells	A	T	0.5716	0.04019	0.003633	1.927E-28	3E+07	European
CTSS	rs7418501	chr1:150711896	2016	173480	2	Monocyte count	A	T	0.5716	-0.03839	0.003633	4.267E-26	3E+07	European
CTSS	rs7418501	chr1:150711896	2016	173480	2	Monocyte percentage of white cells	A	T	0.5716	-0.03939	0.003623	1.567E-27	3E+07	European
CTSS	rs7418501	chr1:150711896	2016	173480	2	Neutrophil percentage of white cells	A	T	0.5716	0.02848	0.00363	4.328E-15	3E+07	European
CTSS	rs7418501	chr1:150711896	2016	173480	2	Neutrophil percentage of granulocytes	A	T	0.5716	0.02119	0.00364	5.88E-09	3E+07	European
CTSS	rs72273354	chr1:150712372	2016	173480	2	Granulocyte percentage of myeloid white cells	G	GTTC	0.4076	-0.03825	0.003656	1.291E-25	3E+07	European
CTSS	rs72273354	chr1:150712372	2016	173480	2	Monocyte count	G	GTTC	0.4076	0.03507	0.003656	8.494E-22	3E+07	European
CTSS	rs72273354	chr1:150712372	2016	173480	2	Monocyte percentage of white cells	G	GTTC	0.4076	0.03686	0.003646	4.998E-24	3E+07	European
CTSS	rs72273354	chr1:150712372	2016	173480	2	Neutrophil percentage of white cells	G	GTTC	0.4076	-0.02804	0.003653	1.63E-14	3E+07	European
CTSS	rs111842513	chr1:150712926	2017	255492	1	Forced vital capacity, best measure	A	G	0.4076	0.01232	0.002217	2.746E-08	UKBB	European
CTSS	rs111842513	chr1:150712926	2017	307638	1	Forced vital capacity	A	G	0.4076	0.01191	0.00202	3.688E-09	UKBB	European
CTSS	rs111842513	chr1:150712926	2016	173480	2	Granulocyte percentage of myeloid white cells	A	G	0.4076	-0.03823	0.003654	1.306E-25	3E+07	European
CTSS	rs111842513	chr1:150712926	2016	173480	2	Monocyte count	A	G	0.4076	0.03505	0.003654	8.585E-22	3E+07	European
CTSS	rs111842513	chr1:150712926	2016	173480	2	Monocyte percentage of white cells	A	G	0.4076	0.03682	0.003643	5.243E-24	3E+07	European
CTSS	rs111842513	chr1:150712926	2016	173480	2	Neutrophil percentage of white cells	A	G	0.4076	-0.02803	0.00365	1.603E-14	3E+07	European
CTSS	rs34826479	chr1:150714360	2016	173480	2	Granulocyte percentage of myeloid white cells	A	AG	0.493	0.04313	0.003576	1.66E-33	3E+07	European
CTSS	rs34826479	chr1:150714360	2016	173480	2	Lymphocyte count	A	AG	0.493	-0.02167	0.003605	1.848E-09	3E+07	European
CTSS	rs34826479	chr1:150714360	2016	173480	2	Lymphocyte percentage of white cells	A	AG	0.493	-0.01993	0.003567	2.307E-08	3E+07	European
CTSS	rs34826479	chr1:150714360	2016	173480	2	Monocyte count	A	AG	0.493	-0.04075	0.003575	4.314E-30	3E+07	European
CTSS	rs34826479	chr1:150714360	2016	173480	2	Monocyte percentage of white cells	A	AG	0.493	-0.04208	0.003566	3.844E-32	3E+07	European
CTSS	rs34826479	chr1:150714360	2016	173480	2	Neutrophil percentage of white cells	A	AG	0.493	0.03043	0.003573	1.63E-17	3E+07	European
CTSS	rs587659692	chr1:150714367	2016	173480	2	Eosinophil count	C	G	0.0209	0.1017	0.01682	1.485E-09	3E+07	European
CTSS	rs587659692	chr1:150714367	2016	173480	2	Sum eosinophil basophil counts	C	G	0.0209	0.1059	0.01685	3.267E-10	3E+07	European

CTSS	rs587659692	chr1:150714367	2016	173480	2	Eosinophil percentage of white cells	C	G	0.0209	0.09389	0.01682	2.395E-08	3E+07	European
CTSS	rs587659692	chr1:150714367	2016	173480	2	Neutrophil percentage of granulocytes	C	G	0.0209	-0.09336	0.01691	3.392E-08	3E+07	European
CTSS	rs113147464	chr1:150714651	2016	173480	2	Eosinophil count	T	TGAACCTTA/	0.4751	-0.02024	0.003566	1.392E-08	3E+07	European
CTSS	rs113147464	chr1:150714651	2016	173480	2	Eosinophil percentage of granulocytes	T	TGAACCTTA/	0.4751	-0.02269	0.003578	2.3E-10	3E+07	European
CTSS	rs113147464	chr1:150714651	2016	173480	2	Granulocyte percentage of myeloid white cells	T	TGAACCTTA/	0.4751	0.04542	0.003571	4.72E-37	3E+07	European
CTSS	rs113147464	chr1:150714651	2016	173480	2	Lymphocyte count	T	TGAACCTTA/	0.4751	-0.02199	0.003601	1.011E-09	3E+07	European
CTSS	rs113147464	chr1:150714651	2016	173480	2	Monocyte count	T	TGAACCTTA/	0.4751	-0.04415	0.003571	4.241E-35	3E+07	European
CTSS	rs113147464	chr1:150714651	2016	173480	2	Monocyte percentage of white cells	T	TGAACCTTA/	0.4751	-0.04494	0.003561	1.686E-36	3E+07	European
CTSS	rs113147464	chr1:150714651	2016	173480	2	Neutrophil percentage of white cells	T	TGAACCTTA/	0.4751	0.03112	0.003568	2.748E-18	3E+07	European
CTSS	rs113147464	chr1:150714651	2016	173480	2	Neutrophil percentage of granulocytes	T	TGAACCTTA/	0.4751	0.0223	0.003579	4.652E-10	3E+07	European
CTSS	rs113650982	chr1:150715668	2016	173480	2	Eosinophil count	T	G	0.0209	0.1021	0.01682	1.251E-09	3E+07	European
CTSS	rs113650982	chr1:150715668	2016	173480	2	Sum eosinophil basophil counts	T	G	0.0209	0.1064	0.01685	2.703E-10	3E+07	European
CTSS	rs113650982	chr1:150715668	2016	173480	2	Eosinophil percentage of white cells	T	G	0.0209	0.09426	0.01682	2.098E-08	3E+07	European
CTSS	rs113650982	chr1:150715668	2016	173480	2	Neutrophil percentage of granulocytes	T	G	0.0209	-0.09368	0.01691	3.028E-08	3E+07	European
CTSS	rs12089989	chr1:150715782	2016	173480	2	Eosinophil percentage of granulocytes	A	G	0.4284	0.02162	0.00364	2.868E-09	3E+07	European
CTSS	rs12089989	chr1:150715782	2016	173480	2	Granulocyte percentage of myeloid white cells	A	G	0.4284	-0.04016	0.003633	2.109E-28	3E+07	European
CTSS	rs12089989	chr1:150715782	2016	173480	2	Monocyte count	A	G	0.4284	0.03811	0.003633	9.559E-26	3E+07	European
CTSS	rs12089989	chr1:150715782	2016	173480	2	Monocyte percentage of white cells	A	G	0.4284	0.03927	0.003623	2.25E-27	3E+07	European
CTSS	rs12089989	chr1:150715782	2016	173480	2	Neutrophil percentage of white cells	A	G	0.4284	-0.02868	0.003629	2.729E-15	3E+07	European
CTSS	rs12089989	chr1:150715782	2016	173480	2	Neutrophil percentage of granulocytes	A	G	0.4284	-0.02116	0.00364	6.169E-09	3E+07	European
CTSS	rs35375032	chr1:150716980	2016	173480	2	Eosinophil count	T	TA	0.2416	-0.024	0.004327	2.934E-08	3E+07	European
CTSS	rs35375032	chr1:150716980	2016	173480	2	Sum eosinophil basophil counts	T	TA	0.2416	-0.02486	0.004333	9.67E-09	3E+07	European
CTSS	rs35375032	chr1:150716980	2016	173480	2	Granulocyte percentage of myeloid white cells	T	TA	0.2416	0.0554	0.004337	2.289E-37	3E+07	European
CTSS	rs35375032	chr1:150716980	2016	173480	2	Monocyte count	T	TA	0.2416	-0.06114	0.004335	3.569E-45	3E+07	European
CTSS	rs35375032	chr1:150716980	2016	173480	2	Monocyte percentage of white cells	T	TA	0.2416	-0.05674	0.004323	2.419E-39	3E+07	European
CTSS	rs35375032	chr1:150716980	2016	173480	2	Neutrophil percentage of white cells	T	TA	0.2416	0.02988	0.004331	5.174E-12	3E+07	European
CTSS	rs35375032	chr1:150716980	2016	173480	2	Neutrophil percentage of granulocytes	T	TA	0.2416	0.02419	0.004345	2.57E-08	3E+07	European
CTSS	rs184794711	chr1:150717013	2016	173480	2	Eosinophil count	A	G	0.9791	-0.09946	0.01674	2.836E-09	3E+07	European
CTSS	rs184794711	chr1:150717013	2016	173480	2	Sum eosinophil basophil counts	A	G	0.9791	-0.1044	0.01677	4.885E-10	3E+07	European
CTSS	rs184794711	chr1:150717013	2016	173480	2	Eosinophil percentage of white cells	A	G	0.9791	-0.09179	0.01674	4.203E-08	3E+07	European
CTSS	rs184794711	chr1:150717013	2016	173480	2	Neutrophil percentage of granulocytes	A	G	0.9791	0.09216	0.01683	4.388E-08	3E+07	European
CTSS	rs11810015	chr1:150717080	2017	336601	1	Hip circumference	A	G	-	0.02494	0.00457	4.808E-08	UKBB	European
CTSS	rs34813433	chr1:150717100	2016	173480	2	Eosinophil percentage of granulocytes	T	TATA	0.5706	-0.02121	0.003644	5.882E-09	3E+07	European
CTSS	rs34813433	chr1:150717100	2016	173480	2	Granulocyte percentage of myeloid white cells	T	TATA	0.5706	0.0396	0.003637	1.308E-27	3E+07	European
CTSS	rs34813433	chr1:150717100	2016	173480	2	Monocyte count	T	TATA	0.5706	-0.03773	0.003637	3.199E-25	3E+07	European
CTSS	rs34813433	chr1:150717100	2016	173480	2	Monocyte percentage of white cells	T	TATA	0.5706	-0.03851	0.003627	2.431E-26	3E+07	European
CTSS	rs34813433	chr1:150717100	2016	173480	2	Neutrophil percentage of white cells	T	TATA	0.5706	0.02863	0.003634	3.303E-15	3E+07	European
CTSS	rs34813433	chr1:150717100	2016	173480	2	Neutrophil percentage of granulocytes	T	TATA	0.5706	0.02087	0.003644	1.015E-08	3E+07	European
CTSS	rs72704611	chr1:150717488	2017	336172	1	Sitting height	T	C	0.9861	-0.06401	0.008177	4.94E-15	UKBB	European
CTSS	rs72704611	chr1:150717488	2017	255492	1	Forced expiratory volume in 1-second, best measure	T	C	0.9861	-0.06597	0.009933	3.116E-11	UKBB	European
CTSS	rs72704611	chr1:150717488	2017	255492	1	Forced vital capacity, best measure	T	C	0.9861	-0.07645	0.009405	4.375E-16	UKBB	European
CTSS	rs72704611	chr1:150717488	2017	331285	1	Leg fat-free mass right	T	C	0.9861	-0.03926	0.00696	1.699E-08	UKBB	European
CTSS	rs72704611	chr1:150717488	2017	331285	1	Leg predicted mass right	T	C	0.9861	-0.03965	0.006915	9.832E-09	UKBB	European
CTSS	rs72704611	chr1:150717488	2017	331258	1	Leg fat-free mass left	T	C	0.9861	-0.03862	0.006963	2.907E-08	UKBB	European
CTSS	rs72704611	chr1:150717488	2017	331253	1	Leg predicted mass left	T	C	0.9861	-0.03775	0.006916	4.796E-08	UKBB	European
CTSS	rs72704611	chr1:150717488	2017	307638	1	Forced vital capacity	T	C	0.9861	-0.06695	0.008574	5.804E-15	UKBB	European
CTSS	rs72704611	chr1:150717488	2017	307638	1	Forced expiratory volume in 1-second	T	C	0.9861	-0.05885	0.009043	7.627E-11	UKBB	European
CTSS	rs72704611	chr1:150717488	2017	307638	1	Peak expiratory flow	T	C	0.9861	-0.05246	0.009399	2.382E-08	UKBB	European
CTSS	rs72704611	chr1:150717488	2017	336474	1	Height	T	C	0.9861	-0.07031	0.007518	8.563E-21	UKBB	European
CTSS	rs74592968	chr1:150717551	2016	173480	2	Eosinophil count	T	C	0.0209	0.102	0.01683	1.364E-09	3E+07	European
CTSS	rs74592968	chr1:150717551	2016	173480	2	Sum eosinophil basophil counts	T	C	0.0209	0.1064	0.01686	2.85E-10	3E+07	European
CTSS	rs74592968	chr1:150717551	2016	173480	2	Eosinophil percentage of white cells	T	C	0.0209	0.0944	0.01684	2.059E-08	3E+07	European
CTSS	rs74592968	chr1:150717551	2016	173480	2	Neutrophil percentage of granulocytes	T	C	0.0209	-0.0942	0.01693	2.612E-08	3E+07	European
CTSS	rs187694055	chr1:150718363	2017	337159	1	Treatment with oramorph 10mg or 5ml oral solution	T	G	0.999	-0.001808	0.0002954	9.433E-10	UKBB	European
CTSS	rs80026546	chr1:150718389	2016	173480	2	Eosinophil count	A	G	0.9791	-0.1029	0.01683	9.788E-10	3E+07	European
CTSS	rs80026546	chr1:150718389	2016	173480	2	Sum eosinophil basophil counts	A	G	0.9791	-0.1072	0.01686	2.051E-10	3E+07	European
CTSS	rs80026546	chr1:150718389	2016	173480	2	Eosinophil percentage of white cells	A	G	0.9791	-0.0952	0.01683	1.555E-08	3E+07	European
CTSS	rs80026546	chr1:150718389	2016	173480	2	Eosinophil percentage of granulocytes	A	G	0.9791	-0.09233	0.01692	4.822E-08	3E+07	European

CTSS	rs80026546	chr1:150718389	2016	173480	2	Neutrophil percentage of granulocytes	A	G	0.9791	0.0949	0.01692	2.05E-08	3E+07	European
CTSS	rs587743939	chr1:150718446	2017	337199	1	Other disorders of thyroid	T	G	0	0.002903	0.0005027	7.75E-09	UKBB	European
CTSS	rs75056606	chr1:150718602	2016	173480	2	Reticulocyte count	T	G	0.8648	-0.0297	0.00504	3.809E-09	3E+07	European
CTSS	rs75056606	chr1:150718602	2016	173480	2	Reticulocyte fraction of red cells	T	G	0.8648	-0.0288	0.005038	1.087E-08	3E+07	European
CTSS	rs72704613	chr1:150718780	2017	332021	1	Comparative height size at age 10	C	G	0.0278	0.03359	0.004637	4.365E-13	UKBB	European
CTSS	rs72704613	chr1:150718780	2017	336172	1	Sitting height	C	G	0.0278	0.0357	0.005251	1.058E-11	UKBB	European
CTSS	rs72704613	chr1:150718780	2017	255492	1	Forced vital capacity, best measure	C	G	0.0278	0.03309	0.006061	4.781E-08	UKBB	European
CTSS	rs72704613	chr1:150718780	2017	331291	1	Whole body fat-free mass	C	G	0.0278	0.02651	0.004294	6.725E-10	UKBB	European
CTSS	rs72704613	chr1:150718780	2017	331315	1	Whole body water mass	C	G	0.0278	0.02663	0.0043	5.958E-10	UKBB	European
CTSS	rs72704613	chr1:150718780	2017	331307	1	Basal metabolic rate	C	G	0.0278	0.02666	0.004502	3.187E-09	UKBB	European
CTSS	rs72704613	chr1:150718780	2017	331285	1	Leg fat-free mass right	C	G	0.0278	0.02515	0.004469	1.842E-08	UKBB	European
CTSS	rs72704613	chr1:150718780	2017	331285	1	Leg predicted mass right	C	G	0.0278	0.02512	0.00444	1.539E-08	UKBB	European
CTSS	rs72704613	chr1:150718780	2017	331258	1	Leg fat-free mass left	C	G	0.0278	0.02484	0.004471	2.782E-08	UKBB	European
CTSS	rs72704613	chr1:150718780	2017	331253	1	Leg predicted mass left	C	G	0.0278	0.02503	0.004441	1.741E-08	UKBB	European
CTSS	rs72704613	chr1:150718780	2017	331221	1	Arm fat-free mass right	C	G	0.0278	0.02484	0.004231	4.329E-09	UKBB	European
CTSS	rs72704613	chr1:150718780	2017	331216	1	Arm predicted mass right	C	G	0.0278	0.02562	0.004216	1.23E-09	UKBB	European
CTSS	rs72704613	chr1:150718780	2017	331159	1	Arm fat-free mass left	C	G	0.0278	0.02617	0.004317	1.345E-09	UKBB	European
CTSS	rs72704613	chr1:150718780	2017	331146	1	Arm predicted mass left	C	G	0.0278	0.02397	0.004301	2.493E-08	UKBB	European
CTSS	rs72704613	chr1:150718780	2017	331030	1	Trunk fat-free mass	C	G	0.0278	0.02759	0.004277	1.117E-10	UKBB	European
CTSS	rs72704613	chr1:150718780	2017	330995	1	Trunk predicted mass	C	G	0.0278	0.02784	0.004263	6.581E-11	UKBB	European
CTSS	rs72704613	chr1:150718780	2017	307638	1	Forced vital capacity	C	G	0.0278	0.03561	0.005523	1.139E-10	UKBB	European
CTSS	rs72704613	chr1:150718780	2017	336474	1	Height	C	G	0.0278	0.04451	0.004828	2.965E-20	UKBB	European
CTSS	rs191137782	chr1:150718979	2017	7637	1	Cause of death: creutzfeldt-jakob disease	T	G	0.999	-0.06535	0.009453	5.115E-12	UKBB	European
CTSS	rs191137782	chr1:150718979	2017	7637	1	Cause of death: tongue, unspecified	T	G	0.999	-0.06595	0.009916	3.128E-11	UKBB	European
CTSS	rs587633777	chr1:150720758	2017	337199	1	Complications peculiar to reattachment and amputation	T	C	0.002	0.001993	0.0003321	1.943E-09	UKBB	European
CTSS	rs147666195	chr1:150721111	2017	7637	1	Cause of death: other specified respiratory disorders	T	C	0.008	0.01763	0.00313	1.83E-08	UKBB	European
CTSS	rs1134067	chr1:150721175	2017	255492	1	Forced vital capacity, best measure	T	G	0.4085	0.01222	0.002218	3.629E-08	UKBB	European
CTSS	rs1134067	chr1:150721175	2017	307638	1	Forced vital capacity	T	G	0.4085	0.01184	0.002021	4.778E-09	UKBB	European
CTSS	rs1134067	chr1:150721175	2016	173480	2	Granulocyte percentage of myeloid white cells	T	G	0.4085	-0.03816	0.003655	1.614E-25	3E+07	European
CTSS	rs1134067	chr1:150721175	2016	173480	2	Monocyte count	T	G	0.4085	0.03466	0.003655	2.425E-21	3E+07	European
CTSS	rs1134067	chr1:150721175	2016	173480	2	Monocyte percentage of white cells	T	G	0.4085	0.03669	0.003644	7.713E-24	3E+07	European
CTSS	rs1134067	chr1:150721175	2016	173480	2	Neutrophil percentage of white cells	T	G	0.4085	-0.02815	0.003651	1.271E-14	3E+07	European
CTSS	rs11587444	chr1:150722844	2017	255492	1	Forced vital capacity, best measure	A	G	0.5915	-0.01221	0.002219	3.785E-08	UKBB	European
CTSS	rs11587444	chr1:150722844	2017	307638	1	Forced vital capacity	A	G	0.5915	-0.01181	0.002022	5.208E-09	UKBB	European
CTSS	rs11587444	chr1:150722844	2016	173480	2	Granulocyte percentage of myeloid white cells	A	G	0.5915	0.03801	0.003657	2.71E-25	3E+07	European
CTSS	rs11587444	chr1:150722844	2016	173480	2	Monocyte count	A	G	0.5915	-0.0346	0.003657	3.01E-21	3E+07	European
CTSS	rs11587444	chr1:150722844	2016	173480	2	Monocyte percentage of white cells	A	G	0.5915	-0.03649	0.003647	1.412E-23	3E+07	European
CTSS	rs11587444	chr1:150722844	2016	173480	2	Neutrophil percentage of white cells	A	G	0.5915	0.0282	0.003654	1.181E-14	3E+07	European
CTSS	rs3768018	chr1:150723747	2016	173480	2	Eosinophil percentage of granulocytes	T	C	0.6093	-0.02271	0.003704	8.715E-10	3E+07	European
CTSS	rs3768018	chr1:150723747	2016	173480	2	Granulocyte percentage of myeloid white cells	T	C	0.6093	0.03727	0.003697	6.751E-24	3E+07	European
CTSS	rs3768018	chr1:150723747	2016	173480	2	Monocyte count	T	C	0.6093	-0.03444	0.003697	1.212E-20	3E+07	European
CTSS	rs3768018	chr1:150723747	2016	173480	2	Monocyte percentage of white cells	T	C	0.6093	-0.03578	0.003686	2.837E-22	3E+07	European
CTSS	rs3768018	chr1:150723747	2016	173480	2	Neutrophil percentage of white cells	T	C	0.6093	0.02891	0.003693	4.968E-15	3E+07	European
CTSS	rs3768018	chr1:150723747	2016	173480	2	Neutrophil percentage of granulocytes	T	C	0.6093	0.02261	0.003704	1.027E-09	3E+07	European
CTSS	rs143084339	chr1:150723858	2017	7637	1	Cause of death: multisystem degeneration	A	G	0.004	0.05177	0.007424	3.362E-12	UKBB	European
CTSS	rs143084339	chr1:150723858	2017	7637	1	Cause of death: home	A	G	0.004	0.05162	0.007429	4E-12	UKBB	European
CTSS	rs1532770	chr1:150725335	2016	173480	2	Eosinophil percentage of granulocytes	T	G	0.3907	0.0226	0.003705	1.061E-09	3E+07	European
CTSS	rs1532770	chr1:150725335	2016	173480	2	Granulocyte percentage of myeloid white cells	T	G	0.3907	-0.03731	0.003699	6.284E-24	3E+07	European
CTSS	rs1532770	chr1:150725335	2016	173480	2	Monocyte count	T	G	0.3907	0.03429	0.003699	1.825E-20	3E+07	European
CTSS	rs1532770	chr1:150725335	2016	173480	2	Monocyte percentage of white cells	T	G	0.3907	0.03575	0.003688	3.197E-22	3E+07	European
CTSS	rs1532770	chr1:150725335	2016	173480	2	Neutrophil percentage of white cells	T	G	0.3907	-0.02905	0.003695	3.787E-15	3E+07	European
CTSS	rs1532770	chr1:150725335	2016	173480	2	Neutrophil percentage of granulocytes	T	G	0.3907	-0.02253	0.003706	1.211E-09	3E+07	European
CTSS	rs61386199	chr1:150725401	2017	255492	1	Forced vital capacity, best measure	A	C	0.1809	-0.01745	0.002665	5.833E-11	UKBB	European
CTSS	rs61386199	chr1:150725401	2017	307638	1	Forced vital capacity	A	C	0.1809	-0.01509	0.002427	5.063E-10	UKBB	European
CTSS	rs61386199	chr1:150725401	2016	173480	2	High light scatter reticulocyte count	A	C	0.1809	0.02547	0.004431	9.02E-09	3E+07	European
CTSS	rs61386199	chr1:150725401	2016	173480	2	Reticulocyte count	A	C	0.1809	0.02716	0.004437	9.334E-10	3E+07	European
CTSS	rs61386199	chr1:150725401	2016	173480	2	Reticulocyte fraction of red cells	A	C	0.1809	0.02565	0.004435	7.294E-09	3E+07	European
CTSS	rs77453473	chr1:150725738	2016	173480	2	Eosinophil count	A	G	0.9791	-0.1023	0.01684	1.262E-09	3E+07	European

CTSS	rs77453473	chr1:150725738	2016	173480	2	Sum eosinophil basophil counts	A	G	0.9791	-0.1067	0.01687	2.532E-10	3E+07	European
CTSS	rs77453473	chr1:150725738	2016	173480	2	Eosinophil percentage of white cells	A	G	0.9791	-0.09477	0.01685	1.85E-08	3E+07	European
CTSS	rs77453473	chr1:150725738	2016	173480	2	Neutrophil percentage of granulocytes	A	G	0.9791	0.09486	0.01694	2.129E-08	3E+07	European
CTSS	rs12757280	chr1:150726049	2016	173480	2	Monocyte count	T	C	0.1243	-0.02956	0.005321	2.774E-08	3E+07	European
CTSS	rs7526489	chr1:150726238	2017	307638	1	Forced vital capacity	T	C	0.3688	0.01191	0.002061	7.445E-09	UKBB	European
CTSS	rs7526489	chr1:150726238	2016	173480	2	Granulocyte percentage of myeloid white cells	T	C	0.3688	-0.03511	0.003725	4.256E-21	3E+07	European
CTSS	rs7526489	chr1:150726238	2016	173480	2	Monocyte count	T	C	0.3688	0.03087	0.003724	1.145E-16	3E+07	European
CTSS	rs7526489	chr1:150726238	2016	173480	2	Monocyte percentage of white cells	T	C	0.3688	0.03309	0.003714	5.094E-19	3E+07	European
CTSS	rs7526489	chr1:150726238	2016	173480	2	Neutrophil percentage of white cells	T	C	0.3688	-0.02826	0.003721	3.102E-14	3E+07	European
CTSS	rs368520811	chr1:150726906	2016	173480	2	Granulocyte percentage of myeloid white cells	C	CT	0.6879	0.03129	0.004211	1.077E-13	3E+07	European
CTSS	rs368520811	chr1:150726906	2016	173480	2	Monocyte count	C	CT	0.6879	-0.02985	0.00421	1.349E-12	3E+07	European
CTSS	rs368520811	chr1:150726906	2016	173480	2	Monocyte percentage of white cells	C	CT	0.6879	-0.03058	0.004199	3.269E-13	3E+07	European
CTSS	rs368520811	chr1:150726906	2016	173480	2	Neutrophil percentage of white cells	C	CT	0.6879	0.02342	0.004207	2.588E-08	3E+07	European
CTSS	rs111836402	chr1:150727037	2016	173480	2	Eosinophil count	A	G	0.0209	0.1025	0.01684	1.144E-09	3E+07	European
CTSS	rs111836402	chr1:150727037	2016	173480	2	Sum eosinophil basophil counts	A	G	0.0209	0.1071	0.01687	2.156E-10	3E+07	European
CTSS	rs111836402	chr1:150727037	2016	173480	2	Eosinophil percentage of white cells	A	G	0.0209	0.09502	0.01684	1.684E-08	3E+07	European
CTSS	rs111836402	chr1:150727037	2016	173480	2	Eosinophil percentage of granulocytes	A	G	0.0209	0.09237	0.01693	4.847E-08	3E+07	European
CTSS	rs111836402	chr1:150727037	2016	173480	2	Neutrophil percentage of granulocytes	A	G	0.0209	-0.09533	0.01693	1.803E-08	3E+07	European
CTSS	rs12068264	chr1:150727329	2017	307638	1	Forced vital capacity	T	C	0.3688	0.01191	0.00206	7.5E-09	UKBB	European
CTSS	rs12068264	chr1:150727329	2016	173480	2	Granulocyte percentage of myeloid white cells	T	C	0.3688	-0.03512	0.003724	4.053E-21	3E+07	European
CTSS	rs12068264	chr1:150727329	2016	173480	2	Lymphocyte percentage of white cells	T	C	0.3688	0.02042	0.003715	3.847E-08	3E+07	European
CTSS	rs12068264	chr1:150727329	2016	173480	2	Monocyte count	T	C	0.3688	0.0307	0.003724	1.667E-16	3E+07	European
CTSS	rs12068264	chr1:150727329	2016	173480	2	Monocyte percentage of white cells	T	C	0.3688	0.03301	0.003714	6.218E-19	3E+07	European
CTSS	rs12068264	chr1:150727329	2016	173480	2	Neutrophil percentage of white cells	T	C	0.3688	-0.02847	0.003721	2E-14	3E+07	European
CTSS	rs2230061	chr1:150727539	2017	307638	1	Forced vital capacity	A	G	0.3698	0.01192	0.00206	7.176E-09	UKBB	European
CTSS	rs2230061	chr1:150727539	2016	173480	2	Granulocyte percentage of myeloid white cells	A	G	0.3698	-0.03511	0.003722	3.962E-21	3E+07	European
CTSS	rs2230061	chr1:150727539	2016	173480	2	Monocyte count	A	G	0.3698	0.03086	0.003722	1.114E-16	3E+07	European
CTSS	rs2230061	chr1:150727539	2016	173480	2	Monocyte percentage of white cells	A	G	0.3698	0.03309	0.003712	4.902E-19	3E+07	European
CTSS	rs2230061	chr1:150727539	2016	173480	2	Neutrophil percentage of white cells	A	G	0.3698	-0.02831	0.003719	2.705E-14	3E+07	European
CTSS	rs2230061	chr1:150727539	2013	-	-	Fat body mass	A	G	0.3698	0.06	0.01093	0.00000004	2E+07	Mixed
CTSS	rs140691474	chr1:150728032	2017	307638	1	Forced vital capacity	A	G	0.6302	-0.0119	0.00206	7.663E-09	UKBB	European
CTSS	rs140691474	chr1:150728032	2016	173480	2	Granulocyte percentage of myeloid white cells	A	G	0.6302	0.03503	0.003723	5.065E-21	3E+07	European
CTSS	rs140691474	chr1:150728032	2016	173480	2	Monocyte count	A	G	0.6302	-0.03076	0.003723	1.421E-16	3E+07	European
CTSS	rs140691474	chr1:150728032	2016	173480	2	Monocyte percentage of white cells	A	G	0.6302	-0.033	0.003712	6.227E-19	3E+07	European
CTSS	rs140691474	chr1:150728032	2016	173480	2	Neutrophil percentage of white cells	A	G	0.6302	0.02824	0.00372	3.149E-14	3E+07	European
CTSS	rs10788794	chr1:150728676	2016	173480	2	Eosinophil percentage of granulocytes	T	C	0.6093	-0.02258	0.003701	1.042E-09	3E+07	European
CTSS	rs10788794	chr1:150728676	2016	173480	2	Granulocyte percentage of myeloid white cells	T	C	0.6093	0.03718	0.003694	7.828E-24	3E+07	European
CTSS	rs10788794	chr1:150728676	2016	173480	2	Monocyte count	T	C	0.6093	-0.03423	0.003694	1.93E-20	3E+07	European
CTSS	rs10788794	chr1:150728676	2016	173480	2	Monocyte percentage of white cells	T	C	0.6093	-0.03568	0.003683	3.421E-22	3E+07	European
CTSS	rs10788794	chr1:150728676	2016	173480	2	Neutrophil percentage of white cells	T	C	0.6093	0.0289	0.00369	4.8E-15	3E+07	European
CTSS	rs10788794	chr1:150728676	2016	173480	2	Neutrophil percentage of granulocytes	T	C	0.6093	0.02249	0.003701	1.216E-09	3E+07	European
CTSS	rs372780292	chr1:150729184	2016	173480	2	Granulocyte percentage of myeloid white cells	C	CT	0.7495	0.02971	0.004359	9.278E-12	3E+07	European
CTSS	rs372780292	chr1:150729184	2016	173480	2	Monocyte count	C	CT	0.7495	-0.02615	0.004358	1.957E-09	3E+07	European
CTSS	rs372780292	chr1:150729184	2016	173480	2	Monocyte percentage of white cells	C	CT	0.7495	-0.02685	0.004346	6.529E-10	3E+07	European
CTSS	rs372780292	chr1:150729184	2016	173480	2	Neutrophil percentage of white cells	C	CT	0.7495	0.02709	0.004355	4.976E-10	3E+07	European
CTSS	rs12568757	chr1:150729793	2017	255492	1	Forced vital capacity, best measure	A	G	0.5139	-0.0153	0.002177	2.109E-12	UKBB	European
CTSS	rs12568757	chr1:150729793	2017	307638	1	Forced vital capacity	A	G	0.5139	-0.01219	0.001983	7.95E-10	UKBB	European
CTSS	rs12568757	chr1:150729793	2016	173480	2	Eosinophil count	A	G	0.5139	-0.02011	0.003578	1.896E-08	3E+07	European
CTSS	rs12568757	chr1:150729793	2016	173480	2	Eosinophil percentage of granulocytes	A	G	0.5139	-0.02301	0.00359	1.452E-10	3E+07	European
CTSS	rs12568757	chr1:150729793	2016	173480	2	Granulocyte percentage of myeloid white cells	A	G	0.5139	0.04179	0.003584	2.018E-31	3E+07	European
CTSS	rs12568757	chr1:150729793	2016	173480	2	Lymphocyte count	A	G	0.5139	-0.02151	0.003613	2.63E-09	3E+07	European
CTSS	rs12568757	chr1:150729793	2016	173480	2	Monocyte count	A	G	0.5139	-0.03984	0.003583	1.027E-28	3E+07	European
CTSS	rs12568757	chr1:150729793	2016	173480	2	Monocyte percentage of white cells	A	G	0.5139	-0.04075	0.003574	4.049E-30	3E+07	European
CTSS	rs12568757	chr1:150729793	2016	173480	2	Neutrophil percentage of white cells	A	G	0.5139	0.03052	0.003581	1.55E-17	3E+07	European
CTSS	rs12568757	chr1:150729793	2016	173480	2	Neutrophil percentage of granulocytes	A	G	0.5139	0.02289	0.003591	1.828E-10	3E+07	European
CTSS	rs10888391	chr1:150729983	2016	173480	2	Eosinophil percentage of granulocytes	A	G	0.3907	0.02261	0.0037	9.908E-10	3E+07	European
CTSS	rs10888391	chr1:150729983	2016	173480	2	Granulocyte percentage of myeloid white cells	A	G	0.3907	-0.03714	0.003694	8.706E-24	3E+07	European
CTSS	rs10888391	chr1:150729983	2016	173480	2	Monocyte count	A	G	0.3907	0.03422	0.003694	1.945E-20	3E+07	European

CTSS	rs10888391	chr1:150729983	2016	173480	2	Monocyte percentage of white cells	A	G	0.3907	0.03562	0.003683	4.02E-22	3E+07	European
CTSS	rs10888391	chr1:150729983	2016	173480	2	Neutrophil percentage of white cells	A	G	0.3907	-0.02894	0.00369	4.48E-15	3E+07	European
CTSS	rs10888391	chr1:150729983	2016	173480	2	Neutrophil percentage of granulocytes	A	G	0.3907	-0.02253	0.003701	1.144E-09	3E+07	European
CTSS	rs11204722	chr1:150730184	2016	173480	2	Eosinophil percentage of granulocytes	A	C	0.3907	0.02258	0.003701	1.046E-09	3E+07	European
CTSS	rs11204722	chr1:150730184	2016	173480	2	Granulocyte percentage of myeloid white cells	A	C	0.3907	-0.0372	0.003694	7.501E-24	3E+07	European
CTSS	rs11204722	chr1:150730184	2016	173480	2	Monocyte count	A	C	0.3907	0.03425	0.003694	1.839E-20	3E+07	European
CTSS	rs11204722	chr1:150730184	2016	173480	2	Monocyte percentage of white cells	A	C	0.3907	0.0357	0.003684	3.276E-22	3E+07	European
CTSS	rs11204722	chr1:150730184	2016	173480	2	Neutrophil percentage of white cells	A	C	0.3907	-0.02892	0.003691	4.645E-15	3E+07	European
CTSS	rs11204722	chr1:150730184	2016	173480	2	Neutrophil percentage of granulocytes	A	C	0.3907	-0.0225	0.003701	1.208E-09	3E+07	European
CTSS	rs2275235	chr1:150730279	2017	307638	1	Forced vital capacity	A	G	0.6282	-0.01196	0.00206	6.332E-09	UKBB	European
CTSS	rs2275235	chr1:150730279	2016	173480	2	Granulocyte percentage of myeloid white cells	A	G	0.6282	0.03492	0.003723	6.608E-21	3E+07	European
CTSS	rs2275235	chr1:150730279	2016	173480	2	Monocyte count	A	G	0.6282	-0.03063	0.003723	1.906E-16	3E+07	European
CTSS	rs2275235	chr1:150730279	2016	173480	2	Monocyte percentage of white cells	A	G	0.6282	-0.03285	0.003713	8.973E-19	3E+07	European
CTSS	rs2275235	chr1:150730279	2016	173480	2	Neutrophil percentage of white cells	A	G	0.6282	0.02826	0.00372	3.041E-14	3E+07	European
CTSS	rs151223618	chr1:150731513	2016	173480	2	Eosinophil percentage of granulocytes	T	TA	0.3757	0.02138	0.003815	2.092E-08	3E+07	European
CTSS	rs151223618	chr1:150731513	2016	173480	2	Granulocyte percentage of myeloid white cells	T	TA	0.3757	-0.03966	0.003808	2.101E-25	3E+07	European
CTSS	rs151223618	chr1:150731513	2016	173480	2	Monocyte count	T	TA	0.3757	0.03616	0.003808	2.152E-21	3E+07	European
CTSS	rs151223618	chr1:150731513	2016	173480	2	Monocyte percentage of white cells	T	TA	0.3757	0.03805	0.003797	1.228E-23	3E+07	European
CTSS	rs151223618	chr1:150731513	2016	173480	2	Neutrophil percentage of white cells	T	TA	0.3757	-0.03012	0.003804	2.428E-15	3E+07	European
CTSS	rs151223618	chr1:150731513	2016	173480	2	Neutrophil percentage of granulocytes	T	TA	0.3757	-0.02162	0.003815	1.445E-08	3E+07	European
CTSS	rs59805030	chr1:150731514	2016	173480	2	Granulocyte percentage of myeloid white cells	A	T	0.8598	0.03597	0.006091	3.538E-09	3E+07	European
CTSS	rs59805030	chr1:150731514	2016	173480	2	Monocyte count	A	T	0.8598	-0.03388	0.006092	2.668E-08	3E+07	European
CTSS	rs59805030	chr1:150731514	2016	173480	2	Monocyte percentage of white cells	A	T	0.8598	-0.03846	0.006076	2.437E-10	3E+07	European
CTSS	rs11204723	chr1:150731618	2017	307638	1	Forced vital capacity	T	C	0.6272	-0.01201	0.00206	5.622E-09	UKBB	European
CTSS	rs11204723	chr1:150731618	2016	173480	2	Granulocyte percentage of myeloid white cells	T	C	0.6272	0.03487	0.003722	7.412E-21	3E+07	European
CTSS	rs11204723	chr1:150731618	2016	173480	2	Lymphocyte percentage of white cells	T	C	0.6272	-0.0203	0.003713	4.556E-08	3E+07	European
CTSS	rs11204723	chr1:150731618	2016	173480	2	Monocyte count	T	C	0.6272	-0.03057	0.003722	2.13E-16	3E+07	European
CTSS	rs11204723	chr1:150731618	2016	173480	2	Monocyte percentage of white cells	T	C	0.6272	-0.03275	0.003711	1.114E-18	3E+07	European
CTSS	rs11204723	chr1:150731618	2016	173480	2	Neutrophil percentage of white cells	T	C	0.6272	0.02831	0.003719	2.689E-14	3E+07	European
CTSS	rs74470446	chr1:150732298	2016	173480	2	Eosinophil count	C	G	0.0209	0.1023	0.01684	1.255E-09	3E+07	European
CTSS	rs74470446	chr1:150732298	2016	173480	2	Sum eosinophil basophil counts	C	G	0.0209	0.1068	0.01687	2.407E-10	3E+07	European
CTSS	rs74470446	chr1:150732298	2016	173480	2	Eosinophil percentage of white cells	C	G	0.0209	0.09485	0.01684	1.785E-08	3E+07	European
CTSS	rs74470446	chr1:150732298	2016	173480	2	Neutrophil percentage of granulocytes	C	G	0.0209	-0.09507	0.01693	1.969E-08	3E+07	European
CTSS	rs112821524	chr1:150733612	2016	173480	2	Eosinophil count	A	G	0.0209	0.1023	0.01683	1.204E-09	3E+07	European
CTSS	rs112821524	chr1:150733612	2016	173480	2	Sum eosinophil basophil counts	A	G	0.0209	0.1068	0.01686	2.382E-10	3E+07	European
CTSS	rs112821524	chr1:150733612	2016	173480	2	Eosinophil percentage of white cells	A	G	0.0209	0.09527	0.01683	1.504E-08	3E+07	European
CTSS	rs112821524	chr1:150733612	2016	173480	2	Eosinophil percentage of granulocytes	A	G	0.0209	0.093	0.01691	3.825E-08	3E+07	European
CTSS	rs112821524	chr1:150733612	2016	173480	2	Neutrophil percentage of granulocytes	A	G	0.0209	-0.09598	0.01692	1.402E-08	3E+07	European
CTSS	rs60427350	chr1:150733654	2016	173480	2	Granulocyte percentage of myeloid white cells	C	CAA	0.4473	-0.03232	0.003664	1.137E-18	3E+07	European
CTSS	rs60427350	chr1:150733654	2016	173480	2	Monocyte count	C	CAA	0.4473	0.03052	0.003664	7.957E-17	3E+07	European
CTSS	rs60427350	chr1:150733654	2016	173480	2	Monocyte percentage of white cells	C	CAA	0.4473	0.0308	0.003653	3.435E-17	3E+07	European
CTSS	rs60427350	chr1:150733654	2016	173480	2	Neutrophil percentage of white cells	C	CAA	0.4473	-0.02534	0.00366	4.43E-12	3E+07	European
CTSS	rs112658271	chr1:150734215	2016	173480	2	Eosinophil count	C	CAAGAAAAA	0.5119	-0.02023	0.00358	1.605E-08	3E+07	European
CTSS	rs112658271	chr1:150734215	2016	173480	2	Eosinophil percentage of granulocytes	C	CAAGAAAAA	0.5119	-0.02313	0.003592	1.198E-10	3E+07	European
CTSS	rs112658271	chr1:150734215	2016	173480	2	Granulocyte percentage of myeloid white cells	C	CAAGAAAAA	0.5119	0.04169	0.003585	2.927E-31	3E+07	European
CTSS	rs112658271	chr1:150734215	2016	173480	2	Lymphocyte count	C	CAAGAAAAA	0.5119	-0.0216	0.003615	2.308E-09	3E+07	European
CTSS	rs112658271	chr1:150734215	2016	173480	2	Monocyte count	C	CAAGAAAAA	0.5119	-0.03961	0.003585	2.199E-28	3E+07	European
CTSS	rs112658271	chr1:150734215	2016	173480	2	Monocyte percentage of white cells	C	CAAGAAAAA	0.5119	-0.04054	0.003575	8.436E-30	3E+07	European
CTSS	rs112658271	chr1:150734215	2016	173480	2	Neutrophil percentage of white cells	C	CAAGAAAAA	0.5119	0.03062	0.003582	1.265E-17	3E+07	European
CTSS	rs112658271	chr1:150734215	2016	173480	2	Neutrophil percentage of granulocytes	C	CAAGAAAAA	0.5119	0.023	0.003592	1.523E-10	3E+07	European
CTSS	rs111671311	chr1:150734331	2016	173480	2	Eosinophil count	A	G	0.0209	0.1033	0.01692	1.048E-09	3E+07	European
CTSS	rs111671311	chr1:150734331	2016	173480	2	Sum eosinophil basophil counts	A	G	0.0209	0.1079	0.01695	1.931E-10	3E+07	European
CTSS	rs111671311	chr1:150734331	2016	173480	2	Eosinophil percentage of white cells	A	G	0.0209	0.09592	0.01693	1.454E-08	3E+07	European
CTSS	rs111671311	chr1:150734331	2016	173480	2	Eosinophil percentage of granulocytes	A	G	0.0209	0.09316	0.01701	4.335E-08	3E+07	European
CTSS	rs111671311	chr1:150734331	2016	173480	2	Neutrophil percentage of granulocytes	A	G	0.0209	-0.0963	0.01702	1.523E-08	3E+07	European
CTSS	rs4537557	chr1:150734436	2017	307638	1	Forced vital capacity	A	G	0.6282	-0.01201	0.00206	5.566E-09	UKBB	European
CTSS	rs4537557	chr1:150734436	2016	173480	2	Granulocyte percentage of myeloid white cells	A	G	0.6282	0.03492	0.003722	6.545E-21	3E+07	European
CTSS	rs4537557	chr1:150734436	2016	173480	2	Lymphocyte percentage of white cells	A	G	0.6282	-0.02031	0.003713	4.503E-08	3E+07	European

CTSS	rs4537557	chr1:150734436	2016	173480	2	Monocyte count	A	G	0.6282	-0.03064	0.003722	1.846E-16	3E+07	European
CTSS	rs4537557	chr1:150734436	2016	173480	2	Monocyte percentage of white cells	A	G	0.6282	-0.0328	0.003712	9.842E-19	3E+07	European
CTSS	rs4537557	chr1:150734436	2016	173480	2	Neutrophil percentage of white cells	A	G	0.6282	0.02834	0.003719	2.526E-14	3E+07	European
CTSS	rs2864869	chr1:150734655	2017	307638	1	Forced vital capacity	A	G	0.3718	0.01201	0.00206	5.54E-09	UKBB	European
CTSS	rs2864869	chr1:150734655	2016	173480	2	Granulocyte percentage of myeloid white cells	A	G	0.3718	-0.03492	0.003722	6.465E-21	3E+07	European
CTSS	rs2864869	chr1:150734655	2016	173480	2	Lymphocyte percentage of white cells	A	G	0.3718	0.02031	0.003713	4.522E-08	3E+07	European
CTSS	rs2864869	chr1:150734655	2016	173480	2	Monocyte count	A	G	0.3718	0.03061	0.003722	1.947E-16	3E+07	European
CTSS	rs2864869	chr1:150734655	2016	173480	2	Monocyte percentage of white cells	A	G	0.3718	0.0328	0.003712	9.696E-19	3E+07	European
CTSS	rs2864869	chr1:150734655	2016	173480	2	Neutrophil percentage of white cells	A	G	0.3718	-0.02834	0.003719	2.542E-14	3E+07	European
CTSS	rs77089247	chr1:150735414	2017	7637	1	Cause of death: multisystem degeneration	T	C	0.002	0.0566	0.007777	3.725E-13	UKBB	European
CTSS	rs77089247	chr1:150735414	2017	7637	1	Cause of death: home	T	C	0.002	0.05693	0.007782	2.811E-13	UKBB	European
CTSS	rs113350565	chr1:150735505	2017	7637	1	Cause of death: acute pancreatitis, unspecified	C	G	0.0169	0.03131	0.004857	1.218E-10	UKBB	European
CTSS	rs202184518	chr1:150735580	2016	173480	2	Eosinophil percentage of granulocytes	T	TA	0.4016	0.02268	0.003711	9.933E-10	3E+07	European
CTSS	rs202184518	chr1:150735580	2016	173480	2	Granulocyte percentage of myeloid white cells	T	TA	0.4016	-0.03748	0.003704	4.627E-24	3E+07	European
CTSS	rs202184518	chr1:150735580	2016	173480	2	Monocyte count	T	TA	0.4016	0.03413	0.003704	3.107E-20	3E+07	European
CTSS	rs202184518	chr1:150735580	2016	173480	2	Monocyte percentage of white cells	T	TA	0.4016	0.03582	0.003694	3.116E-22	3E+07	European
CTSS	rs202184518	chr1:150735580	2016	173480	2	Neutrophil percentage of white cells	T	TA	0.4016	-0.02933	0.003701	2.265E-15	3E+07	European
CTSS	rs202184518	chr1:150735580	2016	173480	2	Neutrophil percentage of granulocytes	T	TA	0.4016	-0.02263	0.003711	1.081E-09	3E+07	European
CTSS	rs56404059	chr1:150737090	2017	336172	1	Sitting height	T	C	0.0139	0.05787	0.007579	2.243E-14	UKBB	European
CTSS	rs56404059	chr1:150737090	2017	255492	1	Forced expiratory volume in 1-second, best measure	T	C	0.0139	0.06391	0.009207	3.909E-12	UKBB	European
CTSS	rs56404059	chr1:150737090	2017	255492	1	Forced vital capacity, best measure	T	C	0.0139	0.07365	0.008718	2.986E-17	UKBB	European
CTSS	rs56404059	chr1:150737090	2017	331285	1	Leg predicted mass right	T	C	0.0139	0.03502	0.006409	4.664E-08	UKBB	European
CTSS	rs56404059	chr1:150737090	2017	307638	1	Forced vital capacity	T	C	0.0139	0.06472	0.007949	3.9E-16	UKBB	European
CTSS	rs56404059	chr1:150737090	2017	307638	1	Forced expiratory volume in 1-second	T	C	0.0139	0.05704	0.008384	1.026E-11	UKBB	European
CTSS	rs56404059	chr1:150737090	2017	307638	1	Peak expiratory flow	T	C	0.0139	0.04904	0.008714	1.824E-08	UKBB	European
CTSS	rs56404059	chr1:150737090	2017	336474	1	Height	T	C	0.0139	0.06564	0.006968	4.513E-21	UKBB	European
CTSS	rs41271951	chr1:150737220	2017	-	-	Blood protein levels	A	G	0.9235	-0.9647	0.07276	4E-40	3E+07	European
CTSS	rs3831278	chr1:150737565	2016	173480	2	Eosinophil percentage of granulocytes	T	TTCTC	0.6083	-0.02269	0.003702	8.907E-10	3E+07	European
CTSS	rs3831278	chr1:150737565	2016	173480	2	Granulocyte percentage of myeloid white cells	T	TTCTC	0.6083	0.03703	0.003695	1.238E-23	3E+07	European
CTSS	rs3831278	chr1:150737565	2016	173480	2	Monocyte count	T	TTCTC	0.6083	-0.03432	0.003695	1.563E-20	3E+07	European
CTSS	rs3831278	chr1:150737565	2016	173480	2	Monocyte percentage of white cells	T	TTCTC	0.6083	-0.03552	0.003685	5.458E-22	3E+07	European
CTSS	rs3831278	chr1:150737565	2016	173480	2	Neutrophil percentage of white cells	T	TTCTC	0.6083	0.0288	0.003692	6.144E-15	3E+07	European
CTSS	rs3831278	chr1:150737565	2016	173480	2	Neutrophil percentage of granulocytes	T	TTCTC	0.6083	0.02254	0.003702	1.146E-09	3E+07	European
CTSS	rs12061988	chr1:150737776	2017	307638	1	Forced vital capacity	T	C	0.6193	-0.01198	0.002074	7.605E-09	UKBB	European
CTSS	rs12061988	chr1:150737776	2016	173480	2	Eosinophil percentage of granulocytes	T	C	0.6193	-0.02056	0.003708	2.959E-08	3E+07	European
CTSS	rs12061988	chr1:150737776	2016	173480	2	Granulocyte percentage of myeloid white cells	T	C	0.6193	0.03575	0.003702	4.536E-22	3E+07	European
CTSS	rs12061988	chr1:150737776	2016	173480	2	Monocyte count	T	C	0.6193	-0.03246	0.003702	1.824E-18	3E+07	European
CTSS	rs12061988	chr1:150737776	2016	173480	2	Monocyte percentage of white cells	T	C	0.6193	-0.03402	0.003691	3.051E-20	3E+07	European
CTSS	rs12061988	chr1:150737776	2016	173480	2	Neutrophil percentage of white cells	T	C	0.6193	0.02825	0.003699	2.22E-14	3E+07	European
CTSS	rs12061988	chr1:150737776	2016	173480	2	Neutrophil percentage of granulocytes	T	C	0.6193	0.02033	0.003709	4.221E-08	3E+07	European
CTSS	rs55772167	chr1:150738095	2016	173480	2	Eosinophil count	A	G	0.9791	-0.1024	0.01683	1.183E-09	3E+07	European
CTSS	rs55772167	chr1:150738095	2016	173480	2	Sum eosinophil basophil counts	A	G	0.9791	-0.107	0.01686	2.233E-10	3E+07	European
CTSS	rs55772167	chr1:150738095	2016	173480	2	Eosinophil percentage of white cells	A	G	0.9791	-0.09462	0.01683	1.897E-08	3E+07	European
CTSS	rs55772167	chr1:150738095	2016	173480	2	Neutrophil percentage of granulocytes	A	G	0.9791	0.09462	0.01692	2.26E-08	3E+07	European
CTSS	rs1136774	chr1:150738197	2017	255492	1	Forced vital capacity, best measure	T	C	0.5119	-0.0154	0.002176	1.479E-12	UKBB	European
CTSS	rs1136774	chr1:150738197	2017	307638	1	Forced vital capacity	T	C	0.5119	-0.01225	0.001982	6.44E-10	UKBB	European
CTSS	rs1136774	chr1:150738197	2016	173480	2	Eosinophil count	T	C	0.5119	-0.02026	0.003577	1.478E-08	3E+07	European
CTSS	rs1136774	chr1:150738197	2016	173480	2	Eosinophil percentage of granulocytes	T	C	0.5119	-0.02308	0.003589	1.283E-10	3E+07	European
CTSS	rs1136774	chr1:150738197	2016	173480	2	Granulocyte percentage of myeloid white cells	T	C	0.5119	0.04167	0.003583	2.869E-31	3E+07	European
CTSS	rs1136774	chr1:150738197	2016	173480	2	Lymphocyte count	T	C	0.5119	-0.02163	0.003612	2.131E-09	3E+07	European
CTSS	rs1136774	chr1:150738197	2016	173480	2	Monocyte count	T	C	0.5119	-0.0398	0.003583	1.14E-28	3E+07	European
CTSS	rs1136774	chr1:150738197	2016	173480	2	Monocyte percentage of white cells	T	C	0.5119	-0.04058	0.003573	6.834E-30	3E+07	European
CTSS	rs1136774	chr1:150738197	2016	173480	2	Neutrophil percentage of white cells	T	C	0.5119	0.0305	0.00358	1.601E-17	3E+07	European
CTSS	rs1136774	chr1:150738197	2016	173480	2	Neutrophil percentage of granulocytes	T	C	0.5119	0.02289	0.00359	1.801E-10	3E+07	European
CTSS	rs3754212	chr1:150738200	2017	307638	1	Forced vital capacity	A	G	0.6282	-0.01201	0.00206	5.483E-09	UKBB	European
CTSS	rs3754212	chr1:150738200	2016	173480	2	Granulocyte percentage of myeloid white cells	A	G	0.6282	0.03481	0.003723	8.721E-21	3E+07	European
CTSS	rs3754212	chr1:150738200	2016	173480	2	Monocyte count	A	G	0.6282	-0.03075	0.003722	1.453E-16	3E+07	European
CTSS	rs3754212	chr1:150738200	2016	173480	2	Monocyte percentage of white cells	A	G	0.6282	-0.03269	0.003712	1.297E-18	3E+07	European

CTSS	rs3754212	chr1:150738200	2016	173480	2	Neutrophil percentage of white cells	A	G	0.6282	0.02814	0.003719	3.806E-14	3E+07	European
GSK3A	rs545790552	chr19:42734824	2017	337159	1	Self-reported liver or hepatocellular cancer	T	C	0.001	0.004475	0.0006865	7.068E-11	UKBB	European
GSK3A	rs545790552	chr19:42734824	2017	7637	1	Cause of death: ill-defined sites within the digestive system	T	C	0.001	0.063	0.01012	4.975E-10	UKBB	European
GSK3A	rs74259566	chr19:42735177	2017	336172	1	Sitting height	C	G	0.9085	0.01822	0.003149	7.235E-09	UKBB	European
GSK3A	rs74259566	chr19:42735177	2017	255492	1	Forced expiratory volume in 1-second, best measure	C	G	0.9085	0.02153	0.003835	1.979E-08	UKBB	European
GSK3A	rs74259566	chr19:42735177	2017	255492	1	Forced vital capacity, best measure	C	G	0.9085	0.02169	0.003631	2.338E-09	UKBB	European
GSK3A	rs74259566	chr19:42735177	2017	307638	1	Forced vital capacity	C	G	0.9085	0.02136	0.003307	1.065E-10	UKBB	European
GSK3A	rs74259566	chr19:42735177	2017	307638	1	Forced expiratory volume in 1-second	C	G	0.9085	0.02069	0.003488	3.014E-09	UKBB	European
GSK3A	rs74259566	chr19:42735177	2017	336474	1	Height	C	G	0.9085	0.01848	0.002895	1.738E-10	UKBB	European
GSK3A	rs8108096	chr19:42735798	2017	336172	1	Sitting height	T	C	0.9076	0.01792	0.003135	1.096E-08	UKBB	European
GSK3A	rs8108096	chr19:42735798	2017	255492	1	Forced expiratory volume in 1-second, best measure	T	C	0.9076	0.02094	0.003818	4.121E-08	UKBB	European
GSK3A	rs8108096	chr19:42735798	2017	255492	1	Forced vital capacity, best measure	T	C	0.9076	0.02111	0.003615	5.251E-09	UKBB	European
GSK3A	rs8108096	chr19:42735798	2017	307638	1	Forced vital capacity	T	C	0.9076	0.02075	0.003292	2.956E-10	UKBB	European
GSK3A	rs8108096	chr19:42735798	2017	307638	1	Forced expiratory volume in 1-second	T	C	0.9076	0.02	0.003472	8.408E-09	UKBB	European
GSK3A	rs8108096	chr19:42735798	2017	336474	1	Height	T	C	0.9076	0.01809	0.002882	3.451E-10	UKBB	European
GSK3A	rs78304176	chr19:42740612	2017	255492	1	Forced expiratory volume in 1-second, best measure	T	G	0.0656	-0.02737	0.004713	6.378E-09	UKBB	European
GSK3A	rs78304176	chr19:42740612	2017	255492	1	Forced vital capacity, best measure	T	G	0.0656	-0.02849	0.004462	1.724E-10	UKBB	European
GSK3A	rs78304176	chr19:42740612	2017	307638	1	Forced vital capacity	T	G	0.0656	-0.0254	0.004068	4.285E-10	UKBB	European
GSK3A	rs78304176	chr19:42740612	2017	336474	1	Height	T	G	0.0656	-0.02204	0.003563	6.18E-10	UKBB	European
GSK3A	rs78304176	chr19:42740612	2016	173480	2	Monocyte count	T	G	0.0656	0.04353	0.007297	2.438E-09	3E+07	European
GSK3A	rs55744031	chr19:42740863	2017	7637	1	Cause of death: other and unspecified intestinal obstruction	A	G	0.003	0.02633	0.003526	9.181E-14	UKBB	European
GSK3A	rs2302485	chr19:42741124	2014	239624	79	Height	A	G	0.9354	0.042	0.0064	2.6E-11	3E+07	European
GSK3A	rs2302485	chr19:42741124	2017	255492	1	Forced expiratory volume in 1-second, best measure	A	G	0.9354	0.02651	0.00469	1.588E-08	UKBB	European
GSK3A	rs2302485	chr19:42741124	2017	255492	1	Forced vital capacity, best measure	A	G	0.9354	0.02754	0.004441	5.622E-10	UKBB	European
GSK3A	rs2302485	chr19:42741124	2017	307638	1	Forced vital capacity	A	G	0.9354	0.02441	0.004048	1.638E-09	UKBB	European
GSK3A	rs2302485	chr19:42741124	2017	336474	1	Height	A	G	0.9354	0.02139	0.003545	1.609E-09	UKBB	European
GSK3A	rs2302485	chr19:42741124	2016	173480	2	Monocyte count	A	G	0.9354	-0.04267	0.007269	4.376E-09	3E+07	European
GSK3A	rs112654436	chr19:42741544	2017	255492	1	Forced expiratory volume in 1-second, best measure	T	C	0.9354	0.02669	0.004706	1.426E-08	UKBB	European
GSK3A	rs112654436	chr19:42741544	2017	255492	1	Forced vital capacity, best measure	T	C	0.9354	0.02765	0.004456	5.476E-10	UKBB	European
GSK3A	rs112654436	chr19:42741544	2017	307638	1	Forced vital capacity	T	C	0.9354	0.02439	0.004061	1.898E-09	UKBB	European
GSK3A	rs112654436	chr19:42741544	2017	336474	1	Height	T	C	0.9354	0.02149	0.003557	1.524E-09	UKBB	European
GSK3A	rs112654436	chr19:42741544	2016	173480	2	Monocyte count	T	C	0.9354	-0.04198	0.007293	8.591E-09	3E+07	European
GSK3A	rs148978874	chr19:42741845	2017	255492	1	Forced expiratory volume in 1-second, best measure	T	C	0.0646	-0.02633	0.004683	1.873E-08	UKBB	European
GSK3A	rs148978874	chr19:42741845	2017	255492	1	Forced vital capacity, best measure	T	C	0.0646	-0.02743	0.004434	6.226E-10	UKBB	European
GSK3A	rs148978874	chr19:42741845	2017	307638	1	Forced vital capacity	T	C	0.0646	-0.02434	0.004041	1.722E-09	UKBB	European
GSK3A	rs148978874	chr19:42741845	2017	336474	1	Height	T	C	0.0646	-0.02171	0.00354	8.614E-10	UKBB	European
GSK3A	rs148978874	chr19:42741845	2016	173480	2	Monocyte count	T	C	0.0646	0.04237	0.007289	6.137E-09	3E+07	European
GSK3A	rs535451872	chr19:42742031	2017	7637	1	Cause of death: malignant neoplasm of thyroid gland	A	T	0.003	0.04306	0.004763	1.967E-19	UKBB	European
GSK3A	rs117388241	chr19:42742125	2017	255492	1	Forced expiratory volume in 1-second, best measure	A	G	0.9354	0.02636	0.004684	1.831E-08	UKBB	European
GSK3A	rs117388241	chr19:42742125	2017	255492	1	Forced vital capacity, best measure	A	G	0.9354	0.02743	0.004436	6.287E-10	UKBB	European
GSK3A	rs117388241	chr19:42742125	2017	307638	1	Forced vital capacity	A	G	0.9354	0.02434	0.004042	1.733E-09	UKBB	European
GSK3A	rs117388241	chr19:42742125	2017	336474	1	Height	A	G	0.9354	0.02176	0.003541	7.977E-10	UKBB	European
GSK3A	rs117388241	chr19:42742125	2016	173480	2	Monocyte count	A	G	0.9354	-0.04259	0.007266	4.592E-09	3E+07	European
GSK3A	rs533278973	chr19:42742626	2017	337159	1	Self-reported benign or essential tremor	A	G	0.005	0.001894	0.0003179	2.547E-09	UKBB	European
GSK3A	rs708598	chr19:42745923	2017	7637	1	Cause of death: fatty liver	C	G	0.998	-0.01995	0.003545	1.902E-08	UKBB	European
GSK3A	rs372714233	chr19:42746289	2017	7637	1	Cause of death: acute pancreatitis, unspecified	T	C	0.998	-0.0686	0.01027	2.525E-11	UKBB	European
KRT5	rs79752942	chr12:52910837	2017	337159	1	Self-reported fracture rib	A	G	0.004	0.006302	0.001152	4.443E-08	UKBB	European
KRT5	rs79752942	chr12:52910837	2017	337159	1	Treatment with madopar 62.5 capsule	A	G	0.004	0.003517	0.0006398	3.888E-08	UKBB	European
KRT5	rs79752942	chr12:52910837	2017	7637	1	Cause of death: duodenum	A	G	0.004	0.06146	0.009338	4.977E-11	UKBB	European
KRT5	rs543384101	chr12:52912482	2017	337199	1	Dystonia	C	G	1	-0.002749	0.0004979	3.361E-08	UKBB	European
KRT5	rs641621	chr12:52912906	2017	337199	1	Other malignant neoplasms of skin	T	G	0.2823	-0.001859	0.0003324	2.235E-08	UKBB	European
KRT5	rs641615	chr12:52912909	2017	337199	1	Other malignant neoplasms of skin	T	G	0.2823	-0.001859	0.0003325	2.239E-08	UKBB	European
KRT5	rs149784665	chr12:52913182	2017	7637	1	Cause of death: pharynx, unspecified	A	G	0.005	0.04725	0.006618	1.028E-12	UKBB	European
KRT5	rs149784665	chr12:52913182	2017	7637	1	Cause of death: lobar pneumonia, unspecified	A	G	0.005	0.04719	0.007914	2.583E-09	UKBB	European
KRT5	rs638907	chr12:52913517	2017	337199	1	Other malignant neoplasms of skin	A	G	0.2773	-0.001794	0.0003289	4.926E-08	UKBB	European
KRT5	rs11170164	chr12:52913668	2017	337159	1	Self-reported basal cell carcinoma	T	C	0.0676	0.00257	0.0004374	4.201E-09	UKBB	European
KRT5	rs11170164	chr12:52913668	2017	337199	1	Other malignant neoplasms of skin	T	C	0.0676	0.003224	0.0005394	2.29E-09	UKBB	European
KRT5	rs11170164	chr12:52913668	2015	-	-	Basal cell carcinoma	T	C	0.0676	1.29	0.2244	0.000000009	3E+07	European

KRT5	rs11170164	chr12:52913668	2016	-	-	Basal cell carcinoma	T	C	0.0676	0.174	0.02167	1E-15	3E+07	European
KRT5	rs74093478	chr12:52913925	2017	337159	1	Treatment with olmetec 10mg tablet	T	C	0.003	0.004309	0.0006505	3.508E-11	UKBB	European
SHANK3	rs530240325	chr22:51113661	2017	7637	1	Cause of death: bronchopneumonia, unspecified	T	G	0	0.08922	0.01371	8.158E-11	UKBB	European
SHANK3	rs9616911	chr22:51114518	2016	173480	2	Mean platelet volume	T	C	0.5089	-0.02107	0.003609	5.301E-09	3E+07	European
SHANK3	rs9616914	chr22:51117137	2016	328917	64	Years of educational attainment	A	G	0.4314	0.015	0.003	2.565E-09	3E+07	European
SHANK3	rs9616914	chr22:51117137	2017	334070	1	Qualifications: none	A	G	0.4314	-0.006058	0.00093	7.347E-11	UKBB	European
SHANK3	rs9616914	chr22:51117137	2016	173480	2	Mean platelet volume	A	G	0.4314	-0.02202	0.003646	1.548E-09	3E+07	European
SHANK3	rs9616915	chr22:51117580	2016	173480	2	Mean platelet volume	T	C	0.4911	0.02056	0.003609	1.219E-08	3E+07	European
SHANK3	rs13054155	chr22:51118478	2016	173480	2	Mean platelet volume	T	C	0.5089	0.02254	0.003633	5.435E-10	3E+07	European
SHANK3	rs9616921	chr22:51119445	2016	173480	2	Mean platelet volume	C	G	0.4891	0.02062	0.003622	1.255E-08	3E+07	European
SHANK3	rs28712889	chr22:51119858	2016	173480	2	Mean platelet volume	C	G	0.495	0.0203	0.003626	2.178E-08	3E+07	European
SHANK3	rs7292776	chr22:51121171	2016	173480	2	Mean platelet volume	T	C	0.4294	-0.02174	0.003651	2.595E-09	3E+07	European
SHANK3	rs7286601	chr22:51121416	2016	328917	64	Years of educational attainment	T	G	0.5467	-0.015	0.003	3.644E-09	3E+07	European
SHANK3	rs7286601	chr22:51121416	2017	334070	1	Qualifications: none	T	G	0.5467	0.005773	0.0009289	5.139E-10	UKBB	European
SHANK3	rs7286601	chr22:51121416	2016	173480	2	Mean platelet volume	T	G	0.5467	0.02208	0.003653	1.492E-09	3E+07	European
SHANK3	rs7286601	chr22:51121416	2016	-	-	Educational attainment years of education	T	G	0.5467	NA	NA	0.000000002	3E+07	European
SHANK3	rs7284093	chr22:51121521	2016	173480	2	Mean corpuscular volume	T	C	0.1392	0.02849	0.004768	2.285E-09	3E+07	European
SHANK3	rs9616933	chr22:51121561	2016	173480	2	Mean corpuscular volume	T	G	0.0755	0.03663	0.005942	7.088E-10	3E+07	European
SHANK3	rs150022934	chr22:51121700	2017	7637	1	Cause of death: asthma, unspecified	T	C	0.0089	0.01928	0.002879	2.254E-11	UKBB	European
SHANK3	rs201282170	chr22:51121773	2017	337159	1	Treatment with nasobec aqueous 50micrograms nasal spray	T	C	0.003	0.002666	0.0004647	9.566E-09	UKBB	European
SHANK3	rs201282170	chr22:51121773	2017	7637	1	Cause of death: larynx, unspecified	T	C	0.003	0.03624	0.006612	4.365E-08	UKBB	European
SHANK3	rs78334860	chr22:51122590	2017	7637	1	Cause of death: appendix	A	G	0.007	0.03114	0.004573	1.054E-11	UKBB	European
SHANK3	rs571742670	chr22:51122883	2017	7637	1	Cause of death: gastro-intestinal haemorrhage, unspecified	A	G	0.003	0.03429	0.006181	2.977E-08	UKBB	European
SHANK3	rs187458677	chr22:51123092	2017	7637	1	Cause of death: pharynx, unspecified	T	C	0.002	0.05049	0.006985	5.339E-13	UKBB	European
SHANK3	rs75558741	chr22:51125798	2017	323978	1	Hearing difficulty or problems	A	G	0.0378	0.0212	0.003277	9.889E-11	UKBB	European
SHANK3	rs184396983	chr22:51126010	2017	7637	1	Cause of death: duodenum	T	C	0.0139	0.03148	0.003776	8.834E-17	UKBB	European
SHANK3	rs7354797	chr22:51128648	2016	328917	64	Years of educational attainment	T	C	0.4463	-0.014	0.003	1.975E-08	3E+07	European
SHANK3	rs7354799	chr22:51128649	2016	328917	64	Years of educational attainment	A	G	0.5537	0.014	0.003	2.621E-08	3E+07	European
SHANK3	rs13056386	chr22:51129324	2017	334070	1	Qualifications: none	T	C	0.4483	0.005669	0.0009284	1.021E-09	UKBB	European
SHANK3	rs145056584	chr22:51130635	2017	7637	1	Cause of death: acute and subacute infective endocarditis	T	C	0.0089	0.02182	0.003832	1.283E-08	UKBB	European
SHANK3	rs536296465	chr22:51131439	2017	7637	1	Cause of death: chronic myeloid leukaemia	T	C	0.001	0.04824	0.007222	2.566E-11	UKBB	European
SHANK3	rs536296465	chr22:51131439	2017	7637	1	Cause of death: lobar pneumonia, unspecified	T	C	0.001	0.04839	0.008075	2.152E-09	UKBB	European
SHANK3	rs536296465	chr22:51131439	2017	7637	1	Cause of death: emphysema, unspecified	T	C	0.001	0.04836	0.007664	2.934E-10	UKBB	European
SHANK3	rs776587561	chr22:51132704	2017	7637	1	Cause of death: alcoholic hepatitis	T	C	-	0.09289	0.009308	2.6E-23	UKBB	European
SHANK3	rs13055562	chr22:51133518	2016	328917	64	Years of educational attainment	A	G	0.5298	0.015	0.003	8.434E-09	3E+07	European
SHANK3	rs13055562	chr22:51133518	2017	334070	1	Qualifications: none	A	G	0.5298	-0.005358	0.0009263	7.313E-09	UKBB	European
SHANK3	rs77452243	chr22:51134387	2017	323978	1	Hearing difficulty or problems	A	G	0.0408	0.01908	0.00288	3.48E-11	UKBB	European
SHANK3	22:51136799_G_	chr22:51136799	2017	7637	1	Cause of death: other specified respiratory disorders	A	G	-	0.07113	0.00615	1.11E-30	UKBB	European
SHANK3	22:51136799_G_	chr22:51136799	2017	7637	1	Cause of death: calculus of gallbladder without cholecystitis	A	G	-	0.06905	0.009278	1.098E-13	UKBB	European
SHANK3	rs561931570	chr22:51137348	2017	337159	1	Treatment with minims artificial tears single-use eye drops	A	C	0.999	-0.002424	0.000434	2.346E-08	UKBB	European
SHANK3	rs561931570	chr22:51137348	2017	7637	1	Cause of death: chronic myeloid leukaemia	A	C	0.999	-0.0477	0.007181	3.288E-11	UKBB	European
SHANK3	rs561931570	chr22:51137348	2017	7637	1	Cause of death: lobar pneumonia, unspecified	A	C	0.999	-0.04784	0.008029	2.662E-09	UKBB	European
SHANK3	rs561931570	chr22:51137348	2017	7637	1	Cause of death: emphysema, unspecified	A	C	0.999	-0.04782	0.007621	3.688E-10	UKBB	European
SHANK3	rs528580229	chr22:51138334	2017	7637	1	Cause of death: chronic myeloid leukaemia	A	G	0.001	0.0486	0.007232	1.955E-11	UKBB	European
SHANK3	rs528580229	chr22:51138334	2017	7637	1	Cause of death: lobar pneumonia, unspecified	A	G	0.001	0.04855	0.008086	2.011E-09	UKBB	European
SHANK3	rs528580229	chr22:51138334	2017	7637	1	Cause of death: emphysema, unspecified	A	G	0.001	0.04853	0.007675	2.718E-10	UKBB	European
SHANK3	rs28526373	chr22:51145142	2017	7637	1	Cause of death: malignant neoplasm of thyroid gland	T	C	0.004	0.03019	0.004019	6.452E-14	UKBB	European
SHANK3	rs145518091	chr22:51146145	2017	7637	1	Cause of death: urinary organ, unspecified	A	T	0.993	-0.01538	0.00276	2.632E-08	UKBB	European
SHANK3	rs764343349	chr22:51146332	2017	7637	1	Cause of death: cardiomyopathy, unspecified	A	G	-	-0.07834	0.01387	1.685E-08	UKBB	European
SHANK3	rs764343349	chr22:51146332	2017	7637	1	Cause of death: inquest adjourned death	A	G	-	-0.05624	0.009809	1.02E-08	UKBB	European
SHANK3	rs147311580	chr22:51148083	2017	7637	1	Cause of death: appendix	A	G	0.999	-0.05185	0.005852	9.811E-19	UKBB	European
SHANK3	rs147311580	chr22:51148083	2017	7637	1	Cause of death: unspecified place	A	G	0.999	-0.05038	0.007421	1.217E-11	UKBB	European
SHANK3	rs550352371	chr22:51148647	2017	337159	1	Self-reported hypertrophic cardiomyopathy	T	G	0.997	-0.001889	0.0003123	1.478E-09	UKBB	European
SHANK3	rs565930645	chr22:51148661	2017	7637	1	Cause of death: aortic valve disorder, unspecified	A	T	0.997	-0.02423	0.004319	2.099E-08	UKBB	European
SHANK3	rs6009950	chr22:51150428	2017	337199	1	Dentofacial anomalies	T	C	0.002	0.008794	0.00115	2.057E-14	UKBB	European
SHANK3	rs5770820	chr22:51150473	2018	-	-	Intelligence multi trait analysis	A	G	0.2306	0.02047	0.003478	0.000000004	3E+07	Mixed
SHANK3	rs6009951	chr22:51151350	2017	334070	1	Qualifications: none	T	C	0.4135	0.005189	0.0009438	3.844E-08	UKBB	European
SHANK3	rs9616947	chr22:51151631	2017	334070	1	Qualifications: none	T	C	0.3877	0.005371	0.0009528	1.729E-08	UKBB	European

SHANK3	rs6010061	chr22:51151724	2017	334070	1	Qualifications: none	T	C	0.4095	0.005147	0.0009437	4.926E-08	UKBB	European
SHANK3	rs143299890	chr22:51153501	2017	7637	1	Cause of death: appendix	A	G	0.007	0.03573	0.004893	3.124E-13	UKBB	European
SHANK3	rs115459966	chr22:51153656	2017	7637	1	Cause of death: other specified respiratory disorders	T	C	0.004	0.05848	0.005534	6.378E-26	UKBB	European
SHANK3	rs185367504	chr22:51155581	2017	7637	1	Cause of death: pharynx, unspecified	C	G	0.005	0.03246	0.004276	3.557E-14	UKBB	European
SHANK3	rs564292820	chr22:51157916	2017	7637	1	Cause of death: cerebrum, except lobes and ventricles	T	C	0.008	0.02667	0.003303	7.848E-16	UKBB	European
SHANK3	rs144470529	chr22:51159258	2017	337159	1	Self-reported incisional hernia	T	C	0.004	0.003105	0.0004812	1.089E-10	UKBB	European
SHANK3	rs144470529	chr22:51159258	2017	337159	1	Treatment with care cetirizine hayfever relief 10mg tablet	T	C	0.004	0.003182	0.0005772	3.543E-08	UKBB	European
SHANK3	rs144470529	chr22:51159258	2017	337159	1	Treatment with avodart 500micrograms capsule	T	C	0.004	0.003102	0.0004471	3.984E-12	UKBB	European
SHANK3	rs144470529	chr22:51159258	2017	7637	1	Cause of death: other specified respiratory disorders	T	C	0.004	0.04636	0.004925	6.257E-21	UKBB	European
SHANK3	rs200077311	chr22:51159624	2017	7637	1	Cause of death: cerebrum, except lobes and ventricles	T	C	0.007	0.02592	0.003248	1.7E-15	UKBB	European
SHANK3	rs201483867	chr22:51160154	2017	7637	1	Cause of death: chronic myeloid leukaemia	A	G	0.001	0.04628	0.007054	5.729E-11	UKBB	European
SHANK3	rs201483867	chr22:51160154	2017	7637	1	Cause of death: lobar pneumonia, unspecified	A	G	0.001	0.04616	0.007887	5.038E-09	UKBB	European
SHANK3	rs201483867	chr22:51160154	2017	7637	1	Cause of death: emphysema, unspecified	A	G	0.001	0.0462	0.007486	7.104E-10	UKBB	European
SHANK3	rs563134354	chr22:51168035	2017	7637	1	Cause of death: peripheral vascular disease, unspecified	A	G	0.001	0.03221	0.004626	3.585E-12	UKBB	European
SHANK3	rs563134354	chr22:51168035	2017	7637	1	Cause of death: home	A	G	0.001	0.03182	0.00507	3.677E-10	UKBB	European
SHBG	rs569617452	chr17:7521126	2017	7637	1	Cause of death: alcoholic cirrhosis of liver	T	G	-	0.08332	0.0133	3.991E-10	UKBB	European
SHBG	rs569617452	chr17:7521126	2017	337199	1	Sarcoidosis	T	G	-	0.004912	0.0007113	5.015E-12	UKBB	European
SHBG	rs547194752	chr17:7521156	2017	337199	1	Other congenital malformations of skin	T	C	0.001	0.003382	0.0004867	3.707E-12	UKBB	European
SHBG	rs12150660	chr17:7521915	2017	4387	1	Testosterone	T	G	0.2416	0.09	0.01	5.952E-19	3E+07	European
SHBG	rs12150660	chr17:7521915	2017	337159	1	Self-reported hypertension	T	G	0.2416	0.006866	0.001228	2.285E-08	UKBB	European
SHBG	rs12150660	chr17:7521915	2017	331284	1	Impedance of whole body	T	G	0.2416	0.01274	0.002134	2.359E-09	UKBB	European
SHBG	rs12150660	chr17:7521915	2017	331279	1	Impedance of arm right	T	G	0.2416	0.01547	0.001968	3.9E-15	UKBB	European
SHBG	rs12150660	chr17:7521915	2017	331292	1	Impedance of arm left	T	G	0.2416	0.01499	0.001977	3.407E-14	UKBB	European
SHBG	rs12150660	chr17:7521915	2017	317754	1	Systolic blood pressure	T	G	0.2416	0.01664	0.002836	4.407E-09	UKBB	European
SHBG	rs12150660	chr17:7521915	2017	336683	1	Vascular or heart problems diagnosed by doctor: high blood pressure	T	G	0.2416	0.007092	0.001245	1.236E-08	UKBB	European
SHBG	rs12150660	chr17:7521915	2011	-	-	Testosterone levels	T	G	0.2416	31.8	2.35	1E-41	2E+07	European
SHBG	rs12150660	chr17:7521915	2012	-	-	Sex hormone binding globulin levels	T	G	0.2416	0.082	0.005229	2E-55	2E+07	European
SHBG	rs12150660	chr17:7521915	2012	-	-	Sex hormone binding globulin levels	T	G	0.2416	0.11	0.005803	4E-80	2E+07	European
SHBG	rs12150660	chr17:7521915	2012	-	-	Sex hormone binding globulin levels	T	G	0.2416	0.086	0.007565	6E-30	2E+07	European
SHBG	rs12150660	chr17:7521915	2012	-	-	Sex hormone binding globulin levels	T	G	0.2416	0.103	0.004701	2E-106	2E+07	European
SHBG	rs57828263	chr17:7522579	2017	4387	1	Testosterone	A	C	0.7247	-0.08	0.01	1.339E-16	3E+07	European
SHBG	rs57828263	chr17:7522579	2017	337159	1	Self-reported hypertension	A	C	0.7247	-0.006887	0.001189	6.869E-09	UKBB	European
SHBG	rs57828263	chr17:7522579	2017	331284	1	Impedance of whole body	A	C	0.7247	-0.01212	0.002065	4.379E-09	UKBB	European
SHBG	rs57828263	chr17:7522579	2017	331279	1	Impedance of arm right	A	C	0.7247	-0.01482	0.001905	7.105E-15	UKBB	European
SHBG	rs57828263	chr17:7522579	2017	331292	1	Impedance of arm left	A	C	0.7247	-0.01412	0.001913	1.527E-13	UKBB	European
SHBG	rs57828263	chr17:7522579	2017	317754	1	Systolic blood pressure	A	C	0.7247	-0.01513	0.002744	3.534E-08	UKBB	European
SHBG	rs57828263	chr17:7522579	2017	336683	1	Vascular or heart problems diagnosed by doctor: high blood pressure	A	C	0.7247	-0.007247	0.001205	1.81E-09	UKBB	European
SHBG	rs62059834	chr17:7523728	2017	4387	1	Testosterone	A	G	0.2416	0.09	0.01	5.815E-19	3E+07	European
SHBG	rs62059834	chr17:7523728	2017	337159	1	Self-reported hypertension	A	G	0.2416	0.006951	0.001229	1.55E-08	UKBB	European
SHBG	rs62059834	chr17:7523728	2017	331284	1	Impedance of whole body	A	G	0.2416	0.01267	0.002135	2.968E-09	UKBB	European
SHBG	rs62059834	chr17:7523728	2017	331279	1	Impedance of arm right	A	G	0.2416	0.01545	0.001969	4.234E-15	UKBB	European
SHBG	rs62059834	chr17:7523728	2017	331292	1	Impedance of arm left	A	G	0.2416	0.01496	0.001977	3.872E-14	UKBB	European
SHBG	rs62059834	chr17:7523728	2017	317754	1	Systolic blood pressure	A	G	0.2416	0.01675	0.002837	3.537E-09	UKBB	European
SHBG	rs62059834	chr17:7523728	2017	336683	1	Vascular or heart problems diagnosed by doctor: high blood pressure	A	G	0.2416	0.007195	0.001246	7.687E-09	UKBB	European
SHBG	rs569055729	chr17:7523951	2017	337159	1	Treatment with arcoxia 60mg tablet	T	G	1	-0.004254	0.0006749	2.921E-10	UKBB	European
SHBG	rs569055729	chr17:7523951	2017	7637	1	Cause of death: aortic valve disorder, unspecified	T	G	1	-0.07697	0.01065	5.365E-13	UKBB	European
SHBG	rs569055729	chr17:7523951	2017	7637	1	Cause of death: cerebral infarction, unspecified	T	G	1	-0.1477	0.01869	3.046E-15	UKBB	European
SHBG	rs67603370	chr17:7524504	2017	331693	1	Comparative body size at age 10	A	G	0.0765	0.01776	0.003178	2.298E-08	UKBB	European
SHBG	rs62059835	chr17:7525001	2017	4387	1	Testosterone	A	G	0.7584	-0.09	0.01	5.882E-19	3E+07	European
SHBG	rs62059835	chr17:7525001	2017	337159	1	Self-reported hypertension	A	G	0.7584	-0.006868	0.001228	2.26E-08	UKBB	European
SHBG	rs62059835	chr17:7525001	2017	331284	1	Impedance of whole body	A	G	0.7584	-0.0127	0.002134	2.661E-09	UKBB	European
SHBG	rs62059835	chr17:7525001	2017	331279	1	Impedance of arm right	A	G	0.7584	-0.01545	0.001968	4.22E-15	UKBB	European
SHBG	rs62059835	chr17:7525001	2017	331292	1	Impedance of arm left	A	G	0.7584	-0.01496	0.001977	3.74E-14	UKBB	European
SHBG	rs62059835	chr17:7525001	2017	317754	1	Systolic blood pressure	A	G	0.7584	-0.01663	0.002836	4.54E-09	UKBB	European
SHBG	rs62059835	chr17:7525001	2017	336683	1	Vascular or heart problems diagnosed by doctor: high blood pressure	A	G	0.7584	-0.007097	0.001245	1.209E-08	UKBB	European
SHBG	rs577897755	chr17:7526826	2017	337159	1	Treatment with colestyramine	C	G	0.002	0.003732	0.0005606	2.813E-11	UKBB	European
SHBG	rs577897755	chr17:7526826	2017	7637	1	Cause of death: ill-defined sites within the digestive system	C	G	0.002	0.06523	0.01023	1.89E-10	UKBB	European
SHBG	rs537645940	chr17:7527323	2017	7637	1	Cause of death: secondary malignant neoplasm of retroperitoneum and peritoneum	A	G	0.003	0.03898	0.006905	1.712E-08	UKBB	European

SHBG	rs180677275	chr17:7527635	2017	7637	1	Cause of death: tongue, unspecified	A	G	0.998	-0.05086	0.008693	5.077E-09	UKBB	European
SHBG	rs180677275	chr17:7527635	2017	7637	1	Cause of death: multiple sclerosis	A	G	0.998	-0.0759	0.01283	3.41E-09	UKBB	European
SHBG	rs180677275	chr17:7527635	2017	7637	1	Cause of death: thoracic aortic aneurysm, ruptured	A	G	0.998	-0.05172	0.006925	8.983E-14	UKBB	European
SHBG	rs142675740	chr17:7527668	2017	4387	1	Testosterone	T	C	0.2714	0.08	0.01	1.042E-16	3E+07	European
SHBG	rs142675740	chr17:7527668	2017	337159	1	Self-reported hypertension	T	C	0.2714	0.006715	0.001189	1.62E-08	UKBB	European
SHBG	rs142675740	chr17:7527668	2017	331284	1	Impedance of whole body	T	C	0.2714	0.01215	0.002066	4.11E-09	UKBB	European
SHBG	rs142675740	chr17:7527668	2017	331279	1	Impedance of arm right	T	C	0.2714	0.0149	0.001905	5.239E-15	UKBB	European
SHBG	rs142675740	chr17:7527668	2017	331292	1	Impedance of arm left	T	C	0.2714	0.01423	0.001913	1.034E-13	UKBB	European
SHBG	rs142675740	chr17:7527668	2017	317754	1	Systolic blood pressure	T	C	0.2714	0.01502	0.002745	4.471E-08	UKBB	European
SHBG	rs142675740	chr17:7527668	2017	336683	1	Vascular or heart problems diagnosed by doctor: high blood pressure	T	C	0.2714	0.007122	0.001205	3.45E-09	UKBB	European
SHBG	rs62059836	chr17:7527711	2017	4387	1	Testosterone	A	G	0.2714	0.08	0.01	1.001E-16	3E+07	European
SHBG	rs62059836	chr17:7527711	2017	337159	1	Self-reported hypertension	A	G	0.2714	0.006713	0.001189	1.638E-08	UKBB	European
SHBG	rs62059836	chr17:7527711	2017	331284	1	Impedance of whole body	A	G	0.2714	0.01214	0.002065	4.112E-09	UKBB	European
SHBG	rs62059836	chr17:7527711	2017	331279	1	Impedance of arm right	A	G	0.2714	0.0149	0.001905	5.225E-15	UKBB	European
SHBG	rs62059836	chr17:7527711	2017	331292	1	Impedance of arm left	A	G	0.2714	0.01422	0.001913	1.047E-13	UKBB	European
SHBG	rs62059836	chr17:7527711	2017	317754	1	Systolic blood pressure	A	G	0.2714	0.01504	0.002744	4.269E-08	UKBB	European
SHBG	rs62059836	chr17:7527711	2017	336683	1	Vascular or heart problems diagnosed by doctor: high blood pressure	A	G	0.2714	0.007118	0.001205	3.505E-09	UKBB	European
SHBG	rs62059837	chr17:7527946	2017	336172	1	Sitting height	A	C	0.9195	-0.02338	0.00293	1.486E-15	UKBB	European
SHBG	rs62059837	chr17:7527946	2017	331291	1	Whole body fat-free mass	A	C	0.9195	-0.0171	0.002397	9.706E-13	UKBB	European
SHBG	rs62059837	chr17:7527946	2017	331315	1	Whole body water mass	A	C	0.9195	-0.01704	0.0024	1.258E-12	UKBB	European
SHBG	rs62059837	chr17:7527946	2017	331307	1	Basal metabolic rate	A	C	0.9195	-0.01669	0.002513	3.114E-11	UKBB	European
SHBG	rs62059837	chr17:7527946	2017	331285	1	Leg fat-free mass right	A	C	0.9195	-0.01407	0.002495	1.714E-08	UKBB	European
SHBG	rs62059837	chr17:7527946	2017	331285	1	Leg predicted mass right	A	C	0.9195	-0.01423	0.002479	9.469E-09	UKBB	European
SHBG	rs62059837	chr17:7527946	2017	331221	1	Arm fat-free mass right	A	C	0.9195	-0.01432	0.002362	1.346E-09	UKBB	European
SHBG	rs62059837	chr17:7527946	2017	331216	1	Arm predicted mass right	A	C	0.9195	-0.01431	0.002353	1.189E-09	UKBB	European
SHBG	rs62059837	chr17:7527946	2017	331159	1	Arm fat-free mass left	A	C	0.9195	-0.01684	0.00241	2.772E-12	UKBB	European
SHBG	rs62059837	chr17:7527946	2017	331146	1	Arm predicted mass left	A	C	0.9195	-0.01685	0.002401	2.237E-12	UKBB	European
SHBG	rs62059837	chr17:7527946	2017	331030	1	Trunk fat-free mass	A	C	0.9195	-0.01939	0.002387	4.602E-16	UKBB	European
SHBG	rs62059837	chr17:7527946	2017	330995	1	Trunk predicted mass	A	C	0.9195	-0.01939	0.00238	3.676E-16	UKBB	European
SHBG	rs62059837	chr17:7527946	2017	336474	1	Height	A	C	0.9195	-0.02955	0.002693	5.297E-28	UKBB	European
SHBG	rs62059838	chr17:7528688	2017	336172	1	Sitting height	A	G	0.0974	0.02127	0.002829	5.598E-14	UKBB	European
SHBG	rs62059838	chr17:7528688	2017	331291	1	Whole body fat-free mass	A	G	0.0974	0.01471	0.002314	2.066E-10	UKBB	European
SHBG	rs62059838	chr17:7528688	2017	331315	1	Whole body water mass	A	G	0.0974	0.01468	0.002318	2.393E-10	UKBB	European
SHBG	rs62059838	chr17:7528688	2017	331307	1	Basal metabolic rate	A	G	0.0974	0.01425	0.002427	4.324E-09	UKBB	European
SHBG	rs62059838	chr17:7528688	2017	331216	1	Arm predicted mass right	A	G	0.0974	0.0125	0.002272	3.737E-08	UKBB	European
SHBG	rs62059838	chr17:7528688	2017	331159	1	Arm fat-free mass left	A	G	0.0974	0.0149	0.002327	1.536E-10	UKBB	European
SHBG	rs62059838	chr17:7528688	2017	331146	1	Arm predicted mass left	A	G	0.0974	0.01483	0.002318	1.582E-10	UKBB	European
SHBG	rs62059838	chr17:7528688	2017	331030	1	Trunk fat-free mass	A	G	0.0974	0.01727	0.002305	6.708E-14	UKBB	European
SHBG	rs62059838	chr17:7528688	2017	330995	1	Trunk predicted mass	A	G	0.0974	0.01725	0.002298	5.973E-14	UKBB	European
SHBG	rs62059838	chr17:7528688	2017	336474	1	Height	A	G	0.0974	0.02754	0.0026	3.269E-26	UKBB	European
SHBG	rs13894	chr17:7529902	2017	331291	1	Whole body fat-free mass	A	G	0.0815	-0.01609	0.002938	4.364E-08	UKBB	European
SHBG	rs13894	chr17:7529902	2017	331315	1	Whole body water mass	A	G	0.0815	-0.01611	0.002943	4.405E-08	UKBB	European
SHBG	rs13894	chr17:7529902	2017	331307	1	Basal metabolic rate	A	G	0.0815	-0.01731	0.003081	1.929E-08	UKBB	European
SHBG	rs13894	chr17:7529902	2017	331285	1	Leg fat-free mass right	A	G	0.0815	-0.01789	0.003058	4.914E-09	UKBB	European
SHBG	rs13894	chr17:7529902	2017	331285	1	Leg predicted mass right	A	G	0.0815	-0.0177	0.003038	5.745E-09	UKBB	European
SHBG	rs13894	chr17:7529902	2017	331258	1	Leg fat-free mass left	A	G	0.0815	-0.01802	0.003059	3.882E-09	UKBB	European
SHBG	rs13894	chr17:7529902	2017	331253	1	Leg predicted mass left	A	G	0.0815	-0.01775	0.003039	5.166E-09	UKBB	European
SHBG	rs13894	chr17:7529902	2017	336474	1	Height	A	G	0.0815	-0.02208	0.0033	2.223E-11	UKBB	European
SHBG	rs858519	chr17:7531965	2017	4387	1	Testosterone	T	C	0.4264	-0.06	0.01	8.181E-13	3E+07	European
SHBG	rs62059839	chr17:7533015	2017	4387	1	Testosterone	T	C	0.2565	0.09	0.01	7.618E-19	3E+07	European
SHBG	rs62059839	chr17:7533015	2017	337159	1	Self-reported hypertension	T	C	0.2565	0.006623	0.001215	4.991E-08	UKBB	European
SHBG	rs62059839	chr17:7533015	2017	331284	1	Impedance of whole body	T	C	0.2565	0.01192	0.002111	1.629E-08	UKBB	European
SHBG	rs62059839	chr17:7533015	2017	331279	1	Impedance of arm right	T	C	0.2565	0.01514	0.001946	7.278E-15	UKBB	European
SHBG	rs62059839	chr17:7533015	2017	331292	1	Impedance of arm left	T	C	0.2565	0.01466	0.001955	6.46E-14	UKBB	European
SHBG	rs62059839	chr17:7533015	2017	317754	1	Systolic blood pressure	T	C	0.2565	0.01535	0.002804	4.449E-08	UKBB	European
SHBG	rs62059839	chr17:7533015	2017	336683	1	Vascular or heart problems diagnosed by doctor: high blood pressure	T	C	0.2565	0.006803	0.001232	3.325E-08	UKBB	European
SHBG	rs858518	chr17:7533025	2017	4387	1	Testosterone	A	G	0.5905	0.06	0.01	1.029E-10	3E+07	European
SHBG	rs858518	chr17:7533025	2016	173480	2	Red cell distribution width	A	G	0.5905	0.0208	0.003593	7.083E-09	3E+07	European

SHBG	rs858518	chr17:7533025	2016	-	-	Red cell distribution width	A	G	0.5905	0.0208	0.003592	0.000000007	3E+07	European
SHBG	rs3760213	chr17:7533214	2017	336172	1	Sitting height	A	G	0.0994	0.02172	0.002842	2.116E-14	UKBB	European
SHBG	rs3760213	chr17:7533214	2017	331291	1	Whole body fat-free mass	A	G	0.0994	0.01451	0.002325	4.32E-10	UKBB	European
SHBG	rs3760213	chr17:7533214	2017	331315	1	Whole body water mass	A	G	0.0994	0.0145	0.002328	4.801E-10	UKBB	European
SHBG	rs3760213	chr17:7533214	2017	331307	1	Basal metabolic rate	A	G	0.0994	0.01395	0.002438	1.051E-08	UKBB	European
SHBG	rs3760213	chr17:7533214	2017	331159	1	Arm fat-free mass left	A	G	0.0994	0.0146	0.002337	4.244E-10	UKBB	European
SHBG	rs3760213	chr17:7533214	2017	331146	1	Arm predicted mass left	A	G	0.0994	0.01465	0.002329	3.159E-10	UKBB	European
SHBG	rs3760213	chr17:7533214	2017	331030	1	Trunk fat-free mass	A	G	0.0994	0.01705	0.002316	1.787E-13	UKBB	European
SHBG	rs3760213	chr17:7533214	2017	330995	1	Trunk predicted mass	A	G	0.0994	0.01705	0.002308	1.503E-13	UKBB	European
SHBG	rs3760213	chr17:7533214	2017	336474	1	Height	A	G	0.0994	0.02744	0.002612	8.427E-26	UKBB	European
SHBG	rs1799941	chr17:7533423	2017	4387	1	Testosterone	A	G	0.2555	0.09	0.01	7.708E-19	3E+07	European
SHBG	rs1799941	chr17:7533423	2017	337159	1	Self-reported hypertension	A	G	0.2555	0.006663	0.001216	4.31E-08	UKBB	European
SHBG	rs1799941	chr17:7533423	2017	331284	1	Impedance of whole body	A	G	0.2555	0.01174	0.002114	2.818E-08	UKBB	European
SHBG	rs1799941	chr17:7533423	2017	331279	1	Impedance of arm right	A	G	0.2555	0.01497	0.001949	1.567E-14	UKBB	European
SHBG	rs1799941	chr17:7533423	2017	331292	1	Impedance of arm left	A	G	0.2555	0.01449	0.001957	1.33E-13	UKBB	European
SHBG	rs1799941	chr17:7533423	2017	336683	1	Vascular or heart problems diagnosed by doctor: high blood pressure	A	G	0.2555	0.006865	0.001233	2.593E-08	UKBB	European
SHBG	rs9913778	chr17:7533901	2017	4387	1	Testosterone	T	C	0.0865	-0.09	0.02	4.19E-08	3E+07	European
SHBG	rs9913778	chr17:7533901	2017	336172	1	Sitting height	T	C	0.0865	-0.02021	0.003552	1.274E-08	UKBB	European
SHBG	rs9913778	chr17:7533901	2017	331291	1	Whole body fat-free mass	T	C	0.0865	-0.01698	0.002907	5.168E-09	UKBB	European
SHBG	rs9913778	chr17:7533901	2017	331315	1	Whole body water mass	T	C	0.0865	-0.01697	0.002911	5.575E-09	UKBB	European
SHBG	rs9913778	chr17:7533901	2017	331307	1	Basal metabolic rate	T	C	0.0865	-0.01808	0.003048	2.993E-09	UKBB	European
SHBG	rs9913778	chr17:7533901	2017	331285	1	Leg fat-free mass right	T	C	0.0865	-0.01854	0.003025	8.895E-10	UKBB	European
SHBG	rs9913778	chr17:7533901	2017	331285	1	Leg predicted mass right	T	C	0.0865	-0.01836	0.003006	0.000000001	UKBB	European
SHBG	rs9913778	chr17:7533901	2017	331258	1	Leg fat-free mass left	T	C	0.0865	-0.01877	0.003027	5.552E-10	UKBB	European
SHBG	rs9913778	chr17:7533901	2017	331253	1	Leg predicted mass left	T	C	0.0865	-0.01853	0.003006	7.142E-10	UKBB	European
SHBG	rs9913778	chr17:7533901	2017	331030	1	Trunk fat-free mass	T	C	0.0865	-0.01639	0.002895	1.505E-08	UKBB	European
SHBG	rs9913778	chr17:7533901	2017	330995	1	Trunk predicted mass	T	C	0.0865	-0.0162	0.002886	0.00000002	UKBB	European
SHBG	rs9913778	chr17:7533901	2017	336474	1	Height	T	C	0.0865	-0.02268	0.003265	3.728E-12	UKBB	European
SHBG	rs6258	chr17:7534678	2011	-	-	Testosterone levels	T	C	0.007	82.3	8.448	2E-22	2E+07	European
SHBG	rs6258	chr17:7534678	2012	-	-	Sex hormone binding globulin levels	T	C	0.007	0.272	0.01905	3E-46	2E+07	European
SHBG	rs6259	chr17:7536527	2014	253134	79	Height	A	G	0.1024	0.028	0.0046	1.7E-09	3E+07	European
SHBG	rs6259	chr17:7536527	2017	381625	106	Height	A	G	0.1024	0.028	0.004	1.1E-12	3E+07	European
SHBG	rs6259	chr17:7536527	2017	458927	147	Height	A	G	0.1024	0.026	0.0037	2E-12	3E+07	Mixed
SHBG	rs6259	chr17:7536527	2017	336172	1	Sitting height	A	G	0.1024	0.02091	0.002818	1.162E-13	UKBB	European
SHBG	rs6259	chr17:7536527	2017	331291	1	Whole body fat-free mass	A	G	0.1024	0.01422	0.002305	6.948E-10	UKBB	European
SHBG	rs6259	chr17:7536527	2017	331315	1	Whole body water mass	A	G	0.1024	0.01416	0.002308	8.529E-10	UKBB	European
SHBG	rs6259	chr17:7536527	2017	331307	1	Basal metabolic rate	A	G	0.1024	0.01369	0.002417	1.471E-08	UKBB	European
SHBG	rs6259	chr17:7536527	2017	331159	1	Arm fat-free mass left	A	G	0.1024	0.0144	0.002317	5.236E-10	UKBB	European
SHBG	rs6259	chr17:7536527	2017	331146	1	Arm predicted mass left	A	G	0.1024	0.01445	0.002309	3.857E-10	UKBB	European
SHBG	rs6259	chr17:7536527	2017	331030	1	Trunk fat-free mass	A	G	0.1024	0.01678	0.002296	2.659E-13	UKBB	European
SHBG	rs6259	chr17:7536527	2017	330995	1	Trunk predicted mass	A	G	0.1024	0.01677	0.002288	2.363E-13	UKBB	European
SHBG	rs6259	chr17:7536527	2017	336474	1	Height	A	G	0.1024	0.02709	0.00259	1.342E-25	UKBB	European
STX8	rs16958105	chr17:9153925	2017	7637	1	Cause of death: vascular dementia, unspecified	A	G	0.001	0.05301	0.009409	1.828E-08	UKBB	European
STX8	rs2072322	chr17:9154103	2017	194398	1	Heel bone mineral density	T	C	0.8161	0.02839	0.00409	3.854E-12	UKBB	European
STX8	rs78627041	chr17:9154317	2017	194398	1	Heel bone mineral density	T	C	0.8161	0.02838	0.00409	4.018E-12	UKBB	European
STX8	rs78068712	chr17:9154855	2017	194398	1	Heel bone mineral density	A	G	0.8062	0.02961	0.003994	1.244E-13	UKBB	European
STX8	rs78477453	chr17:9155053	2017	7637	1	Cause of death: intracranial haemorrhage, unspecified	T	C	0.0109	0.03452	0.005012	6.164E-12	UKBB	European
STX8	rs11870775	chr17:9155067	2017	194398	1	Heel bone mineral density	A	G	0.173	-0.02892	0.004178	4.465E-12	UKBB	European
STX8	rs75860636	chr17:9155271	2017	194398	1	Heel bone mineral density	A	G	0.828	0.02921	0.004185	2.956E-12	UKBB	European
STX8	rs11869111	chr17:9155330	2017	194398	1	Heel bone mineral density	A	T	0.173	-0.02898	0.004184	4.368E-12	UKBB	European
STX8	rs75111163	chr17:9155479	2017	194398	1	Heel bone mineral density	A	T	0.173	-0.0291	0.004184	3.53E-12	UKBB	European
STX8	rs56088438	chr17:9155702	2017	194398	1	Heel bone mineral density	A	C	0.4394	-0.01809	0.003217	1.887E-08	UKBB	European
STX8	rs374332946	chr17:9155854	2017	7637	1	Cause of death: intracranial haemorrhage, unspecified	A	T	0.9891	-0.03496	0.005042	4.44E-12	UKBB	European
STX8	rs145530443	chr17:9156622	2017	7637	1	Cause of death: intracranial haemorrhage, unspecified	A	G	0.0109	0.03518	0.005058	3.802E-12	UKBB	European
STX8	rs140314627	chr17:9157323	2017	7637	1	Cause of death: intracranial haemorrhage, unspecified	T	C	0.0099	0.03164	0.004802	4.702E-11	UKBB	European
STX8	rs373524262	chr17:9158672	2017	7637	1	Cause of death: intracranial haemorrhage, unspecified	T	C	0.0089	0.02272	0.004074	2.545E-08	UKBB	European
STX8	rs373696786	chr17:9161120	2017	7637	1	Cause of death: intracranial haemorrhage, unspecified	T	C	0.0089	0.02285	0.004083	2.264E-08	UKBB	European
STX8	rs78402762	chr17:9162188	2017	337199	1	Rheumatic mitral valve diseases	A	G	0.003	0.001811	0.0002973	1.129E-09	UKBB	European

STX8	rs182810125	chr17:9163746	2017	7637	1	Cause of death: intracranial haemorrhage, unspecified	C	G	0.0089	0.02265	0.004065	2.61E-08	UKBB	European
STX8	rs182640725	chr17:9164920	2017	7637	1	Cause of death: alcoholic hepatitis	A	G	0	0.03379	0.00562	1.918E-09	UKBB	European
STX8	rs537771387	chr17:9166752	2017	7637	1	Cause of death: home	T	C	0.001	0.04429	0.008084	4.429E-08	UKBB	European
STX8	rs533273842	chr17:9170004	2017	7637	1	Cause of death: follicular non-hodgkins lymphoma, unspecified	T	C	-	-0.03789	0.006924	4.587E-08	UKBB	European
STX8	rs190893650	chr17:9171377	2017	337199	1	Dislocation, sprain and strain of joints and ligaments at neck level	T	C	0.998	-0.002374	0.0004251	2.333E-08	UKBB	European
STX8	rs182003708	chr17:9173625	2017	7637	1	Cause of death: home	A	G	0.005	0.02633	0.00453	6.385E-09	UKBB	European
STX8	rs559023247	chr17:9178224	2017	337159	1	Treatment with climesse tablet	T	C	1	-0.004364	0.000766	1.215E-08	UKBB	European
STX8	rs559023247	chr17:9178224	2017	7637	1	Cause of death: home	T	C	1	-0.06019	0.009379	1.469E-10	UKBB	European
STX8	rs762378383	chr17:9179895	2017	7637	1	Cause of death: pneumonitis due to food and vomit	T	C	-	0.03964	0.006544	1.45E-09	UKBB	European
STX8	rs141862184	chr17:9180157	2017	7637	1	Cause of death: cerebrum, except lobes and ventricles	A	G	0.002	0.03122	0.005051	6.693E-10	UKBB	European
STX8	rs556605525	chr17:9180524	2017	7637	1	Cause of death: thoracic aortic aneurysm, ruptured	A	G	0.999	-0.04168	0.007468	2.476E-08	UKBB	European
STX8	rs138904077	chr17:9192473	2017	337159	1	Treatment with cyclizine	T	C	0.003	0.003102	0.0005251	3.467E-09	UKBB	European
STX8	rs138904077	chr17:9192473	2017	7637	1	Cause of death: cervix uteri, unspecified	T	C	0.003	0.03947	0.006945	1.373E-08	UKBB	European
STX8	rs138904077	chr17:9192473	2017	7637	1	Cause of death: pneumonia, unspecified	T	C	0.003	0.1028	0.01878	4.483E-08	UKBB	European
STX8	rs183985438	chr17:9194351	2017	7637	1	Cause of death: malignant neoplasm of thyroid gland	T	C	0.003	0.01725	0.003047	1.547E-08	UKBB	European
STX8	rs537633930	chr17:9196094	2017	7637	1	Cause of death: stomach, unspecified	A	C	0.001	0.2055	0.03242	2.451E-10	UKBB	European
STX8	rs537633930	chr17:9196094	2017	337199	1	Abnormal findings in specimens from male genital organs	A	C	0.001	0.003689	0.0006519	1.524E-08	UKBB	European
STX8	rs148965136	chr17:9199017	2017	337159	1	Treatment with bromelains	A	G	0.006	0.001584	0.0002727	6.285E-09	UKBB	European
STX8	rs568965578	chr17:9199579	2017	7637	1	Cause of death: inquest adjourned death	T	C	0.003	0.04422	0.007327	1.661E-09	UKBB	European
STX8	rs545497952	chr17:9201316	2017	337159	1	Treatment with cyclizine	A	G	0.997	-0.002692	0.0004261	2.681E-10	UKBB	European
STX8	rs145125101	chr17:9201931	2017	7637	1	Cause of death: inquest adjourned death	T	C	0.003	0.04173	0.007127	4.962E-09	UKBB	European
STX8	rs7222409	chr17:9204395	2017	337159	1	Self-reported chronic myeloid	T	C	0.005	0.003469	0.0005699	1.153E-09	UKBB	European
STX8	rs7222409	chr17:9204395	2017	7637	1	Cause of death: rheumatoid arthritis, unspecified	T	C	0.005	0.06768	0.009562	1.589E-12	UKBB	European
STX8	rs57851911	chr17:9206196	2017	7637	1	Cause of death: thoracic aortic aneurysm, ruptured	A	G	0.994	-0.0461	0.006734	8.234E-12	UKBB	European
STX8	rs57851911	chr17:9206196	2017	7637	1	Cause of death: chronic obstructive pulmonary disease with acute lower respiratory infection	A	G	0.994	-0.1249	0.02029	7.916E-10	UKBB	European
STX8	rs57851911	chr17:9206196	2017	7637	1	Cause of death: rheumatoid arthritis, unspecified	A	G	0.994	-0.04749	0.008058	3.947E-09	UKBB	European
STX8	rs561927585	chr17:9207174	2017	337159	1	Treatment with avodart 500micrograms capsule	A	G	0.999	-0.002794	0.0005033	2.837E-08	UKBB	European
STX8	rs561927585	chr17:9207174	2017	7637	1	Cause of death: home	A	G	0.999	-0.08835	0.01135	8.1E-15	UKBB	European
STX8	rs555468838	chr17:9207523	2017	7637	1	Cause of death: rheumatoid arthritis, unspecified	T	C	-	0.0452	0.007782	6.578E-09	UKBB	European
STX8	rs555468838	chr17:9207523	2017	7637	1	Cause of death: unspecified place	T	C	-	0.04535	0.00696	7.712E-11	UKBB	European
STX8	rs1263876	chr17:9207841	2017	7637	1	Cause of death: rheumatoid arthritis, unspecified	A	G	0.9851	-0.08831	0.0109	6.251E-16	UKBB	European
STX8	rs7210370	chr17:9209702	2017	7637	1	Cause of death: rheumatoid arthritis, unspecified	A	G	0.004	0.08901	0.01094	4.619E-16	UKBB	European
STX8	rs558515822	chr17:9209839	2017	337159	1	Self-reported hereditary or genetic haematological disorder	A	G	-	0.005318	0.000891	2.4E-09	UKBB	European
STX8	rs558515822	chr17:9209839	2017	7637	1	Cause of death: acute lymphoblastic leukaemia	A	G	-	0.03753	0.005483	8.336E-12	UKBB	European
STX8	rs558515822	chr17:9209839	2017	7637	1	Cause of death: peripheral vascular disease, unspecified	A	G	-	0.03787	0.005004	4.25E-14	UKBB	European
STX8	rs188259205	chr17:9210434	2017	7637	1	Cause of death: parkinsons disease	A	G	0.001	0.1165	0.01288	1.915E-19	UKBB	European
STX8	rs113446493	chr17:9210528	2017	7637	1	Cause of death: rheumatoid arthritis, unspecified	A	C	0.007	0.06809	0.009602	1.453E-12	UKBB	European
STX8	rs8067593	chr17:9211051	2017	7637	1	Cause of death: rheumatoid arthritis, unspecified	T	G	0.005	0.08686	0.01081	1.07E-15	UKBB	European
STX8	rs8068752	chr17:9211053	2017	7637	1	Cause of death: rheumatoid arthritis, unspecified	T	C	0.005	0.08684	0.01081	1.078E-15	UKBB	European
STX8	rs570381891	chr17:9211187	2017	7637	1	Cause of death: parkinsons disease	A	C	0.001	0.1069	0.01232	4.856E-18	UKBB	European
STX8	rs8069648	chr17:9211212	2017	7637	1	Cause of death: rheumatoid arthritis, unspecified	A	G	0.996	-0.08851	0.01091	5.655E-16	UKBB	European
STX8	rs111646715	chr17:9211780	2017	7637	1	Cause of death: rheumatoid arthritis, unspecified	A	G	0.005	0.08685	0.01081	1.065E-15	UKBB	European
STX8	rs73266002	chr17:9213411	2017	7637	1	Cause of death: rheumatoid arthritis, unspecified	T	G	0.995	-0.08692	0.01081	1.038E-15	UKBB	European
STX8	rs56928304	chr17:9213991	2017	7637	1	Cause of death: thoracic aortic aneurysm, ruptured	A	C	0.994	-0.04683	0.006781	5.388E-12	UKBB	European
STX8	rs56928304	chr17:9213991	2017	7637	1	Cause of death: chronic obstructive pulmonary disease with acute lower respiratory infection	A	C	0.994	-0.1262	0.02043	6.844E-10	UKBB	European
STX8	rs56928304	chr17:9213991	2017	7637	1	Cause of death: rheumatoid arthritis, unspecified	A	C	0.994	-0.04804	0.008114	3.351E-09	UKBB	European
STX8	rs532135821	chr17:9216973	2017	7637	1	Cause of death: other specified respiratory disorders	T	C	0	0.06414	0.005928	4.365E-27	UKBB	European
STX8	rs532135821	chr17:9216973	2017	337199	1	Plasmodium falciparum malaria	T	C	0	0.00278	0.0003745	1.152E-13	UKBB	European
STX8	rs554697925	chr17:9217746	2017	7637	1	Cause of death: home	A	T	1	-0.09455	0.008895	3.313E-26	UKBB	European
STX8	rs78075109	chr17:9218613	2017	7637	1	Cause of death: hodgkins disease, unspecified	A	C	0.9761	-0.01294	0.002222	5.982E-09	UKBB	European
STX8	rs532538974	chr17:9219920	2017	7637	1	Cause of death: malignant neoplasm of thyroid gland	T	C	-	0.0387	0.004502	9.881E-18	UKBB	European
STX8	rs532538974	chr17:9219920	2017	7637	1	Cause of death: gastro-intestinal haemorrhage, unspecified	T	C	-	0.03775	0.00597	2.718E-10	UKBB	European
STX8	rs577127746	chr17:9221493	2017	7637	1	Cause of death: parkinsons disease	T	G	0.001	0.1144	0.01277	4.215E-19	UKBB	European
STX8	rs564451266	chr17:9226174	2017	7637	1	Cause of death: perforation of intestine	T	C	-	-0.04148	0.007518	3.558E-08	UKBB	European
STX8	rs564451266	chr17:9226174	2017	7637	1	Cause of death: gastro-intestinal haemorrhage, unspecified	T	C	-	-0.04194	0.006287	2.73E-11	UKBB	European
STX8	rs191850692	chr17:9228175	2017	7637	1	Cause of death: acute lymphoblastic leukaemia	C	G	0.997	-0.03063	0.004965	7.263E-10	UKBB	European
STX8	rs191850692	chr17:9228175	2017	7637	1	Cause of death: peripheral vascular disease, unspecified	C	G	0.997	-0.03086	0.004532	1.053E-11	UKBB	European
STX8	rs146112587	chr17:9236767	2017	7637	1	Cause of death: hodgkins disease, unspecified	T	C	0.996	-0.02123	0.003236	5.757E-11	UKBB	European

STX8	rs543864140	chr17:9236862	2017	7637	1	Cause of death: rheumatoid arthritis, unspecified	A	G	0.001	0.0505	0.008246	9.538E-10	UKBB	European
STX8	rs180965943	chr17:9236914	2017	337159	1	Treatment with cyclizine	C	G	0.0109	0.002639	0.0004056	7.712E-11	UKBB	European
STX8	rs180965943	chr17:9236914	2017	7637	1	Cause of death: home	C	G	0.0109	0.02187	0.003841	1.278E-08	UKBB	European
STX8	rs192641683	chr17:9238435	2017	7637	1	Cause of death: endocarditis, valve unspecified	T	C	0.001	0.05074	0.007361	5.895E-12	UKBB	European
STX8	rs148578316	chr17:9239270	2017	337159	1	Treatment with bromelains	A	T	0.006	0.001749	0.0002753	2.091E-10	UKBB	European
STX8	rs148578316	chr17:9239270	2017	7637	1	Cause of death: home	A	T	0.006	0.01913	0.003245	3.9E-09	UKBB	European
STX8	rs186196815	chr17:9240995	2017	337159	1	Treatment with aminophylline	A	T	0.996	-0.002233	0.0004075	4.252E-08	UKBB	European
STX8	rs186196815	chr17:9240995	2017	7637	1	Cause of death: malignant neoplasm of thyroid gland	A	T	0.996	-0.04458	0.004838	3.937E-20	UKBB	European
STX8	rs186196815	chr17:9240995	2017	7637	1	Cause of death: emphysema, unspecified	A	T	0.996	-0.04246	0.007283	5.781E-09	UKBB	European
STX8	rs182947122	chr17:9242252	2017	337199	1	Fracture of foot, except ankle	A	G	0.0159	0.002145	0.0003911	4.176E-08	UKBB	European
STX8	rs192423586	chr17:9243065	2017	7637	1	Cause of death: interstitial pulmonary disease, unspecified	T	C	0.998	-0.04551	0.007924	9.626E-09	UKBB	European
STX8	rs192423586	chr17:9243065	2017	7637	1	Cause of death: home	T	C	0.998	-0.02506	0.004065	7.466E-10	UKBB	European
STX8	rs78997488	chr17:9243355	2017	337159	1	Treatment with lecithin product	A	G	0.998	-0.004281	0.0007552	1.433E-08	UKBB	European
STX8	rs78997488	chr17:9243355	2017	337159	1	Treatment with estriol product	A	G	0.998	-0.003524	0.0006309	2.324E-08	UKBB	European
STX8	rs144939675	chr17:9243849	2017	7637	1	Cause of death: aortic stenosis	T	C	0.008	0.03428	0.005836	4.449E-09	UKBB	European
STX8	rs118018936	chr17:9244646	2017	7637	1	Cause of death: pharynx, unspecified	C	G	0.0119	0.0185	0.003132	3.614E-09	UKBB	European
STX8	.	chr17:9248070	2017	7637	1	Cause of death: oropharynx, unspecified	T	G	-	-0.06413	0.009985	1.419E-10	UKBB	European
STX8	.	chr17:9248070	2017	7637	1	Cause of death: subarachnoid haemorrhage from intracranial artery, unspecified	T	G	-	-0.07539	0.008332	1.81E-19	UKBB	European
STX8	rs187101106	chr17:9248388	2017	7637	1	Cause of death: malignant neoplasm of thyroid gland	T	C	0.999	-0.03824	0.004478	1.597E-17	UKBB	European
STX8	rs187101106	chr17:9248388	2017	7637	1	Cause of death: gastro-intestinal haemorrhage, unspecified	T	C	0.999	-0.03738	0.005938	3.241E-10	UKBB	European
STX8	rs187101106	chr17:9248388	2017	337199	1	Viral warts	T	C	0.999	-0.006097	0.001049	6.21E-09	UKBB	European
STX8	rs555337864	chr17:9249353	2017	7637	1	Cause of death: other and unspecified t-cell lymphomas	T	C	0	0.03875	0.006953	2.594E-08	UKBB	European
STX8	rs555337864	chr17:9249353	2017	7637	1	Cause of death: peripheral vascular disease, unspecified	T	C	0	0.04047	0.005174	5.895E-15	UKBB	European
STX8	rs555337864	chr17:9249353	2017	337199	1	Acute and transient psychotic disorders	T	C	0	0.002494	0.0004524	3.555E-08	UKBB	European
STX8	rs147672610	chr17:9249690	2017	7637	1	Cause of death: asthma, unspecified	T	C	0.003	0.0578	0.01019	1.444E-08	UKBB	European
STX8	rs147672610	chr17:9249690	2017	337199	1	Alcoholic liver disease	T	C	0.003	0.006023	0.001013	2.732E-09	UKBB	European
STX8	rs191855390	chr17:9251509	2017	7637	1	Cause of death: chronic or unspecified with haemorrhage	A	G	0.005	0.02415	0.004314	2.26E-08	UKBB	European
STX8	rs185648506	chr17:9253708	2017	7637	1	Cause of death: malignant neoplasm, primary site unknown, so stated	T	C	0.002	0.1014	0.01715	3.447E-09	UKBB	European
STX8	rs144583355	chr17:9257404	2017	7637	1	Cause of death: cerebrum, except lobes and ventricles	A	G	0.997	-0.04302	0.00587	2.544E-13	UKBB	European
STX8	rs192801391	chr17:9263379	2017	337159	1	Self-reported bursitis	A	G	0.006	0.003291	0.0005582	3.733E-09	UKBB	European
STX8	rs192801391	chr17:9263379	2017	7637	1	Cause of death: perforation of intestine	A	G	0.006	0.0603	0.008041	7.17E-14	UKBB	European
STX8	rs192801391	chr17:9263379	2017	337199	1	Other congenital malformations of skin	A	G	0.006	0.003101	0.0004652	2.653E-11	UKBB	European
STX8	rs144904727	chr17:9264236	2017	7637	1	Cause of death: home	A	G	0.003	0.02008	0.002979	1.684E-11	UKBB	European
STX8	rs189325843	chr17:9264305	2017	7637	1	Cause of death: interstitial pulmonary disease, unspecified	T	C	0.997	-0.04549	0.007221	3.142E-10	UKBB	European
STX8	rs189325843	chr17:9264305	2017	7637	1	Cause of death: home	T	C	0.997	-0.02051	0.003708	3.255E-08	UKBB	European
STX8	rs567692971	chr17:9264854	2017	7637	1	Cause of death: without complications	T	C	0.001	0.05318	0.006508	3.545E-16	UKBB	European
STX8	rs184044675	chr17:9266205	2017	7637	1	Cause of death: malignant neoplasm of thyroid gland	A	G	0.999	-0.03499	0.004292	4.087E-16	UKBB	European
STX8	rs184044675	chr17:9266205	2017	7637	1	Cause of death: gastro-intestinal haemorrhage, unspecified	A	G	0.999	-0.0341	0.00569	2.142E-09	UKBB	European
STX8	rs184044675	chr17:9266205	2017	337199	1	Viral warts	A	G	0.999	-0.006106	0.001048	5.622E-09	UKBB	European
STX8	rs191480592	chr17:9266932	2017	7637	1	Cause of death: peripheral vascular disease, unspecified	T	C	0	0.03542	0.004849	3.075E-13	UKBB	European
STX8	rs145643921	chr17:9270946	2017	337199	1	Other nontraumatic intracranial haemorrhage	A	C	0.005	0.001922	0.0003496	3.855E-08	UKBB	European
STX8	rs190649298	chr17:9271416	2017	7637	1	Cause of death: septicaemia, unspecified	A	G	0.001	0.05053	0.007974	2.479E-10	UKBB	European
STX8	rs190649298	chr17:9271416	2017	7637	1	Cause of death: peritoneum, unspecified	A	G	0.001	0.07718	0.01002	1.468E-14	UKBB	European
STX8	rs548102068	chr17:9276168	2017	7637	1	Cause of death: cerebrum, except lobes and ventricles	C	G	0.995	-0.0175	0.003027	7.788E-09	UKBB	European
STX8	rs548102068	chr17:9276168	2017	337199	1	Dystonia	C	G	0.995	-0.001541	0.0002617	3.885E-09	UKBB	European
STX8	rs182152515	chr17:9279705	2017	7637	1	Cause of death: bronchiectasis	T	C	0	0.06377	0.009731	5.968E-11	UKBB	European
STX8	rs565310117	chr17:9280317	2017	7637	1	Cause of death: oropharynx, unspecified	A	G	0.004	0.02866	0.004464	1.456E-10	UKBB	European
STX8	rs138013675	chr17:9281873	2017	337159	1	Treatment with mefenamic acid	A	G	0.996	-0.005152	0.0009072	1.355E-08	UKBB	European
STX8	rs549571981	chr17:9283688	2017	337159	1	Treatment with betnesol 0.1% eye or ear/nose drops	A	C	-	-0.00247	0.0004391	1.87E-08	UKBB	European
STX8	rs183517191	chr17:9287490	2017	7637	1	Cause of death: septicaemia, unspecified	T	C	0.001	0.05226	0.008266	2.726E-10	UKBB	European
STX8	rs183517191	chr17:9287490	2017	7637	1	Cause of death: peritoneum, unspecified	T	C	0.001	0.07844	0.01038	4.698E-14	UKBB	European
STX8	rs185979418	chr17:9291846	2017	7637	1	Cause of death: appendix	T	C	-	0.01909	0.003215	2.995E-09	UKBB	European
STX8	rs35954664	chr17:9292744	2017	7637	1	Cause of death: other specified place	A	G	-	0.05	0.007801	1.546E-10	UKBB	European
STX8	rs142810279	chr17:9296390	2017	7637	1	Cause of death: peripheral vascular disease, unspecified	T	C	0.002	0.02271	0.003908	6.384E-09	UKBB	European
STX8	rs146088475	chr17:9296417	2017	7637	1	Cause of death: home	T	C	0.004	0.02008	0.002938	8.992E-12	UKBB	European
STX8	rs192761811	chr17:9298644	2017	337159	1	Treatment with ritonavir	T	C	-	0.003386	0.0004501	5.448E-14	UKBB	European
STX8	rs192761811	chr17:9298644	2017	7637	1	Cause of death: diverticular disease of intestine, part unspecified, with perforation and abscess	T	C	-	0.04263	0.007603	2.136E-08	UKBB	European
STX8	rs192761811	chr17:9298644	2017	7637	1	Cause of death: inquest adjourned death	T	C	-	0.04304	0.007209	2.473E-09	UKBB	European

STX8	rs571418204	chr17:9304589	2017	7637	1	Cause of death: oropharynx, unspecified	T	C	0.995	-0.03287	0.004755	5.169E-12	UKBB	European
STX8	rs183722951	chr17:9306937	2017	7637	1	Cause of death: mesothelioma of pleura	A	C	0.001	0.06482	0.01032	3.552E-10	UKBB	European
STX8	rs183722951	chr17:9306937	2017	7637	1	Cause of death: acute lymphoblastic leukaemia	A	C	0.001	0.03336	0.005163	1.103E-10	UKBB	European
STX8	rs183722951	chr17:9306937	2017	7637	1	Cause of death: endocarditis, valve unspecified	A	C	0.001	0.03282	0.00597	3.991E-08	UKBB	European
STX8	rs183722951	chr17:9306937	2017	337199	1	Mesothelioma	A	C	0.001	0.00191	0.0003372	1.482E-08	UKBB	European
STX8	rs531216177	chr17:9311974	2017	7637	1	Cause of death: other and unspecified intestinal obstruction	T	C	0.998	-0.03023	0.005336	1.525E-08	UKBB	European
STX8	rs182757264	chr17:9312193	2017	337159	1	Treatment with betnesol 0.1% eye or ear/nose drops	A	T	0.997	-0.002733	0.0004548	1.863E-09	UKBB	European
STX8	rs556268529	chr17:9314437	2017	337159	1	Self-reported lichen planus	A	G	-	-0.004071	0.0006887	3.395E-09	UKBB	European
STX8	rs556268529	chr17:9314437	2017	7637	1	Cause of death: chronic myeloid leukaemia	A	G	-	-0.05094	0.007376	5.401E-12	UKBB	European
STX8	rs556268529	chr17:9314437	2017	7637	1	Cause of death: fatty liver	A	G	-	-0.04998	0.007817	1.715E-10	UKBB	European
STX8	rs556268529	chr17:9314437	2017	7637	1	Cause of death: gastro-intestinal haemorrhage, unspecified	A	G	-	-0.04931	0.006899	9.735E-13	UKBB	European
STX8	rs146649689	chr17:9315688	2017	7637	1	Cause of death: malignant neoplasm of thyroid gland	T	C	0.005	0.01648	0.002968	2.893E-08	UKBB	European
STX8	rs571061278	chr17:9316432	2017	7637	1	Cause of death: malignant neoplasm of thyroid gland	A	G	0.002	0.0359	0.004358	2.048E-16	UKBB	European
STX8	rs571061278	chr17:9316432	2017	7637	1	Cause of death: gastro-intestinal haemorrhage, unspecified	A	G	0.002	0.0353	0.005778	1.054E-09	UKBB	European
STX8	rs571061278	chr17:9316432	2017	337199	1	Viral warts	A	G	0.002	0.007001	0.001009	4.04E-12	UKBB	European
STX8	rs140783904	chr17:9316441	2017	337199	1	Stroke, not specified as haemorrhage or infarction	C	G	0.001	0.007385	0.00133	2.851E-08	UKBB	European
STX8	rs181849653	chr17:9318694	2017	337199	1	Other congenital malformations of skin	T	C	0.998	-0.002261	0.0004016	0.000000018	UKBB	European
STX8	rs116864056	chr17:9319081	2017	7637	1	Cause of death: cervix uteri, unspecified	A	G	0.0099	0.01399	0.002531	3.344E-08	UKBB	European
STX8	rs113174674	chr17:9320700	2017	337159	1	Treatment with brimonidine tartrate	A	C	0	0.003117	0.000563	3.093E-08	UKBB	European
STX8	rs113174674	chr17:9320700	2017	7637	1	Cause of death: rheumatoid arthritis, unspecified	A	C	0	0.06952	0.009605	5.003E-13	UKBB	European
STX8	rs541087781	chr17:9323424	2017	7637	1	Cause of death: chronic lymphocytic leukaemia	C	G	-	0.1296	0.02086	5.527E-10	UKBB	European
STX8	rs541087781	chr17:9323424	2017	7637	1	Cause of death: alcoholic cirrhosis of liver	C	G	-	0.08208	0.01321	5.378E-10	UKBB	European
STX8	rs111320977	chr17:9324269	2017	337159	1	Treatment with brimonidine tartrate	T	C	0	0.003279	0.0005745	1.15E-08	UKBB	European
STX8	rs111320977	chr17:9324269	2017	7637	1	Cause of death: rheumatoid arthritis, unspecified	T	C	0	0.08065	0.01032	6.258E-15	UKBB	European
STX8	rs116933920	chr17:9327313	2017	337159	1	Treatment with cyclizine	T	G	0.0129	0.001341	0.0002245	2.344E-09	UKBB	European
STX8	rs540552157	chr17:9328723	2017	7637	1	Cause of death: creutzfeldt-jakob disease	A	G	-	-0.04504	0.005988	6.026E-14	UKBB	European
STX8	rs540552157	chr17:9328723	2017	337199	1	Other diseases of upper respiratory tract	A	G	-	-0.004091	0.0007003	5.152E-09	UKBB	European
STX8	rs72816196	chr17:9329335	2017	337159	1	Self-reported vitiligo	C	G	0.005	0.001811	0.0003263	2.891E-08	UKBB	European
STX8	rs147682368	chr17:9331486	2017	337159	1	Treatment with cyclizine	A	T	0.9871	-0.001378	0.0002276	1.401E-09	UKBB	European
STX8	rs10521157	chr17:9336370	2010	1727	-	Exceptional Longevity	A	G	0.2614	NA	NA	1.21E-08	2E+07	European
STX8	rs7211026	chr17:9336464	2017	7637	1	Cause of death: urinary tract infection, site not specified	C	G	0.006	0.06684	0.01211	3.489E-08	UKBB	European
STX8	rs7211026	chr17:9336464	2007	-	-	Follicle stimulating hormone	C	G	0.006	NA	NA	9.749E-13	2E+07	European
STX8	rs7211026	chr17:9336464	2007	-	-	Intra abdominal fat	C	G	0.006	NA	NA	4.093E-08	2E+07	European
STX8	rs7211026	chr17:9336464	2007	-	-	Intra abdominal fat	C	G	0.006	NA	NA	4.312E-08	2E+07	European
STX8	rs539616890	chr17:9337413	2017	7637	1	Cause of death: endocarditis, valve unspecified	A	C	0.001	0.05044	0.007331	6.436E-12	UKBB	European
STX8	rs78671435	chr17:9338503	2017	337199	1	Bacterial pneumonia	T	C	0.995	-0.004732	0.0007692	7.68E-10	UKBB	European
STX8	rs8065709	chr17:9339793	2017	337199	1	Bacterial pneumonia	C	G	0.005	0.004793	0.0007736	5.793E-10	UKBB	European
STX8	rs8071502	chr17:9339841	2017	337199	1	Bacterial pneumonia	T	C	0.995	-0.004475	0.0007496	2.375E-09	UKBB	European
STX8	rs545219137	chr17:9340981	2017	7637	1	Cause of death: inquest adjourned death	A	G	0.002	0.04984	0.007744	1.304E-10	UKBB	European
STX8	rs191355256	chr17:9342200	2017	7637	1	Cause of death: endocarditis, valve unspecified	A	C	0.006	0.02041	0.003393	1.886E-09	UKBB	European
STX8	rs9900029	chr17:9343888	2017	7637	1	Cause of death: urinary tract infection, site not specified	C	G	0.006	0.08352	0.01356	7.626E-10	UKBB	European
STX8	rs541189374	chr17:9343986	2017	7637	1	Cause of death: thoracic aortic aneurysm, ruptured	T	C	-	0.04556	0.006531	3.318E-12	UKBB	European
STX8	rs112383718	chr17:9344591	2017	7637	1	Cause of death: aortic stenosis	A	G	0.003	0.09052	0.01607	1.826E-08	UKBB	European
STX8	rs559323534	chr17:9351654	2017	7637	1	Cause of death: atrial fibrillation and flutter	T	C	0.001	0.06548	0.01033	2.489E-10	UKBB	European
STX8	rs559323534	chr17:9351654	2017	337199	1	Bacterial pneumonia	T	C	0.001	0.005226	0.0008287	2.867E-10	UKBB	European
STX8	rs191245794	chr17:9353234	2017	7637	1	Cause of death: tongue, unspecified	A	G	0.002	0.05132	0.008733	4.376E-09	UKBB	European
STX8	rs191245794	chr17:9353234	2017	7637	1	Cause of death: brain, unspecified	A	G	0.002	0.05111	0.008329	8.857E-10	UKBB	European
STX8	rs543482931	chr17:9356913	2017	7637	1	Cause of death: cervix uteri, unspecified	A	G	0.002	0.02712	0.0041	3.979E-11	UKBB	European
STX8	rs141668819	chr17:9358127	2017	337159	1	Self-reported pulmonary fibrosis	T	C	1	-0.002731	0.0003462	3.066E-15	UKBB	European
STX8	rs141668819	chr17:9358127	2017	337159	1	Treatment with securon 40mg tablet	T	C	1	-0.002183	0.0003481	3.608E-10	UKBB	European
STX8	rs141668819	chr17:9358127	2017	337159	1	Treatment with solpadol caplet	T	C	1	-0.003643	0.0006073	1.983E-09	UKBB	European
STX8	rs117260302	chr17:9361465	2017	7637	1	Cause of death: cervix uteri, unspecified	T	G	0.002	0.02637	0.00404	7.173E-11	UKBB	European
STX8	rs55807461	chr17:9361483	2017	337159	1	Treatment with isotard 25xl m or r tablet	T	C	0.995	-0.002355	0.0004242	2.835E-08	UKBB	European
STX8	rs2125352	chr17:9361668	2017	337159	1	Self-reported pulmonary fibrosis	A	G	0.996	-0.001672	0.0002614	1.597E-10	UKBB	European
STX8	rs2125352	chr17:9361668	2017	7637	1	Cause of death: home	A	G	0.996	-0.02445	0.003184	1.819E-14	UKBB	European
STX8	rs563573301	chr17:9365091	2017	7637	1	Cause of death: rheumatoid arthritis, unspecified	A	T	0.996	-0.02643	0.00428	6.974E-10	UKBB	European
STX8	rs566362084	chr17:9371642	2017	7637	1	Cause of death: alcoholic liver disease, unspecified	C	G	0.002	0.1144	0.01899	1.763E-09	UKBB	European
STX8	rs548534821	chr17:9374346	2017	7637	1	Cause of death: home	T	G	-	-0.03136	0.004994	3.555E-10	UKBB	European

STX8	rs147911333	chr17:9375809	2017	7637	1	Cause of death: cervix uteri, unspecified	C	G	0.002	0.02606	0.004017	9.215E-11	UKBB	European
STX8	rs191141303	chr17:9376490	2017	337159	1	Treatment with isosorbide dinitrate	A	C	0.001	0.002733	0.0004899	2.425E-08	UKBB	European
STX8	rs191141303	chr17:9376490	2017	7637	1	Cause of death: acute lymphoblastic leukaemia	A	C	0.001	0.04936	0.006732	2.504E-13	UKBB	European
STX8	rs72818045	chr17:9377412	2017	337159	1	Treatment with isotard 25xl m or r tablet	T	C	0.004	0.002568	0.0004296	2.264E-09	UKBB	European
STX8	rs192970442	chr17:9380274	2017	7637	1	Cause of death: pharynx, unspecified	A	G	0.002	0.0363	0.005943	1.061E-09	UKBB	European
STX8	rs147498090	chr17:9382665	2017	7637	1	Cause of death: cervix uteri, unspecified	A	G	0.999	-0.02525	0.00396	1.929E-10	UKBB	European
STX8	rs145253691	chr17:9385042	2017	337159	1	Self-reported spinal stenosis	A	G	0.003	0.003759	0.0005174	3.72E-13	UKBB	European
STX8	rs145253691	chr17:9385042	2017	7637	1	Cause of death: acute and subacute infective endocarditis	A	G	0.003	0.06056	0.006307	1.031E-21	UKBB	European
STX8	rs145253691	chr17:9385042	2017	337199	1	Disorders of tooth development and eruption	A	G	0.003	0.002902	0.0005174	2.038E-08	UKBB	European
STX8	rs7209910	chr17:9385150	2017	337159	1	Treatment with ketoprofen	A	G	0.0229	0.001225	0.0002081	3.943E-09	UKBB	European
STX8	rs766953720	chr17:9385494	2017	337159	1	Treatment with salbutamol+ipratropium 100micrograms or 20micrograms inhaler	T	C	-	-0.003425	0.0005632	1.192E-09	UKBB	European
STX8	rs539427744	chr17:9389725	2017	7637	1	Cause of death: calculus of gallbladder without cholecystitis	A	G	0	0.04663	0.007656	1.179E-09	UKBB	European
STX8	rs544154461	chr17:9390741	2017	7637	1	Cause of death: unspecified place	A	C	0	0.03527	0.00616	1.067E-08	UKBB	European
STX8	rs184448687	chr17:9394369	2017	337159	1	Treatment with avodart 500micrograms capsule	T	C	0.003	0.001613	0.0002786	7.12E-09	UKBB	European
STX8	rs544921674	chr17:9404148	2017	7637	1	Cause of death: malignant neoplasm of ureter	T	C	0.999	-0.08361	0.01064	4.381E-15	UKBB	European
STX8	rs544921674	chr17:9404148	2017	7637	1	Cause of death: peripheral vascular disease, unspecified	T	C	0.999	-0.09197	0.007895	4.245E-31	UKBB	European
STX8	rs745959807	chr17:9405383	2017	7637	1	Cause of death: emphysema, unspecified	A	G	-	-0.05236	0.007952	4.875E-11	UKBB	European
STX8	rs563747261	chr17:9406625	2017	337159	1	Self-reported incisional hernia	T	G	1	-0.003159	0.0004673	1.38E-11	UKBB	European
STX8	rs563747261	chr17:9406625	2017	337159	1	Treatment with hydroxocobalamin product	T	G	1	-0.003009	0.0005271	1.145E-08	UKBB	European
STX8	rs563747261	chr17:9406625	2017	7637	1	Cause of death: myelodysplastic syndrome, unspecified	T	G	1	-0.04906	0.008109	1.516E-09	UKBB	European
STX8	rs143206911	chr17:9407638	2017	337199	1	Superficial injury of lower leg	A	G	0.998	-0.003918	0.0006899	1.355E-08	UKBB	European
STX8	rs568044104	chr17:9414049	2017	337159	1	Treatment with enalapril maleate+hydrochlorothiazide 20mg or 12.5mg tablet	A	C	-	-0.003462	0.0005881	3.969E-09	UKBB	European
STX8	rs568044104	chr17:9414049	2017	337159	1	Treatment with minims artificial tears single-use eye drops	A	C	-	-0.003482	0.000535	7.628E-11	UKBB	European
STX8	rs568044104	chr17:9414049	2017	7637	1	Cause of death: malignant neoplasm, unspecified	A	C	-	-0.0619	0.009837	3.287E-10	UKBB	European
STX8	rs535468806	chr17:9414185	2017	337159	1	Self-reported incisional hernia	T	G	0	0.003237	0.0004711	6.369E-12	UKBB	European
STX8	rs535468806	chr17:9414185	2017	337159	1	Treatment with hydroxocobalamin product	T	G	0	0.003138	0.0005315	3.556E-09	UKBB	European
STX8	rs183922500	chr17:9420291	2017	337159	1	Treatment with colestyramine	A	G	0.001	0.003789	0.0005649	1.969E-11	UKBB	European
STX8	rs183922500	chr17:9420291	2017	7637	1	Cause of death: brain, unspecified	A	G	0.001	0.05456	0.008573	2.078E-10	UKBB	European
STX8	rs183922500	chr17:9420291	2017	7637	1	Cause of death: chronic obstructive pulmonary disease with acute exacerbation, unspecified	A	G	0.001	0.1086	0.01482	2.511E-13	UKBB	European
STX8	rs183922500	chr17:9420291	2017	7637	1	Cause of death: calculus of gallbladder without cholecystitis	A	G	0.001	0.05192	0.008133	1.825E-10	UKBB	European
STX8	rs117187418	chr17:9429555	2017	337159	1	Self-reported pulmonary fibrosis	T	G	0.997	-0.002081	0.0003105	2.049E-11	UKBB	European
STX8	rs117187418	chr17:9429555	2017	337159	1	Treatment with rhumalgan 25mg e or c tablet	T	G	0.997	-0.001482	0.0002715	4.743E-08	UKBB	European
STX8	rs369035657	chr17:9432949	2017	337159	1	Self-reported tennis elbow or lateral epicondylitis	A	G	0.998	-0.003354	0.0005968	1.921E-08	UKBB	European
STX8	rs144374455	chr17:9433724	2017	7637	1	Cause of death: cardia	T	C	0.9911	-0.03386	0.005129	4.32E-11	UKBB	European
STX8	rs151173788	chr17:9435042	2017	7637	1	Cause of death: other specified respiratory disorders	T	C	0.995	-0.01688	0.003007	2.04E-08	UKBB	European
STX8	rs185134681	chr17:9435496	2017	7637	1	Cause of death: pharynx, unspecified	T	C	0.0119	0.0184	0.003046	1.621E-09	UKBB	European
STX8	rs192449623	chr17:9441778	2017	7637	1	Cause of death: cerebrovascular disease, unspecified	C	G	0.005	0.05177	0.008768	3.68E-09	UKBB	European
STX8	rs145780944	chr17:9443283	2017	7637	1	Cause of death: chronic or unspecified with haemorrhage	A	T	0.002	0.03444	0.005177	3.076E-11	UKBB	European
STX8	rs142703184	chr17:9448137	2017	7637	1	Cause of death: pharynx, unspecified	A	G	0.9891	-0.03064	0.003959	1.131E-14	UKBB	European
STX8	rs141761084	chr17:9451668	2017	7637	1	Cause of death: chronic or unspecified with haemorrhage	T	C	0.001	0.03044	0.004903	5.61E-10	UKBB	European
STX8	rs546522989	chr17:9459902	2017	7637	1	Cause of death: cerebrovascular disease, unspecified	A	G	0.002	0.04451	0.008137	4.635E-08	UKBB	European
STX8	rs4488492	chr17:9467552	2014	8631	5	Linoleic acid 18:2n6	C	G	0.0875	2.037	0.3137	8.417E-11	2E+07	European
STX8	rs527991608	chr17:9475041	2017	337159	1	Self-reported pyloric stenosis	C	G	0.001	0.004502	0.0005848	1.383E-14	UKBB	European
STX8	rs527991608	chr17:9475041	2017	7637	1	Cause of death: atrial fibrillation and flutter	C	G	0.001	0.05219	0.009194	1.428E-08	UKBB	European
STX8	rs141094888	chr17:9477903	2017	337159	1	Self-reported pulmonary fibrosis	A	G	0.003	0.001825	0.0003169	8.451E-09	UKBB	European
TNFSF8	rs762343491	chr9:117656516	2017	7637	1	Cause of death: pharynx, unspecified	A	C	-	0.05238	0.006991	7.548E-14	UKBB	European
TNFSF8	rs762343491	chr9:117656516	2017	7637	1	Cause of death: motor neuron disease	A	C	-	0.1676	0.0248	1.477E-11	UKBB	European
TNFSF8	rs3833492	chr9:117656633	2016	173480	2	Eosinophil count	C	CCTG	0.5567	0.02216	0.003574	5.581E-10	3E+07	European
TNFSF8	rs3833492	chr9:117656633	2016	173480	2	Sum eosinophil basophil counts	C	CCTG	0.5567	0.0211	0.003578	3.714E-09	3E+07	European
TNFSF8	rs3833492	chr9:117656633	2016	173480	2	Eosinophil percentage of white cells	C	CCTG	0.5567	0.02461	0.003571	5.457E-12	3E+07	European
TNFSF8	rs3833492	chr9:117656633	2016	173480	2	Eosinophil percentage of granulocytes	C	CCTG	0.5567	0.02513	0.003586	2.4E-12	3E+07	European
TNFSF8	rs3833492	chr9:117656633	2016	173480	2	Neutrophil percentage of granulocytes	C	CCTG	0.5567	-0.02435	0.003586	1.117E-11	3E+07	European
TNFSF8	rs78689526	chr9:117656773	2017	7637	1	Cause of death: alcoholic hepatitis	A	G	0.003	0.03196	0.00543	4.104E-09	UKBB	European
TNFSF8	rs11353389	chr9:117659544	2016	173480	2	Eosinophil count	G	GT	0.4433	-0.02208	0.003573	6.47E-10	3E+07	European
TNFSF8	rs11353389	chr9:117659544	2016	173480	2	Sum eosinophil basophil counts	G	GT	0.4433	-0.02117	0.003578	3.276E-09	3E+07	European
TNFSF8	rs11353389	chr9:117659544	2016	173480	2	Eosinophil percentage of white cells	G	GT	0.4433	-0.02437	0.00357	8.705E-12	3E+07	European
TNFSF8	rs11353389	chr9:117659544	2016	173480	2	Eosinophil percentage of granulocytes	G	GT	0.4433	-0.02482	0.003585	4.459E-12	3E+07	European
TNFSF8	rs11353389	chr9:117659544	2016	173480	2	Neutrophil percentage of granulocytes	G	GT	0.4433	0.02405	0.003585	1.972E-11	3E+07	European

TNFSF8	rs10667388	chr9:117659573	2016	173480	2	Eosinophil count	C	CTTAT	0.5567	0.02266	0.003575	2.308E-10	3E+07	European
TNFSF8	rs10667388	chr9:117659573	2016	173480	2	Sum eosinophil basophil counts	C	CTTAT	0.5567	0.02159	0.003579	1.601E-09	3E+07	European
TNFSF8	rs10667388	chr9:117659573	2016	173480	2	Eosinophil percentage of white cells	C	CTTAT	0.5567	0.02543	0.003571	1.066E-12	3E+07	European
TNFSF8	rs10667388	chr9:117659573	2016	173480	2	Eosinophil percentage of granulocytes	C	CTTAT	0.5567	0.02612	0.003587	3.249E-13	3E+07	European
TNFSF8	rs10667388	chr9:117659573	2016	173480	2	Neutrophil percentage of granulocytes	C	CTTAT	0.5567	-0.0254	0.003587	1.428E-12	3E+07	European
TNFSF8	rs33943117	chr9:117659574	2016	173480	2	Eosinophil count	T	TTTTT	-	0.0226	0.003641	5.338E-10	3E+07	European
TNFSF8	rs33943117	chr9:117659574	2016	173480	2	Sum eosinophil basophil counts	T	TTTTT	-	0.02178	0.003645	2.309E-09	3E+07	European
TNFSF8	rs33943117	chr9:117659574	2016	173480	2	Eosinophil percentage of white cells	T	TTTTT	-	0.02472	0.003637	1.079E-11	3E+07	European
TNFSF8	rs33943117	chr9:117659574	2016	173480	2	Eosinophil percentage of granulocytes	T	TTTTT	-	0.02504	0.003653	7.16E-12	3E+07	European
TNFSF8	rs33943117	chr9:117659574	2016	173480	2	Neutrophil percentage of granulocytes	T	TTTTT	-	-0.0246	0.003653	1.642E-11	3E+07	European
TNFSF8	rs10982445	chr9:117659691	2016	173480	2	Eosinophil count	T	C	0.5557	0.02285	0.003573	1.602E-10	3E+07	European
TNFSF8	rs10982445	chr9:117659691	2016	173480	2	Sum eosinophil basophil counts	T	C	0.5557	0.02175	0.003577	1.194E-09	3E+07	European
TNFSF8	rs10982445	chr9:117659691	2016	173480	2	Eosinophil percentage of white cells	T	C	0.5557	0.02551	0.003569	8.815E-13	3E+07	European
TNFSF8	rs10982445	chr9:117659691	2016	173480	2	Eosinophil percentage of granulocytes	T	C	0.5557	0.02621	0.003585	2.651E-13	3E+07	European
TNFSF8	rs10982445	chr9:117659691	2016	173480	2	Neutrophil percentage of granulocytes	T	C	0.5557	-0.02546	0.003585	1.233E-12	3E+07	European
TNFSF8	rs34397268	chr9:117660286	2016	173480	2	Eosinophil count	A	AT	0.4433	-0.02204	0.003627	1.225E-09	3E+07	European
TNFSF8	rs34397268	chr9:117660286	2016	173480	2	Sum eosinophil basophil counts	A	AT	0.4433	-0.02089	0.003631	8.833E-09	3E+07	European
TNFSF8	rs34397268	chr9:117660286	2016	173480	2	Eosinophil percentage of white cells	A	AT	0.4433	-0.02418	0.003624	2.503E-11	3E+07	European
TNFSF8	rs34397268	chr9:117660286	2016	173480	2	Eosinophil percentage of granulocytes	A	AT	0.4433	-0.02464	0.003639	1.275E-11	3E+07	European
TNFSF8	rs34397268	chr9:117660286	2016	173480	2	Neutrophil percentage of granulocytes	A	AT	0.4433	0.02354	0.003639	9.828E-11	3E+07	European
TNFSF8	rs72371482	chr9:117660456	2016	173480	2	Eosinophil count	G	GTAT	0.4414	-0.02204	0.003571	6.729E-10	3E+07	European
TNFSF8	rs72371482	chr9:117660456	2016	173480	2	Sum eosinophil basophil counts	G	GTAT	0.4414	-0.021	0.003575	4.27E-09	3E+07	European
TNFSF8	rs72371482	chr9:117660456	2016	173480	2	Eosinophil percentage of white cells	G	GTAT	0.4414	-0.02454	0.003568	5.999E-12	3E+07	European
TNFSF8	rs72371482	chr9:117660456	2016	173480	2	Eosinophil percentage of granulocytes	G	GTAT	0.4414	-0.02511	0.003583	2.417E-12	3E+07	European
TNFSF8	rs72371482	chr9:117660456	2016	173480	2	Neutrophil percentage of granulocytes	G	GTAT	0.4414	0.02435	0.003583	1.07E-11	3E+07	European
TNFSF8	rs4979471	chr9:117660562	2016	173480	2	Eosinophil count	C	G	0.5557	0.02247	0.003572	3.127E-10	3E+07	European
TNFSF8	rs4979471	chr9:117660562	2016	173480	2	Sum eosinophil basophil counts	C	G	0.5557	0.02142	0.003576	2.084E-09	3E+07	European
TNFSF8	rs4979471	chr9:117660562	2016	173480	2	Eosinophil percentage of white cells	C	G	0.5557	0.02524	0.003568	1.526E-12	3E+07	European
TNFSF8	rs4979471	chr9:117660562	2016	173480	2	Eosinophil percentage of granulocytes	C	G	0.5557	0.02593	0.003584	4.666E-13	3E+07	European
TNFSF8	rs4979471	chr9:117660562	2016	173480	2	Neutrophil percentage of granulocytes	C	G	0.5557	-0.02521	0.003584	2.023E-12	3E+07	European
TNFSF8	rs5003740	chr9:117660601	2012	963	-	Primary biliary cirrhosis	C	G	0.1869	NA	NA	2.86E-11	2E+07	East Asian
TNFSF8	rs1322067	chr9:117660933	2016	173480	2	Eosinophil count	A	G	0.5596	0.0222	0.003575	5.315E-10	3E+07	European
TNFSF8	rs1322067	chr9:117660933	2016	173480	2	Sum eosinophil basophil counts	A	G	0.5596	0.02116	0.00358	3.382E-09	3E+07	European
TNFSF8	rs1322067	chr9:117660933	2016	173480	2	Eosinophil percentage of white cells	A	G	0.5596	0.02477	0.003572	4.097E-12	3E+07	European
TNFSF8	rs1322067	chr9:117660933	2016	173480	2	Eosinophil percentage of granulocytes	A	G	0.5596	0.02541	0.003587	1.419E-12	3E+07	European
TNFSF8	rs1322067	chr9:117660933	2016	173480	2	Neutrophil percentage of granulocytes	A	G	0.5596	-0.02467	0.003587	6.076E-12	3E+07	European
TNFSF8	rs72756567	chr9:117661625	2016	173480	2	Eosinophil count	A	T	0.5547	0.02234	0.003583	4.47E-10	3E+07	European
TNFSF8	rs72756567	chr9:117661625	2016	173480	2	Sum eosinophil basophil counts	A	T	0.5547	0.02133	0.003587	2.748E-09	3E+07	European
TNFSF8	rs72756567	chr9:117661625	2016	173480	2	Eosinophil percentage of white cells	A	T	0.5547	0.02509	0.003579	2.387E-12	3E+07	European
TNFSF8	rs72756567	chr9:117661625	2016	173480	2	Eosinophil percentage of granulocytes	A	T	0.5547	0.02579	0.003594	7.292E-13	3E+07	European
TNFSF8	rs72756567	chr9:117661625	2016	173480	2	Neutrophil percentage of granulocytes	A	T	0.5547	-0.02508	0.003594	3.009E-12	3E+07	European
TNFSF8	rs7025577	chr9:117661665	2016	173480	2	Eosinophil count	A	C	0.4463	-0.02242	0.003575	3.607E-10	3E+07	European
TNFSF8	rs7025577	chr9:117661665	2016	173480	2	Sum eosinophil basophil counts	A	C	0.4463	-0.02139	0.003579	2.297E-09	3E+07	European
TNFSF8	rs7025577	chr9:117661665	2016	173480	2	Eosinophil percentage of white cells	A	C	0.4463	-0.02507	0.003572	2.244E-12	3E+07	European
TNFSF8	rs7025577	chr9:117661665	2016	173480	2	Eosinophil percentage of granulocytes	A	C	0.4463	-0.02579	0.003587	6.538E-13	3E+07	European
TNFSF8	rs7025577	chr9:117661665	2016	173480	2	Neutrophil percentage of granulocytes	A	C	0.4463	0.02507	0.003587	2.784E-12	3E+07	European
TNFSF8	rs6478117	chr9:117662144	2016	173480	2	Eosinophil count	A	G	0.5577	0.02196	0.003574	8.092E-10	3E+07	European
TNFSF8	rs6478117	chr9:117662144	2016	173480	2	Sum eosinophil basophil counts	A	G	0.5577	0.02085	0.003578	5.685E-09	3E+07	European
TNFSF8	rs6478117	chr9:117662144	2016	173480	2	Eosinophil percentage of white cells	A	G	0.5577	0.02448	0.003571	7.144E-12	3E+07	European
TNFSF8	rs6478117	chr9:117662144	2016	173480	2	Eosinophil percentage of granulocytes	A	G	0.5577	0.025	0.003586	3.148E-12	3E+07	European
TNFSF8	rs6478117	chr9:117662144	2016	173480	2	Neutrophil percentage of granulocytes	A	G	0.5577	-0.02411	0.003586	1.76E-11	3E+07	European
TNFSF8	rs118117537	chr9:117662268	2017	7637	1	Cause of death: alcoholic hepatitis	T	C	0.996	-0.0321	0.005431	3.541E-09	UKBB	European
TNFSF8	rs75264082	chr9:117662389	2017	7637	1	Cause of death: alcoholic hepatitis	T	C	0.003	0.03206	0.005428	3.647E-09	UKBB	European
TNFSF8	rs7030090	chr9:117662730	2016	173480	2	Eosinophil count	T	C	0.4423	-0.02002	0.003494	1.013E-08	3E+07	European
TNFSF8	rs7030090	chr9:117662730	2016	173480	2	Sum eosinophil basophil counts	T	C	0.4423	-0.01916	0.003498	4.347E-08	3E+07	European
TNFSF8	rs7030090	chr9:117662730	2016	173480	2	Eosinophil percentage of white cells	T	C	0.4423	-0.02245	0.003491	1.275E-10	3E+07	European
TNFSF8	rs7030090	chr9:117662730	2016	173480	2	Eosinophil percentage of granulocytes	T	C	0.4423	-0.02288	0.003506	6.762E-11	3E+07	European
TNFSF8	rs7030090	chr9:117662730	2016	173480	2	Neutrophil percentage of granulocytes	T	C	0.4423	0.02222	0.003506	2.303E-10	3E+07	European

TNFSF8	rs7858603	chr9:117663270	2016	173480	2	Eosinophil count	C	G	0.4423	-0.02236	0.003575	3.996E-10	3E+07	European
TNFSF8	rs7858603	chr9:117663270	2016	173480	2	Sum eosinophil basophil counts	C	G	0.4423	-0.02133	0.003579	2.518E-09	3E+07	European
TNFSF8	rs7858603	chr9:117663270	2016	173480	2	Eosinophil percentage of white cells	C	G	0.4423	-0.02486	0.003572	3.367E-12	3E+07	European
TNFSF8	rs7858603	chr9:117663270	2016	173480	2	Eosinophil percentage of granulocytes	C	G	0.4423	-0.02547	0.003587	1.242E-12	3E+07	European
TNFSF8	rs7858603	chr9:117663270	2016	173480	2	Neutrophil percentage of granulocytes	C	G	0.4423	0.02474	0.003587	5.27E-12	3E+07	European
TNFSF8	rs3181202	chr9:117663550	2016	173480	2	Eosinophil count	T	C	0.5577	0.02228	0.003575	4.552E-10	3E+07	European
TNFSF8	rs3181202	chr9:117663550	2016	173480	2	Sum eosinophil basophil counts	T	C	0.5577	0.02132	0.003579	2.566E-09	3E+07	European
TNFSF8	rs3181202	chr9:117663550	2016	173480	2	Eosinophil percentage of white cells	T	C	0.5577	0.0247	0.003571	4.6E-12	3E+07	European
TNFSF8	rs3181202	chr9:117663550	2016	173480	2	Eosinophil percentage of granulocytes	T	C	0.5577	0.0253	0.003586	1.745E-12	3E+07	European
TNFSF8	rs3181202	chr9:117663550	2016	173480	2	Neutrophil percentage of granulocytes	T	C	0.5577	-0.02461	0.003587	6.822E-12	3E+07	European
TNFSF8	rs3181201	chr9:117663800	2016	173480	2	Eosinophil count	C	G	0.4423	-0.02215	0.003575	5.751E-10	3E+07	European
TNFSF8	rs3181201	chr9:117663800	2016	173480	2	Sum eosinophil basophil counts	C	G	0.4423	-0.02116	0.003579	3.368E-09	3E+07	European
TNFSF8	rs3181201	chr9:117663800	2016	173480	2	Eosinophil percentage of white cells	C	G	0.4423	-0.02457	0.003571	6.029E-12	3E+07	European
TNFSF8	rs3181201	chr9:117663800	2016	173480	2	Eosinophil percentage of granulocytes	C	G	0.4423	-0.02515	0.003586	2.322E-12	3E+07	European
TNFSF8	rs3181201	chr9:117663800	2016	173480	2	Neutrophil percentage of granulocytes	C	G	0.4423	0.02443	0.003586	9.674E-12	3E+07	European
TNFSF8	rs3181201	chr9:117663800	2012	963	-	Primary biliary cirrhosis	C	G	0.4423	NA	NA	3.13E-10	2E+07	East Asian
TNFSF8	rs3181200	chr9:117663884	2016	173480	2	Eosinophil count	T	G	0.4423	-0.02223	0.003575	4.986E-10	3E+07	European
TNFSF8	rs3181200	chr9:117663884	2016	173480	2	Sum eosinophil basophil counts	T	G	0.4423	-0.02126	0.003579	2.841E-09	3E+07	European
TNFSF8	rs3181200	chr9:117663884	2016	173480	2	Eosinophil percentage of white cells	T	G	0.4423	-0.02463	0.003571	5.309E-12	3E+07	European
TNFSF8	rs3181200	chr9:117663884	2016	173480	2	Eosinophil percentage of granulocytes	T	G	0.4423	-0.02522	0.003586	2.05E-12	3E+07	European
TNFSF8	rs3181200	chr9:117663884	2016	173480	2	Neutrophil percentage of granulocytes	T	G	0.4423	0.02452	0.003586	8.152E-12	3E+07	European
TNFSF8	rs2974	chr9:117664172	2012	963	1	Primary biliary cholangitis	T	C	0.5577	-0.437	NA	0	2E+07	East Asian
TNFSF8	rs2974	chr9:117664172	2016	173480	2	Eosinophil count	T	C	0.5577	0.02218	0.003575	5.476E-10	3E+07	European
TNFSF8	rs2974	chr9:117664172	2016	173480	2	Sum eosinophil basophil counts	T	C	0.5577	0.02116	0.003579	3.378E-09	3E+07	European
TNFSF8	rs2974	chr9:117664172	2016	173480	2	Eosinophil percentage of white cells	T	C	0.5577	0.02462	0.003571	5.395E-12	3E+07	European
TNFSF8	rs2974	chr9:117664172	2016	173480	2	Eosinophil percentage of granulocytes	T	C	0.5577	0.02522	0.003587	2.03E-12	3E+07	European
TNFSF8	rs2974	chr9:117664172	2016	173480	2	Neutrophil percentage of granulocytes	T	C	0.5577	-0.02449	0.003587	8.632E-12	3E+07	European
TNFSF8	rs2295800	chr9:117664211	2016	173480	2	Eosinophil count	T	C	0.5577	0.02219	0.003576	5.468E-10	3E+07	European
TNFSF8	rs2295800	chr9:117664211	2016	173480	2	Sum eosinophil basophil counts	T	C	0.5577	0.0212	0.00358	3.191E-09	3E+07	European
TNFSF8	rs2295800	chr9:117664211	2016	173480	2	Eosinophil percentage of white cells	T	C	0.5577	0.02466	0.003572	5.095E-12	3E+07	European
TNFSF8	rs2295800	chr9:117664211	2016	173480	2	Eosinophil percentage of granulocytes	T	C	0.5577	0.02528	0.003588	1.844E-12	3E+07	European
TNFSF8	rs2295800	chr9:117664211	2016	173480	2	Neutrophil percentage of granulocytes	T	C	0.5577	-0.02457	0.003588	7.397E-12	3E+07	European
TNFSF8	rs2295800	chr9:117664211	2005	846	-	Crohns disease	T	C	0.5577	NA	NA	3.06E-08	2E+07	East Asian
TNFSF8	rs77691228	chr9:117664380	2017	7637	1	Cause of death: alcoholic hepatitis	T	C	0.003	0.0326	0.00547	2.649E-09	UKBB	European
TNFSF8	rs140962100	chr9:117664616	2017	7637	1	Cause of death: multisystem degeneration	T	G	0.995	-0.02213	0.003502	2.762E-10	UKBB	European
TNFSF8	rs3181374	chr9:117665187	2016	173480	2	Eosinophil count	A	G	0.5547	0.02195	0.003573	8.039E-10	3E+07	European
TNFSF8	rs3181374	chr9:117665187	2016	173480	2	Sum eosinophil basophil counts	A	G	0.5547	0.0209	0.003577	5.079E-09	3E+07	European
TNFSF8	rs3181374	chr9:117665187	2016	173480	2	Eosinophil percentage of white cells	A	G	0.5547	0.02424	0.003569	1.102E-11	3E+07	European
TNFSF8	rs3181374	chr9:117665187	2016	173480	2	Eosinophil percentage of granulocytes	A	G	0.5547	0.02477	0.003584	4.858E-12	3E+07	European
TNFSF8	rs3181374	chr9:117665187	2016	173480	2	Neutrophil percentage of granulocytes	A	G	0.5547	-0.02406	0.003584	1.917E-11	3E+07	European
TNFSF8	rs76486122	chr9:117665193	2017	7637	1	Cause of death: alcoholic hepatitis	T	C	0.003	0.03225	0.005433	3.04E-09	UKBB	European
TNFSF8	rs116851933	chr9:117665357	2017	7637	1	Cause of death: alcoholic hepatitis	T	C	0.003	0.03227	0.005434	3.003E-09	UKBB	European
TNFSF8	rs1126711	chr9:117665379	2016	173480	2	Eosinophil count	A	G	0.5547	0.02202	0.003573	7.104E-10	3E+07	European
TNFSF8	rs1126711	chr9:117665379	2016	173480	2	Sum eosinophil basophil counts	A	G	0.5547	0.02099	0.003577	4.381E-09	3E+07	European
TNFSF8	rs1126711	chr9:117665379	2016	173480	2	Eosinophil percentage of white cells	A	G	0.5547	0.02433	0.00357	9.407E-12	3E+07	European
TNFSF8	rs1126711	chr9:117665379	2016	173480	2	Eosinophil percentage of granulocytes	A	G	0.5547	0.02481	0.003585	4.446E-12	3E+07	European
TNFSF8	rs1126711	chr9:117665379	2016	173480	2	Neutrophil percentage of granulocytes	A	G	0.5547	-0.02411	0.003585	1.758E-11	3E+07	European
TNFSF8	rs3181372	chr9:117665435	2016	173480	2	Eosinophil count	A	G	0.5517	0.02241	0.003575	3.599E-10	3E+07	European
TNFSF8	rs3181372	chr9:117665435	2016	173480	2	Sum eosinophil basophil counts	A	G	0.5517	0.02136	0.003579	2.393E-09	3E+07	European
TNFSF8	rs3181372	chr9:117665435	2016	173480	2	Eosinophil percentage of white cells	A	G	0.5517	0.02495	0.003571	2.825E-12	3E+07	European
TNFSF8	rs3181372	chr9:117665435	2016	173480	2	Eosinophil percentage of granulocytes	A	G	0.5517	0.02558	0.003587	9.969E-13	3E+07	European
TNFSF8	rs3181372	chr9:117665435	2016	173480	2	Neutrophil percentage of granulocytes	A	G	0.5517	-0.02487	0.003587	4.076E-12	3E+07	European
TNFSF8	rs3181372	chr9:117665435	2012	963	-	Primary biliary cirrhosis	A	G	0.5517	NA	NA	3.69E-08	2E+07	East Asian
TNFSF8	rs3181371	chr9:117665570	2016	173480	2	Eosinophil percentage of white cells	C	G	0.5964	0.02035	0.003628	2.044E-08	3E+07	European
TNFSF8	rs3181371	chr9:117665570	2016	173480	2	Eosinophil percentage of granulocytes	C	G	0.5964	0.02156	0.003644	3.277E-09	3E+07	European
TNFSF8	rs3181371	chr9:117665570	2016	173480	2	Neutrophil percentage of granulocytes	C	G	0.5964	-0.02095	0.003644	9.018E-09	3E+07	European
TNFSF8	rs188416662	chr9:117665600	2017	7637	1	Cause of death: appendix	A	G	0.003	0.02561	0.004119	5.279E-10	UKBB	European
TNFSF8	rs3181370	chr9:117665752	2016	173480	2	Eosinophil percentage of white cells	T	C	0.5974	0.02084	0.003629	9.371E-09	3E+07	European

TNFSF8	rs3181370	chr9:117665752	2016	173480	2	Eosinophil percentage of granulocytes	T	C	0.5974	0.02218	0.003644	1.151E-09	3E+07	European
TNFSF8	rs3181370	chr9:117665752	2016	173480	2	Neutrophil percentage of granulocytes	T	C	0.5974	-0.02161	0.003645	3.053E-09	3E+07	European
TNFSF8	rs3181369	chr9:117665866	2016	173480	2	Eosinophil percentage of white cells	A	G	0.4026	-0.02083	0.003629	9.464E-09	3E+07	European
TNFSF8	rs3181369	chr9:117665866	2016	173480	2	Eosinophil percentage of granulocytes	A	G	0.4026	-0.02215	0.003644	1.212E-09	3E+07	European
TNFSF8	rs3181369	chr9:117665866	2016	173480	2	Neutrophil percentage of granulocytes	A	G	0.4026	0.02158	0.003644	3.173E-09	3E+07	European
TNFSF8	rs760306687	chr9:117665889	2017	7637	1	Cause of death: caecum	A	C	-	0.1048	0.01578	3.336E-11	UKBB	European
TNFSF8	rs760306687	chr9:117665889	2017	7637	1	Cause of death: secondary malignant neoplasm of retroperitoneum and peritoneum	A	C	-	0.05514	0.008135	1.305E-11	UKBB	European
TNFSF8	rs760306687	chr9:117665889	2017	337199	1	Somnolence, stupor and coma	A	C	-	0.002843	0.0005126	2.927E-08	UKBB	European
TNFSF8	rs3181368	chr9:117665931	2016	173480	2	Eosinophil percentage of white cells	A	T	0.5974	0.02073	0.00363	1.129E-08	3E+07	European
TNFSF8	rs3181368	chr9:117665931	2016	173480	2	Eosinophil percentage of granulocytes	A	T	0.5974	0.02197	0.003645	1.659E-09	3E+07	European
TNFSF8	rs3181368	chr9:117665931	2016	173480	2	Neutrophil percentage of granulocytes	A	T	0.5974	-0.02147	0.003645	3.872E-09	3E+07	European
TNFSF8	rs3181367	chr9:117666678	2016	173480	2	Eosinophil count	A	G	0.4463	-0.02251	0.003574	3.021E-10	3E+07	European
TNFSF8	rs3181367	chr9:117666678	2016	173480	2	Sum eosinophil basophil counts	A	G	0.4463	-0.02152	0.003578	1.814E-09	3E+07	European
TNFSF8	rs3181367	chr9:117666678	2016	173480	2	Eosinophil percentage of white cells	A	G	0.4463	-0.02508	0.003571	2.172E-12	3E+07	European
TNFSF8	rs3181367	chr9:117666678	2016	173480	2	Eosinophil percentage of granulocytes	A	G	0.4463	-0.0258	0.003586	6.224E-13	3E+07	European
TNFSF8	rs3181367	chr9:117666678	2016	173480	2	Neutrophil percentage of granulocytes	A	G	0.4463	0.02521	0.003586	2.079E-12	3E+07	European
TNFSF8	rs3181367	chr9:117666678	2012	963	-	Primary biliary cirrhosis	A	G	0.4463	NA	NA	3.26E-08	2E+07	East Asian
TNFSF8	rs3181366	chr9:117666776	2016	173480	2	Eosinophil percentage of white cells	A	G	0.4016	-0.02075	0.003628	1.062E-08	3E+07	European
TNFSF8	rs3181366	chr9:117666776	2016	173480	2	Eosinophil percentage of granulocytes	A	G	0.4016	-0.02206	0.003644	1.398E-09	3E+07	European
TNFSF8	rs3181366	chr9:117666776	2016	173480	2	Neutrophil percentage of granulocytes	A	G	0.4016	0.02149	0.003644	3.697E-09	3E+07	European
TNFSF8	rs3181365	chr9:117666924	2016	173480	2	Eosinophil count	A	T	0.5537	0.02301	0.003578	1.264E-10	3E+07	European
TNFSF8	rs3181365	chr9:117666924	2016	173480	2	Sum eosinophil basophil counts	A	T	0.5537	0.02202	0.003582	7.837E-10	3E+07	European
TNFSF8	rs3181365	chr9:117666924	2016	173480	2	Eosinophil percentage of white cells	A	T	0.5537	0.02555	0.003574	8.703E-13	3E+07	European
TNFSF8	rs3181365	chr9:117666924	2016	173480	2	Eosinophil percentage of granulocytes	A	T	0.5537	0.02623	0.003589	2.735E-13	3E+07	European
TNFSF8	rs3181365	chr9:117666924	2016	173480	2	Neutrophil percentage of granulocytes	A	T	0.5537	-0.02558	0.003589	1.03E-12	3E+07	European
TNFSF8	rs3181364	chr9:117667161	2016	173480	2	Eosinophil percentage of white cells	A	G	0.5984	0.0208	0.003628	9.843E-09	3E+07	European
TNFSF8	rs3181364	chr9:117667161	2016	173480	2	Eosinophil percentage of granulocytes	A	G	0.5984	0.02212	0.003644	1.283E-09	3E+07	European
TNFSF8	rs3181364	chr9:117667161	2016	173480	2	Neutrophil percentage of granulocytes	A	G	0.5984	-0.02153	0.003644	3.476E-09	3E+07	European
TNFSF8	rs3181363	chr9:117667242	2016	173480	2	Eosinophil percentage of white cells	T	C	0.4016	-0.02087	0.003629	8.917E-09	3E+07	European
TNFSF8	rs3181363	chr9:117667242	2016	173480	2	Eosinophil percentage of granulocytes	T	C	0.4016	-0.02218	0.003645	1.163E-09	3E+07	European
TNFSF8	rs3181363	chr9:117667242	2016	173480	2	Neutrophil percentage of granulocytes	T	C	0.4016	0.02156	0.003645	3.301E-09	3E+07	European
TNFSF8	rs3181362	chr9:117667443	2012	3761	-	Crohns disease	T	C	0.9553	NA	NA	6.38E-10	2E+07	East Asian
TNFSF8	rs3181197	chr9:117667771	2016	173480	2	Eosinophil count	T	C	0.5537	0.02274	0.003576	2.029E-10	3E+07	European
TNFSF8	rs3181197	chr9:117667771	2016	173480	2	Sum eosinophil basophil counts	T	C	0.5537	0.02178	0.00358	1.183E-09	3E+07	European
TNFSF8	rs3181197	chr9:117667771	2016	173480	2	Eosinophil percentage of white cells	T	C	0.5537	0.02533	0.003573	1.342E-12	3E+07	European
TNFSF8	rs3181197	chr9:117667771	2016	173480	2	Eosinophil percentage of granulocytes	T	C	0.5537	0.02603	0.003588	4.037E-13	3E+07	European
TNFSF8	rs3181197	chr9:117667771	2016	173480	2	Neutrophil percentage of granulocytes	T	C	0.5537	-0.02545	0.003588	1.318E-12	3E+07	European
TNFSF8	rs3181195	chr9:117668142	2016	173480	2	Eosinophil percentage of white cells	T	C	0.4016	-0.02082	0.003632	9.872E-09	3E+07	European
TNFSF8	rs3181195	chr9:117668142	2016	173480	2	Eosinophil percentage of granulocytes	T	C	0.4016	-0.02211	0.003647	1.338E-09	3E+07	European
TNFSF8	rs3181195	chr9:117668142	2016	173480	2	Neutrophil percentage of granulocytes	T	C	0.4016	0.02152	0.003648	3.66E-09	3E+07	European
TNFSF8	rs1322054	chr9:117669299	2016	173480	2	Eosinophil count	A	G	0.5537	0.02281	0.003579	1.831E-10	3E+07	European
TNFSF8	rs1322054	chr9:117669299	2016	173480	2	Sum eosinophil basophil counts	A	G	0.5537	0.02184	0.003583	1.081E-09	3E+07	European
TNFSF8	rs1322054	chr9:117669299	2016	173480	2	Eosinophil percentage of white cells	A	G	0.5537	0.02531	0.003575	1.447E-12	3E+07	European
TNFSF8	rs1322054	chr9:117669299	2016	173480	2	Eosinophil percentage of granulocytes	A	G	0.5537	0.02606	0.003591	3.954E-13	3E+07	European
TNFSF8	rs1322054	chr9:117669299	2016	173480	2	Neutrophil percentage of granulocytes	A	G	0.5537	-0.02545	0.003591	1.358E-12	3E+07	European
TNFSF8	rs1322054	chr9:117669299	2005	846	-	Crohns disease	A	G	0.5537	NA	NA	9.13E-09	2E+07	East Asian
TNFSF8	rs3789882	chr9:117669699	2005	846	-	Crohns disease	A	T	0.9553	NA	NA	4.45E-09	2E+07	East Asian
TNFSF8	rs17292087	chr9:117669814	2016	173480	2	Eosinophil percentage of white cells	C	G	0.5974	0.02072	0.003633	1.176E-08	3E+07	European
TNFSF8	rs17292087	chr9:117669814	2016	173480	2	Eosinophil percentage of granulocytes	C	G	0.5974	0.02194	0.003649	1.828E-09	3E+07	European
TNFSF8	rs17292087	chr9:117669814	2016	173480	2	Neutrophil percentage of granulocytes	C	G	0.5974	-0.02137	0.003649	4.739E-09	3E+07	European
TNFSF8	rs12337739	chr9:117670664	2016	173480	2	Eosinophil count	A	T	0.5517	0.02286	0.003582	1.741E-10	3E+07	European
TNFSF8	rs12337739	chr9:117670664	2016	173480	2	Sum eosinophil basophil counts	A	T	0.5517	0.02182	0.003586	1.161E-09	3E+07	European
TNFSF8	rs12337739	chr9:117670664	2016	173480	2	Eosinophil percentage of white cells	A	T	0.5517	0.02543	0.003579	1.2E-12	3E+07	European
TNFSF8	rs12337739	chr9:117670664	2016	173480	2	Eosinophil percentage of granulocytes	A	T	0.5517	0.02619	0.003594	3.185E-13	3E+07	European
TNFSF8	rs12337739	chr9:117670664	2016	173480	2	Neutrophil percentage of granulocytes	A	T	0.5517	-0.02555	0.003594	1.165E-12	3E+07	European
TNFSF8	rs1322056	chr9:117672760	2016	173480	2	Eosinophil count	T	C	0.5527	0.02259	0.00358	2.812E-10	3E+07	European
TNFSF8	rs1322056	chr9:117672760	2016	173480	2	Sum eosinophil basophil counts	T	C	0.5527	0.02164	0.003584	1.566E-09	3E+07	European
TNFSF8	rs1322056	chr9:117672760	2016	173480	2	Eosinophil percentage of white cells	T	C	0.5527	0.0252	0.003577	1.837E-12	3E+07	European

TNFSF8	rs1322056	chr9:117672760	2016	173480	2	Eosinophil percentage of granulocytes	T	C	0.5527	0.02595	0.003592	5.072E-13	3E+07	European
TNFSF8	rs1322056	chr9:117672760	2016	173480	2	Neutrophil percentage of granulocytes	T	C	0.5527	-0.02539	0.003592	1.581E-12	3E+07	European
TNFSF8	rs117601227	chr9:117673793	2017	7637	1	Cause of death: sigmoid colon	T	G	0.997	-0.0545	0.009457	8.593E-09	UKBB	European
TNFSF8	rs7028578	chr9:117674814	2016	173480	2	Eosinophil percentage of white cells	T	C	0.4036	-0.02068	0.00363	1.222E-08	3E+07	European
TNFSF8	rs7028578	chr9:117674814	2016	173480	2	Eosinophil percentage of granulocytes	T	C	0.4036	-0.022	0.003646	1.612E-09	3E+07	European
TNFSF8	rs7028578	chr9:117674814	2016	173480	2	Neutrophil percentage of granulocytes	T	C	0.4036	0.02138	0.003646	4.531E-09	3E+07	European
TNFSF8	rs2208640	chr9:117675454	2016	173480	2	Eosinophil percentage of white cells	A	G	0.5954	0.02059	0.003633	1.439E-08	3E+07	European
TNFSF8	rs2208640	chr9:117675454	2016	173480	2	Eosinophil percentage of granulocytes	A	G	0.5954	0.02191	0.003648	1.917E-09	3E+07	European
TNFSF8	rs2208640	chr9:117675454	2016	173480	2	Neutrophil percentage of granulocytes	A	G	0.5954	-0.0213	0.003648	5.31E-09	3E+07	European
TNFSF8	rs927373	chr9:117675813	2016	173480	2	Eosinophil percentage of white cells	A	G	0.4046	-0.02071	0.003631	1.181E-08	3E+07	European
TNFSF8	rs927373	chr9:117675813	2016	173480	2	Eosinophil percentage of granulocytes	A	G	0.4046	-0.02202	0.003647	1.574E-09	3E+07	European
TNFSF8	rs927373	chr9:117675813	2016	173480	2	Neutrophil percentage of granulocytes	A	G	0.4046	0.02143	0.003647	4.234E-09	3E+07	European
TNFSF8	rs548286330	chr9:117676009	2017	337159	1	Treatment with priadel 200mg m or r tablet	A	C	-	-0.003005	0.0005216	8.362E-09	UKBB	European
TNFSF8	rs140014926	chr9:117676261	2017	7637	1	Cause of death: ischaemic cardiomyopathy	A	G	0.002	0.04562	0.007897	7.894E-09	UKBB	European
TNFSF8	rs10817682	chr9:117676314	2016	173480	2	Eosinophil count	T	C	0.5507	0.02294	0.003581	1.493E-10	3E+07	European
TNFSF8	rs10817682	chr9:117676314	2016	173480	2	Sum eosinophil basophil counts	T	C	0.5507	0.02201	0.003586	8.27E-10	3E+07	European
TNFSF8	rs10817682	chr9:117676314	2016	173480	2	Eosinophil percentage of white cells	T	C	0.5507	0.02545	0.003578	1.135E-12	3E+07	European
TNFSF8	rs10817682	chr9:117676314	2016	173480	2	Eosinophil percentage of granulocytes	T	C	0.5507	0.02627	0.003594	2.662E-13	3E+07	European
TNFSF8	rs10817682	chr9:117676314	2016	173480	2	Neutrophil percentage of granulocytes	T	C	0.5507	-0.02574	0.003594	7.85E-13	3E+07	European
TNFSF8	rs12352646	chr9:117676518	2016	173480	2	Eosinophil count	T	C	0.5507	0.02309	0.003584	1.173E-10	3E+07	European
TNFSF8	rs12352646	chr9:117676518	2016	173480	2	Sum eosinophil basophil counts	T	C	0.5507	0.02206	0.003588	7.863E-10	3E+07	European
TNFSF8	rs12352646	chr9:117676518	2016	173480	2	Eosinophil percentage of white cells	T	C	0.5507	0.02539	0.00358	1.329E-12	3E+07	European
TNFSF8	rs12352646	chr9:117676518	2016	173480	2	Eosinophil percentage of granulocytes	T	C	0.5507	0.02618	0.003596	3.349E-13	3E+07	European
TNFSF8	rs12352646	chr9:117676518	2016	173480	2	Neutrophil percentage of granulocytes	T	C	0.5507	-0.0255	0.003596	1.326E-12	3E+07	European
TNFSF8	rs10982449	chr9:117676541	2016	173480	2	Eosinophil percentage of white cells	A	G	0.5944	0.02087	0.003638	9.686E-09	3E+07	European
TNFSF8	rs10982449	chr9:117676541	2016	173480	2	Eosinophil percentage of granulocytes	A	G	0.5944	0.02228	0.003654	1.084E-09	3E+07	European
TNFSF8	rs10982449	chr9:117676541	2016	173480	2	Neutrophil percentage of granulocytes	A	G	0.5944	-0.02163	0.003654	3.233E-09	3E+07	European
TNFSF8	rs12347977	chr9:117676830	2012	963	1	Primary biliary cholangitis	A	G	0.4493	0.4095	NA	0	2E+07	East Asian
TNFSF8	rs12347977	chr9:117676830	2016	173480	2	Eosinophil count	A	G	0.4493	-0.02282	0.003594	2.169E-10	3E+07	European
TNFSF8	rs12347977	chr9:117676830	2016	173480	2	Sum eosinophil basophil counts	A	G	0.4493	-0.02176	0.003598	1.461E-09	3E+07	European
TNFSF8	rs12347977	chr9:117676830	2016	173480	2	Eosinophil percentage of white cells	A	G	0.4493	-0.02543	0.00359	1.408E-12	3E+07	European
TNFSF8	rs12347977	chr9:117676830	2016	173480	2	Eosinophil percentage of granulocytes	A	G	0.4493	-0.02619	0.003606	3.763E-13	3E+07	European
TNFSF8	rs12347977	chr9:117676830	2016	173480	2	Neutrophil percentage of granulocytes	A	G	0.4493	0.02557	0.003606	1.328E-12	3E+07	European
TNFSF8	rs12338765	chr9:117676833	2016	173480	2	Eosinophil count	A	C	0.5507	0.02274	0.003594	2.501E-10	3E+07	European
TNFSF8	rs12338765	chr9:117676833	2016	173480	2	Sum eosinophil basophil counts	A	C	0.5507	0.02172	0.003598	1.568E-09	3E+07	European
TNFSF8	rs12338765	chr9:117676833	2016	173480	2	Eosinophil percentage of white cells	A	C	0.5507	0.02541	0.003591	1.482E-12	3E+07	European
TNFSF8	rs12338765	chr9:117676833	2016	173480	2	Eosinophil percentage of granulocytes	A	C	0.5507	0.02621	0.003606	3.633E-13	3E+07	European
TNFSF8	rs12338765	chr9:117676833	2016	173480	2	Neutrophil percentage of granulocytes	A	C	0.5507	-0.02566	0.003606	1.112E-12	3E+07	European
TNFSF8	rs7037640	chr9:117676931	2016	173480	2	Eosinophil percentage of white cells	C	G	0.4046	-0.02086	0.003632	9.268E-09	3E+07	European
TNFSF8	rs7037640	chr9:117676931	2016	173480	2	Eosinophil percentage of granulocytes	C	G	0.4046	-0.02222	0.003648	1.119E-09	3E+07	European
TNFSF8	rs7037640	chr9:117676931	2016	173480	2	Neutrophil percentage of granulocytes	C	G	0.4046	0.02162	0.003648	3.113E-09	3E+07	European
TNFSF8	rs4978611	chr9:117677305	2016	173480	2	Eosinophil count	A	C	0.5507	0.02304	0.003582	1.259E-10	3E+07	European
TNFSF8	rs4978611	chr9:117677305	2016	173480	2	Sum eosinophil basophil counts	A	C	0.5507	0.02203	0.003586	8.157E-10	3E+07	European
TNFSF8	rs4978611	chr9:117677305	2016	173480	2	Eosinophil percentage of white cells	A	C	0.5507	0.02554	0.003579	9.604E-13	3E+07	European
TNFSF8	rs4978611	chr9:117677305	2016	173480	2	Eosinophil percentage of granulocytes	A	C	0.5507	0.02631	0.003594	2.498E-13	3E+07	European
TNFSF8	rs4978611	chr9:117677305	2016	173480	2	Neutrophil percentage of granulocytes	A	C	0.5507	-0.0257	0.003594	8.67E-13	3E+07	European
TNFSF8	rs78518873	chr9:117677406	2017	7637	1	Cause of death: alcoholic hepatitis	A	G	0.008	0.02733	0.004993	4.547E-08	UKBB	European
TNFSF8	rs150335404	chr9:117677617	2017	7637	1	Cause of death: malignant neoplasms of independent multiple sites	A	G	0.004	0.04571	0.006529	2.763E-12	UKBB	European
TNFSF8	rs3838334	chr9:117678053	2016	173480	2	Eosinophil count	A	AG	0.5507	0.02289	0.003582	1.649E-10	3E+07	European
TNFSF8	rs3838334	chr9:117678053	2016	173480	2	Sum eosinophil basophil counts	A	AG	0.5507	0.02187	0.003586	1.075E-09	3E+07	European
TNFSF8	rs3838334	chr9:117678053	2016	173480	2	Eosinophil percentage of white cells	A	AG	0.5507	0.02538	0.003579	1.329E-12	3E+07	European
TNFSF8	rs3838334	chr9:117678053	2016	173480	2	Eosinophil percentage of granulocytes	A	AG	0.5507	0.02619	0.003594	3.166E-13	3E+07	European
TNFSF8	rs3838334	chr9:117678053	2016	173480	2	Neutrophil percentage of granulocytes	A	AG	0.5507	-0.02554	0.003594	1.182E-12	3E+07	European
TNFSF8	rs3789879	chr9:117678236	2016	173480	2	Eosinophil count	T	C	0.5507	0.02286	0.003582	1.742E-10	3E+07	European
TNFSF8	rs3789879	chr9:117678236	2016	173480	2	Sum eosinophil basophil counts	T	C	0.5507	0.02185	0.003586	1.105E-09	3E+07	European
TNFSF8	rs3789879	chr9:117678236	2016	173480	2	Eosinophil percentage of white cells	T	C	0.5507	0.02536	0.003579	1.373E-12	3E+07	European
TNFSF8	rs3789879	chr9:117678236	2016	173480	2	Eosinophil percentage of granulocytes	T	C	0.5507	0.02613	0.003594	3.549E-13	3E+07	European
TNFSF8	rs3789879	chr9:117678236	2016	173480	2	Neutrophil percentage of granulocytes	T	C	0.5507	-0.02551	0.003594	1.272E-12	3E+07	European

TNFSF8	rs3789879	chr9:117678236	2005	846	-	Crohns disease	T	C	0.5507	NA	NA	3.7E-09	2E+07	East Asian
TNFSF8	rs927374	chr9:117679370	2016	173480	2	Eosinophil count	C	G	0.4493	-0.02291	0.003581	1.582E-10	3E+07	European
TNFSF8	rs927374	chr9:117679370	2016	173480	2	Sum eosinophil basophil counts	C	G	0.4493	-0.02196	0.003585	9.006E-10	3E+07	European
TNFSF8	rs927374	chr9:117679370	2016	173480	2	Eosinophil percentage of white cells	C	G	0.4493	-0.02542	0.003578	1.217E-12	3E+07	European
TNFSF8	rs927374	chr9:117679370	2016	173480	2	Eosinophil percentage of granulocytes	C	G	0.4493	-0.02613	0.003593	3.572E-13	3E+07	European
TNFSF8	rs927374	chr9:117679370	2016	173480	2	Neutrophil percentage of granulocytes	C	G	0.4493	0.02557	0.003593	1.103E-12	3E+07	European
TNFSF8	rs3789878	chr9:117679730	2017	7637	1	Cause of death: alcoholic hepatitis	C	G	0.008	0.02742	0.005001	4.313E-08	UKBB	European
TNFSF8	rs1885383	chr9:117679802	2005	846	-	Crohns disease	A	G	0.0447	NA	NA	1.61E-09	2E+07	East Asian
TNFSF8	rs527725149	chr9:117680645	2017	7637	1	Cause of death: chronic obstructive pulmonary disease with acute exacerbation, unspecified	A	G	0.999	-0.1183	0.01543	1.964E-14	UKBB	European
TNFSF8	rs527725149	chr9:117680645	2017	7637	1	Cause of death: car occupant injured in unspecified traffic accident	A	G	0.999	-0.05915	0.007975	1.325E-13	UKBB	European
TNFSF8	rs11322814	chr9:117680733	2016	173480	2	Eosinophil count	C	CT	0.4622	-0.02131	0.003652	5.401E-09	3E+07	European
TNFSF8	rs11322814	chr9:117680733	2016	173480	2	Sum eosinophil basophil counts	C	CT	0.4622	-0.0202	0.003657	3.294E-08	3E+07	European
TNFSF8	rs11322814	chr9:117680733	2016	173480	2	Eosinophil percentage of white cells	C	CT	0.4622	-0.02352	0.003649	1.14E-10	3E+07	European
TNFSF8	rs11322814	chr9:117680733	2016	173480	2	Eosinophil percentage of granulocytes	C	CT	0.4622	-0.02427	0.003664	3.513E-11	3E+07	European
TNFSF8	rs11322814	chr9:117680733	2016	173480	2	Neutrophil percentage of granulocytes	C	CT	0.4622	0.02354	0.003665	1.336E-10	3E+07	European
TNFSF8	rs761568068	chr9:117680911	2017	337159	1	Treatment with zirtek allergy 10mg tablet	T	C	-	0.003493	0.0006082	9.284E-09	UKBB	European
TNFSF8	rs761568068	chr9:117680911	2017	7637	1	Cause of death: intracranial haemorrhage, unspecified	T	C	-	0.075	0.0123	1.128E-09	UKBB	European
TNFSF8	rs78229723	chr9:117681377	2017	7637	1	Cause of death: alcoholic hepatitis	T	C	0.008	0.02735	0.004994	4.476E-08	UKBB	European
TNFSF8	rs77474901	chr9:117681562	2017	7637	1	Cause of death: alcoholic hepatitis	A	G	0.992	-0.02733	0.004994	4.558E-08	UKBB	European
TNFSF8	rs10982450	chr9:117681870	2016	173480	2	Eosinophil percentage of white cells	T	G	0.4046	-0.02078	0.003631	1.046E-08	3E+07	European
TNFSF8	rs10982450	chr9:117681870	2016	173480	2	Eosinophil percentage of granulocytes	T	G	0.4046	-0.02213	0.003647	1.301E-09	3E+07	European
TNFSF8	rs10982450	chr9:117681870	2016	173480	2	Neutrophil percentage of granulocytes	T	G	0.4046	0.02156	0.003647	3.419E-09	3E+07	European
TNFSF8	rs7872878	chr9:117682077	2016	173480	2	Eosinophil count	C	G	0.4414	-0.02288	0.003586	1.775E-10	3E+07	European
TNFSF8	rs7872878	chr9:117682077	2016	173480	2	Sum eosinophil basophil counts	C	G	0.4414	-0.02189	0.00359	1.086E-09	3E+07	European
TNFSF8	rs7872878	chr9:117682077	2016	173480	2	Eosinophil percentage of white cells	C	G	0.4414	-0.02506	0.003583	2.649E-12	3E+07	European
TNFSF8	rs7872878	chr9:117682077	2016	173480	2	Eosinophil percentage of granulocytes	C	G	0.4414	-0.02577	0.003598	8.013E-13	3E+07	European
TNFSF8	rs7872878	chr9:117682077	2016	173480	2	Neutrophil percentage of granulocytes	C	G	0.4414	0.02515	0.003598	2.744E-12	3E+07	European
TNFSF8	rs10982451	chr9:117682492	2016	173480	2	Eosinophil percentage of white cells	T	C	0.4046	-0.02077	0.003632	1.069E-08	3E+07	European
TNFSF8	rs10982451	chr9:117682492	2016	173480	2	Eosinophil percentage of granulocytes	T	C	0.4046	-0.02214	0.003647	1.28E-09	3E+07	European
TNFSF8	rs10982451	chr9:117682492	2016	173480	2	Neutrophil percentage of granulocytes	T	C	0.4046	0.02157	0.003648	3.37E-09	3E+07	European
TNFSF8	rs765580378	chr9:117683794	2017	337159	1	Treatment with zirtek allergy 10mg tablet	A	G	-	-0.00328	0.0005923	3.065E-08	UKBB	European
TNFSF8	rs1322058	chr9:117684547	2016	173480	2	Eosinophil percentage of white cells	T	C	0.4046	-0.02082	0.003632	9.801E-09	3E+07	European
TNFSF8	rs1322058	chr9:117684547	2016	173480	2	Eosinophil percentage of granulocytes	T	C	0.4046	-0.02219	0.003647	1.178E-09	3E+07	European
TNFSF8	rs1322058	chr9:117684547	2016	173480	2	Neutrophil percentage of granulocytes	T	C	0.4046	0.0216	0.003648	3.212E-09	3E+07	European
TNFSF8	rs4979472	chr9:117685398	2016	173480	2	Eosinophil count	A	G	0.5517	0.02301	0.003581	1.299E-10	3E+07	European
TNFSF8	rs4979472	chr9:117685398	2016	173480	2	Sum eosinophil basophil counts	A	G	0.5517	0.02202	0.003585	8.059E-10	3E+07	European
TNFSF8	rs4979472	chr9:117685398	2016	173480	2	Eosinophil percentage of white cells	A	G	0.5517	0.02553	0.003577	9.657E-13	3E+07	European
TNFSF8	rs4979472	chr9:117685398	2016	173480	2	Eosinophil percentage of granulocytes	A	G	0.5517	0.02634	0.003593	2.29E-13	3E+07	European
TNFSF8	rs4979472	chr9:117685398	2016	173480	2	Neutrophil percentage of granulocytes	A	G	0.5517	-0.02576	0.003593	7.568E-13	3E+07	European
TNFSF8	rs10817683	chr9:117686457	2016	173480	2	Eosinophil count	A	G	0.5596	0.02298	0.003586	1.469E-10	3E+07	European
TNFSF8	rs10817683	chr9:117686457	2016	173480	2	Sum eosinophil basophil counts	A	G	0.5596	0.02201	0.003591	8.777E-10	3E+07	European
TNFSF8	rs10817683	chr9:117686457	2016	173480	2	Eosinophil percentage of white cells	A	G	0.5596	0.02517	0.003583	2.138E-12	3E+07	European
TNFSF8	rs10817683	chr9:117686457	2016	173480	2	Eosinophil percentage of granulocytes	A	G	0.5596	0.02588	0.003598	6.428E-13	3E+07	European
TNFSF8	rs10817683	chr9:117686457	2016	173480	2	Neutrophil percentage of granulocytes	A	G	0.5596	-0.02529	0.003598	2.086E-12	3E+07	European
TNFSF8	rs77428261	chr9:117686601	2017	7637	1	Cause of death: alcoholic hepatitis	T	C	0.992	-0.02745	0.005003	4.23E-08	UKBB	European
TNFSF8	rs10982453	chr9:117686732	2016	173480	2	Eosinophil percentage of white cells	A	T	0.5974	0.02092	0.003632	8.337E-09	3E+07	European
TNFSF8	rs10982453	chr9:117686732	2016	173480	2	Eosinophil percentage of granulocytes	A	T	0.5974	0.02231	0.003648	9.634E-10	3E+07	European
TNFSF8	rs10982453	chr9:117686732	2016	173480	2	Neutrophil percentage of granulocytes	A	T	0.5974	-0.02172	0.003648	2.592E-09	3E+07	European
TNFSF8	rs148342441	chr9:117686798	2017	7637	1	Cause of death: malignant neoplasm of ureter	A	T	0.005	0.07101	0.009377	4.099E-14	UKBB	European
TNFSF8	rs148342441	chr9:117686798	2017	7637	1	Cause of death: acute and subacute infective endocarditis	A	T	0.005	0.06104	0.006987	2.928E-18	UKBB	European
TNFSF8	rs148342441	chr9:117686798	2017	7637	1	Cause of death: calculus of gallbladder without cholecystitis	A	T	0.005	0.05914	0.009393	3.212E-10	UKBB	European
TNFSF8	rs148342441	chr9:117686798	2017	337199	1	Mesothelioma	A	T	0.005	0.002903	0.0005024	7.509E-09	UKBB	European
TNFSF8	rs148342441	chr9:117686798	2017	337199	1	Other disorders of gingiva and edentulous alveolar ridge	A	T	0.005	0.004212	0.0007603	3.04E-08	UKBB	European
TNFSF8	rs148342441	chr9:117686798	2017	337199	1	Fracture of skull and facial bones	A	T	0.005	0.01144	0.002044	2.16E-08	UKBB	European
TNFSF8	rs10982454	chr9:117686847	2016	173480	2	Eosinophil percentage of white cells	A	C	0.4026	-0.02095	0.003632	7.959E-09	3E+07	European
TNFSF8	rs10982454	chr9:117686847	2016	173480	2	Eosinophil percentage of granulocytes	A	C	0.4026	-0.02233	0.003648	9.313E-10	3E+07	European
TNFSF8	rs10982454	chr9:117686847	2016	173480	2	Neutrophil percentage of granulocytes	A	C	0.4026	0.02176	0.003648	2.438E-09	3E+07	European
TNFSF8	rs74680773	chr9:117686858	2017	7637	1	Cause of death: alcoholic hepatitis	C	G	0.992	-0.02745	0.005003	4.224E-08	UKBB	European

TNFSF8	rs10982455	chr9:117687036	2016	173480	2	Eosinophil percentage of white cells	A	C	0.4036	-0.02103	0.003633	7.06E-09	3E+07	European
TNFSF8	rs10982455	chr9:117687036	2016	173480	2	Eosinophil percentage of granulocytes	A	C	0.4036	-0.02238	0.003649	8.595E-10	3E+07	European
TNFSF8	rs10982455	chr9:117687036	2016	173480	2	Neutrophil percentage of granulocytes	A	C	0.4036	0.02181	0.003649	2.282E-09	3E+07	European
TNFSF8	rs7036962	chr9:117687151	2016	173480	2	Eosinophil percentage of white cells	T	G	0.4026	-0.02098	0.003633	7.736E-09	3E+07	European
TNFSF8	rs7036962	chr9:117687151	2016	173480	2	Eosinophil percentage of granulocytes	T	G	0.4026	-0.02234	0.003649	9.19E-10	3E+07	European
TNFSF8	rs7036962	chr9:117687151	2016	173480	2	Neutrophil percentage of granulocytes	T	G	0.4026	0.02176	0.003649	2.465E-09	3E+07	European
TNFSF8	rs10817684	chr9:117689184	2016	173480	2	Eosinophil percentage of white cells	A	G	0.5984	0.02136	0.00364	4.427E-09	3E+07	European
TNFSF8	rs10817684	chr9:117689184	2016	173480	2	Eosinophil percentage of granulocytes	A	G	0.5984	0.02275	0.003656	4.914E-10	3E+07	European
TNFSF8	rs10817684	chr9:117689184	2016	173480	2	Neutrophil percentage of granulocytes	A	G	0.5984	-0.02219	0.003656	1.29E-09	3E+07	European
TNFSF8	rs10817685	chr9:117689305	2016	173480	2	Eosinophil count	T	C	0.4394	-0.02336	0.00359	7.643E-11	3E+07	European
TNFSF8	rs10817685	chr9:117689305	2016	173480	2	Sum eosinophil basophil counts	T	C	0.4394	-0.02236	0.003594	4.922E-10	3E+07	European
TNFSF8	rs10817685	chr9:117689305	2016	173480	2	Eosinophil percentage of white cells	T	C	0.4394	-0.02551	0.003587	1.137E-12	3E+07	European
TNFSF8	rs10817685	chr9:117689305	2016	173480	2	Eosinophil percentage of granulocytes	T	C	0.4394	-0.0262	0.003602	3.491E-13	3E+07	European
TNFSF8	rs10817685	chr9:117689305	2016	173480	2	Neutrophil percentage of granulocytes	T	C	0.4394	0.02557	0.003602	1.254E-12	3E+07	European
TNFSF8	rs78582862	chr9:117689878	2017	7637	1	Cause of death: alcoholic hepatitis	C	G	0.992	-0.02745	0.005003	4.228E-08	UKBB	European
TNFSF8	rs759219652	chr9:117690064	2017	7637	1	Cause of death: chronic myeloid leukaemia	T	C	-	0.03869	0.006959	2.792E-08	UKBB	European
TNFSF8	rs10982456	chr9:117690758	2016	173480	2	Eosinophil count	T	C	0.5527	0.02352	0.003585	5.322E-11	3E+07	European
TNFSF8	rs10982456	chr9:117690758	2016	173480	2	Sum eosinophil basophil counts	T	C	0.5527	0.02255	0.003589	3.296E-10	3E+07	European
TNFSF8	rs10982456	chr9:117690758	2016	173480	2	Eosinophil percentage of white cells	T	C	0.5527	0.02588	0.003582	4.957E-13	3E+07	European
TNFSF8	rs10982456	chr9:117690758	2016	173480	2	Eosinophil percentage of granulocytes	T	C	0.5527	0.02664	0.003597	1.302E-13	3E+07	European
TNFSF8	rs10982456	chr9:117690758	2016	173480	2	Neutrophil percentage of granulocytes	T	C	0.5527	-0.02604	0.003597	4.527E-13	3E+07	European
TNFSF8	rs10982456	chr9:117690758	2016	-	-	Eosinophil percentage of white cells	T	C	0.5527	-0.02588	0.003582	5E-13	3E+07	European
TNFSF8	rs10982456	chr9:117690758	2016	-	-	Neutrophil percentage of granulocytes	T	C	0.5527	-0.02604	0.003604	5E-13	3E+07	European
TNFSF8	rs10982456	chr9:117690758	2016	-	-	Eosinophil percentage of granulocytes	T	C	0.5527	-0.02664	0.00358	1E-13	3E+07	European
TNFSF8	rs2181032	chr9:117690864	2017	7637	1	Cause of death: alcoholic hepatitis	T	C	0.008	0.02738	0.004997	4.405E-08	UKBB	European
TNFSF8	rs1006025	chr9:117691201	2016	173480	2	Eosinophil percentage of white cells	T	C	0.4016	-0.02143	0.003637	3.762E-09	3E+07	European
TNFSF8	rs1006025	chr9:117691201	2016	173480	2	Eosinophil percentage of granulocytes	T	C	0.4016	-0.02279	0.003653	4.416E-10	3E+07	European
TNFSF8	rs1006025	chr9:117691201	2016	173480	2	Neutrophil percentage of granulocytes	T	C	0.4016	0.02218	0.003653	1.265E-09	3E+07	European
TNFSF8	rs1006026	chr9:117691270	2016	173480	2	Eosinophil count	A	G	0.5606	0.02344	0.003593	6.856E-11	3E+07	European
TNFSF8	rs1006026	chr9:117691270	2016	173480	2	Sum eosinophil basophil counts	A	G	0.5606	0.02243	0.003597	4.526E-10	3E+07	European
TNFSF8	rs1006026	chr9:117691270	2016	173480	2	Eosinophil percentage of white cells	A	G	0.5606	0.02556	0.003589	1.078E-12	3E+07	European
TNFSF8	rs1006026	chr9:117691270	2016	173480	2	Eosinophil percentage of granulocytes	A	G	0.5606	0.0263	0.003605	2.98E-13	3E+07	European
TNFSF8	rs1006026	chr9:117691270	2016	173480	2	Neutrophil percentage of granulocytes	A	G	0.5606	-0.02567	0.003605	1.081E-12	3E+07	European
TNFSF8	rs1006027	chr9:117691313	2016	173480	2	Eosinophil percentage of white cells	T	C	0.5984	0.02134	0.003654	5.174E-09	3E+07	European
TNFSF8	rs1006027	chr9:117691313	2016	173480	2	Eosinophil percentage of granulocytes	T	C	0.5984	0.02267	0.003669	6.475E-10	3E+07	European
TNFSF8	rs1006027	chr9:117691313	2016	173480	2	Neutrophil percentage of granulocytes	T	C	0.5984	-0.02209	0.003669	1.729E-09	3E+07	European
TNFSF8	rs3181360	chr9:117691558	2012	3761	-	Crohns disease	T	C	-	NA	NA	7.94E-10	2E+07	East Asian
TNFSF8	rs766975966	chr9:117691755	2017	7637	1	Cause of death: multisystem degeneration	A	C	-	0.06497	0.008315	6.278E-15	UKBB	European
TNFSF8	rs766975966	chr9:117691755	2017	7637	1	Cause of death: intracranial haemorrhage, unspecified	A	C	-	0.06373	0.01141	2.386E-08	UKBB	European
TNFSF8	rs148978384	chr9:117692670	2017	7637	1	Cause of death: chronic myeloid leukaemia	T	C	0.008	0.03554	0.004409	8.792E-16	UKBB	European
